# Comparative population genomics reveals the domestication history of the peach, *Prunus persica*, and human influences on perennial fruit crops

**DOI:** 10.1186/s13059-014-0415-1

**Published:** 2014-07-31

**Authors:** Ke Cao, Zhijun Zheng, Lirong Wang, Xin Liu, Gengrui Zhu, Weichao Fang, Shifeng Cheng, Peng Zeng, Changwen Chen, Xinwei Wang, Min Xie, Xiao Zhong, Xiaoli Wang, Pei Zhao, Chao Bian, Yinling Zhu, Jiahui Zhang, Guosheng Ma, Chengxuan Chen, Yanjun Li, Fengge Hao, Yong Li, Guodong Huang, Yuxiang Li, Haiyan Li, Jian Guo, Xun Xu, Jun Wang

**Affiliations:** Zhengzhou Fruit Research Institute, Chinese Academy of Agriculture Sciences, Zhengzhou, 450009 China; The Key Laboratory of Biology and Genetic Improvement of Horticultural Crops (Fruit Tree Breeding Technology), Ministry of Agriculture, Zhengzhou, 450009 China; BGI-Shenzhen, Shenzhen, 518083 China; MetaGene Genomics Institute, Hangzhou, 310011 China; State Key Laboratory of Agricultural Genomics, BGI-Shenzhen, Shenzhen, 518083 China; Key Laboratory of Genomics, Ministry of Agriculture, BGI-Shenzhen, Shenzhen, 518083 China

## Abstract

**Background:**

Recently, many studies utilizing next generation sequencing have investigated plant evolution and domestication in annual crops. Peach, *Prunus persica*, is a typical perennial fruit crop that has ornamental and edible varieties. Unlike other fruit crops, cultivated peach includes a large number of phenotypes but few polymorphisms. In this study, we explore the genetic basis of domestication in peach and the influence of humans on its evolution.

**Results:**

We perform large-scale resequencing of 10 wild and 74 cultivated peach varieties, including 9 ornamental, 23 breeding, and 42 landrace lines. We identify 4.6 million SNPs, a large number of which could explain the phenotypic variation in cultivated peach. Population analysis shows a single domestication event, the speciation of *P. persica* from wild peach. Ornamental and edible peach both belong to *P. persica*, along with another geographically separated subgroup, *Prunus ferganensis*.

We identify 147 and 262 genes under edible and ornamental selection, respectively. Some of these genes are associated with important biological features. We perform a population heterozygosity analysis in different plants that indicates that free recombination effects could affect domestication history. By applying artificial selection during the domestication of the peach and facilitating its asexual propagation, humans have caused a sharp decline of the heterozygote ratio of SNPs.

**Conclusions:**

Our analyses enhance our knowledge of the domestication history of perennial fruit crops, and the dataset we generated could be useful for future research on comparative population genomics.

**Electronic supplementary material:**

The online version of this article (doi:10.1186/s13059-014-0415-1) contains supplementary material, which is available to authorized users.

## Background

Plant domestication is an evolutionary process that is influenced by human actions [1]. Artificial selection causes the emergence of cultivated populations that differ markedly from their wild progenitors in morphology and genetics. Perennial species, including woody shrubs, forest trees, and fruit crops, have always had much slower rates of evolution than annual plants because they are propagated clonally. Their long juvenile phases further decrease the number of sexual cycles that they experience [2].

Peach (*Prunus persica*) originated in China as long ago as 3000 BC, according to archaeological evidence [3]. Peach is related to five wild species: *Prunus mira* Koehne, *Prunus davidiana* Franch, *Prunus davidiana* var. *potaninii* Rehd., *Prunus kansuensis* Rehd., and *Prunus ferganensis* Kost. & Riab. These species produce fruits of very poor eating quality except for *P. ferganensis*, although they could be valuable as a source of disease-resistance traits or as rootstocks. Several thousand years of domestication have produced more than 1,000 cultivars of *P. persica* worldwide, with significant phenotypic changes in fruit size, flavor, and flower type. Some variations (flat shape, glabrous surface, double flower, and colorful anther) exist in peach but not in other close fruit species, such as apricot (*Prunus armeniaca*), plum (*Prunus salicina*), apple (*Malus domestica*), and grape (*Vitis vinifera*), although peach has a lower level of genetic variability compared with the other *Prunus* crops due to selfing as well as important bottlenecks in its recent breeding history [4].

Recent genetic and genomic analyses of annual crops, such as tomato (*Solanum lycopersicum*) [5], soybean (*Glycine max*) [6], sorghum (*Sorghum bicolor*) [7], maize (*Zea mays*) [8], and rice (*Oryza sativa*) [9] have greatly advanced our understanding of plant domestication. However, little information is known about the response of genomic variation to artificial selection [10] in perennial plants or the influence of human actions such as the use of a different mode of reproduction.

Peach is a model fruit species for use in comparative and functional genomics because it is a diploid species (2n = 16), and has a small genome (approximately 220 Mb, about twice that of *Arabidopsis*). The reference peach genome released by the International Peach Genome Initiative [11] provided a foundation for population analyses of peach. In order to obtain a comprehensive overview of peach population evolution and domestication in perennial plants, we sequenced 84 peach accessions and identified approximately 4.6 million SNPs and other variations, such as indels (short insertion and deletions of 1 to 5 bp) and structure variations (SVs). We found that cultivated peach is distinct from other plants due to its high ratio of average nonsynonymous versus synonymous nucleotides (Nonsyn/Syn) and the low heterozygous rate of SNPs in cultivated populations. We also analyzed the domestication history and artificial selection of certain genes. The large quantity of variation resources provided here will facilitate modern breeding of peach and related species.

## Results and discussion

### Sequencing and variation calling

We selected 84 peach lines (10% of the germplasm repository), including 3 accessions of *P. mira* Koehne, 4 of *P. davidiana* (Carr.) Franch., 2 of *P. kansuensis* Rehd., 4 of *P. ferganensis* Kost. et Riab., 70 of *P. persica* (L.) Batsch, and 1 of *P. persica* × *P. davidiana* (Table S1 in Additional file 1). These peach lines were chosen from 837 accessions in the National Germplasm Repository of China, which includes more than 80% of the peach varieties worldwide (approximately 1,000 accessions). The 84 samples were chosen on the basis of four key rules (see the 'Sample collection' section in Materials and methods) and represent enormous phenotypic diversity (Tables S2 and S3 in Additional file 1; Figure S1 in Additional file 2). We generated 76.6 gigabase pairs of sequence from the 84 peach accessions using Illumina GA II technology. After mapping the sequencing reads from each sample to the reference genome of ‘Lovell’ peach [11], we obtained an average sequencing depth of approximately 3.2× and average genome coverage of approximately 86.6% (Table S4 in Additional file 1). The mapping rate in different accessions varied from 77% to 97%.

Using the mapping results of the 84 accessions, we identified SNPs of each accession through SOAPsnp. In order to obtain the SNPs/genotypes in the population, we estimated the allele frequencies by a Bayesian method and filtered the SNPs considering the sequencing depth and mapping rate (see the 'Variation detection' section in Materials and methods). We detected a total of 4,567,069 SNPs (Table 1) in the population.

**Table 1:** **Summary of single-nucleotide polymorphisms**

**Groups**	**n**	**Total SNPs**	**Intergenic**	**Untranslated region**		**Intronic**	**Coding sequences**			**Ratio of Nonsyn/Syn**
				**3′ UTR**	**5′ UTR**		**Total**	**Nonsynonymous**	**Synonymous**	
Wild	10	3,381,514	2,433,878	32,943	15,748	550,643	348,302	190,986	157,316	1.21
Ornamental	9	1,065,215	811,075	7,355	4,224	135,238	107,323	66,796	40,527	1.65
Edible	65	2,098,002	1,607,679	14,702	8,445	262,954	204,222	126,717	77,505	1.63
All genotypes	84	4,567,069	3,340,294	41,933	20,911	706,799	457,132	258,902	198,230	1.31

We were interested in determining whether the sample size in each group was suitable for the analysis of genotype/SNPs in the population and whether the sequencing depth was suitable for SNP calling. Therefore, we analyzed the relationship between the identified SNPs and the sample sizes (Figure S2 in Additional file 2) and the relationship between the called SNPs and the sequencing depth (Figure S3 in Additional file 2).

### Quality control checks on heterozygous SNP calling

The total pick depth of all the genotype sites in the 84 samples was approximately 250× (Figure S4 in Additional file 2), which was sufficient to perform a reliable analysis of population SNPs. However, we needed to determine the depth distribution of the SNPs in each accession, especially the heterozygous SNPs. Therefore, we performed a statistical analysis of the SNP depth in each sample (Figure S5 in Additional file 2). The SNP peak depth of homozygous sites was 2× to 4×, whereas the SNP peak depth of heterozygous sites was 4× to 6×. The depths of heterozygous SNPs were higher than those of homozygous SNPs and higher than expected (3×).

In order to confirm that the depths of heterozygous SNPs were higher, we re-checked the mapping results in SNP sites (Figure S6 in Additional file 2). The majority of homozygous SNPs had one to three mapping reads, whereas heterozygous SNPs had many more than three mapping reads. Next, we wished to determine whether the mapping reads on heterozygous SNPs came from repeat regions or homologous sequences (reads that aligned to more than one site in the genome were counted just once, randomly assigned to one match site). We performed a statistical analysis of the SNP depth distributions without the SNPs in repeat regions and homologous sequences (Figure S7 in Additional file 2) and determined that the excess mapping reads on heterozygous SNPs were not from repeat regions or homologous sequences. Therefore, we suspected that the higher depth in heterozygous SNP sites probably occurred in the process of Illumina re-sequencing.

In order to verify that this was the case, we drew the depth of homozygous and heterozygous sites in the total genotype of 84 samples (Figure S8 in Additional file 2). We determined that if a site occurred with a higher heterozygote frequency in the population, the total population depth of the site was higher. We also proposed an hypothesis to explain this phenomenon (Figure S9 in Additional file 2). We think that model 2 in Figure S9 in Additional file 2 could be the reason why the depth of heterozygous SNP sites was higher than the depth of homozygous SNP sites.

### Estimation of accuracy of SNP calling

The variation-calling pipelines that we applied are designed for Illumina sequencing platforms and the general accuracy is between 95 and 99% [6,9,12]. To validate the results of the identified SNPs using Sanger sequencing, we randomly selected 864 homologous SNPs, of which 859 were correct (an accuracy of about 99.4%). Next, we randomly selected 22 heterozygous SNPs, of which 14 were correctly predicted/called (an accuracy of about 63.6%). Moreover, the Sequenom MassArray platform was also applied to verify the SNPs. The results showed that the accuracy of homologous SNPs was 92.2%, and the accuracy of heterozygous SNPs was 76.3%. If the ratio of the heterozygous SNPs was ρ, the mean accuracy could be calculated by ρ × 63.6% + (1 - ρ) × 99.4%. As the total mean ratio of the heterozygous SNPs is 1.552% (the data were modified by curve fitting according to [13] with published simulation data, as shown in Figure S10 in Additional file 2), we calculated a mean accuracy of about 91.6 to 98.9% in our study using the two methods. Moreover, the estimated sensitivity of our variant-calling pipeline could also be found in our gene clone experiment (see the 'Detection of variation' section in Materials and methods). Although the average depth of coverage is low, we estimate that we have identified 71 to 83% of the total number of SNPs with an accuracy of 91.6 to 98.9%.

### Genomic distribution of variations

Of the identified SNPs, 1,226,775 (26.9%) were located in the gene region, and 457,132 SNPs (10.0%) were located in coding sequences (CDSs). Of the SNPs in CDS, 198,230 were synonymous SNPs, and 258,902 were nonsynonymous SNPs, which result in amino acid changes. Thus, the ratio of the number of nonsynonymous to synonymous (Nonsyn/Syn) SNPs in the genome was 1.31 (Table 1), higher than that of *Arabidopsis* (0.83) [14] and similar to that of soybean (1.37) [6] and rice (1.29) [9]. The value was higher in edible (1.63) and ornamental (1.65) peach than in wild peach (1.21). The higher Nonsyn/Syn value at the whole-genome level of cultivated peach is most likely caused by positive selection for these changes [6].

In total, 870,420 indels (Table S5 in Additional file 1) and 189,838 SVs (Table S6 in Additional file 1) were also detected. Of the identified indels, 807,589 (92.8%) caused frame shifts, and 259,126 (29.8% of the total) were in gene regions. Only 19,888 (2.3%) of the indels were in coding regions, and 4,175 of these indels caused frame shifts, affecting 1,562 genes. Moreover, among the identified 189,838 SVs, 165,840 (87.4%) were deletions, 16,990 (8.9%) were insertions, and 6,706 (approximately 3.5%) were duplications.

**Figure 1:**
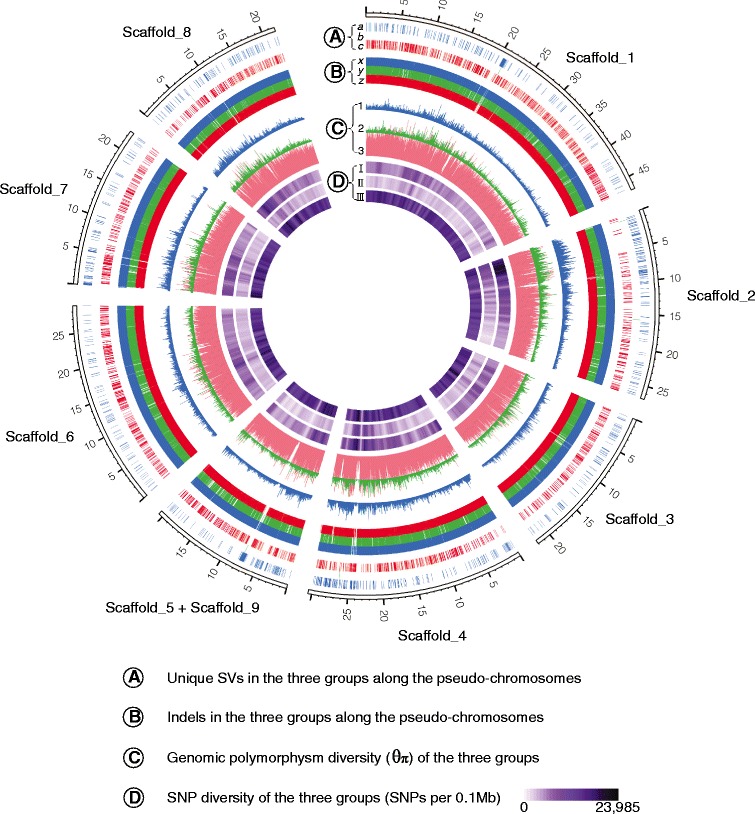
**Variation across the pseudo-chromosomes by Circos. (A)** Unique structure variations (SVs) in three groups, edible peach (a), ornamental peach (b), and wild peach (c). **(B)** Insertions and deletions (indels) in the three groups, edible peach (x), ornamental peach (y), and wild peach (z). **(C)** The genomic polymorphism diversity (*θw*) of the three groups, edible peach (1), ornamental peach (2), and wild peach (3). **(D)** SNP diversity of the three groups, edible peach (I), ornamental peach (II), and wild peach (III).

To shed light on the variation pattern across the genome, we examined the distribution of variations across the genome (Figure 1). There were 20,229 SNPs per megabase, 3,845 indels per megabase, and 836 SVs per megabase at the genome level. In some genomic regions (for example, putative centromere regions), the level of variation was substantially lower.

### Polymorphisms in the wild, ornamental, and edible groups

The number of SNPs in the wild (3,381,514), ornamental (1,065,215) and edible (2,098,002) groups account for 74.04%, 23.32%, and 45.94%, respectively, of all SNPs in the whole population (Figure S11 in Additional file 2). Few unique SNPs in ornamental peach and a large number of common SNPs between ornamental and edible peach indicate that they are closely related. The emergence of those unique SNPs may be the reason that domesticated peach shows various phenotypes. During domestication, the SNPs in CDS versus the total number of SNPs in the whole genome has remained constant in the wild (10.30%), ornamental (10.08%), and edible (9.73%) varieties. Only SNPs in introns have decreased in the edible (12.53%) and ornamental (12.70%) peach varieties compared with wild peach (16.28%). This preservation of SNPs would be advantageous because it would retain the various key genes needed for routine life activities.

Using the SNPs/genotype data, we calculated the polymorphism *θw* values [15] for all genotypes and determined that they were 4.462 × 10^-3^ for CDS regions, 3.610 × 10^-3^ for intronic regions, and 2.624 × 10^-3^ for whole genomes (Table S7 in Additional file 1). When we checked the *θw* values along the pseudo-chromosome within the three groups (Figure 1), we found that the ornamental and edible groups had fewer polymorphisms than the wild group. The decrease was found mainly in intergenic regions (Table S7 in Additional file 1). When we divided the edible group into an ‘edible landrace’ group and an ‘edible breeding’ group and calculated the genomic *θw* for each group, we found that *θw* in the edible breeding group was slightly decreased (Table S7 in Additional file 1; 1.851 × 10^-3^ for the ‘edible landrace’ group, 1.575 × 10^-3^ for the ‘edible breeding’ group). Two bottlenecks have been reported in the peach reference genome [11]. In our study, the difference in the number of *θw* polymorphisms shows that the bottleneck only occurred between wild species and edible peach. It was not apparent that the bottleneck occurred between landraces and all the modern breeding lines, including many Chinese varieties and a few eastern or western improved varieties. Bottlenecks reveal a part of domestication history, but the evolution of the population as a whole shows a broader perspective.

### Phylogenetic tree reveals that domesticated peach is a linear evolution

**Figure 2:**
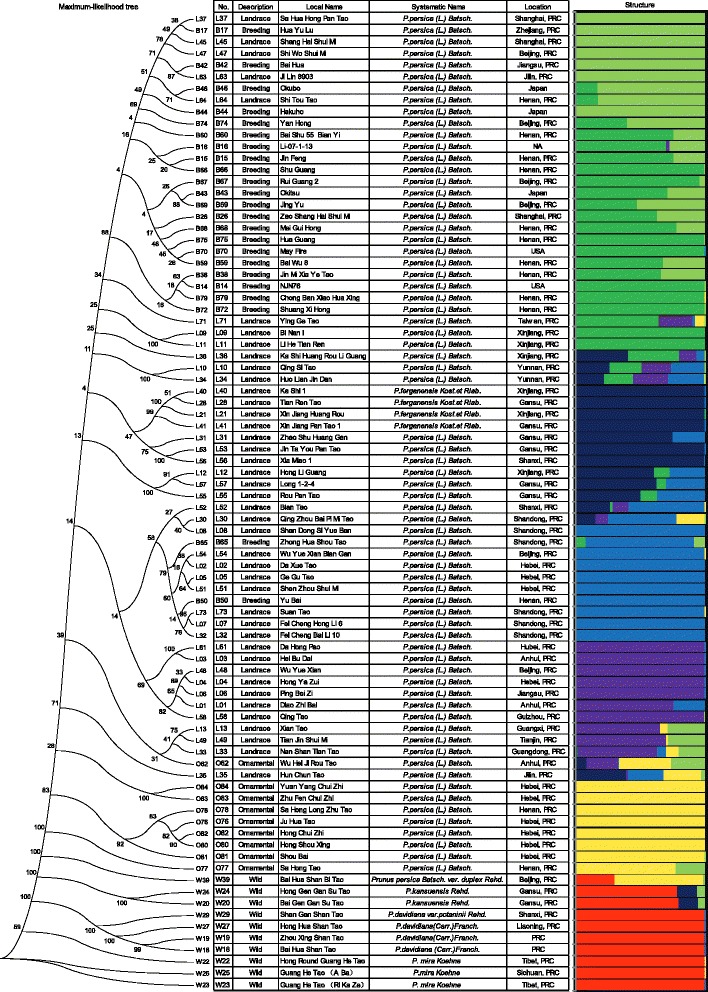
**The domestication history of peach.** Left: the phylogenetic tree of the 84 peach accessions. Right: evolution of the peach population structure (K = 7) using FRAPPE.

Using phenotypes and horticultural traits, the 84 peach accessions can be divided into several groups, especially along ornamental versus edible lines. Although peach has been divided into several categories, as reflected by their scientific names (Figure 2; Table S1 in Additional file 1), the population structure and domestication history of peach was still unclear.

Using the SNPs/genotypes of the 84 peach accessions, we analyzed the population structure and the domestication history of peach. We first constructed a maximum-likelihood (Figure 2) and neighbor-joining (Figure S12 in Additional file 2) phylogenetic tree based on the genetic distances calculated from the genotypes at all the SNP positions of the 84 peach varieties. The clear genetic separation between the wild and cultivated groups (including the edible and ornamental groups) confirmed the existence of a domestication event. Within the phylogenetic tree, nine ornamental accessions formed a unique ornamental group. The rest of the cultivated accessions were all fruit-bearing and were termed the edible group. Within the edible group, the landrace accessions and improved varieties (breeding accessions) can be further divided into subgroups. Within the improved varieties subgroup, some improved varieties from Japan (‘Okubo,’ ‘Hakuho,’ and ‘Okitsu’), the United States (‘NJN76’ and ‘May Fire’), and China (‘BaiHua’ and ‘ShuGuang’) were clustered with an old variety, ‘Chinese Cling’ (accession number L45, also called ‘ShangHaiShuiMi’).

Principal component analysis (PCA) supported the population structure revealed by the phylogenetic tree (Figure S13 in Additional file 2). Within the PCA, edible and ornamental peach formed a tight cultivated cluster that was distant from the wild accessions (Figure S13a in Additional file 2). This structure indicated that the edible and ornamental peach might originate from a single domesticated ancestor. When we magnified the figure of the cultivated cluster, the ornamental group appeared slightly separated from the edible group (Figure S13b in Additional file 2). It appears that the two groups were formed when part of an already domesticated population was recruited to a new purpose.

### Population structure shows different selection directions shaped ornamental peach and the edible landrace of peach

To further analyze the domestication history of peach, we constructed a multi-level (K = 2, 3…7) population structure to estimate the maximum likelihood ancestry and the proportion of the ancestral property in each individual (Figure S14 in Additional file 2). The method has been used in rice [9]. As each variety had already been classified into one of three groups (wild, ornamental, and edible), we could infer the ancestral group within the multi-level population structure: red represents wild; yellow represents ornamental; and purple, blue, dark blue, green, and light green represent different branches of edible peach. From K = 2 to K = 7 can reflect the domestication history of peach as a time lapse. The wild (red) parts existed stably during the time that K increased from 2 to 7, while the edible (blue) group subjected to artificial selection evolved into several subgroups. The ornamental (yellow) group emerged at K = 5, indicating that this group was derived from the ancient cultivated group (blue, K = 2, 3, 4).

In conclusion, peach clearly underwent a domestication event that separated the cultivated group from the wild species. Evidence for this event includes the whole branch of the cultivated groups (including ornamental and edible peach) in the phylogenetic tree (Figure 2; Figure S12 in Additional file 2), the first separation (K = 2) in the evolution of the population structure (Figure S14 in Additional file 2), and the emergence of almost the same points (blue/yellow) in the PCA analysis (Figure S13a in Additional file 2). Selection for different purposes may have resulted in the separation between the ornamental and edible groups, as supported by the second separation (K = 5) in the evolution of the population structure (Figure S14 in Additional file 2). Most varieties of ornamental peach originated directly from *P. persica* or *P. davidiana* [16,17]. Our work provides more evidence (Figure 2; Figure S14 in Additional file 2) that ornamental peach (yellow) originated from the ancient cultivated peach (blue).

Some archaeological records include peach remains [18]. In the Hemudu Site ruins in China (5000 to 3000 BC), archaeologists discovered some peach kernels. The finding indicated that peach was eaten by humans about 7,000 years ago. The kernels had the same appearance as those of the wild species. After persistent domestication took place, cultivated peach was first recorded in a book of songs in China 4,000 years ago. The earliest ornamental peach appeared in a later period during the Han dynasty (about 2,000 years ago). This archaeological evidence suggests that one domestication event and one separation event of peach occurred in China 4,000 to 7,000 years ago and 2,000 years ago, respectively.

### The evolution status of *P. kansuensis* and *P. ferganensis*

*P. mira* is considered the oldest progenitor of peach, but which species is the direct progenitor of peach was unclear. We also examined the evolution status of *P. kansuensis* and *P. ferganensis*. For a long time, northwest China, where *P. kansuensis* is native, was thought to be the origin center of the modern peach. A comparison of all the wild species except *P. ferganensis* with *P. persica* (cultivated peach), reveals that *P. kansuensis* is the most similar to *P. persica* in fruit traits, the presence of bud hair, tree character, and leaf and flower morphology [19]. However, some scientists have suggested that pollen morphology shows *P. davidiana* is closely related to *P. persica* [20]. In the present study, the phylogenetic tree shows that *P. kansuensis* was closer to *P. persica* than *P. davidiana*. This analysis holds that *P. davidiana* is more primitive than *P. kansuensis*.

*P. ferganensis* is closely related to cultivated peach but is distinguished by very long unbranched leaf veins and longitudinal grooves on the pit [21]. *P. ferganensis* was classified as a species [22] or a subspecies of peach on the basis of isozyme analyses [23]. As seen in the phylogenetic tree, the landraces deriving from different geographical populations from southern to northern China (such as accession numbers L33 and L48) are more primitive than *P. ferganensis*. Finally, some accessions, such as ‘ZaoShuHuangGan’ (accession number L31) and ‘JinTaYouPanTao’ (accession number L53), belonging to *P. persica*, are geographically closest to *P. ferganensis*, and are grouped with *P. ferganensis* in the phylogenetic tree. Therefore, *P. ferganensis* is indistinguishable from the cultivated varieties of peach and can only be separated on geographical terms.

### The regions under artificial selection in two cultivated groups

The divergence between the edible and ornamental groups was obviously caused by two different kinds of artificial selection. One form of selection was for better flavor or bigger fruit (edible selection), and the other was for more beautiful flowers or tree style (ornamental selection). We report a new method, based on population structure and Tajima’s D, to identify the two kinds of genes affected by these different kinds of artificial selection. First, we combined the results from the neighbor-joining tree and population structure to divide the cultivated group (ornamental and edible varieties) into six subgroups (Figure S15 in Additional file 2). Secondly, we picked the most representative accessions in each subgroup and took the Tajima’s D (Materials and methods); the region where the value of Tajima’s D was outside the neutral mutation range was treated as the candidate region under selection in this subgroup (Table 2). Third, we set the rule to identify the region under edible selection as follows: the region must be the candidate region in the edible C, D, E, and F subgroups but not in the ornamental A subgroup. We set the rule to identify the region under ornamental selection as follows: the region must be the candidate region in the ornamental A subgroup but not in all the edible C, D, E, and F subgroups (Figure S15 in Additional file 2). We did not consider the edible B subgroup because it was an intermediate subgroup, and the ratios of the common regions under selection between B and the other subgroups were nearly always 50% (Table S8 in Additional file 1), much higher than the ratios among any other pair of subgroups. By applying the rules, we identified 147 genes under edible selection and 262 genes under ornamental selection (Table S9a,b in Additional file 1).

**Table 2:** **Candidate regions under selection in each subgroup by Tajima’s D test**

**Subgroups**	**n**	**Neutral mutation range (Tajima’s D)**	**Selective judgment (Tajima’s D)**	**Amount of the candidate region under selection**
Ornamental (A)	8	[-1.663, 1.975]	(-∞,-1.663)∪(1.975,∞)	2,042
Edible_purple (B)	6	[-1.478, 1.999]	(-∞,-1.478)∪(1.999,∞)	7,906
Edible_blue (C)	8	[-1.663, 1.975]	(-∞,-1.663)∪(1.975,∞)	5,440
Edible_dblue (D)	7	[-1.608, 1.932]	(-∞,-1.608)∪(1.932,∞)	5,799
Edible_green (E)	9	[-1.713, 1.954]	(-∞,-1.713)∪(1.954,∞)	6,444
Edible_lgreen (F)	9	[-1.713, 1.954]	(-∞,-1.713)∪(1.954,∞)	3,628

Using the *ROD* and *Fst* measure (see Materials and methods), two methods to screen the genes under artificial selection, we determined that our results are supported by this method. In the regions that we identified as being under edible selection, the values for the edible subgroups were distinctly higher than those for the ornamental subgroup (Figure S16a,b in Additional file 2), whereas in the regions we identified as being under ornamental selection, the values for the ornamental subgroup were slightly elevated over the others (Figure S16c,d in Additional file 2). These regions were barely identifiable using only the *ROD*/*Fst* measure in a single subgroup/group, but they could be identified by comparing different subgroups. Only in this way could we locate the special regions that have been affected by two different kinds of artificial selection.

The *R* (resistance) genes and genes under selection are displayed along with the chromosomes in Figure S17a,b in Additional file 2. As shown in the figures, *R* genes are not randomly positioned but are gathered within clusters in the genome. Genes under selection are strongly separated from *R* genes across the chromosomes. These findings align with our expectation that fruit improvement practices might have focused only on the edible characteristics of the fruit rather than on biological resistance during domestication history. Our results for genes under selection are credible.

The results identifying genes that were under two different kinds of artificial selections are meaningful. Among the genes under ornamental selection, genes related to flavonoid biosynthesis, flower development, cell division, and carbohydrate metabolism are enriched (Table S9c in Additional file 1). For example, the ppa021198m and ppa001723m genes encode a transcription factor whose function is to be a positive regulator of flower development and signal transduction, and in particular to regulate the vegetative to reproductive phase transition of the meristem. The ppa001723m gene is expressed in roots, leaves, stems, and flowers, achieving its highest expression in flower stems and meristematic regions. These genes are essential for flower differentiation and development. Five genes (ppa002394m, ppa003246m, ppa013561m, ppa013547m, and ppa025412m) of the auxin response factor gene family were identified. This family of genes plays important roles in flowering promotion, stamen development, and floral organ abscission. These genes were also reported to be enriched among maize [24] and rice [9,25] domestication genes, suggesting that they play important and general roles in crop domestication and improvement.

Within the edible group of peach varieties, there was an enrichment of gene families related to the carbohydrate metabolic process, tricarboxylic acid cycle, and photosynthesis under domestication (Table S9c in Additional file 1). Some key genes are related to photosynthesis. The gene ppa010039m encodes a homologous protein of chlorophyll a-b binding protein of the garden pea (*Pisum sativum* L.), which may function in the light-harvesting complex as a light receptor to promote photosynthesis. The genes ppa004343m and ppa011951m encode homologous proteins of cytochrome P450 and thioredoxin in *Arabidopsis thaliana*, both of which function in the electron transport chain. These gene families may function to supply more photosynthesis product in cultivated peach than in wild species.

Another important gene family encodes enzymes that participate in carbohydrate metabolic processes to improve fruit aroma (ppa005746m, ppa005320m, ppa011098m, ppa017599m, ppa024343m, ppa002949m, and ppa010766m) and sweetness (ppa010073m, ppa009027m, ppa005780m, ppa025007m, and ppa000345m) [26]. Sorbitol is a special transport substance of photosynthesis product in the Rosaceae family. We identified a gene, ppa009027m, that encodes a homologous protein to D-sorbitol-6-phosphate dehydrogenase (S6PDH) in the region under selection of the edible group. The gene was also shown to be important in other Rosaceae species. After the cultivated pear genome was sequenced [27], it was apparent that the S6PDH gene family is bigger in pear, apple, and strawberry than in non-Rosaceae species. Transcriptome data in pear also indicated that all four S6PDH family genes are expressed in fruit, especially during later stages of fruit development, indicating that this gene is essential during domestication. It functions in the transportation of sugar to improve flavor in all Rosaceae species.

Part of the domestication process in most crop species is an increase in fruit size relative to the fruit of the progenitor species, sometimes referred to as the 'domestication syndrome' [28]. In *A. thaliana*, small changes in the expression levels of the gene encoding E3 ubiquitin protein ligase substantially alter organ size, most likely by marking cellular proteins for degradation [29]. The gene encoding this protein in rice alters the number of cells in the spikelet hull and contributes to rice grain width and weight [30]. Finally, in a previous study [11], the linkage disequilibrium (LD) peaks that may result from selective sweeps related to peach domestication on scaffold 4 at approximately 2 Mb, approximately 8 Mb, and approximately 20 Mb and on scaffold 5 at approximately 8 Mb, 12 to 13 Mb, and 15 to 17 Mb, were mapped to quantitative trait loci for fruit size [31]. In our study, two genes encoding E3 ubiquitin protein ligase (ppa009446m and ppa000974m) are of particular importance, because they were also found in scaffold 4 at approximately 2 Mb and in scaffold 5 at 8 Mb. Other important genes in edible peach domestication are ppa019174m, ppa025007m, and ppa023784; they encode an expansin-A9 protein, polygalacturonase, and pectinesterase, respectively, and play a role in cell expansion.

We analyzed the Gene Ontology enrichment of the two kinds of genes that were subject to different kinds of selection. The genes in the ornamental selection group are enriched in four functional groups (Figure S18 in Additional file 2): (a) CTP, GTP, and UTP biosynthetic process; (b) lysine, gluconeogenesis, DNA metabolic process, and tRNA aminoacylation; (c) recognition of pollen, response to stimulus, drug transport, and vesicle exocytosis; and (d) regulation. The first and second groups include basic metabolic reactions that occur in plants and animals, whereas the third and fourth groups code for proteins involved in advanced metabolic reactions. Together, they show a functional perspective of all the genes under ornamental selection in peach.

By contrast, the enriched genes in the edible selection group are mostly involved in carbohydrate transport, transposition, protein catabolic process, and regulation of DNA-dependent transcription (Figure S19 in Additional file 2). This difference indicates that the artificial selection pressure on the ornamental and edible groups was divided functionally at the gene level and also suggests that the genes we had identified as being under selection did belong to the assigned groups. The identification of these genes provides opportunities for quick study and improvement of some traits.

### Whole-genome patterns of linkage disequilibrium

LD levels may vary across genomes due to differences in recombination rates, selective pressures, mating systems (selfing versus out-crossing), and effective population sizes. Several reports have suggested that narrow-based germplasm groups have longer LD blocks than broad-based germplasm groups [32,33]. Meanwhile, among vegetatively propagated domesticated trees, the absence of recombination can generate an extended LD block compared with that of undomesticated trees.

We analyzed the LD of different peach groups by calculating *r*^*2*^ between SNPs and the decay of *r*^*2*^ with increasing distance between SNPs (Figure S20a in Additional file 2). The LD level was higher for domesticated (including edible and ornamental) peach than for the wild group. The LD decay in the ornamental group was slower than that in either the wild or the edible group, with half of maximum *r*^*2*^ at about 56 kb, 5 kb, and 14 kb in three groups, respectively. The values show that peach has a medium LD level compared with other self-compatible plants, such as *A. thaliana* (approximately 3 to 4 kb) [34], cultivated soybean (approximately 150 kb) [6], wild soybean (approximately 75 kb) [6], wild rice (approximately 10 kb), [9] and cultivated rice (65 to 200 kb) [9]. These results show that association mapping on the basis of cultivated accessions is feasible.

Since we know the derivations of the five cultivated subgroups (A, C, D, E, F above; Figure S15 in Additional file 2), we assigned names to them as follows: Edible_blue is 'edible landrace 1'; Edible_dblue is 'edible landrace 2'; Edible_green is 'edible breeding 1'; Edible_lgreen is 'edible breeding 2'; and Edible_purple is 'intermediate'. We noted similar LD decay in edible landrace 1 (22 kb), edible breeding 1 (20 kb), edible breeding 2 (20 kb), and intermediate (18 kb). The LD decay in edible landrace 2 (50 kb) was longer than the others, perhaps due to a consistent cultivation environment that resulted in a narrow genetic background (all the members of this subgroup belong to *P. ferganensis* and originated in northwest China) and the founder effect (Figure S20b in Additional file 2). The LD decay in the improved varieties, edible breeding 1 and 2 (19 Kb) was relatively fast compared with that of edible landrace 1 and 2 (28 kb) (Figure S20 in Additional file 2c).

### The sharp decline of heterozygous SNP ratios in peach during domestication

Typically, regions under artificial selection possess a long LD fragment, a phenomenon that is apparent in silkworm (*Bombyx*) [35], rice [9], maize [8], and grape [36]. We identified the LD patterns in two regions under edible selection on scaffold 4 and scaffold 5 (Figure S21a-h in Additional file 2). The LD block was unusually long in the first region of the two edible subgroups (Figure S21c,d in Additional file 2), but an even longer LD block was found in the ornamental group that was not under selection (Figure S21b in Additional file 2). These results show that a long LD block is not always consistent with the region under selection. In another region under edible selection, there were only two SNPs. The vanished heterozygote SNPs (Figure S21g,h in Additional file 2) in the region may be another important reason for it being selected.

Next, we estimated the ratio of the heterozygous SNPs in whole genomes of all the varieties (Table S10 in Additional file 1). The average value for peach (1.552%) was lower than that for other self-compatible crops, including soybean (3.129%) [6], rice (6.495%) [9] and cultivated grapevine (7%) [37], but it was similar to that of a self-incompatible species, pear (1.0%) [27]. Most importantly, the ratios of the heterozygous SNPs in the cultivated group were lower than those in the wild group (1.332% versus 3.770%, respectively). The lowest ratio of heterozygous SNPs appeared in an old landrace accession (accession number L11) and was only 0.153%, less than one-fiftieth of that of a wild accession (accession number W29). These results indicate that there was a sharp decline in the heterozygous SNP ratio in peach during domestication. This reduction in heterozygosity most likely occurred due to inbreeding or to the bottleneck experienced by domesticated lines.

The multiple of the ratio of the heterozygous SNPs in wild versus cultivated peach is 3.012, higher than that of two self-compatible species, soybean (1.552) [6] and rice (1.200) [9], and higher than that of two self-incompatible species, apple [38] and cherry [39] (Table S11 in Additional file 1). The decreased number of heterozygous SNPs in the cultivated group was a result of the change in the number of SNPs and the polymorphisms in intergenic regions during the domestication of the peach.

In order to understand which key factors caused this change, we list the factors that might affect heterozygosity the most, such as self-compatibility or self-incompatibility, grafting or seedling propagation, long or short lifespan, and large or small population size, for different plants in Table S12 in Additional file 1. Among these, mating system and mode of reproduction mainly affected heterozygosity during free recombination in independent assortment, whereas lifespan and population size mainly affected the mutation volume in the population as a whole. Self-compatibility, grafting propagation, short lifespan, and small population size tend to increase the ratio of homozygous genes in a plant, and the other factors lead to the opposite result.

We found no obvious relationship between the final level of heterozygosity in the cultivated group and the effect of mutation factors. Genetic recombination had a greater influence than mutation on the heterozygosity of these plants over the course of their evolution. This finding is supported by many other studies [40]. Of the two factors related to free recombination, the effect of mating system on heterozygosity is much stronger than the effect of reproduction mode, because the change of heterozygosity was coincident with the mating system but not the propagation mode in apple, cherry, rice, and soybean. We reached the following conclusions. First, cultivated apple and cherry were self-incompatible and mostly propagated by grafting, so it is possible for the heterozygosity in some cultivated groups to be even higher than that of a wild group. Second, rice and soybean are self-compatible plants and propagated only by seedling breeding, so the heterozygosity in these cultivated groups is slightly lower than that of the wild group. Third, peach is not only a self-compatible plant but also widely propagated by grafting, so the heterozygosity in some cultivated peach varieties was much lower than that of wild varieties (Table 3).

**Table 3:** **Analysis of the heterozygote ratio and factors that influenced the domestication history of five plants**

**Plants**	**Free recombination effects in independent assortment**				**Mutation effects**				**Result of human actions and natural effects on heterozygosity in cultivated group**
	**Mating system**		**Mode of reproduction**		**Lifespan**		**Population size**		
	**Self-compatibility**	**Self-incompatibility**	**Grafting**	**Seedling**	**Short**	**Long**	**Small**	**Large**	
Apple, cherry		**↑↑**	**↓**			↑	↑		**↑**
Rice, soybean	**↓↓**			**↑**	↑↑			↑↑	**↓**
Peach	**↓↓**		**↓**			↑	↑		**↓↓↓**

Up arrows represent 'increases', down arrows represent 'decreases' and the thickness of the arrows indicates the intensity of different factors that affect the heterozygote ratio in five plants.

### The benefits of the low ratio of heterozygous SNPs against total SNPs in peach

Linkage analysis was long considered a basic method to exploit the genes associated with important traits, such as sugar content, disease resistance [41], chilling requirement, heat requirement, and bloom date [42]. Genome-wide association studies, an emerging popular method to screen quantitative trait loci using a resequencing strategy, have been successfully used in different plants [43–45]. Association mapping is particularly well suited to screening of perennial horticultural crops because it can overcome their characteristic pedigree-based mapping limitations.

In most fruit crops, genetic analysis is complicated due to the presence of high-level heterozygosity [46]. To determine what benefits the low heterozygous SNP ratios in peach found in this study could offer, we performed association studies to determine whether this can be successfully carried out using just the 84 accessions chosen. Flesh adhesion traits [47], which are controlled by two concatenated copies of the *PG* gene, encoding endopolygalacturonase, were our test target. The genes were located in the distal region of scaffold 4 between nucleotides 22,649,519 and 22,687,159 according to a recent analysis [48]. We conducted the association analysis using 100,000 SNPs around scaffold 4 (22.5 Mb) to identify the association signals. We applied both the mixed linear model (MLM) and the general linear model (GLM), using TASSEL 3.0 software [49] (Figure S22 in Additional file 2). The MLM approach, which took genome-wide patterns of genetic relatedness and population structure into account, showed decreasing association signals (*P* < 10^-3^) compared with the GLM approach (*P* < 10^-7^). The result also indicates that peach has a simple origin. Finally, the peak association signals (nucleotide 22,687,059) for flesh adhesion appeared near (but not within) the *PG* genes that were identified previously. The success of the analysis indicates that association studies within the 84 peach accessions are indeed feasible, perhaps due to the low heterozygote ratio.

## Conclusions

The resequencing data reported here provide substantial resources for marker-assisted breeding in peach and for population genetics analysis in woody plants. As there is no sexual barrier between wild and cultivated peach, the available diversity in the wild germplasm could be an important tool to expand the allelic pool of cultivated peach through introgression, particularly to enhance the resistance of peach to adverse conditions and insect pests.

Our work identified a set of domestication genes, including the gene encoding the protein that regulates the vegetative to reproductive phase transition of the meristem in ornamental peach, and the gene encoding S6PDH, E3 ubiquitin ligase in edible peach, that may be of agronomic importance. This dataset will facilitate the identification of important domestication genes in the future and provide information that can be used in marker-assisted breeding in peach and other fruit crops. Functional verification of these candidate genes may enable a comprehensive understanding of the differences in biological processes between wild and cultivated peach.

Peach has long been thought to have a lower level of genetic variability [4] as a consequence of its self-compatible mating system [50], in contrast to the gametophytic self-incompatibility of most species of the *Prunus* genus [51]. Our finding of the small number of SNPs and low *θw* values in cultivated groups confirmed this. The changes mainly occurred in intergenic regions, and a number of unique SNPs were found in edible and ornamental peach varieties. These SNPs may be associated with the emergence of new phenotypes in peach. New SNPs may originate in mutations and be fixed through the self-compatible mating system in combination with the influence of human actions, such as positive artificial selection and vegetative propagation.

## Materials and methods

### Sample collection

All 84 *Prunus* accessions were selected from the peach core collections, which represent most ecotypes in the world, preserved in the National Fruit Tree Germplasm Repository, Zhengzhou Fruit Research Institute, Chinese Academy of Agricultural Sciences, China. In order to enlarge the range of diversity and the representativeness of the 84 samples (10% of the germplasm repository), we applied the following key rules to our selection of samples. First, each sample had no direct family relationships with other samples. Each of the samples that we picked had an independent local name. Second, the wild group contained all four main wild species related to peach and the sample size was at least 10. *P. davidiana*, *P. ferganensis*, *P. kansuensis*, and *P. mira* have been identified as the main wild species that are related to peach [52]. Therefore, we chose three samples of *P. davidiana*, four samples of *P. ferganensis*, two samples of *P. kansuensis*, three samples of *P. mira*, and two samples of the wild varieties *P. davidiana* var. *potaninii* Rehd. (‘W29’) and *P. persica* Batsch var. *duplex* Rehd. (‘W39’). ‘W39’ is not a true member of *P. persica* but a hybrid/cross between *P. persica* and *P. davidiana* that has a lot of phenotypic traits in common with both wild and ornamental peach. Third, the ornamental group contained samples with representative ornamental phenotypic traits. We chose samples whose flower colors were red, pink, white, or mosaic (multicolor); whose petals were double or single; whose broomy growth habit (tree growth habit) contained weeping, standard, open and columnar types; and whose tree size was dwarf or regular. Our final selection included nine samples with different ornamental phenotypic traits. Fourth, the landrace accessions and breeding lines contained as much of the diversity of edible peach as possible. Because peach is native to China and was first domesticated and cultivated in the region between the Tarim Basin and the north slopes of the Kunlun Shan Mountains [53], China contains a wide variety of types of peach, many of which were the parents or grandparents of current peach breeding lines worldwide. When we selected landraces and breeding lines, we included all the typical peach varieties in six important geographical groups: northwest China, northeast China, the YunGui plateau, the middle and lower reaches of the Yangtze River, a wide range of northern China, and southern China [54]. Our final selection included 42 landrace samples and 23 breeding lines. They have a wide range of climatic and geographic regions, from north of 22.5 latitude to 42.5 latitude.

### DNA sequencing and mapping

We prepared DNA to construct the libraries from the fresh leaves of the 84 peach varieties, using the CTAB method [55]. The insert-size of the libraries was 500 bp and the pair-end reads were 49 bp. All the libraries were sequenced by the high throughput instrument Illumina GA2.

We used the published genome ‘Lovell’ as reference [10]. The reference genome was assembled well and the large scaffolds represented eight chromosomes of peach. We mapped all the reads of each accession to the scaffolds of the reference genome through SOAP2 [56] with the following parameters: -m 100 -x 888 -s 35 -l 32 -v 3 -p 4. After the mapping result was obtained, they were sorted by the scaffold coordinates. We used the mapping result to detect variations.

### Detection of variations

#### SNPs

The SNPs/genotypes of the population were identified by following three steps. First, based on the mapping result of each accession, we used SOAPsnp [57] to identify the SNPs of individuals with the parameters '-L 50 -u -F 1'. Second, with the SNPs of each individual, we used GLFmulti to obtain the raw SNP/genotype files in the population. Third, we filtered the raw files to obtain the final SNPs/genotypes of the population that met certain criteria, which included the existence of two alleles, sufficient sequencing depth, and a suitable average mapping rate. We randomly validated 18 selected homozygous SNPs in 48 accessions. We also validated the heterozygous SNPs in 10 accessions that were significantly clustered in some regions by randomly picking 12 heterozygous SNPs by Sanger sequencing. These validations confirmed the high quality of the SNP data set. We also randomly selected 43 SNPs to verify the accuracy using 81 accessions through Sequenom MassArray platform. The results showed that the accuracy of the homologous SNPs was about 92.2%, and that of heterozygous SNPs 76.3%. To acquire more comprehensive statistical data about SNP calling sensitivity, we cloned two genes using Sanger technology in 72 and 45 accessions, respectively. One gene was ppa016711m (Chr3: 12840372..12842225), and the other was ppa010260m (Chr6: 25061436..25062620). After aligning them with the reference, 32 and 17 SNPs were identified in these two regions; using Illumina GA II technology, these were 24 and 11, respectively. Therefore, the sensitivity of the variant calling should be 71.4% ((11 + 24)/(32 + 17)).

#### Indels

In order to identify small insertions and deletions, we mapped all the reads of each accession to the reference using SOAP2 with parameters -m 100 -x 888 -s 35 -l 32 -v 3 -p 4 -g 5 (the added parameter -g 5 indicates that a gap within 5 bp was allowed). Then we used the SOAPindel pipeline [58] to detect the indels (1 to 5 bp) of each accession. We combined all the indels together to obtain the union set of all the indels in the population.

#### Structure variations

SOAPsv was used to identify SVs. The input files included the mapping result of each accession, the gap information of the reference genome, and the insert-size of the mapped paired-end reads. According to the mapping result, a remarkable difference between the gap information and the insert-size of paired-end reads usually indicated candidate SVs. To improve the accuracy of our SVs detected by SOAPsv, we used another SV discovery software program, ‘DELLY’, to re-check the results. First, we used BWA [59] (other than SOAP) to align the reads to the reference genome. Second, we used the DELLY packages [60] to identify deletion, duplication, inversion, and translocation. Third, we compared the new SVs with the old SVs in each accession, and found that there was a greater than 50% overlap between the new SVs and old SVs. These regions are more reliable SVs because they can be identified by two different alignment algorithms.

### Population analysis

#### Phylogenetic tree

The software PHYLIP was used to calculate the clustering tree based on the population genotypes at all the SNP positions. The algorithm we chose used the maximum-likelihood method. We set accession ‘W23’, belonging to *P. mira*, a primitive species of *P. persica*, as the out-group.

#### Principal component analysis

In order to perform PCA within the peach population, we first transformed the population genotypes into a matrix that included only the numbers 0, 1, and 2: 0 indicated that the genotype was homozygous for the reference genotype; 1 meant that it was heterozygous for the reference genotype; and 2 meant that it was homozygous for the non-reference genotype. We calculated the sample covariance of the matrix that contained all individuals’ information (with the numbers 0, 1, and 2). Finally, we calculated the eigenvector decomposition of the matrix using the R function eigen and plotted the PCA (Figure S12 in Additional file 2).

#### Population structure

The program FRAPPE [61] was used to perform population structure analysis. It was based on the maximum-likelihood method. Before using FRAPPE, we used PLINK [62] to generate the needed map files. The input parameter *K* was changed from 2 to 7, representing the assumed groups of the simulated population in ancient times.

### The candidate region under selection

Because artificial selection would create the genomic regions that the Tajima’s D test [63] showed to be 'non-neutral', we used the test in each subgroup and determined which candidate regions were under selection in each subgroup. Specifically, we calculated the Tajima’s D value in a sliding 10 kb window along the genome and compared the value with the confidence limit of D (neutral mutation range), which was related to the sample size *n*. The intensity was calculated by the distance deviated from the middle value of the confidence limit of D. If the intensity in the region was more than 100%, the region was considered to be the candidate region under selection in the subgroup.

The *ROD* and *Fst* measures were also used to screen the candidate region under selection. *ROD* reflects the reduction of diversity between the cultivars and the wild species. We defined it as:

$$ ROD=1-\frac{\pi_{cul}}{\pi_{wild}} $$

where *π*_*cul*_ and *π*_*wild*_ are the values of *π* for the cultivars and the wild species, respectively, calculated in 10 kb windows along the genome.

*F*_*ST*_ is a measure of population differentiation in genetic distance, based on genetic polymorphism data and defined as:

$$ {F}_{ST}=\frac{\pi_{\mathrm{Between}}-{\pi}_{\mathrm{Within}}}{\pi_{\mathrm{Between}}} $$

where *π*_Between_ and *π*_Within_ represent the average number of pairwise differences between two individuals sampled from different populations (*π*_Between_) or the same population (π_Within)_.

### Linkage disequilibrium analysis

Haploview software [64] was used to calculate the LD on the basis of the population SNPs/genotypes in each group or subgroup. We also extracted the genotypes of specific genomic regions of interest. The parameters that we used with Haploview were '-n -pedfile -info -log -maxdistance 1000 -minMAF 0.1 -hwcutoff 0.001 -dprime -memory 5120 -blockoutput GAB -pairwiseTagging -png -svg'.

### Genome-wide association studies

Association analyses were conducted using GLMs and MLMs with TASSEL v.2.1 [48,65]. A kinship matrix (K-matrix), the pair-wise relationship matrix calculated by TASSEL v.2.1, and the Q-matrix calculated by STRUCTURE as a correction for population structure were used in the MLM association models to calculate *P*-values to associate each SNP marker with the trait of interest, to avoid spurious associations by TASSEL v.2.1. Results were compared to determine the best model for our analysis.

### Data access

The sequencing data from the 84 accessions have been submitted to the Sequence Read Archive [66] under accession number SRA073649.

## Additional files

Additional file 1: Table S1. Peach samples and their origins. **Table S2.** Statistical traits and phenotypes of the 84 accessions. **Table S3.** Detailed traits and descriptions of each accession. **Table S4.** Mean depth and coverage in each accession. **Table S5.** Summary of indels (insertions and deletions). **Table S6.** Summary of structure variations (SVs). **Table S7.** Average number of polymorphisms in different groups. **Table S8.** Statistics for the common regions under selection between every two subgroups. **Table S9.** All 147 genes under edible selection and 262 genes under ornamental selection. **Table S9a.** Genes and their function annotations in the regions under ornamental selection. **Table S9b.** Genes and their function annotations in the regions under edible selection. **Table S9c.** Statistics for the density of the related genes in the whole genome and regions under selection. **Table S10.** Ratio of heterozygous SNPs in each group/subgroup of peach. **Table S11.** Ratio of the average heterozygous SNPs in the wild versus cultivated group in five plants. **Table S12.** Factors that influence heterozygosity in different plants. Additional file 2: Figure S1. Geographic distribution of the 84 peach accessions. **Figure S2.** Relationship between the identified SNPs and the sample sizes. **Figure S3.** Relationship between the called SNPs and the sequencing depth. **Figure S4.** Total depth of all genotype sites according to SNPs in the 84 samples. **Figure S5.** SNP depth of each sample (L09, L13, W18, and L34). **Figure S6.** Details of the mapping results in SNP sites. **Figure S7.** SNP depth distributions of samples without SNPs in repeat regions or homologous sequences. **Figure S8.** Depth of homozygous (n = 0) and heterozygous sites (n > 0) in the genotype. **Figure S9.** Two supposed models for explaining why the depth of heterozygous SNP sites was higher than the depth of homozygous SNP sites. **Figure S10.** Relationship between missed genotype ratio and sequencing depth in heterozygotes and homozygotes by data fitting. **Figure S11.** Venn diagram of the unique and common SNPs in three groups. **Figure S12.** The maximum-likelihood tree and the neighbor-joining tree of the 84 peach accessions. **Figure S13.** Principal component analysis of wild, ornamental, and edible peaches. **Figure S14.** Population structure of 84 peach accessions by FRAPPE. **Figure S15.** The selection judgment outline of the 'region under selection', based on population structure. **Figure S16.** *ROD* and *Fst* values in the regions under edible and ornamental selection. **Figure S17.** *R* (resistance) genes and the genes under selection in the chromosomes. **Figure S18.** Gene Ontology analysis of the genes under ornamental selection. **Figure S19.** Gene Ontology analysis of the genes under edible selection. **Figure S20.** Linkage disequilibrium decay rates in different groups and subgroups. **Figure S21.** Linkage disequilibrium analysis of two regions under selection. **Figure S22.** Genome-wide association studies of the flesh adhesion trait.

## Abbreviations

bp: base pair
CDS: coding sequence
GLM: general linear model
LD: linkage disequilibrium
MLM: mixed linear model
PCA: principal component analysis
SNP: single-nucleotide polymorphism
SV: structure variation
UTR: untranslated region
