# Supplementary material for: Comparative population genomics reveals the domestication history of the peach, Prunus persica, and human influences on perennial fruit crops
Source: Genome Biol. 2014 Jul 31;15(7):415. doi: 10.1186/s13059-014-0415-1 (PMC4174323; doi:10.1186/s13059-014-0415-1)
Supplement: Additional file 1: Table S1. — Peach samples and their origins. Table S2. Statistical traits and phenotypes of the 84 accessions. Table S3. Detailed traits and descriptions of each accession. Table S4. Mean depth and coverage in each accession. Table S5. Summary of indels (insertions and deletions). Table S6. Summary of structure variations (SVs). Table S7. Average number of polymorphisms in different groups. Table S8. Statistics for the common regions under selection between every two subgroups. Table S9. All 147 genes under edible selection and 262 genes under ornamental selection. Table S9a. Genes and their function annotations in the regions under ornamental selection. Table S9b. Genes and their function annotations in the regions under edible selection. Table S9c. Statistics for the density of the related genes in the whole genome and regions under selection. Table S10. Ratio of heterozygous SNPs in each group/subgroup of peach. Table S11. Ratio of the average heterozygous SNPs in the wild versus cultivated group in five plants. Table S12. Factors that influence heterozygosity in different plants. [file 13059_2014_415_MOESM1_ESM.docx]

#### Supplementary Table 1. Peach samples list with the origin.

| **Number** | **Sample ID** | **Name** | **Systematic Name** | **Origin** |
| --- | --- | --- | --- | --- |
| **L01** | 11034342 | Diao Zhi Bai | *P. persica* (L.) Batsch. | Anhui, PRC |
| **L02** | 11034344 | Da Xue Tao | *P. persica* (L.) Batsch. | Hebei, PRC |
| **L03** | 11034345 | Hei Bu Dai | *P. persica* (L.) Batsch. | Anhui, PRC |
| **L04** | 11034346 | Hong Ya Zui | *P. persica* (L.) Batsch. | Hebei, PRC |
| **L05** | 11034347 | Ge Gu Tao | *P. persica* (L.) Batsch. | Hebei, PRC |
| **L06** | 11034348 | Ping Bei Zi | *P. persica* (L.) Batsch. | Jiangsu, PRC |
| **L07** | 11034349 | Fei Cheng Hong Li 6 | *P. persica* (L.) Batsch. | Shandong, PRC |
| **L08** | 11034350 | Shan Dong Si Yue Ban | *P. persica* (L.) Batsch. | Shandong, PRC |
| **L09** | 11034351 | Bi Nan I | *P. persica* (L.) Batsch. | Xinjiang, PRC |
| **L10** | 11034352 | Qing Si Tao | *P. persica* (L.) Batsch. | Yunnan, PRC |
| **L11** | 11034353 | Li He Tian Ren | *P. persica* (L.) Batsch. | Xinjiang, PRC |
| **L12** | 11034354 | Hong Li Guang | *P. persica* (L.) Batsch. | Xinjiang, PRC |
| **L13** | 11034355 | Xian Tao | *P. persica* (L.) Batsch. | Guangxi, PRC |
| **B14** | 11034356 | NJN76 | *P. persica* (L.) Batsch. | USA |
| **B15** | 11034357 | Jin Feng | *P. persica* (L.) Batsch. | Henan, PRC |
| **B16** | 11034358 | Li-07-1-13 | *P. persica* (L.) Batsch. | NA |
| **B17** | 10105210 | Hua Yu Lu | *P. persica* (L.) Batsch. | Zhejiang, PRC |
| **W18** | 10113155 | Bai Hua Shan Tao | *P. davidiana*(Carr.)　Franch. | PRC |
| **W19** | 10113156 | Zhou Xing Shan Tao | *P. davidiana*(Carr.)　Franch. | PRC |
| **W20** | 10113157 | Bai Gen Gan Su Tao | *P. kansuensis* Rehd. | Gansu, PRC |
| **L21** | 10113158 | Xin Jiang Huang Rou | *P. ferganensis* Kost.et Riab. | Xinjiang, PRC |
| **W22** | 10113159 | Hong Round Guang He Tao | *P. mira* Koehne | Tibet, PRC |
| **W23** | 10113160 | Guang He Tao （Ri Ka Ze） | *P. mira* Koehne | Tibet, PRC |
| **W24** | 1008635 | Hong Gen Gan Su Tao | *P. kansuensis* Rehd. | Gansu, PRC |
| **W25** | 1008637 | Guang He Tao （A Ba） | *P. mira* Koehne | Sichuan, PRC |
| **B26** | 1008642 | Zao Shang Hai Shui Mi | *P. persica* (L.) Batsch. | Shanghai, PRC |
| **W27** | 10094868 | Hong Hua Shan Tao | *P. davidiana* (Carr.) Franch. | Liaoning, PRC |
| **L28** | 10094869 | Tian Ren Tao | *P. ferganensis* Kost.et Riab. | Gansu, PRC |
| **W29** | 10094870 | Shan Gan Shan Tao | *P. davidiana var.potaninii* Rehd. | Shanxi, PRC |
| **L30** | 10094871 | Qing Zhou Bai Pi Mi Tao | *P. persica* (L.) Batsch. | Shandong, PRC |
| **L31** | 10094872 | Zhao Shu Huang Gan | *P. persica* (L.) Batsch. | Gansu, PRC |
| **L32** | 10094873 | Fei Cheng Bai Li 10 | *P. persica* (L.) Batsch. | Shandong, PRC |
| **L33** | 10094874 | Nan Shan Tian Tao | *P. persica* (L.) Batsch. | Guangdong, PRC |
| **L34** | 10094875 | Huo Lian Jin Dan | *P. persica* (L.) Batsch. | Yunnan, PRC |
| **L35** | 10094876 | Hun Chun Tao | *P. persica* (L.) Batsch. | Jilin, PRC |
| **L36** | 10094877 | Ka Shi Huang Rou Li Guang | *P. persica* (L.) Batsch. | Xinjiang, PRC |
| **L37** | 10094878 | Sa Hua Hong Pan Tao | *P. persica* (L.) Batsch. | Shanghai, PRC |
| **B38** | 10094879 | Jin Mi Xia Ye Tao | *P. persica* (L.) Batsch. | Henan, PRC |
| **W39** | 10105146 | Bai Hua Shan Bi Tao | *P. persica* Batsch. *var. duplex* Rehd | Beijing, PRC |
| **L40** | 10105148 | Ka Shi 1 | *P. ferganensis* Kost.et Riab. | Xinjiang, PRC |
| **L41** | 10105149 | Xin Jiang Pan Tao 1 | *P. ferganensis* Kost.et Riab. | Gansu, PRC |
| **B42** | 10105151 | Bai Hua | *P. persica* (L.) Batsch. | Jiangsu, PRC |
| **B43** | 10105152 | Okitsu | *P. persica* (L.) Batsch. | Japan |
| **B44** | 10105153 | Hakuho | *P. persica* (L.) Batsch. | Japan |
| **L45** | 10105154 | Shang Hai Shui Mi | *P. persica* (L.) Batsch. | Shanghai, PRC |
| **B46** | 10105156 | Okubo | *P. persica* (L.) Batsch. | Japan |
| **L47** | 10105157 | Shi Wo Shui Mi | *P. persica* (L.) Batsch. | Beijing, PRC |
| **L48** | 10105158 | Wu Yue Xian | *P. persica* (L.) Batsch. | Beijing, PRC |
| **L49** | 10105159 | Tian Jin Shui Mi | *P. persica* (L.) Batsch. | Tianjin, PRC |
| **B50** | 10105161 | Yu Bai | *P. persica* (L.) Batsch. | Henan, PRC |
| **L51** | 10105162 | Shen Zhou Shui Mi | *P. persica* (L.) Batsch. | Hebei, PRC |
| **L52** | 10105163 | Bian Tao | *P. persica* (L.) Batsch. | Shanxi, PRC |
| **L53** | 10105164 | Jin Ta You Pan Tao | *P. persica* (L.) Batsch. | Gansu, PRC |
| **L54** | 10105165 | Wu Yue Xian Bian Gan | *P. persica* (L.) Batsch. | Beijing, PRC |
| **L55** | 10105166 | Rou Pan Tao | *P. persica* (L.) Batsch. | Gansu, PRC |
| **L56** | 10105167 | Xia Miao 1 | *P. persica* (L.) Batsch. | Shanxi, PRC |
| **L57** | 10105168 | Long 1-2-4 | *P. persica* (L.) Batsch. | Gansu, PRC |
| **L58** | 10105169 | Qing Tao | *P. persica* (L.) Batsch. | Guizhou, PRC |
| **B59** | 10105170 | Bai Wu 8 | *P. persica* (L.) Batsch. | Henan, PRC |
| **B60** | 10105171 | Bai Shu 55 Bian Yi | *P. persica* (L.) Batsch. | Henan, PRC |
| **L61** | 10105174 | Da Hong Pao | *P. persica* (L.) Batsch. | Hubei, PRC |
| **O62** | 10105175 | Wu Hei Ji Rou Tao | *P. persica* (L.) Batsch. | Anhui, PRC |
| **L63** | 10105176 | Ji Lin 8903 | *P. persica* (L.) Batsch. | Jilin, PRC |
| **L64** | 10105177 | Shi Tou Tao | *P. persica* (L.) Batsch. | Henan, PRC |
| **B65** | 10105178 | Zhong Hua Shou Tao | *P. persica* (L.) Batsch. | Shandong, PRC |
| **B66** | 10105183 | Shu Guang | *P. persica* (L.) Batsch. | Henan, PRC |
| **B67** | 10105185 | Rui Guang 2 | *P. persica* (L.) Batsch. | Beijing, PRC |
| **B68** | 10105186 | Mei Gui Hong | *P. persica* (L.) Batsch. | Henan, PRC |
| **B69** | 10105187 | Jing Yu | *P. persica* (L.) Batsch. | Beijing, PRC |
| **B70** | 10105188 | May Fire | *P. persica* (L.) Batsch. | USA |
| **L71** | 10105189 | Ying Ge Tao | *P. persica* (L.) Batsch. | Taiwan, PRC |
| **B72** | 10105191 | Shuang Xi Hong | *P. persica* (L.) Batsch. | Henan, PRC |
| **L73** | 10105193 | Suan Tao | *P. persica* (L.) Batsch. | Shandong, PRC |
| **B74** | 10105195 | Yan Hong | *P. persica* (L.) Batsch. | Beijing, PRC |
| **B75** | 10105196 | Hua Guang | *P. persica* (L.) Batsch. | Henan, PRC |
| **O76** | 10105197 | Ju Hua Tao | *P. persica* (L.) Batsch. | Hebei, PRC |
| **O77** | 10105199 | Sa Hong Tao | *P. persica* (L.) Batsch. | Henan, PRC |
| **O78** | 10105200 | Sa Hong Long Zhu Tao | *P. persica* (L.) Batsch. | Henan, PRC |
| **B79** | 10105201 | Chong Ban Xiao Hua Xing | *P. persica* (L.) Batsch. | Henan, PRC |
| **O80** | 10105202 | Hong Shou Xing | *P. persica* (L.) Batsch. | Hebei, PRC |
| **O81** | 10105203 | Shou Bai | *P. persica* (L.) Batsch. | Hebei, PRC |
| **O82** | 10105205 | Hong Chui Zhi | *P. persica* (L.) Batsch. | Hebei, PRC |
| **O83** | 10105206 | Zhu Fen Chui Zhi | *P. persica* (L.) Batsch. | Hebei, PRC |
| **O84** | 10105207 | Yuan Yang Chui Zhi | *P. persica* (L.) Batsch. | Hebei, PRC |

#### Supplementary Table 2. The statistical traits and phenotypes in the 84 accessions.

| **No.** | **Position** | **Main Biological Traits** | **The number of the phenotypes in the trait** |
| --- | --- | --- | --- |
| 1 | Fruit | Flesh taste | 2 |
| 2 |  | Skin hairiness | 2 |
| 3 |  | Fruit shape | 2 |
| 4 |  | Flesh firmness | 2 |
| 5 |  | Fruit flesh color | 2 |
| 6 |  | Red pigment in flesh | 5 |
| 7 |  | Flesh color around stone | 4 |
| 8 |  | Flesh adhesion | 2 |
| 9 |  | Fruit weight (g) | quantative trait |
| 10 |  | Soluble solid (%) | quantative trait |
| 11 | Seed | Kernel flavor | 2 |
| 12 | Flower | Flower type | 3 |
| 13 |  | Flower color | 4 |
| 14 |  | Petal Double | 2 |
| 15 |  | Male sterility | 2 |
| 16 | Leaves | Leaves gland | 4 |
| 17 | Branches | Broomy growth habit | 4 |
| 18 |  | Internode length (cm) | quantative trait |
| 19 | Time statistics | Fruit development period (d) | quantative trait |
| 20 |  | Plant growth period (d) | quantative trait |
| 21 |  | Chilling requirement (h) | quantative trait |

#### Supplementary Table 3. The detail phenotypes in 84 accessions.

| Number | Flesh taste | Skin hairiness | Fruit shape | Flesh firmness | Fruit flesh color | Red pigment in flesh | Flesh color around stone | Flesh adhesion | Fruit weight (g) | Soluble solid (%) | Kernel flavor | Flower type | Flower color | Petal Double | Male sterility | Leaves gland | Broomy growth habit | Internode length (cm) | Fruit development period (d) | Plant growth period (d) | Chilling requirement (h) |
| --- | --- | --- | --- | --- | --- | --- | --- | --- | --- | --- | --- | --- | --- | --- | --- | --- | --- | --- | --- | --- | --- |
| L01 | Sweet | Peach | Round | Soft | White | Few | Few | Freestone | 129.6 | 12 | Bitter | Showy flower | Pink | Single | Fertile | Reniform | Common | 2.52 | 106 | 256 | 950 |
| L02 | Sweet | Peach | Round | Soft | White | No | No | Clingstone | 126.1 | 17.2 | Bitter | Showy flower | Pink | Single | Fertile | Reniform | Common | 2.05 | 158 | 268 | 900 |
| L03 | Sweet | Peach | Round | Soft | White | Enormous | Many | Freestone | 123 | 14 | Bitter | Showy flower | Pink | Single | Fertile | Reniform | Common | 2.74 | 105 | 261 | 800 |
| L04 | Sweet | Peach | Round | Firm | White | Many | Many | Clingstone | 103.8 | 12.3 | Bitter | Showy flower | Pink | Single | Fertile | Reniform | Common | 2.1 | 88 | 256 | 950 |
| L05 | Sweet | Peach | Round | Firm | White | Medium | No | Clingstone | 118 | 12.1 | Bitter | Showy flower | Pink | Single | Sterility | Reniform | Common | 2.17 | 93 | 237 | 1050 |
| L06 | Sweet | Peach | Round | Soft | White | Many | Many | Freestone | 91.1 | 12.5 | Bitter | Showy flower | Pink | Single | Fertile | Reniform | Common | 2.6 | 88 | 258 | 800 |
| L07 | Sweet | Peach | Round | Soft | White | No | Few | Clingstone | 221.35 | 13.3 | Bitter | Showy flower | Pink | Single | Fertile | Reniform | Common | 1.89 | 141 | 256 | 1150 |
| L08 | Acid | Peach | Round | Soft | White | Medium | No | Clingstone | 51.9 | 10.3 | Bitter | Showy flower | Pink | Single | Sterility | Reniform | Common | 2.23 | 80 | 254 | 850 |
| L09 | Acid | Peach | Round | Soft | White | Few | NA | Freestone | 70 | NA | Bitter | Showy flower | Pink | Single | Fertile | Reniform | Common | 2.32 | NA | 270 | 800 |
| L10 | Acid | Peach | Round | Firm | Yellow | No | Medium | Clingstone | 125.65 | 12 | Bitter | Showy flower | Pink | Single | Fertile | Reniform | Common | 2.08 | 152 | 275 | 700 |
| L11 | Acid | Peach | Round | Soft | White | No | Few | Freestone | 111 | 11.5 | Sweet | Showy flower | Pink | Single | Fertile | Reniform | Common | 2.7 | 168 | 272 | 750 |
| L12 | Acid | Nectarine | Round | Soft | White | No | Few | Freestone | 90 | 14.7 | Bitter | Showy flower | Pink | Single | Fertile | Reniform | Common | 2.11 | 165 | 270 | 750 |
| L13 | Sweet | Peach | Round | Soft | White | No | Many | Clingstone | 129 | 11 | Bitter | Showy flower | Pink | Single | Fertile | Reniform | Common | 1.65 | 159 | 245 | 450 |
| B14 | Acid | Nectarine | Round | Firm | Yellow | Few | No | Clingstone | 109.4 | 12.7 | Bitter | Non-showy flower | Pink | Single | Fertile | Reniform | Common | 2.17 | 94 | 267 | 800 |
| B15 | Sweet | Peach | Round | Soft | Yellow | Few | Few | Clingstone | 130 | 10 | Bitter | Showy flower | Pink | Single | Fertile | Reniform | Common | 2.56 | 109 | 261 | 750 |
| B16 | Sweet | Peach | Round | Soft | White | Few | Medium | Clingstone | 90 | 10 | Bitter | Showy flower | Pink | Single | Fertile | Reniform | Common | 2.5 | 71 | NA | 750 |
| B17 | Sweet | Peach | Round | Soft | White | Many | Many | Clingstone | 115.8 | 14.5 | Bitter | Showy flower | Pink | Double | Fertile | Reniform | Common | 2.12 | 130 | 238 | 850 |
| W18 | Acid | Peach | Round | Soft | White | No | No | Freestone | 11 | 11 | Bitter | Showy flower | White | Single | Fertile | Globose | Common | 2.65 | 129 | 265 | 400 |
| W19 | Acid | Peach | Round | Soft | White | No | No | Freestone | 8.15 | 10 | Bitter | Showy flower | Pink | Single | Fertile | Globose | Broomy | 2.56 | 129 | 280 | 400 |
| W20 | Acid | Peach | Round | Soft | White | No | No | Clingstone | 16 | 13.5 | Bitter | Showy flower | Pink | Single | Fertile | Eglandular | Common | 1.67 | 154 | 278 | 400 |
| L21 | Acid | Peach | Round | Soft | Yellow | No | No | Freestone | 124 | 11.5 | Bitter | Showy flower | Pink | Single | Fertile | Reniform | Common | 2.19 | 158 | 273 | 700 |
| W22 | Acid | Peach | Round | Soft | White | Many | Many | Clingstone | 30 | 10 | Bitter | Showy flower | Pink | Single | Fertile | Globose | Common | NA | 148 | NA | 650 |
| W23 | Acid | Peach | Round | Soft | White | No | No | Clingstone | 20 | 10 | Bitter | Showy flower | Pink | Single | Fertile | Globose | Common | NA | 148 | NA | 650 |
| W24 | Acid | Peach | Round | Soft | White | No | No | Freestone | 12.9 | 11 | Bitter | Showy flower | Pink | Single | Fertile | Eglandular | Common | 1.67 | 154 | 278 | 400 |
| W25 | Acid | Peach | Round | Soft | White | No | No | Clingstone | 9.6 | 10 | Bitter | Showy flower | Pink | Single | Fertile | Globose | Common | NA | 137 | NA | 650 |
| B26 | Acid | Peach | Round | Soft | White | Few | No | Clingstone | 90.2 | 10 | Bitter | Showy flower | Pink | Single | Fertile | Reniform | Common | 2.58 | 84 | 266 | 800 |
| W27 | Acid | Peach | Round | Soft | White | No | No | Freestone | 10.8 | 11 | Bitter | Showy flower | Pink | Single | Fertile | Globose | Common | 2.22 | 125 | 280 | 400 |
| L28 | Acid | Peach | Round | Soft | White | No | No | Freestone | 48 | 16 | Sweet | Showy flower | Pink | Single | Fertile | Reniform | Common | 2.33 | 149 | 268 | 750 |
| W29 | Acid | Peach | Round | Soft | White | No | No | Freestone | 8 | 11 | Bitter | Showy flower | Pink | Single | Fertile | Globose | Common | 1.93 | 153 | 255 | 400 |
| L30 | Sweet | Peach | Round | Soft | White | No | No | Freestone | 45.5 | 14.5 | Bitter | Showy flower | Pink | Single | Fertile | Reniform | Common | 2.13 | 175 | 254 | 1100 |
| L31 | Acid | Peach | Round | Firm | Yellow | Medium | Many | Clingstone | 143.5 | 10.3 | Bitter | Showy flower | Pink | Single | Fertile | Reniform | Common | 2.48 | 116 | 266 | 700 |
| L32 | Sweet | Peach | Round | Soft | White | No | No | Clingstone | 191 | 13.3 | Bitter | Showy flower | Pink | Single | Fertile | Reniform | Common | 2.03 | 148 | 260 | 1100 |
| L33 | Sweet | Peach | Round | Soft | White | Many | Many | Freestone | 105 | 12.2 | Bitter | Showy flower | Pink | Single | Fertile | Reniform | Common | 2.13 | 146 | 274 | 200 |
| L34 | Acid | Peach | Round | Soft | Yellow | Few | Medium | Clingstone | 131 | 10.7 | Bitter | Showy flower | Pink | Single | Fertile | Reniform | Common | 1.88 | 149 | 275 | 600 |
| L35 | Acid | Peach | Round | Soft | White | Few | Many | Freestone | 94 | 13 | Bitter | Showy flower | Pink | Single | Fertile | Reniform | Common | 2.4 | 146 | 252 | 700 |
| L36 | Acid | Nectarine | Round | Soft | Yellow | Few | Few | Freestone | 85.5 | 12.2 | Bitter | Showy flower | Pink | Single | Fertile | Reniform | Common | 2.11 | 116 | 270 | 900 |
| L37 | Sweet | Peach | Flat | Soft | White | Few | Few | Freestone | 132 | 10.7 | Bitter | Showy flower | Pink | Single | Fertile | Reniform | Common | 2.63 | 118 | 258 | 800 |
| B38 | Sweet | Nectarine | Round | Firm | Yellow | No | No | Clingstone | 153 | 12 | Bitter | Non-showy flower | Pink | Single | Fertile | Globose | Common | 2.26 | 103 | 267 | 750 |
| W39 | NA | Peach | Round | NA | White | NA | NA | NA | NA | NA | NA | Showy flower | White | Double | Fertile | Reniform | Common | 2.2 | NA | NA | 400 |
| L40 | Acid | Peach | Round | Soft | White | No | Few | Freestone | 102 | 15 | Sweet | Showy flower | Pink | Single | Fertile | Reniform | Common | 2.15 | 158 | 268 | 750 |
| L41 | Acid | Peach | Flat | Soft | White | No | No | Freestone | 52 | 13 | Bitter | Showy flower | Pink | Single | Fertile | Reniform | Common | 2.19 | 157 | 263 | 950 |
| B42 | Sweet | Peach | Round | Soft | White | Medium | Many | Clingstone | 155.1 | 10.7 | Bitter | Showy flower | Pink | Single | Sterility | Reniform | Common | 2.03 | 137 | 259 | 800 |
| B43 | Acid | Nectarine | Round | Soft | Yellow | Medium | Medium | Freestone | 113.7 | 13.5 | Bitter | Showy flower | Pink | Single | Fertile | Reniform | Common | 2.34 | 121 | 252 | 850 |
| B44 | Sweet | Peach | Round | Soft | White | Few | Few | Clingstone | 127.95 | 12 | Bitter | Showy flower | Pink | Single | Fertile | Reniform | Common | 2.19 | 106 | 243 | 900 |
| L45 | Acid | Peach | Round | Soft | White | Few | Many | Clingstone | 172.15 | 11.8 | Bitter | Showy flower | Pink | Single | Sterility | Reniform | Common | 2.21 | 137 | 259 | 800 |
| B46 | Sweet | Peach | Round | Soft | White | Medium | Medium | Freestone | 159 | 11.8 | Bitter | Showy flower | Pink | Single | Fertile | Reniform | Common | 2.09 | 112 | 261 | 900 |
| L47 | Sweet | Peach | Round | Soft | White | No | No | Clingstone | 131 | 10 | Bitter | Showy flower | Pink | Single | Fertile | Reniform | Common | 2.5 | 124 | 246 | 850 |
| L48 | Sweet | Peach | Round | Soft | White | Few | No | Freestone | 111.6 | 14.3 | Bitter | Showy flower | Pink | Single | Sterility | Reniform | Common | 2.16 | 87 | 236 | 1050 |
| L49 | Acid | Peach | Round | Soft | White | Enormous | Many | Freestone | 100.3 | 10.7 | Bitter | Showy flower | Pink | Single | Fertile | Reniform | Common | 2.1 | 103 | 261 | 950 |
| B50 | Sweet | Peach | Round | Firm | White | No | No | Clingstone | 101.7 | 11 | Bitter | Showy flower | Pink | Single | Fertile | Reniform | Common | 2.03 | 103 | 260 | 900 |
| L51 | Sweet | Peach | Round | Soft | White | No | Medium | Clingstone | 224.5 | 13.3 | Bitter | Showy flower | Pink | Single | Sterility | Reniform | Common | 2.05 | 129 | 254 | 1150 |
| L52 | Sweet | Peach | Flat | Soft | White | No | No | Freestone | 89.7 | 14 | Bitter | Showy flower | Pink | Single | Fertile | Reniform | Common | 2.01 | 143 | 257 | 800 |
| L53 | Acid | Nectarine | Flat | Firm | White | No | Few | Clingstone | 36.8 | 16.5 | Bitter | Showy flower | Pink | Single | Fertile | Reniform | Common | 2.4 | 157 | 250 | 800 |
| L54 | Sweet | Peach | Flat | Firm | White | Medium | Few | Clingstone | 81.5 | 12.9 | Bitter | Showy flower | Pink | Single | Fertile | Reniform | Common | 1.89 | 87 | 258 | 850 |
| L55 | Acid | Peach | Flat | Firm | White | Few | Medium | Clingstone | 82.1 | 12 | Bitter | Showy flower | Pink | Single | Sterility | Reniform | Common | 2.28 | 144 | 266 | 750 |
| L56 | Acid | Peach | Round | Firm | Yellow | Few | Medium | Clingstone | 161.9 | 7.7 | Bitter | Showy flower | Pink | Single | Fertile | Reniform | Common | 2.6 | 104 | 269 | 700 |
| L57 | Acid | Peach | Round | Firm | Yellow | Many | Medium | Clingstone | 138 | 11.7 | Bitter | Showy flower | Pink | Single | Fertile | Reniform | Common | 2.52 | 138 | 269 | 700 |
| L58 | Sweet | Peach | Round | Soft | White | Few | Medium | Freestone | 160 | 11.9 | Bitter | Showy flower | Pink | Single | Fertile | Reniform | Common | 0.9 | 127 | 257 | 650 |
| B59 | Sweet | Peach | Round | Soft | White | Few | No | Clingstone | 80 | 11 | Bitter | Showy flower | Pink | Single | Fertile | Globose | Common | 2.44 | 78 | NA | 750 |
| B60 | Sweet | Nectarine | Round | Soft | Yellow | Medium | Few | Clingstone | 61 | 11 | Bitter | Showy flower | Pink | Single | Fertile | Reniform | Common | 2.32 | 78 | 269 | 750 |
| L61 | Sweet | Peach | Round | Soft | White | Enormous | Many | Freestone | 108 | 12 | Bitter | Showy flower | Pink | Single | Fertile | Reniform | Common | 2.76 | 99 | 254 | 750 |
| O62 | Acid | Peach | Round | Soft | White | Enormous | Many | Clingstone | 67.5 | 14 | Bitter | Showy flower | Pink | Single | Fertile | Reniform | Common | 2.24 | 148 | 261 | 850 |
| L63 | Sweet | Peach | Round | Soft | White | Many | Few | Clingstone | 158 | 9.5 | Bitter | Showy flower | Pink | Single | Sterility | Reniform | Common | 2.54 | 83 | 257 | 800 |
| L64 | Sweet | Peach | Round | Soft | White | Few | Few | Clingstone | 136 | 11 | Bitter | Showy flower | Pink | Single | Fertile | Reniform | Common | 2.18 | 126 | 239 | 850 |
| B65 | Sweet | Peach | Round | Soft | White | Few | Many | Clingstone | 260 | 17.5 | Bitter | Showy flower | Pink | Single | Fertile | Reniform | Common | 2.12 | 190 | 256 | 950 |
| B66 | Sweet | Nectarine | Round | Soft | Yellow | Few | No | Clingstone | 112.8 | 10 | Bitter | Showy flower | Pink | Single | Fertile | Reniform | Common | 2.69 | 76 | 266 | 650 |
| B67 | Sweet | Nectarine | Round | Soft | Yellow | No | No | Clingstone | 119.95 | 12.8 | Bitter | Non-showy flower | Pink | Single | Fertile | Reniform | Common | 2.645 | 91 | 256 | 800 |
| B68 | Sweet | Nectarine | Round | Soft | White | Many | Many | Clingstone | 123 | 11 | Bitter | Showy flower | Pink | Single | Fertile | Globose | Common | 2.39 | 99 | 260 | 650 |
| B69 | Sweet | Peach | Round | Soft | White | Medium | Medium | Freestone | 145.75 | 12.8 | Bitter | Showy flower | Pink | Single | Fertile | Reniform | Common | 2.42 | 136 | 250 | 800 |
| B70 | Acid | Nectarine | Round | Soft | Yellow | No | No | Clingstone | 71.9 | 8.7 | Bitter | Showy flower | Pink | Single | Fertile | Globose | Common | 1.82 | 65 | 257 | 550 |
| L71 | Sweet | Peach | Round | Soft | White | Many | Many | Clingstone | 68 | 11 | Bitter | Showy flower | Pink | Single | Fertile | Reniform | Common | 2.96 | 110 | 280 | 200 |
| B72 | Sweet | Nectarine | Round | Soft | Yellow | Few | Few | Clingstone | 125 | 13 | Bitter | Non-showy flower | Pink | Single | Fertile | Reniform | Common | 2.72 | 94 | 267 | 650 |
| L73 | Acid | Peach | Round | Soft | White | No | Medium | Clingstone | 192.5 | 15.2 | Bitter | Showy flower | Pink | Single | Sterility | Reniform | Common | 1.98 | 147 | 256 | 900 |
| B74 | Sweet | Peach | Round | Soft | White | Few | Medium | Clingstone | 172 | 14 | Bitter | Showy flower | Pink | Single | Fertile | Reniform | Common | 2.37 | 138 | 269 | 900 |
| B75 | Sweet | Nectarine | Round | Soft | White | Few | No | Clingstone | 78.4 | 13 | Bitter | Showy flower | Pink | Single | Fertile | Globose | Common | 2.34 | 76 | 265 | 650 |
| O76 | Acid | Peach | Round | Soft | White | No | No | Clingstone | 50 | 10 | Bitter | Chrysanthemum | Pink | Double | Fertile | Reniform | Common | 2.47 | 170 | 268 | 1200 |
| O77 | Acid | Peach | Round | Soft | White | Few | NA | Clingstone | 30 | 11 | Bitter | Showy flower | Mosaic | Double | Fertile | Reniform | Common | 2.11 | 164 | 246 | 1200 |
| O78 | Acid | Peach | Round | Soft | White | No | No | Clingstone | 40 | 11 | Bitter | Showy flower | Mosaic | Double | Fertile | Reniform | Broomy | NA | 155 | NA | 1000 |
| B79 | Acid | Peach | Round | Soft | White | Few | No | Clingstone | 197.4 | 13.7 | Bitter | Non-showy flower | Pink | Double | Fertile | Reniform | Common | NA | 111 | NA | 750 |
| O80 | Acid | Peach | Round | Soft | White | No | No | Freestone | 20 | 9 | Bitter | Showy flower | Red | Double | Fertile | Reniform | Dwarf | 0.65 | 146 | 273 | 900 |
| O81 | Acid | Peach | Round | Soft | White | No | No | Clingstone | 29 | 9 | Bitter | Showy flower | White | Double | Fertile | Reniform | Dwarf | 0.48 | 144 | 272 | 1000 |
| O82 | Acid | Peach | Round | Soft | White | No | No | Clingstone | 28 | 11 | Bitter | Showy flower | Red | Double | Fertile | Reniform | Willow | 2.32 | 154 | 270 | 950 |
| O83 | Acid | Peach | Round | Soft | White | No | No | Clingstone | 16 | 10 | Bitter | Showy flower | Pink | Double | Fertile | Reniform | Willow | 2.01 | 169 | 256 | 1050 |
| O84 | Acid | Peach | Round | Soft | White | No | No | Clingstone | 18 | 10 | Bitter | Showy flower | Mosaic | Double | Fertile | Reniform | Willow | 1.9 | 139 | 268 | 1150 |

#### Supplementary Table 4. Mean depth and coverage in each acession.

| **Number** | **Mean depth** | **Coverage** | **Number** | **Mean depth** | **Coverage** |
| --- | --- | --- | --- | --- | --- |
| L01 | 3.82 | 90.20% | B43 | 2.37 | 83.90% |
| L02 | 3.68 | 88.40% | B44 | 2.33 | 82.70% |
| L03 | 3.62 | 84.70% | L45 | 2.47 | 83.40% |
| L04 | 4.03 | 89.80% | B46 | 2.08 | 78.80% |
| L05 | 2.94 | 84.40% | L47 | 2.44 | 83.90% |
| L06 | 3.54 | 88.20% | L48 | 2.37 | 83.60% |
| L07 | 2.46 | 81.70% | L49 | 2.84 | 87.00% |
| L08 | 3.07 | 84.80% | B50 | 2.37 | 83.80% |
| L09 | 3.13 | 89.70% | L51 | 2.44 | 84.00% |
| L10 | 3.41 | 88.20% | L52 | 2.44 | 85.00% |
| L11 | 2.45 | 80.90% | L53 | 2.9 | 88.30% |
| L12 | 3.65 | 89.90% | L54 | 2.51 | 85.30% |
| L13 | 3.25 | 86.80% | L55 | 2.47 | 85.20% |
| B14 | 3.34 | 89.70% | L56 | 2.48 | 85.40% |
| B15 | 2.49 | 85.90% | L57 | 2.18 | 82.70% |
| B16 | 3.42 | 90.10% | L58 | 2.38 | 84.60% |
| B17 | 4.03 | 89.70% | B59 | 2.31 | 83.80% |
| W18 | 2.87 | 80.00% | B60 | 2.43 | 85.00% |
| W19 | 2.97 | 80.90% | L61 | 2.23 | 82.30% |
| W20 | 2.58 | 79.10% | O62 | 2.49 | 83.90% |
| L21 | 3.09 | 89.60% | L63 | 2.46 | 84.50% |
| W22 | 2.81 | 78.30% | L64 | 2.41 | 84.40% |
| W23 | 2.87 | 77.20% | B65 | 2.12 | 80.30% |
| W24 | 4.89 | 88.50% | B66 | 2.48 | 86.00% |
| W25 | 4.67 | 84.90% | B67 | 2.86 | 88.50% |
| B26 | 5.36 | 96.80% | B68 | 2.7 | 87.70% |
| W27 | 4.42 | 85.40% | B69 | 2.82 | 88.10% |
| L28 | 5.12 | 95.00% | B70 | 2.94 | 88.50% |
| W29 | 4.77 | 87.60% | L71 | 2.74 | 88.00% |
| L30 | 5.31 | 95.40% | B72 | 2.98 | 89.60% |
| L31 | 5.3 | 95.50% | L73 | 3.21 | 89.70% |
| L32 | 5.43 | 95.70% | B74 | 3.14 | 90.10% |
| L33 | 4.63 | 93.40% | B75 | 3.51 | 92.80% |
| L34 | 5.74 | 96.00% | O76 | 2.46 | 83.30% |
| L35 | 5.77 | 95.00% | O77 | 2.73 | 85.50% |
| L36 | 5.8 | 97.00% | O78 | 2.51 | 84.70% |
| L37 | 5.57 | 95.40% | B79 | 2.19 | 81.90% |
| B38 | 5.31 | 95.80% | O80 | 2.57 | 84.50% |
| W39 | 2.12 | 79.70% | O81 | 2.59 | 83.90% |
| L40 | 2.55 | 85.40% | O82 | 2.18 | 79.80% |
| L41 | 2.2 | 81.60% | O83 | 2.45 | 82.50% |
| B42 | 2.28 | 81.50% | O84 | 2.45 | 82.10% |

#### Supplementary Table 5. Indels (insertions and deletions) summary.

| **Groups**  **Group capacity (n)** | | **Edible**  **65** | **Ornamental**  **9** | **Wild**  **10** | **All indels**  **84** |
| --- | --- | --- | --- | --- | --- |
| **Gene region** | CDS | 11,296 | 1,291 | 7,301 | 19,888 |
|  | Intron | 101,384 | 12,935 | 124,919 | 239,238 |
| **Untranslated Region** | 3_UTR | 5,463 | 654 | 7,986 | 14,103 |
|  | 5_UTR | 2,846 | 334 | 3,188 | 6,368 |
| **Intergenic** | | 298,497 | 39,719 | 252,607 | 590,823 |
| **Whole genome** | | 419,486 | 54,933 | 396,001 | 870,420 |

#### Supplementary Table 6. SVs(Structure Variations) summary.

| **Groups**  **Group capacity (n)** | **Wild**  **10** | **Ornamental**  **9** | **Edible**  **65** | **All SVs**  **84** |
| --- | --- | --- | --- | --- |
| **Deletion** | 53,414 | 11,648 | 100,778 | 165,840 |
| **Insertion** | 5,387 | 689 | 10,914 | 16,990 |
| **Tandem Duplication** | 1,408 | 338 | 3,309 | 5,055 |
| **Dispersed Duplication** | 910 | 51 | 690 | 1,651 |
| **Other Complex SVs** | 125 | 8 | 169 | 302 |
| **Total** | 61,244 | 12,734 | 115,860 | 189,838 |
| **unique in Each group** | 3,655 | 9 | 1,671 | / |

#### Supplementary Table 7. The average polymorphism in different groups.

| **Group**  **Group capacity (n)** | | **Wild**  **10** | **Ornamental**  **9** | **Edible**  **65** | **Edible_landrace**  **39** | **Edible_breeding**  **26** | **Total**  **84** |
| --- | --- | --- | --- | --- | --- | --- | --- |
| **polymorphism *π* (10-3)** | Total genome | 6.172 | 1.868 | 2.174 | 2.140 | 1.832 | 3.405 |
|  | CDS | 5.779 | 3.371 | 2.347 | 2.634 | 2.493 | 3.833 |
|  | Intron | 7.645 | 3.789 | 2.942 | 3.210 | 2.840 | 4.792 |
| **polymorphism *θ_w_* (10-3)** | Total genome | 4.490 | 1.557 | 1.827 | 1.851 | 1.575 | 2.624 |
|  | CDS | 6.036 | 3.071 | 4.279 | 4.124 | 3.810 | 4.462 |
|  | Intron | 4.677 | 2.809 | 3.343 | 3.334 | 3.228 | 3.610 |

#### Supplementary Table 8. The statistics of the common regions under selection between every two subgroups.

| Subgroup | the other | A | B | C | D | E | F |
| --- | --- | --- | --- | --- | --- | --- | --- |
| self | Amount | 2042 | 7906 | 5440 | 5799 | 6444 | 3628 |
| A | 2042 | / | A∩B=973 | A∩C=707 | A∩D=798 | A∩E=899 | A∩F=507 |
|  |  | / | (A∩B)/B=12.307% | (A∩C)/C=12.996% | (A∩D)/D=13.761% | (A∩E)/E=13.951% | (A∩F)/F=13.975% ^※^ |
| B | 7906 | B∩A=973 | / | B∩C=2572 | B∩D=2789 | B∩E=2856 | B∩F=1647 |
|  |  | (B∩A)/A=47.649% | / | (B∩C)/C=47.279% | (B∩D)/D=48.094% | (B∩E)/E=44.320% | (B∩F)/F=45.397% ^※※^ |
| C | 5440 | C∩A=707 | C∩B=2572 | / | C∩D=2046 | C∩E=1850 | C∩F=1251 |
|  |  | (C∩A)/A=34.623% | (C∩B)/B=32.532% | / | (C∩D)/D=35.282% | (C∩E)/E=28.709% | (C∩F)/F=34.482% |
| D | 5799 | D∩A=798 | D∩B=2789 | D∩C=2046 | / | D∩E=2137 | D∩F=1203 |
|  |  | (D∩A)/A=39.079% | (D∩B)/B=35.277% | (D∩C)/C=37.610% | / | (D∩E)/E=33.163% | (D∩F)/F=33.159% |
| E | 6444 | E∩A=899 | E∩B=2856 | E∩C=1850 | E∩D=2137 | / | E∩F=1719 |
|  |  | (E∩A)/A=44.025% | (E∩B)/B=36.124% | (E∩C)/C=34.007% | (E∩D)/D=36.851% | / | (E∩F)/F=47.381% |
| F | 3628 | F∩A=507 | F∩B=1647 | F∩C=1251 | F∩D=1203 | F∩E=1719 | / |
|  |  | (F∩A)/A=24.829% | (F∩B)/B=20.832% | (F∩C)/C=22.996% | (F∩D)/D=20.745% | (F∩E)/E=26.676% | / |

^※^ A subgroup (ornamental) has very few common candidate regions with the edible subgroups (all less than 15%);

^※※^ B subgroup (edible purple) is an intermediate subgroup, because the ratios of the common candidate regions between B subgroup and other subgroups are quite high (nearly 50%), much higher than any ratios of other two subgroups

#### Supplementary Table 9. The lists of all the 147 genes under edible selection and 262 genes under ornamental selection.

**Supplementary Table 9a**. The genes and their function annotations in the regions under ornamental selection.

| Scaffold | Region under selection | | Branches | Gene | mRNA region | | synonymous | Function Annotation | Database |
| --- | --- | --- | --- | --- | --- | --- | --- | --- | --- |
|  | start point | end point |  | name/ID | start point | end point | gene ID |  |  |
| Scaffold_1 | 330000 | 339999 | yellow | ppa002982m | 333005 | 340422 | AT3G23940.1 | Arabidopsis thaliana dehydratase family | TAIR |
| Scaffold_1 | 630000 | 639999 | yellow | ppa020579m | 639233 | 641217 | MDL1_PRUDU | Prunus dulcis (Almond) (Prunus amygdalus) (R)-mandelonitrile lyase 1 | Swiss-Prot |
| Scaffold_1 | 1320000 | 1329999 | yellow | ppa002058m | 1329052 | 1333783 | LIN54_DROME | Drosophila melanogaster (Fruit fly) Protein lin-54 homolog | Swiss-Prot |
| Scaffold_1 | 1340000 | 1349999 | yellow | ppa013506m | 1347346 | 1348350 | AT1G69090.1 | Arabidopsis thaliana F-box family protein | TAIR |
| Scaffold_1 | 1730000 | 1739999 | yellow | ppa007183m | 1737127 | 1739669 | GME1_ORYSJ | Oryza sativa subsp. japonica (Rice) "GDP-mannose 3,5-epimerase 1" | Swiss-Prot |
| Scaffold_1 | 4510000 | 4519999 | yellow | ppa018049m | 4517084 | 4524997 | DNLI4_ARATH | Arabidopsis thaliana (Mouse-ear cress) DNA ligase 4 | Swiss-Prot |
| Scaffold_1 | 4860000 | 4869999 | yellow | ppa012115m | 4860322 | 4863458 | EAPP_MOUSE | Mus musculus (Mouse) E2F-associated phosphoprotein | Swiss-Prot |
| Scaffold_1 | 5010000 | 5019999 | yellow | ppa010830m | 5019913 | 5021002 | AT5G35320.1 | Arabidopsis thaliana unknown protein | TAIR |
| Scaffold_1 | 5020000 | 5029999 | yellow | ppa025097m | 5028623 | 5037496 | ISOA1_ARATH | Arabidopsis thaliana (Mouse-ear cress) "Isoamylase 1, chloroplastic" | Swiss-Prot |
| Scaffold_1 | 5320000 | 5329999 | yellow | ppa008705m | 5318937 | 5325783 | ADRM1_DANRE | Danio rerio (Zebrafish) (Brachydanio rerio) Proteasomal ubiquitin receptor ADRM1 | Swiss-Prot |
| Scaffold_1 | 5710000 | 5719999 | yellow | ppb014378m | 5718220 | 5720832 | A5ACJ6_VITVI | Vitis vinifera (Grape) Putative uncharacterized protein | TrEMBL |
| Scaffold_1 | 6310000 | 6319999 | yellow | ppa017663m | 6307947 | 6312165 | TT12_ARATH | Arabidopsis thaliana (Mouse-ear cress) Protein TRANSPARENT TESTA 12 | Swiss-Prot |
| Scaffold_1 | 6640000 | 6649999 | yellow | ppa022899m | 6642989 | 6644763 | D1H9A4_VITVI | Vitis vinifera (Grape) "Whole genome shotgun sequence of line PN40024, Scaffold_143.assembly12x" | TrEMBL |
| Scaffold_1 | 6850000 | 6859999 | yellow | ppa015992m | 6855590 | 6858994 | Y1534_ARATH | Arabidopsis thaliana (Mouse-ear cress) Probable LRR receptor-like serine/threonine-protein kinase At1g53420 | Swiss-Prot |
| Scaffold_1 | 6870000 | 6879999 | yellow | ppa012155m | 6870631 | 6871240 | AT5G57685.1 | Arabidopsis thaliana AtGDU3 (Arabidopsis thaliana GLUTAMINE DUMPER 3) | TAIR |
| Scaffold_1 | 7420000 | 7429999 | yellow | ppa022392m | 7429492 | 7432023 | COX12_SCHPO | Schizosaccharomyces pombe (Fission yeast) Cytochrome c oxidase subunit 6B | Swiss-Prot |
| Scaffold_1 | 10230000 | 10239999 | yellow | ppa003596m | 10235296 | 10237619 | AT4G15820.1 | Arabidopsis thaliana FUNCTIONS IN: molecular_function unknown;BEST Arabidopsis thaliana protein match is: emb1703 (embryo defective 1703) (TAIR:AT3G61780.1) | TAIR |
| Scaffold_1 | 14470000 | 14479999 | yellow | ppa026036m | 14469614 | 14470651 | AT4G16050.1 | Arabidopsis thaliana unknown protein | TAIR |
| Scaffold_1 | 15580000 | 15589999 | yellow | ppb017844m | 15585891 | 15586109 | N/A | N/A N/A | N/A |
| Scaffold_1 | 16600000 | 16609999 | yellow | ppa014364m | 16609512 | 16610299 | N/A | N/A N/A | N/A |
| Scaffold_1 | 16880000 | 16889999 | yellow | ppa024120m | 16884103 | 16884324 | N/A | N/A N/A | N/A |
| Scaffold_1 | 19170000 | 19179999 | yellow | ppa015530m | 19169115 | 19172659 | D1H9A4_VITVI | Vitis vinifera (Grape) "Whole genome shotgun sequence of line PN40024, Scaffold_143.assembly12x" | TrEMBL |
| Scaffold_1 | 31280000 | 31289999 | yellow | ppa001640m | 31272688 | 31282208 | AGD2_ARATH | Arabidopsis thaliana (Mouse-ear cress) ADP-ribosylation factor GTPase-activating protein AGD2 | Swiss-Prot |
| Scaffold_1 | 31720000 | 31729999 | yellow | ppa002012m | 31718963 | 31727992 | AT5G23390.1 | Arabidopsis thaliana unknown protein | TAIR |
| Scaffold_1 | 32330000 | 32339999 | yellow | ppa023913m | 32337020 | 32338972 | EXL3_ARATH | Arabidopsis thaliana (Mouse-ear cress) GDSL esterase/lipase EXL3 | Swiss-Prot |
| Scaffold_1 | 32570000 | 32579999 | yellow | ppa026043m | 32578554 | 32578918 | SNAK2_SOLTU | Solanum tuberosum (Potato) Snakin-2 | Swiss-Prot |
| Scaffold_1 | 33880000 | 33889999 | yellow | ppa015490m | 33888067 | 33889133 | YB95_ARATH | Arabidopsis thaliana (Mouse-ear cress) "Uncharacterized protein At2g39795, mitochondrial" | Swiss-Prot |
| Scaffold_1 | 34090000 | 34099999 | yellow | ppa020452m | 34097151 | 34099874 | PP344_ARATH | Arabidopsis thaliana (Mouse-ear cress) "Pentatricopeptide repeat-containing protein At4g31850, chloroplastic" | Swiss-Prot |
| Scaffold_1 | 34260000 | 34269999 | yellow | ppa005616m | 34261277 | 34263165 | NEP2_NEPGR | Nepenthes gracilis (Slender pitcher plant) Aspartic proteinase nepenthesin-2 | Swiss-Prot |
| Scaffold_1 | 41210000 | 41219999 | yellow | ppa008975m | 41215824 | 41218302 | GPMA_PARUW | Protochlamydia amoebophila (strain UWE25) "2,3-bisphosphoglycerate-dependent phosphoglycerate mutase" | Swiss-Prot |
| Scaffold_1 | 41310000 | 41319999 | yellow | ppa007001m | 41305555 | 41311006 | ERGI3_XENTR | Xenopus tropicalis (Western clawed frog) (Silurana tropicalis) Endoplasmic reticulum-Golgi intermediate compartment protein 3 | Swiss-Prot |
| Scaffold_1 | 42310000 | 42319999 | yellow | ppa002394m | 42309541 | 42318350 | ARFA_ARATH | Arabidopsis thaliana (Mouse-ear cress) Auxin response factor 1 | Swiss-Prot |
| Scaffold_1 | 44850000 | 44859999 | yellow | ppa015218m | 44855196 | 44861371 | REN3A_DANRE | Danio rerio (Zebrafish) (Brachydanio rerio) Regulator of nonsense transcripts 3A | Swiss-Prot |
| Scaffold_1 | 45150000 | 45159999 | yellow | ppa024949m | 45157162 | 45157383 | N/A | N/A N/A | N/A |
| Scaffold_1 | 46430000 | 46439999 | yellow | ppb022686m | 46439580 | 46440977 | RL142_ARATH | Arabidopsis thaliana (Mouse-ear cress) 60S ribosomal protein L14-2 | Swiss-Prot |
| Scaffold_2 | 1030000 | 1039999 | yellow | ppa026290m | 1036914 | 1038524 | AT4G31560.1 | Arabidopsis thaliana HCF153 | TAIR |
| Scaffold_2 | 2400000 | 2409999 | yellow | ppa000773m | 2402837 | 2413044 | LONH2_MAIZE | Zea mays (Maize) "Lon protease homolog 2, mitochondrial" | Swiss-Prot |
| Scaffold_2 | 2460000 | 2469999 | yellow | ppa025102m | 2465906 | 2469519 | THIO_CHLAA | Chloroflexus aurantiacus (strain ATCC 29366 / DSM 635 / J-10-fl) Thioredoxin | Swiss-Prot |
| Scaffold_2 | 3400000 | 3409999 | yellow | ppa015695m | 3396219 | 3401352 | AT2G38720.1 | Arabidopsis thaliana MAP65-5 (MICROTUBULE-ASSOCIATED PROTEIN 65-5); microtubule binding | TAIR |
| Scaffold_2 | 4380000 | 4389999 | yellow | ppb024625m | 4389990 | 4390196 | N/A | N/A N/A | N/A |
| Scaffold_2 | 9190000 | 9199999 | yellow | ppa025856m | 9194933 | 9197789 | YTX2_XENLA | Xenopus laevis (African clawed frog) Transposon TX1 uncharacterized 149 kDa protein | Swiss-Prot |
| Scaffold_2 | 10790000 | 10799999 | yellow | ppa013642m | 10789121 | 10790270 | Q9ZS84_SOLLC | Solanum lycopersicum (Tomato) (Lycopersicon esculentum) Polyprotein | TrEMBL |
| Scaffold_2 | 13840000 | 13849999 | yellow | ppa001813m | 13834375 | 13842278 | SEC23_USTMA | Ustilago maydis (Smut fungus) Protein transport protein SEC23 | Swiss-Prot |
| Scaffold_2 | 14480000 | 14489999 | yellow | ppa012700m | 14481363 | 14482494 | PIGP_ARATH | Arabidopsis thaliana (Mouse-ear cress) Phosphatidylinositol N-acetylglucosaminyltransferase subunit P | Swiss-Prot |
| Scaffold_2 | 17020000 | 17029999 | yellow | ppa014428m | 17028372 | 17029252 | AGL14_ARATH | Arabidopsis thaliana (Mouse-ear cress) Agamous-like MADS-box protein AGL14 | Swiss-Prot |
| Scaffold_2 | 17040000 | 17049999 | yellow | ppa009051m | 17047525 | 17050233 | CAF1M_ARATH | Arabidopsis thaliana (Mouse-ear cress) "CRS2-associated factor 1, mitochondrial" | Swiss-Prot |
| Scaffold_2 | 17110000 | 17119999 | yellow | ppa013561m | 17119786 | 17122172 | RBX1A_ARATH | Arabidopsis thaliana (Mouse-ear cress) RING-box protein 1a | Swiss-Prot |
| Scaffold_2 | 17140000 | 17149999 | yellow | ppa013547m | 17147208 | 17149783 | RBX1A_ARATH | Arabidopsis thaliana (Mouse-ear cress) RING-box protein 1a | Swiss-Prot |
| Scaffold_2 | 17380000 | 17389999 | yellow | ppa001292m | 17389500 | 17392223 | FLS2_ARATH | Arabidopsis thaliana (Mouse-ear cress) LRR receptor-like serine/threonine-protein kinase FLS2 | Swiss-Prot |
| Scaffold_2 | 17410000 | 17419999 | yellow | ppb020132m | 17410267 | 17413696 | N/A | N/A N/A | N/A |
| Scaffold_2 | 17720000 | 17729999 | yellow | ppa018152m | 17727936 | 17729723 | SDR1_ARATH | Arabidopsis thaliana (Mouse-ear cress) (+)-neomenthol dehydrogenase | Swiss-Prot |
| Scaffold_2 | 18580000 | 18589999 | yellow | ppa003091m | 18587695 | 18592197 | CTBP_DROME | Drosophila melanogaster (Fruit fly) C-terminal-binding protein | Swiss-Prot |
| Scaffold_2 | 19230000 | 19239999 | yellow | ppa000743m | 19233647 | 19241300 | ATX3_ARATH | Arabidopsis thaliana (Mouse-ear cress) Histone-lysine N-methyltransferase ATX3 | Swiss-Prot |
| Scaffold_2 | 20610000 | 20619999 | yellow | ppa000625m | 20603937 | 20619574 | EXOC4_ARATH | Arabidopsis thaliana (Mouse-ear cress) Probable exocyst complex component 4 | Swiss-Prot |
| Scaffold_2 | 20640000 | 20649999 | yellow | ppa003188m | 20641729 | 20648812 | VPS33_ARATH | Arabidopsis thaliana (Mouse-ear cress) Vacuolar protein sorting-associated protein 33 homolog | Swiss-Prot |
| Scaffold_2 | 20650000 | 20659999 | yellow | ppa025412m | 20658793 | 20659218 | SKP1A_ARATH | Arabidopsis thaliana (Mouse-ear cress) SKP1-like protein 1A | Swiss-Prot |
| Scaffold_2 | 20950000 | 20959999 | yellow | ppa007543m | 20959606 | 20961969 | AT5G19500.1 | Arabidopsis thaliana tryptophan/tyrosine permease family protein | TAIR |
| Scaffold_2 | 21050000 | 21059999 | yellow | ppa023509m | 21047268 | 21050870 | SCP27_ARATH | Arabidopsis thaliana (Mouse-ear cress) Serine carboxypeptidase-like 27 | Swiss-Prot |
| Scaffold_2 | 21690000 | 21699999 | yellow | ppa008409m | 21697838 | 21699890 | PEL4_ARATH | Arabidopsis thaliana (Mouse-ear cress) Probable pectate lyase 4 | Swiss-Prot |
| Scaffold_2 | 22160000 | 22169999 | yellow | ppa005160m | 22169250 | 22172409 | E137_ARATH | Arabidopsis thaliana (Mouse-ear cress) "Glucan endo-1,3-beta-glucosidase 7" | Swiss-Prot |
| Scaffold_2 | 22450000 | 22459999 | yellow | ppa012782m | 22458824 | 22461522 | U497L_ARATH | Arabidopsis thaliana (Mouse-ear cress) UPF0497 membrane protein At3g53850 | Swiss-Prot |
| Scaffold_2 | 25490000 | 25499999 | yellow | ppa009248m | 25499701 | 25500603 | CHLY_HEVBR | Hevea brasiliensis (Para rubber tree) Hevamine-A | Swiss-Prot |
| Scaffold_2 | 26220000 | 26229999 | yellow | ppa007524m | 26228162 | 26230296 | COMT1_PRUDU | Prunus dulcis (Almond) (Prunus amygdalus) Caffeic acid 3-O-methyltransferase | Swiss-Prot |
| Scaffold_2 | 26240000 | 26249999 | yellow | ppa018991m | 26246015 | 26246802 | AT4G27590.1 | Arabidopsis thaliana copper-binding protein-related | TAIR |
| Scaffold_2 | 26310000 | 26319999 | yellow | ppa002889m | 26318728 | 26322401 | ATG13_DEBHA | Debaryomyces hansenii (Yeast) (Torulaspora hansenii) Autophagy-related protein 13 | Swiss-Prot |
| Scaffold_2 | 26380000 | 26389999 | yellow | ppa001649m | 26386005 | 26390127 | Y1745_ARATH | Arabidopsis thaliana (Mouse-ear cress) Uncharacterized protein At1g51745 | Swiss-Prot |
| Scaffold_2 | 26620000 | 26629999 | yellow | ppa000413m | 26620682 | 26627917 | BAZ2B_CHICK | Gallus gallus (Chicken) Bromodomain adjacent to zinc finger domain protein 2B | Swiss-Prot |
| Scaffold_2 | 26700000 | 26709999 | yellow | ppa004553m | 26706303 | 26711777 | RS5_COXBU | Coxiella burnetii 30S ribosomal protein S5 | Swiss-Prot |
| Scaffold_2 | 26740000 | 26749999 | yellow | ppa005954m | 26729626 | 26740628 | AMP2B_ARATH | Arabidopsis thaliana (Mouse-ear cress) Methionine aminopeptidase 2B | Swiss-Prot |
| Scaffold_3 | 890000 | 899999 | yellow | ppa000072m | 888600 | 902916 | SEN1_YEAST | Saccharomyces cerevisiae (Baker's yeast) Helicase SEN1 | Swiss-Prot |
| Scaffold_3 | 1670000 | 1679999 | yellow | ppa000756m | 1675443 | 1684272 | CEBPZ_HUMAN | Homo sapiens (Human) CCAAT/enhancer-binding protein zeta | Swiss-Prot |
| Scaffold_3 | 2180000 | 2189999 | yellow | ppa023112m | 2188072 | 2190087 | RS33_ARATH | Arabidopsis thaliana (Mouse-ear cress) 40S ribosomal protein S3-3 | Swiss-Prot |
| Scaffold_3 | 3760000 | 3769999 | yellow | ppa019587m | 3769505 | 3772655 | AT2G25737.1 | Arabidopsis thaliana unknown protein | TAIR |
| Scaffold_3 | 3850000 | 3859999 | yellow | ppa019079m | 3859223 | 3864590 | FTSH4_SYNY3 | Synechocystis sp. (strain PCC 6803) Cell division protease ftsH homolog 4 | Swiss-Prot |
| Scaffold_3 | 5500000 | 5509999 | yellow | ppb024105m | 5504254 | 5506026 | RNHX1_ARATH | Arabidopsis thaliana (Mouse-ear cress) Putative ribonuclease H protein At1g65750 | Swiss-Prot |
| Scaffold_3 | 5510000 | 5519999 | yellow | ppa009989m | 5518625 | 5523952 | AT3G15790.1 | Arabidopsis thaliana MBD11; DNA binding / methyl-CpG binding | TAIR |
| Scaffold_3 | 5650000 | 5659999 | yellow | ppa022649m | 5657462 | 5661579 | RGA3_SOLBU | Solanum bulbocastanum (Wild potato) Putative disease resistance protein RGA3 | Swiss-Prot |
| Scaffold_3 | 5740000 | 5749999 | yellow | ppb019479m | 5745553 | 5747922 | RGA3_SOLBU | Solanum bulbocastanum (Wild potato) Putative disease resistance protein RGA3 | Swiss-Prot |
| Scaffold_3 | 5870000 | 5879999 | yellow | ppa006603m | 5871937 | 5875703 | FPG_DESHY | Desulfitobacterium hafniense (strain Y51) Formamidopyrimidine-DNA glycosylase | Swiss-Prot |
| Scaffold_3 | 6200000 | 6209999 | yellow | ppa006579m | 6207454 | 6213314 | LEU3_BRANA | Brassica napus (Rape) "3-isopropylmalate dehydrogenase, chloroplastic" | Swiss-Prot |
| Scaffold_3 | 6960000 | 6969999 | yellow | ppa020169m | 6967278 | 6967499 | N/A | N/A N/A | N/A |
| Scaffold_3 | 7080000 | 7089999 | yellow | ppa018475m | 7081430 | 7084743 | Y3475_ARATH | Arabidopsis thaliana (Mouse-ear cress) Probable LRR receptor-like serine/threonine-protein kinase At3g47570 | Swiss-Prot |
| Scaffold_3 | 7190000 | 7199999 | yellow | ppa013615m | 7193040 | 7194047 | N/A | N/A N/A | N/A |
| Scaffold_3 | 7220000 | 7229999 | yellow | ppa025808m | 7222352 | 7224248 | D1H9A4_VITVI | Vitis vinifera (Grape) "Whole genome shotgun sequence of line PN40024, Scaffold_143.assembly12x" | TrEMBL |
| Scaffold_3 | 7660000 | 7669999 | yellow | ppa016055m | 7664931 | 7666219 | PPR60_ARATH | Arabidopsis thaliana (Mouse-ear cress) "Pentatricopeptide repeat-containing protein At1g26900, mitochondrial" | Swiss-Prot |
| Scaffold_3 | 7670000 | 7679999 | yellow | ppa009570m | 7670812 | 7671838 | Y4102_ARATH | Arabidopsis thaliana (Mouse-ear cress) "Putative uncharacterized protein At4g01020, chloroplastic" | Swiss-Prot |
| Scaffold_3 | 7770000 | 7779999 | yellow | ppa023773m | 7777384 | 7777975 | AT1G73120.1 | Arabidopsis thaliana unknown protein | TAIR |
| Scaffold_3 | 7850000 | 7859999 | yellow | ppa015807m | 7859642 | 7860782 | DOCK8_HUMAN | Homo sapiens (Human) Dedicator of cytokinesis protein 8 | Swiss-Prot |
| Scaffold_3 | 7860000 | 7869999 | yellow | ppa015468m | 7868833 | 7871737 | HFA4B_ORYSJ | Oryza sativa subsp. japonica (Rice) Heat stress transcription factor A-4b | Swiss-Prot |
| Scaffold_3 | 7890000 | 7899999 | yellow | ppb006863m | 7899074 | 7905347 | YDB1_SCHPO | Schizosaccharomyces pombe (Fission yeast) Uncharacterized transporter C22E12.01 | Swiss-Prot |
| Scaffold_3 | 7930000 | 7939999 | yellow | ppb014370m | 7933310 | 7933675 | AT2G01050.1 | Arabidopsis thaliana nucleic acid binding / zinc ion binding | TAIR |
| Scaffold_3 | 8960000 | 8969999 | yellow | ppa020747m | 8966954 | 8967765 | ANXD1_ARATH | Arabidopsis thaliana (Mouse-ear cress) Annexin D1 | Swiss-Prot |
| Scaffold_3 | 9790000 | 9799999 | yellow | ppb017493m | 9798777 | 9802352 | YG31B_YEAST | Saccharomyces cerevisiae (Baker's yeast) Transposon Ty3-G Gag-Pol polyprotein | Swiss-Prot |
| Scaffold_3 | 12580000 | 12589999 | yellow | ppa007069m | 12579614 | 12589028 | SCRK5_ARATH | Arabidopsis thaliana (Mouse-ear cress) Putative fructokinase-5 | Swiss-Prot |
| Scaffold_3 | 15100000 | 15109999 | yellow | ppa001723m | 15103893 | 15107016 | FPA_ARATH | Arabidopsis thaliana (Mouse-ear cress) Flowering time control protein FPA | Swiss-Prot |
| Scaffold_3 | 18840000 | 18849999 | yellow | ppa007590m | 18849392 | 18852062 | YHM3_SCHPO | Schizosaccharomyces pombe (Fission yeast) Uncharacterized FCP1 homology domain-containing protein C1271.03c | Swiss-Prot |
| Scaffold_3 | 18900000 | 18909999 | yellow | ppa021452m | 18899747 | 18901162 | ANTA_GENTR | Gentiana triflora (Clustered gentian) Anthocyanin 5-aromatic acyltransferase | Swiss-Prot |
| Scaffold_3 | 18980000 | 18989999 | yellow | ppa020216m | 18979935 | 18982632 | ANTA_GENTR | Gentiana triflora (Clustered gentian) Anthocyanin 5-aromatic acyltransferase | Swiss-Prot |
| Scaffold_3 | 19580000 | 19589999 | yellow | ppa004961m | 19583491 | 19587280 | PTBP1_ARATH | Arabidopsis thaliana (Mouse-ear cress) Polypyrimidine tract-binding protein homolog 1 | Swiss-Prot |
| Scaffold_3 | 21910000 | 21919999 | yellow | ppa005931m | 21918982 | 21921442 | ERF1Z_ARATH | Arabidopsis thaliana (Mouse-ear cress) Eukaryotic peptide chain release factor subunit 1-3 | Swiss-Prot |
| Scaffold_4 | 500000 | 509999 | yellow | ppa007617m | 500764 | 503472 | UAF30_SCHPO | Schizosaccharomyces pombe (Fission yeast) Upstream activation factor subunit spp27 | Swiss-Prot |
| Scaffold_4 | 1520000 | 1529999 | yellow | ppa002497m | 1528486 | 1531795 | SRK6_BRAOE | Brassica oleracea var. acephala (Flowering kale) Putative serine/threonine-protein kinase receptor | Swiss-Prot |
| Scaffold_4 | 1530000 | 1539999 | yellow | ppa017664m | 1539617 | 1543222 | SRK6_BRAOE | Brassica oleracea var. acephala (Flowering kale) Putative serine/threonine-protein kinase receptor | Swiss-Prot |
| Scaffold_4 | 3600000 | 3609999 | yellow | ppa001307m | 3608188 | 3614082 | MEI2_SCHPO | Schizosaccharomyces pombe (Fission yeast) Meiosis protein mei2 | Swiss-Prot |
| Scaffold_4 | 3730000 | 3739999 | yellow | ppa027174m | 3739659 | 3740247 | E13B_WHEAT | Triticum aestivum (Wheat) "Glucan endo-1,3-beta-glucosidase" | Swiss-Prot |
| Scaffold_4 | 4230000 | 4239999 | yellow | ppa004073m | 4234167 | 4239454 | AT2G34640.1 | Arabidopsis thaliana PTAC12 (PLASTID TRANSCRIPTIONALLY ACTIVE12) | TAIR |
| Scaffold_4 | 4320000 | 4329999 | yellow | ppa013313m | 4329559 | 4330296 | PSAK_ARATH | Arabidopsis thaliana (Mouse-ear cress) "Photosystem I reaction center subunit psaK, chloroplastic" | Swiss-Prot |
| Scaffold_4 | 4370000 | 4379999 | yellow | ppa002587m | 4379209 | 4382865 | AT2G34670.1 | Arabidopsis thaliana proline-rich family protein | TAIR |
| Scaffold_4 | 4590000 | 4599999 | yellow | ppa020427m | 4594509 | 4595204 | WAK3_ARATH | Arabidopsis thaliana (Mouse-ear cress) Wall-associated receptor kinase 3 | Swiss-Prot |
| Scaffold_4 | 4640000 | 4649999 | yellow | ppa013448m | 4639904 | 4643419 | N/A | N/A N/A | N/A |
| Scaffold_4 | 4690000 | 4699999 | yellow | ppa024967m | 4697719 | 4700460 | WAK2_ARATH | Arabidopsis thaliana (Mouse-ear cress) Wall-associated receptor kinase 2 | Swiss-Prot |
| Scaffold_4 | 4730000 | 4739999 | yellow | ppa020949m | 4736395 | 4739933 | WAK2_ARATH | Arabidopsis thaliana (Mouse-ear cress) Wall-associated receptor kinase 2 | Swiss-Prot |
| Scaffold_4 | 4740000 | 4749999 | yellow | ppa025082m | 4748163 | 4750216 | WAK5_ARATH | Arabidopsis thaliana (Mouse-ear cress) Wall-associated receptor kinase 5 | Swiss-Prot |
| Scaffold_4 | 4770000 | 4779999 | yellow | ppa023155m | 4779321 | 4780079 | GL21_ORYSJ | Oryza sativa subsp. japonica (Rice) Putative germin-like protein 2-1 | Swiss-Prot |
| Scaffold_4 | 4780000 | 4789999 | yellow | ppa026076m | 4786682 | 4787450 | GL21_ORYSJ | Oryza sativa subsp. japonica (Rice) Putative germin-like protein 2-1 | Swiss-Prot |
| Scaffold_4 | 4810000 | 4819999 | yellow | ppa020787m | 4817812 | 4824534 | AT2G34780.1 | Arabidopsis thaliana MEE22 (MATERNAL EFFECT EMBRYO ARREST 22) | TAIR |
| Scaffold_4 | 5040000 | 5049999 | yellow | ppa018446m | 5039565 | 5041106 | RETOL_ARATH | Arabidopsis thaliana (Mouse-ear cress) Reticuline oxidase-like protein | Swiss-Prot |
| Scaffold_4 | 5150000 | 5159999 | yellow | ppa004535m | 5152653 | 5155480 | WRK14_ARATH | Arabidopsis thaliana (Mouse-ear cress) Probable WRKY transcription factor 14 | Swiss-Prot |
| Scaffold_4 | 5190000 | 5199999 | yellow | ppa006315m | 5199428 | 5204798 | ARAE1_ARATH | Arabidopsis thaliana (Mouse-ear cress) UDP-arabinose 4-epimerase 1 | Swiss-Prot |
| Scaffold_4 | 5310000 | 5319999 | yellow | ppa010619m | 5317466 | 5320763 | NMNA1_HUMAN | Homo sapiens (Human) Nicotinamide mononucleotide adenylyltransferase 1 | Swiss-Prot |
| Scaffold_4 | 5350000 | 5359999 | yellow | ppa000890m | 5347023 | 5353415 | Y1561_ARATH | Arabidopsis thaliana (Mouse-ear cress) Probable LRR receptor-like serine/threonine-protein kinase At1g56130 | Swiss-Prot |
| Scaffold_4 | 5920000 | 5929999 | yellow | ppa023652m | 5923161 | 5929267 | SEY1_DICDI | Dictyostelium discoideum (Slime mold) Protein SEY1 homolog | Swiss-Prot |
| Scaffold_4 | 6200000 | 6209999 | yellow | ppa004903m | 6203915 | 6210952 | AT1G31070.2 | Arabidopsis thaliana UDP-N-acetylglucosamine pyrophosphorylase-related | TAIR |
| Scaffold_4 | 6280000 | 6289999 | yellow | ppa012179m | 6287959 | 6288664 | ATL5B_ARATH | Arabidopsis thaliana (Mouse-ear cress) RING-H2 finger protein ATL5B | Swiss-Prot |
| Scaffold_4 | 6570000 | 6579999 | yellow | ppa000615m | 6575076 | 6578483 | PRP16_BOVIN | Bos taurus (Bovine) Pre-mRNA-splicing factor ATP-dependent RNA helicase PRP16 | Swiss-Prot |
| Scaffold_4 | 8890000 | 8899999 | yellow | ppa009145m | 8896182 | 8899115 | AT3G14750.1 | Arabidopsis thaliana unknown protein | TAIR |
| Scaffold_4 | 10080000 | 10089999 | yellow | ppa003461m | 10077453 | 10083438 | YCF45_PORPU | Porphyra purpurea Uncharacterized protein ycf45 | Swiss-Prot |
| Scaffold_4 | 10320000 | 10329999 | yellow | ppa011129m | 10329524 | 10330247 | CN138_MOUSE | Mus musculus (Mouse) Uncharacterized protein C14orf138 homolog | Swiss-Prot |
| Scaffold_4 | 14500000 | 14509999 | yellow | ppa021198m | 14500258 | 14501142 | AT5G55390.1 | Arabidopsis thaliana EDM2; transcription factor | TAIR |
| Scaffold_4 | 16570000 | 16579999 | yellow | ppa002305m | 16579565 | 16583575 | COG6_BOVIN | Bos taurus (Bovine) Conserved oligomeric Golgi complex subunit 6 | Swiss-Prot |
| Scaffold_4 | 16580000 | 16589999 | yellow | ppa001549m | 16589115 | 16592033 | SF3A1_ARATH | Arabidopsis thaliana (Mouse-ear cress) Probable splicing factor 3A subunit 1 | Swiss-Prot |
| Scaffold_4 | 16670000 | 16679999 | yellow | ppa019764m | 16677111 | 16677781 | AT1G09520.1 | Arabidopsis thaliana protein binding / zinc ion binding | TAIR |
| Scaffold_4 | 17360000 | 17369999 | yellow | ppb012970m | 17369501 | 17369877 | COGT2_ARATH | Arabidopsis thaliana (Mouse-ear cress) Cytokinin-O-glucosyltransferase 2 | Swiss-Prot |
| Scaffold_4 | 18230000 | 18239999 | yellow | ppa019921m | 18230874 | 18236461 | LPAT2_BRANA | Brassica napus (Rape) 1-acyl-sn-glycerol-3-phosphate acyltransferase 2 | Swiss-Prot |
| Scaffold_4 | 23190000 | 23199999 | yellow | ppa025506m | 23192404 | 23192625 | N/A | N/A N/A | N/A |
| Scaffold_4 | 26710000 | 26719999 | yellow | ppb016991m | 26712009 | 26712227 | N/A | N/A N/A | N/A |
| Scaffold_4 | 27840000 | 27849999 | yellow | ppa016386m | 27843764 | 27844325 | AT3G19430.1 | Arabidopsis thaliana late embryogenesis abundant protein-related / LEA protein-related | TAIR |
| Scaffold_4 | 29800000 | 29809999 | yellow | ppa019671m | 29807676 | 29809402 | EFR_ARATH | Arabidopsis thaliana (Mouse-ear cress) LRR receptor-like serine/threonine-protein kinase EFR | Swiss-Prot |
| Scaffold_5 | 790000 | 799999 | yellow | ppa001063m | 784870 | 792831 | AT5G43130.1 | Arabidopsis thaliana TAF4 (TBP-ASSOCIATED FACTOR 4); transcription initiation factor | TAIR |
| Scaffold_5 | 1470000 | 1479999 | yellow | ppa012828m | 1477289 | 1479342 | U497B_VITVI | Vitis vinifera (Grape) UPF0497 membrane protein 11 | Swiss-Prot |
| Scaffold_5 | 1500000 | 1509999 | yellow | ppa014756m | 1502810 | 1508438 | Y492_MYCTU | Mycobacterium tuberculosis Uncharacterized GMC-type oxidoreductase Rv0492c/MT0511/MT0512 | Swiss-Prot |
| Scaffold_5 | 1700000 | 1709999 | yellow | ppa011789m | 1706036 | 1711188 | AT4G19400.1 | Arabidopsis thaliana actin binding | TAIR |
| Scaffold_5 | 1720000 | 1729999 | yellow | ppa008825m | 1726809 | 1731523 | DUS2L_HUMAN | Homo sapiens (Human) tRNA-dihydrouridine synthase 2-like | Swiss-Prot |
| Scaffold_5 | 1810000 | 1819999 | yellow | ppb023334m | 1815081 | 1816997 | Q8W3A4_ORYSA | Oryza sativa (Rice) Putative gag-pol polyprotein | TrEMBL |
| Scaffold_5 | 1850000 | 1859999 | yellow | ppa003168m | 1857996 | 1871009 | FEI1_ARATH | Arabidopsis thaliana (Mouse-ear cress) LRR receptor-like serine/threonine-protein kinase FEI 1 | Swiss-Prot |
| Scaffold_5 | 1870000 | 1879999 | yellow | ppa013644m | 1878611 | 1884227 | B9MUE4_POPTR | Populus trichocarpa (Western balsam poplar) (Populus balsamifera subsp. trichocarpa) Predicted protein | TrEMBL |
| Scaffold_5 | 2000000 | 2009999 | yellow | ppa026000m | 2004139 | 2009791 | RECQ1_HUMAN | Homo sapiens (Human) ATP-dependent DNA helicase Q1 | Swiss-Prot |
| Scaffold_5 | 2020000 | 2029999 | yellow | ppa027028m | 2028615 | 2029027 | AT2G13770.1 | Arabidopsis thaliana unknown protein | TAIR |
| Scaffold_5 | 2040000 | 2049999 | yellow | ppa014817m | 2041010 | 2042704 | AOX1A_ARATH | Arabidopsis thaliana (Mouse-ear cress) "Alternative oxidase 1a, mitochondrial" | Swiss-Prot |
| Scaffold_5 | 2060000 | 2069999 | yellow | ppa000927m | 2065546 | 2079300 | AGL2_BACTQ | Bacillus thermoamyloliquefaciens Alpha-glucosidase 2 | Swiss-Prot |
| Scaffold_5 | 2080000 | 2089999 | yellow | ppa024249m | 2082828 | 2086675 | TMVRN_NICGU | Nicotiana glutinosa (Tobacco) TMV resistance protein N | Swiss-Prot |
| Scaffold_5 | 2090000 | 2099999 | yellow | ppa000575m | 2095988 | 2105374 | FH14_ARATH | Arabidopsis thaliana (Mouse-ear cress) Formin-like protein 14 | Swiss-Prot |
| Scaffold_5 | 2110000 | 2119999 | yellow | ppa018396m | 2119805 | 2121972 | RH2_ARATH | Arabidopsis thaliana (Mouse-ear cress) DEAD-box ATP-dependent RNA helicase 2 | Swiss-Prot |
| Scaffold_5 | 2130000 | 2139999 | yellow | ppa011506m | 2132335 | 2134649 | RL131_ARATH | Arabidopsis thaliana (Mouse-ear cress) 60S ribosomal protein L13-1 | Swiss-Prot |
| Scaffold_5 | 2180000 | 2189999 | yellow | ppa010532m | 2187108 | 2188759 | Y381_RICFE | Rickettsia felis (Rickettsia azadi) Putative ankyrin repeat protein RF_0381 | Swiss-Prot |
| Scaffold_5 | 2190000 | 2199999 | yellow | ppa024275m | 2195736 | 2200224 | D1IVR9_VITVI | Vitis vinifera (Grape) "Whole genome shotgun sequence of line PN40024, Scaffold_33.assembly12x" | TrEMBL |
| Scaffold_5 | 2230000 | 2239999 | yellow | ppa017316m | 2239080 | 2240766 | M310_ARATH | Arabidopsis thaliana (Mouse-ear cress) Uncharacterized mitochondrial protein AtMg00310 | Swiss-Prot |
| Scaffold_5 | 2310000 | 2319999 | yellow | ppa027062m | 2319223 | 2321152 | ZMYM1_HUMAN | Homo sapiens (Human) Zinc finger MYM-type protein 1 | Swiss-Prot |
| Scaffold_5 | 2410000 | 2419999 | yellow | ppa000750m | 2415750 | 2426878 | NU160_MOUSE | Mus musculus (Mouse) Nuclear pore complex protein Nup160 | Swiss-Prot |
| Scaffold_5 | 2450000 | 2459999 | yellow | ppa011305m | 2449132 | 2459627 | MOB1_ARATH | Arabidopsis thaliana (Mouse-ear cress) Mps one binder kinase activator-like 1 | Swiss-Prot |
| Scaffold_5 | 3280000 | 3289999 | yellow | ppa019223m | 3277559 | 3280575 | D1IKJ8_VITVI | Vitis vinifera (Grape) "Whole genome shotgun sequence of line PN40024, Scaffold_12.assembly12x" | TrEMBL |
| Scaffold_5 | 3410000 | 3419999 | yellow | ppa014792m | 3416210 | 3420889 | ROC3_ORYSJ | Oryza sativa subsp. japonica (Rice) Homeobox-leucine zipper protein ROC3 | Swiss-Prot |
| Scaffold_5 | 3510000 | 3519999 | yellow | ppa022098m | 3515765 | 3517094 | A5BLY4_VITVI | Vitis vinifera (Grape) Putative uncharacterized protein | TrEMBL |
| Scaffold_5 | 3620000 | 3629999 | yellow | ppa000340m | 3627867 | 3635541 | AB2B_ARATH | Arabidopsis thaliana (Mouse-ear cress) ABC transporter B family member 2 | Swiss-Prot |
| Scaffold_5 | 4000000 | 4009999 | yellow | ppa006556m | 4000692 | 4006526 | AGD8_ARATH | Arabidopsis thaliana (Mouse-ear cress) Probable ADP-ribosylation factor GTPase-activating protein AGD8 | Swiss-Prot |
| Scaffold_5 | 4040000 | 4049999 | yellow | ppb021022m | 4046473 | 4048275 | AT5G43400.1 | Arabidopsis thaliana unknown protein | TAIR |
| Scaffold_5 | 4060000 | 4069999 | yellow | ppb020591m | 4063730 | 4065532 | AT5G43400.1 | Arabidopsis thaliana unknown protein | TAIR |
| Scaffold_5 | 4500000 | 4509999 | yellow | ppa003243m | 4508047 | 4512981 | SYFB_ARATH | Arabidopsis thaliana (Mouse-ear cress) Probable phenylalanyl-tRNA synthetase beta chain | Swiss-Prot |
| Scaffold_5 | 4550000 | 4559999 | yellow | ppa022548m | 4556573 | 4562840 | Y4523_ARATH | Arabidopsis thaliana (Mouse-ear cress) Probable serine/threonine-protein kinase At4g35230 | Swiss-Prot |
| Scaffold_5 | 4620000 | 4629999 | yellow | ppb023529m | 4624850 | 4625237 | N/A | N/A N/A | N/A |
| Scaffold_5 | 4680000 | 4689999 | yellow | ppa004974m | 4685123 | 4692247 | KPYG_TOBAC | Nicotiana tabacum (Common tobacco) "Pyruvate kinase isozyme G, chloroplastic" | Swiss-Prot |
| Scaffold_5 | 4720000 | 4729999 | yellow | ppa014425m | 4728262 | 4730474 | SC61G_ORYSJ | Oryza sativa subsp. japonica (Rice) Protein transport protein Sec61 subunit gamma | Swiss-Prot |
| Scaffold_5 | 4730000 | 4739999 | yellow | ppa002576m | 4732208 | 4734298 | ILVB2_TOBAC | Nicotiana tabacum (Common tobacco) "Acetolactate synthase 2, chloroplastic" | Swiss-Prot |
| Scaffold_5 | 4850000 | 4859999 | yellow | ppa016105m | 4858746 | 4860521 | A5ACJ6_VITVI | Vitis vinifera (Grape) Putative uncharacterized protein | TrEMBL |
| Scaffold_5 | 5890000 | 5899999 | yellow | ppa009803m | 5887693 | 5892435 | YLBA_ECOLI | Escherichia coli (strain K12) Uncharacterized protein ylbA | Swiss-Prot |
| Scaffold_5 | 5940000 | 5949999 | yellow | ppa026189m | 5947202 | 5953031 | TARB1_HUMAN | Homo sapiens (Human) Probable methyltransferase TARBP1 | Swiss-Prot |
| Scaffold_5 | 6740000 | 6749999 | yellow | ppa009707m | 6745332 | 6746183 | EF100_ARATH | Arabidopsis thaliana (Mouse-ear cress) Ethylene-responsive transcription factor 1A | Swiss-Prot |
| Scaffold_5 | 9800000 | 9809999 | yellow | ppa015340m | 9803845 | 9805747 | D1H9A4_VITVI | Vitis vinifera (Grape) "Whole genome shotgun sequence of line PN40024, Scaffold_143.assembly12x" | TrEMBL |
| Scaffold_5 | 10210000 | 10219999 | yellow | ppa023932m | 10218042 | 10221574 | SEUSS_ARATH | Arabidopsis thaliana (Mouse-ear cress) Transcriptional corepressor SEUSS | Swiss-Prot |
| Scaffold_5 | 9330000 | 9339999 | yellow | ppa019592m | 9333135 | 9333674 | CML13_ARATH | Arabidopsis thaliana (Mouse-ear cress) Probable calcium-binding protein CML13 | Swiss-Prot |
| Scaffold_5 | 9510000 | 9519999 | yellow | ppa004487m | 9515392 | 9517147 | S47A1_MOUSE | Mus musculus (Mouse) Multidrug and toxin extrusion protein 1 | Swiss-Prot |
| Scaffold_6 | 2070000 | 2079999 | yellow | ppa004913m | 2079255 | 2082024 | WNK4_ARATH | Arabidopsis thaliana (Mouse-ear cress) Probable serine/threonine-protein kinase WNK4 | Swiss-Prot |
| Scaffold_6 | 2180000 | 2189999 | yellow | ppa004544m | 2186962 | 2190064 | TCMO_PHAAU | Phaseolus aureus (Mung bean) (Vigna radiata) Trans-cinnamate 4-monooxygenase | Swiss-Prot |
| Scaffold_6 | 2240000 | 2249999 | yellow | ppa006246m | 2244417 | 2248850 | PRS6B_ARATH | Arabidopsis thaliana (Mouse-ear cress) 26S protease regulatory subunit 6B homolog | Swiss-Prot |
| Scaffold_6 | 2770000 | 2779999 | yellow | ppa008513m | 2777768 | 2779913 | TDPZ4_MOUSE | Mus musculus (Mouse) TD and POZ domain-containing protein 4 | Swiss-Prot |
| Scaffold_6 | 3290000 | 3299999 | yellow | ppa024429m | 3299540 | 3302459 | TT1_ARATH | Arabidopsis thaliana (Mouse-ear cress) Protein TRANSPARENT TESTA 1 | Swiss-Prot |
| Scaffold_6 | 3930000 | 3939999 | yellow | ppb016735m | 3938748 | 3940945 | A5AWV0_VITVI | Vitis vinifera (Grape) Putative uncharacterized protein | TrEMBL |
| Scaffold_6 | 7220000 | 7229999 | yellow | ppa008177m | 7224909 | 7226913 | Y1689_ARATH | Arabidopsis thaliana (Mouse-ear cress) Uncharacterized membrane protein At1g06890 | Swiss-Prot |
| Scaffold_6 | 7940000 | 7949999 | yellow | ppa026488m | 7939700 | 7941433 | AT2G28120.1 | Arabidopsis thaliana nodulin family protein | TAIR |
| Scaffold_6 | 8750000 | 8759999 | yellow | ppa001268m | 8743871 | 8751298 | AT5G22640.1 | Arabidopsis thaliana emb1211 (embryo defective 1211) | TAIR |
| Scaffold_6 | 8770000 | 8779999 | yellow | ppa020464m | 8779985 | 8780564 | PSK6_ARATH | Arabidopsis thaliana (Mouse-ear cress) Putative phytosulfokines 6 | Swiss-Prot |
| Scaffold_6 | 9350000 | 9359999 | yellow | ppa018042m | 9352374 | 9354211 | AT3G19430.1 | Arabidopsis thaliana late embryogenesis abundant protein-related / LEA protein-related | TAIR |
| Scaffold_6 | 9830000 | 9839999 | yellow | ppa001509m | 9836289 | 9841397 | PARP3_SOYBN | Glycine max (Soybean) Poly [ADP-ribose] polymerase 3 | Swiss-Prot |
| Scaffold_6 | 11530000 | 11539999 | yellow | ppa021465m | 11534883 | 11540615 | NEK5_ARATH | Arabidopsis thaliana (Mouse-ear cress) Serine/threonine-protein kinase Nek5 | Swiss-Prot |
| Scaffold_6 | 11840000 | 11849999 | yellow | ppa024653m | 11845200 | 11849278 | BGL24_ORYSJ | Oryza sativa subsp. japonica (Rice) Beta-glucosidase 24 | Swiss-Prot |
| Scaffold_6 | 11970000 | 11979999 | yellow | ppa002898m | 11968323 | 11970611 | AT5G22090.1 | Arabidopsis thaliana unknown protein | TAIR |
| Scaffold_6 | 12000000 | 12009999 | yellow | ppa010651m | 12007523 | 12013134 | SPF31_SCHPO | Schizosaccharomyces pombe (Fission yeast) J domain-containing protein spf31 | Swiss-Prot |
| Scaffold_6 | 12050000 | 12059999 | yellow | ppa015176m | 12053169 | 12055556 | A5BEV8_VITVI | Vitis vinifera (Grape) Putative uncharacterized protein | TrEMBL |
| Scaffold_6 | 12080000 | 12089999 | yellow | ppa018003m | 12081909 | 12084543 | RPM1_ARATH | Arabidopsis thaliana (Mouse-ear cress) Disease resistance protein RPM1 | Swiss-Prot |
| Scaffold_6 | 12190000 | 12199999 | yellow | ppa010930m | 12192186 | 12197666 | Y2179_DICDI | Dictyostelium discoideum (Slime mold) Putative phosphatidylglycerol/phosphatidylinositol transfer protein DDB_G0282179 | Swiss-Prot |
| Scaffold_6 | 12540000 | 12549999 | yellow | ppa025059m | 12548362 | 12549758 | D1H9A4_VITVI | Vitis vinifera (Grape) "Whole genome shotgun sequence of line PN40024, Scaffold_143.assembly12x" | TrEMBL |
| Scaffold_6 | 12580000 | 12589999 | yellow | ppa003246m | 12587366 | 12594034 | FKB42_ARATH | Arabidopsis thaliana (Mouse-ear cress) 42 kDa peptidyl-prolyl isomerase | Swiss-Prot |
| Scaffold_6 | 13220000 | 13229999 | yellow | ppa020926m | 13220901 | 13225082 | TMVRN_NICGU | Nicotiana glutinosa (Tobacco) TMV resistance protein N | Swiss-Prot |
| Scaffold_6 | 13240000 | 13249999 | yellow | ppa009910m | 13246594 | 13250421 | DAPB2_ARATH | Arabidopsis thaliana (Mouse-ear cress) "Dihydrodipicolinate reductase 2, chloroplastic" | Swiss-Prot |
| Scaffold_6 | 13340000 | 13349999 | yellow | ppa011041m | 13344667 | 13349077 | AT5G21070.1 | Arabidopsis thaliana unknown protein | TAIR |
| Scaffold_6 | 13480000 | 13489999 | yellow | ppa000990m | 13474100 | 13482168 | AGO4A_ORYSJ | Oryza sativa subsp. japonica (Rice) Protein argonaute 4A | Swiss-Prot |
| Scaffold_6 | 13490000 | 13499999 | yellow | ppa011333m | 13490428 | 13494409 | SERK1_ARATH | Arabidopsis thaliana (Mouse-ear cress) Somatic embryogenesis receptor kinase 1 | Swiss-Prot |
| Scaffold_6 | 13580000 | 13589999 | yellow | ppa001740m | 13580380 | 13587267 | AT2G27090.1 | Arabidopsis thaliana unknown protein | TAIR |
| Scaffold_6 | 13590000 | 13599999 | yellow | ppb022979m | 13594552 | 13598143 | D1H9A4_VITVI | Vitis vinifera (Grape) "Whole genome shotgun sequence of line PN40024, Scaffold_143.assembly12x" | TrEMBL |
| Scaffold_6 | 13600000 | 13609999 | yellow | ppa023249m | 13605021 | 13605287 | N/A | N/A | N/A |
| Scaffold_6 | 13620000 | 13629999 | yellow | ppb022054m | 13626016 | 13626237 | N/A | N/A | N/A |
| Scaffold_6 | 13630000 | 13639999 | yellow | ppa001319m | 13639324 | 13647668 | LARP1_MOUSE | Mus musculus (Mouse) La-related protein 1 | Swiss-Prot |
| Scaffold_6 | 13720000 | 13729999 | yellow | ppa020595m | 13728144 | 13730313 | POLX_TOBAC | Nicotiana tabacum (Common tobacco) Retrovirus-related Pol polyprotein from transposon TNT 1-94 | Swiss-Prot |
| Scaffold_6 | 14180000 | 14189999 | yellow | ppa012729m | 14184980 | 14187997 | RL17_GEOSL | Geobacter sulfurreducens 50S ribosomal protein L17 | Swiss-Prot |
| Scaffold_6 | 14270000 | 14279999 | yellow | ppa026328m | 14267817 | 14270627 | M310_ARATH | Arabidopsis thaliana (Mouse-ear cress) Uncharacterized mitochondrial protein AtMg00310 | Swiss-Prot |
| Scaffold_6 | 14470000 | 14479999 | yellow | ppa026969m | 14466967 | 14471009 | CRK10_ARATH | Arabidopsis thaliana (Mouse-ear cress) Cysteine-rich receptor-like protein kinase 10 | Swiss-Prot |
| Scaffold_6 | 14760000 | 14769999 | yellow | ppa016888m | 14759818 | 14760668 | N/A | N/A | N/A |
| Scaffold_6 | 15900000 | 15909999 | yellow | ppa014715m | 15904920 | 15906020 | SKIP6_ARATH | Arabidopsis thaliana (Mouse-ear cress) F-box/kelch-repeat protein SKIP6 | Swiss-Prot |
| Scaffold_6 | 20040000 | 20049999 | yellow | ppa003728m | 20048926 | 20051504 | DMTF1_HUMAN | Homo sapiens (Human) Cyclin-D-binding Myb-like transcription factor 1 | Swiss-Prot |
| Scaffold_6 | 20070000 | 20079999 | yellow | ppa002424m | 20066599 | 20072111 | PCKA_ARATH | Arabidopsis thaliana (Mouse-ear cress) Phosphoenolpyruvate carboxykinase [ATP] | Swiss-Prot |
| Scaffold_6 | 20170000 | 20179999 | yellow | ppa025661m | 20176525 | 20179338 | HXKL2_ARATH | Arabidopsis thaliana (Mouse-ear cress) Probable hexokinase-like 2 protein | Swiss-Prot |
| Scaffold_6 | 21650000 | 21659999 | yellow | ppa001536m | 21644503 | 21651166 | TKI1_ARATH | Arabidopsis thaliana (Mouse-ear cress) TSL-kinase interacting protein 1 | Swiss-Prot |
| Scaffold_6 | 22050000 | 22059999 | yellow | ppa023602m | 22058079 | 22058642 | N/A | N/A | N/A |
| Scaffold_6 | 22740000 | 22749999 | yellow | ppa008713m | 22745312 | 22748077 | RDH12_MOUSE | Mus musculus (Mouse) Retinol dehydrogenase 12 | Swiss-Prot |
| Scaffold_7 | 1110000 | 1119999 | yellow | ppa011317m | 1108894 | 1111843 | AT3G56820.1 | Arabidopsis thaliana unknown protein | TAIR |
| Scaffold_7 | 1180000 | 1189999 | yellow | ppa009512m | 1174649 | 1181452 | APL_ARATH | Arabidopsis thaliana (Mouse-ear cress) Myb family transcription factor APL | Swiss-Prot |
| Scaffold_7 | 2590000 | 2599999 | yellow | ppa020288m | 2595954 | 2598051 | MX_DICLA | Dicentrarchus labrax (European sea bass) Interferon-induced GTP-binding protein Mx | Swiss-Prot |
| Scaffold_7 | 2950000 | 2959999 | yellow | ppa013037m | 2948705 | 2951617 | RPAB2_RAT | Rattus norvegicus (Rat) "DNA-directed RNA polymerases I, II, and III subunit RPABC2" | Swiss-Prot |
| Scaffold_7 | 3340000 | 3349999 | yellow | ppa001746m | 3342864 | 3347699 | Y1143_ARATH | Arabidopsis thaliana (Mouse-ear cress) Probable LRR receptor-like serine/threonine-protein kinase At1g14390 | Swiss-Prot |
| Scaffold_7 | 4020000 | 4029999 | yellow | ppa015806m | 4026892 | 4028935 | EXT1_HUMAN | Homo sapiens (Human) Exostosin-1 | Swiss-Prot |
| Scaffold_7 | 4290000 | 4299999 | yellow | ppa001125m | 4285640 | 4292806 | IPO5_HUMAN | Homo sapiens (Human) Importin-5 | Swiss-Prot |
| Scaffold_7 | 4310000 | 4319999 | yellow | ppa011287m | 4317881 | 4319896 | AT1G55340.1 | Arabidopsis thaliana unknown protein | TAIR |
| Scaffold_7 | 5390000 | 5399999 | yellow | ppa021006m | 5394186 | 5394752 | N/A | N/A | N/A |
| Scaffold_7 | 5730000 | 5739999 | yellow | ppa019409m | 5736122 | 5739666 | D1H9A4_VITVI | Vitis vinifera (Grape) "Whole genome shotgun sequence of line PN40024, Scaffold_143.assembly12x" | TrEMBL |
| Scaffold_7 | 6910000 | 6919999 | yellow | ppb016802m | 6911610 | 6911954 | N/A | N/A | N/A |
| Scaffold_7 | 6930000 | 6939999 | yellow | ppa018323m | 6937553 | 6938179 | TMVRN_NICGU | Nicotiana glutinosa (Tobacco) TMV resistance protein N | Swiss-Prot |
| Scaffold_7 | 7550000 | 7559999 | yellow | ppa023889m | 7559792 | 7560280 | N/A | N/A | N/A |
| Scaffold_7 | 7570000 | 7579999 | yellow | ppb014228m | 7579700 | 7580132 | A5BEV8_VITVI | Vitis vinifera (Grape) Putative uncharacterized protein | TrEMBL |
| Scaffold_7 | 10130000 | 10139999 | yellow | ppa024220m | 10137978 | 10139014 | AT3G52110.1 | Arabidopsis thaliana unknown protein | TAIR |
| Scaffold_7 | 10390000 | 10399999 | yellow | ppa025467m | 10398512 | 10400477 | PP227_ARATH | Arabidopsis thaliana (Mouse-ear cress) "Putative pentatricopeptide repeat-containing protein At3g13770, mitochondrial" | Swiss-Prot |
| Scaffold_7 | 11830000 | 11839999 | yellow | ppa015039m | 11829298 | 11830761 | PP201_ARATH | Arabidopsis thaliana (Mouse-ear cress) Pentatricopeptide repeat-containing protein At2g44880 | Swiss-Prot |
| Scaffold_7 | 13980000 | 13989999 | yellow | ppa017738m | 13987467 | 13989314 | HSP7C_PETHY | Petunia hybrida (Petunia) Heat shock cognate 70 kDa protein | Swiss-Prot |
| Scaffold_7 | 13990000 | 13999999 | yellow | ppa020538m | 13994460 | 13996384 | HSP7C_PETHY | Petunia hybrida (Petunia) Heat shock cognate 70 kDa protein | Swiss-Prot |
| Scaffold_7 | 15030000 | 15039999 | yellow | ppa023784m | 15028865 | 15030661 | PME15_ARATH | Arabidopsis thaliana (Mouse-ear cress) Probable pectinesterase 15 | Swiss-Prot |
| Scaffold_7 | 18170000 | 18179999 | yellow | ppa011922m | 18179453 | 18180885 | CA057_BOVIN | Bos taurus (Bovine) Nucleoside-triphosphatase C1orf57 homolog | Swiss-Prot |
| Scaffold_7 | 22080000 | 22089999 | yellow | ppa005254m | 22085223 | 22088497 | BH066_ARATH | Arabidopsis thaliana (Mouse-ear cress) Transcription factor bHLH66 | Swiss-Prot |
| Scaffold_8 | 1330000 | 1339999 | yellow | ppa026764m | 1328670 | 1331578 | Q7F966_ORYSJ | Oryza sativa subsp. japonica (Rice) OSJNBa0091C07.2 protein | TrEMBL |
| Scaffold_8 | 3270000 | 3279999 | yellow | ppa016020m | 3277570 | 3278537 | GL17_ARATH | Arabidopsis thaliana (Mouse-ear cress) Germin-like protein subfamily 1 member 7 | Swiss-Prot |
| Scaffold_8 | 8180000 | 8189999 | yellow | ppa002411m | 8179946 | 8183458 | RPT3_ARATH | Arabidopsis thaliana (Mouse-ear cress) Root phototropism protein 3 | Swiss-Prot |
| Scaffold_8 | 8190000 | 8199999 | yellow | ppa023069m | 8196066 | 8200497 | AT1G19260.1 | Arabidopsis thaliana hAT dimerisation domain-containing protein | TAIR |
| Scaffold_8 | 11440000 | 11449999 | yellow | ppa001492m | 11432297 | 11442422 | VATM_DICDI | Dictyostelium discoideum (Slime mold) Vacuolar proton translocating ATPase 100 kDa subunit | Swiss-Prot |
| Scaffold_8 | 13860000 | 13869999 | yellow | ppa019027m | 13867371 | 13867694 | B9IDT5_POPTR | Populus trichocarpa (Western balsam poplar) (Populus balsamifera subsp. trichocarpa) Predicted protein | TrEMBL |
| Scaffold_8 | 17600000 | 17609999 | yellow | ppa007372m | 17607435 | 17610433 | ACOT9_MOUSE | Mus musculus (Mouse) "Acyl-coenzyme A thioesterase 9, mitochondrial" | Swiss-Prot |
| Scaffold_8 | 17660000 | 17669999 | yellow | ppa020331m | 17669750 | 17672135 | YQXC_BACSU | Bacillus subtilis Uncharacterized protein yqxC | Swiss-Prot |
| Scaffold_8 | 17670000 | 17679999 | yellow | ppa024775m | 17677320 | 17679704 | AT4G12840.2 | Arabidopsis thaliana unknown protein | TAIR |
| Scaffold_8 | 18160000 | 18169999 | yellow | ppa011864m | 18168960 | 18170796 | AT3G25120.1 | Arabidopsis thaliana mitochondrial import inner membrane translocase subunit Tim17/Tim22/Tim23 family protein | TAIR |
| Scaffold_8 | 18690000 | 18699999 | yellow | ppa000220m | 18697531 | 18707191 | CLAP1_XENLA | Xenopus laevis (African clawed frog) CLIP-associating protein 1 | Swiss-Prot |
| Scaffold_8 | 18740000 | 18749999 | yellow | ppa014958m | 18749472 | 18751705 | GSTX1_TOBAC | Nicotiana tabacum (Common tobacco) Probable glutathione S-transferase | Swiss-Prot |
| Scaffold_rest | 4350000 | 4359999 | yellow | ppa019177m | 329870 | 331013 | NDK1_PEA | Pisum sativum (Garden pea) Nucleoside diphosphate kinase 1 | Swiss-Prot |
| Scaffold_rest | 5950000 | 5959999 | yellow | ppa026195m | 8744 | 10243 | PPME1_ARATH | Arabidopsis thaliana (Mouse-ear cress) Pectinesterase PPME1 | Swiss-Prot |
| Scaffold_rest | 6050000 | 6059999 | yellow | ppa020722m | 5627 | 8093 | TT12_ARATH | Arabidopsis thaliana (Mouse-ear cress) Protein TRANSPARENT TESTA 12 | Swiss-Prot |
| Scaffold_rest | 6760000 | 6769999 | yellow | ppa025424m | 322 | 3863 | A5AEN7_VITVI | Vitis vinifera (Grape) Putative uncharacterized protein | TrEMBL |

**Supplementary Table 9b**. The genes and their function annotation in the regions under edible selection.

| Scaffold | Region under selection | | Branches | Gene name/ID | mRNA region | | synonymous gene ID | Function Annotation | Database |
| --- | --- | --- | --- | --- | --- | --- | --- | --- | --- |
|  | start point | end point |  |  | start point | end point |  |  |  |
| Scaffold_1 | 4850000 | 4859999 | blue | ppa011951m | 4857173 | 4858812 | TRXX_ARATH | Arabidopsis thaliana (Mouse-ear cress) "Thioredoxin-X, chloroplastic" | Swiss-Prot |
| Scaffold_1 | 4850000 | 4859999 | dblue | ppa011951m | 4857173 | 4858812 | TRXX_ARATH | Arabidopsis thaliana (Mouse-ear cress) "Thioredoxin-X, chloroplastic" | Swiss-Prot |
| Scaffold_1 | 4850000 | 4859999 | green | ppa011951m | 4857173 | 4858812 | TRXX_ARATH | Arabidopsis thaliana (Mouse-ear cress) "Thioredoxin-X, chloroplastic" | Swiss-Prot |
| Scaffold_1 | 4850000 | 4859999 | lgreen | ppa011951m | 4857173 | 4858812 | TRXX_ARATH | Arabidopsis thaliana (Mouse-ear cress) "Thioredoxin-X, chloroplastic" | Swiss-Prot |
| Scaffold_1 | 7830000 | 7839999 | blue | ppa000312m | 7830411 | 7836055 | MDR3_HUMAN | Homo sapiens (Human) Multidrug resistance protein 3 | Swiss-Prot |
| Scaffold_1 | 7830000 | 7839999 | dblue | ppa000312m | 7830411 | 7836055 | MDR3_HUMAN | Homo sapiens (Human) Multidrug resistance protein 3 | Swiss-Prot |
| Scaffold_1 | 7830000 | 7839999 | green | ppa000312m | 7830411 | 7836055 | MDR3_HUMAN | Homo sapiens (Human) Multidrug resistance protein 3 | Swiss-Prot |
| Scaffold_1 | 7830000 | 7839999 | lgreen | ppa000312m | 7830411 | 7836055 | MDR3_HUMAN | Homo sapiens (Human) Multidrug resistance protein 3 | Swiss-Prot |
| Scaffold_1 | 7830000 | 7839999 | purple | ppa000312m | 7830411 | 7836055 | MDR3_HUMAN | Homo sapiens (Human) Multidrug resistance protein 3 | Swiss-Prot |
| Scaffold_1 | 8110000 | 8119999 | blue | ppa023381m | 8117825 | 8125610 | PDR3_TOBAC | Nicotiana tabacum (Common tobacco) Pleiotropic drug resistance protein 3 | Swiss-Prot |
| Scaffold_1 | 8110000 | 8119999 | dblue | ppa023381m | 8117825 | 8125610 | PDR3_TOBAC | Nicotiana tabacum (Common tobacco) Pleiotropic drug resistance protein 3 | Swiss-Prot |
| Scaffold_1 | 8110000 | 8119999 | green | ppa023381m | 8117825 | 8125610 | PDR3_TOBAC | Nicotiana tabacum (Common tobacco) Pleiotropic drug resistance protein 3 | Swiss-Prot |
| Scaffold_1 | 8110000 | 8119999 | lgreen | ppa023381m | 8117825 | 8125610 | PDR3_TOBAC | Nicotiana tabacum (Common tobacco) Pleiotropic drug resistance protein 3 | Swiss-Prot |
| Scaffold_1 | 8110000 | 8119999 | purple | ppa023381m | 8117825 | 8125610 | PDR3_TOBAC | Nicotiana tabacum (Common tobacco) Pleiotropic drug resistance protein 3 | Swiss-Prot |
| Scaffold_1 | 9360000 | 9369999 | blue | ppa008676m | 9365387 | 9367830 | HYES_HUMAN | Homo sapiens (Human) Epoxide hydrolase 2 | Swiss-Prot |
| Scaffold_1 | 9360000 | 9369999 | dblue | ppa008676m | 9365387 | 9367830 | HYES_HUMAN | Homo sapiens (Human) Epoxide hydrolase 2 | Swiss-Prot |
| Scaffold_1 | 9360000 | 9369999 | green | ppa008676m | 9365387 | 9367830 | HYES_HUMAN | Homo sapiens (Human) Epoxide hydrolase 2 | Swiss-Prot |
| Scaffold_1 | 9360000 | 9369999 | lgreen | ppa008676m | 9365387 | 9367830 | HYES_HUMAN | Homo sapiens (Human) Epoxide hydrolase 2 | Swiss-Prot |
| Scaffold_1 | 9360000 | 9369999 | purple | ppa008676m | 9365387 | 9367830 | HYES_HUMAN | Homo sapiens (Human) Epoxide hydrolase 2 | Swiss-Prot |
| Scaffold_1 | 24620000 | 24629999 | blue | ppa004343m | 24625651 | 24627353 | C71BX_ARATH | Arabidopsis thaliana (Mouse-ear cress) Cytochrome P450 71B36 | Swiss-Prot |
| Scaffold_1 | 24620000 | 24629999 | dblue | ppa004343m | 24625651 | 24627353 | C71BX_ARATH | Arabidopsis thaliana (Mouse-ear cress) Cytochrome P450 71B36 | Swiss-Prot |
| Scaffold_1 | 24620000 | 24629999 | green | ppa004343m | 24625651 | 24627353 | C71BX_ARATH | Arabidopsis thaliana (Mouse-ear cress) Cytochrome P450 71B36 | Swiss-Prot |
| Scaffold_1 | 24620000 | 24629999 | lgreen | ppa004343m | 24625651 | 24627353 | C71BX_ARATH | Arabidopsis thaliana (Mouse-ear cress) Cytochrome P450 71B36 | Swiss-Prot |
| Scaffold_1 | 24620000 | 24629999 | purple | ppa004343m | 24625651 | 24627353 | C71BX_ARATH | Arabidopsis thaliana (Mouse-ear cress) Cytochrome P450 71B36 | Swiss-Prot |
| Scaffold_1 | 27860000 | 27869999 | blue | ppa010073m | 27869871 | 27873321 | 6PGL1_ARATH | Arabidopsis thaliana (Mouse-ear cress) Probable 6-phosphogluconolactonase 1 | Swiss-Prot |
| Scaffold_1 | 27860000 | 27869999 | dblue | ppa010073m | 27869871 | 27873321 | 6PGL1_ARATH | Arabidopsis thaliana (Mouse-ear cress) Probable 6-phosphogluconolactonase 1 | Swiss-Prot |
| Scaffold_1 | 27860000 | 27869999 | green | ppa010073m | 27869871 | 27873321 | 6PGL1_ARATH | Arabidopsis thaliana (Mouse-ear cress) Probable 6-phosphogluconolactonase 1 | Swiss-Prot |
| Scaffold_1 | 27860000 | 27869999 | lgreen | ppa010073m | 27869871 | 27873321 | 6PGL1_ARATH | Arabidopsis thaliana (Mouse-ear cress) Probable 6-phosphogluconolactonase 1 | Swiss-Prot |
| Scaffold_1 | 27860000 | 27869999 | purple | ppa010073m | 27869871 | 27873321 | 6PGL1_ARATH | Arabidopsis thaliana (Mouse-ear cress) Probable 6-phosphogluconolactonase 1 | Swiss-Prot |
| Scaffold_1 | 29100000 | 29109999 | blue | ppa025892m | 29104089 | 29109590 | ZMYM5_BOVIN | Bos taurus (Bovine) Zinc finger MYM-type protein 5 | Swiss-Prot |
| Scaffold_1 | 29100000 | 29109999 | dblue | ppa025892m | 29104089 | 29109590 | ZMYM5_BOVIN | Bos taurus (Bovine) Zinc finger MYM-type protein 5 | Swiss-Prot |
| Scaffold_1 | 29100000 | 29109999 | green | ppa025892m | 29104089 | 29109590 | ZMYM5_BOVIN | Bos taurus (Bovine) Zinc finger MYM-type protein 5 | Swiss-Prot |
| Scaffold_1 | 29100000 | 29109999 | lgreen | ppa025892m | 29104089 | 29109590 | ZMYM5_BOVIN | Bos taurus (Bovine) Zinc finger MYM-type protein 5 | Swiss-Prot |
| Scaffold_1 | 29100000 | 29109999 | purple | ppa025892m | 29104089 | 29109590 | ZMYM5_BOVIN | Bos taurus (Bovine) Zinc finger MYM-type protein 5 | Swiss-Prot |
| Scaffold_1 | 29110000 | 29119999 | blue | ppa004666m | 29117214 | 29121203 | PTR5_ARATH | Arabidopsis thaliana (Mouse-ear cress) Peptide transporter PTR5 | Swiss-Prot |
| Scaffold_1 | 29110000 | 29119999 | dblue | ppa004666m | 29117214 | 29121203 | PTR5_ARATH | Arabidopsis thaliana (Mouse-ear cress) Peptide transporter PTR5 | Swiss-Prot |
| Scaffold_1 | 29110000 | 29119999 | green | ppa004666m | 29117214 | 29121203 | PTR5_ARATH | Arabidopsis thaliana (Mouse-ear cress) Peptide transporter PTR5 | Swiss-Prot |
| Scaffold_1 | 29110000 | 29119999 | lgreen | ppa004666m | 29117214 | 29121203 | PTR5_ARATH | Arabidopsis thaliana (Mouse-ear cress) Peptide transporter PTR5 | Swiss-Prot |
| Scaffold_1 | 29180000 | 29189999 | blue | ppa008514m | 29188725 | 29190132 | EF118_ARATH | Arabidopsis thaliana (Mouse-ear cress) Ethylene-responsive transcription factor ERF118 | Swiss-Prot |
| Scaffold_1 | 29180000 | 29189999 | dblue | ppa008514m | 29188725 | 29190132 | EF118_ARATH | Arabidopsis thaliana (Mouse-ear cress) Ethylene-responsive transcription factor ERF118 | Swiss-Prot |
| Scaffold_1 | 29180000 | 29189999 | green | ppa008514m | 29188725 | 29190132 | EF118_ARATH | Arabidopsis thaliana (Mouse-ear cress) Ethylene-responsive transcription factor ERF118 | Swiss-Prot |
| Scaffold_1 | 29180000 | 29189999 | lgreen | ppa008514m | 29188725 | 29190132 | EF118_ARATH | Arabidopsis thaliana (Mouse-ear cress) Ethylene-responsive transcription factor ERF118 | Swiss-Prot |
| Scaffold_1 | 29190000 | 29199999 | blue | ppa008777m | 29194751 | 29196273 | DFRA_MALDO | Malus domestica (Apple) (Pyrus malus) Bifunctional dihydroflavonol 4-reductase/flavanone 4-reductase | Swiss-Prot |
| Scaffold_1 | 29190000 | 29199999 | dblue | ppa008777m | 29194751 | 29196273 | DFRA_MALDO | Malus domestica (Apple) (Pyrus malus) Bifunctional dihydroflavonol 4-reductase/flavanone 4-reductase | Swiss-Prot |
| Scaffold_1 | 29190000 | 29199999 | green | ppa008777m | 29194751 | 29196273 | DFRA_MALDO | Malus domestica (Apple) (Pyrus malus) Bifunctional dihydroflavonol 4-reductase/flavanone 4-reductase | Swiss-Prot |
| Scaffold_1 | 29190000 | 29199999 | lgreen | ppa008777m | 29194751 | 29196273 | DFRA_MALDO | Malus domestica (Apple) (Pyrus malus) Bifunctional dihydroflavonol 4-reductase/flavanone 4-reductase | Swiss-Prot |
| Scaffold_1 | 31060000 | 31069999 | blue | ppa002079m | 31060102 | 31065329 | PI5K8_ARATH | Arabidopsis thaliana (Mouse-ear cress) Phosphatidylinositol-4-phosphate 5-kinase 8 | Swiss-Prot |
| Scaffold_1 | 31060000 | 31069999 | dblue | ppa002079m | 31060102 | 31065329 | PI5K8_ARATH | Arabidopsis thaliana (Mouse-ear cress) Phosphatidylinositol-4-phosphate 5-kinase 8 | Swiss-Prot |
| Scaffold_1 | 31060000 | 31069999 | green | ppa002079m | 31060102 | 31065329 | PI5K8_ARATH | Arabidopsis thaliana (Mouse-ear cress) Phosphatidylinositol-4-phosphate 5-kinase 8 | Swiss-Prot |
| Scaffold_1 | 31060000 | 31069999 | lgreen | ppa002079m | 31060102 | 31065329 | PI5K8_ARATH | Arabidopsis thaliana (Mouse-ear cress) Phosphatidylinositol-4-phosphate 5-kinase 8 | Swiss-Prot |
| Scaffold_1 | 31060000 | 31069999 | purple | ppa002079m | 31060102 | 31065329 | PI5K8_ARATH | Arabidopsis thaliana (Mouse-ear cress) Phosphatidylinositol-4-phosphate 5-kinase 8 | Swiss-Prot |
| Scaffold_1 | 36220000 | 36229999 | blue | ppa010039m | 36228199 | 36230042 | CB24_PEA | Pisum sativum (Garden pea) "Chlorophyll a-b binding protein P4, chloroplastic" | Swiss-Prot |
| Scaffold_1 | 36220000 | 36229999 | dblue | ppa010039m | 36228199 | 36230042 | CB24_PEA | Pisum sativum (Garden pea) "Chlorophyll a-b binding protein P4, chloroplastic" | Swiss-Prot |
| Scaffold_1 | 36220000 | 36229999 | green | ppa010039m | 36228199 | 36230042 | CB24_PEA | Pisum sativum (Garden pea) "Chlorophyll a-b binding protein P4, chloroplastic" | Swiss-Prot |
| Scaffold_1 | 36220000 | 36229999 | lgreen | ppa010039m | 36228199 | 36230042 | CB24_PEA | Pisum sativum (Garden pea) "Chlorophyll a-b binding protein P4, chloroplastic" | Swiss-Prot |
| Scaffold_1 | 36220000 | 36229999 | purple | ppa010039m | 36228199 | 36230042 | CB24_PEA | Pisum sativum (Garden pea) "Chlorophyll a-b binding protein P4, chloroplastic" | Swiss-Prot |
| Scaffold_1 | 37660000 | 37669999 | blue | ppa006900m | 37668489 | 37672355 | Y2433_ARATH | Arabidopsis thaliana (Mouse-ear cress) Uncharacterized protein At2g24330 | Swiss-Prot |
| Scaffold_1 | 37660000 | 37669999 | dblue | ppa006900m | 37668489 | 37672355 | Y2433_ARATH | Arabidopsis thaliana (Mouse-ear cress) Uncharacterized protein At2g24330 | Swiss-Prot |
| Scaffold_1 | 37660000 | 37669999 | green | ppa006900m | 37668489 | 37672355 | Y2433_ARATH | Arabidopsis thaliana (Mouse-ear cress) Uncharacterized protein At2g24330 | Swiss-Prot |
| Scaffold_1 | 37660000 | 37669999 | lgreen | ppa006900m | 37668489 | 37672355 | Y2433_ARATH | Arabidopsis thaliana (Mouse-ear cress) Uncharacterized protein At2g24330 | Swiss-Prot |
| Scaffold_1 | 37660000 | 37669999 | purple | ppa006900m | 37668489 | 37672355 | Y2433_ARATH | Arabidopsis thaliana (Mouse-ear cress) Uncharacterized protein At2g24330 | Swiss-Prot |
| Scaffold_1 | 38380000 | 38389999 | blue | ppa020390m | 38389458 | 38393842 | CRK10_ARATH | Arabidopsis thaliana (Mouse-ear cress) Cysteine-rich receptor-like protein kinase 10 | Swiss-Prot |
| Scaffold_1 | 38380000 | 38389999 | dblue | ppa020390m | 38389458 | 38393842 | CRK10_ARATH | Arabidopsis thaliana (Mouse-ear cress) Cysteine-rich receptor-like protein kinase 10 | Swiss-Prot |
| Scaffold_1 | 38380000 | 38389999 | green | ppa020390m | 38389458 | 38393842 | CRK10_ARATH | Arabidopsis thaliana (Mouse-ear cress) Cysteine-rich receptor-like protein kinase 10 | Swiss-Prot |
| Scaffold_1 | 38380000 | 38389999 | lgreen | ppa020390m | 38389458 | 38393842 | CRK10_ARATH | Arabidopsis thaliana (Mouse-ear cress) Cysteine-rich receptor-like protein kinase 10 | Swiss-Prot |
| Scaffold_1 | 38380000 | 38389999 | purple | ppa020390m | 38389458 | 38393842 | CRK10_ARATH | Arabidopsis thaliana (Mouse-ear cress) Cysteine-rich receptor-like protein kinase 10 | Swiss-Prot |
| Scaffold_1 | 38480000 | 38489999 | blue | ppa024353m | 38489931 | 38492114 | AT1G64260.1 | Arabidopsis thaliana zinc finger protein-related | TAIR |
| Scaffold_1 | 38480000 | 38489999 | dblue | ppa024353m | 38489931 | 38492114 | AT1G64260.1 | Arabidopsis thaliana zinc finger protein-related | TAIR |
| Scaffold_1 | 38480000 | 38489999 | green | ppa024353m | 38489931 | 38492114 | AT1G64260.1 | Arabidopsis thaliana zinc finger protein-related | TAIR |
| Scaffold_1 | 38480000 | 38489999 | lgreen | ppa024353m | 38489931 | 38492114 | AT1G64260.1 | Arabidopsis thaliana zinc finger protein-related | TAIR |
| Scaffold_1 | 38540000 | 38549999 | blue | ppa011523m | 38545200 | 38546068 | RD22_ARATH | Arabidopsis thaliana (Mouse-ear cress) Dehydration-responsive protein RD22 | Swiss-Prot |
| Scaffold_1 | 38540000 | 38549999 | dblue | ppa011523m | 38545200 | 38546068 | RD22_ARATH | Arabidopsis thaliana (Mouse-ear cress) Dehydration-responsive protein RD22 | Swiss-Prot |
| Scaffold_1 | 38540000 | 38549999 | green | ppa011523m | 38545200 | 38546068 | RD22_ARATH | Arabidopsis thaliana (Mouse-ear cress) Dehydration-responsive protein RD22 | Swiss-Prot |
| Scaffold_1 | 38540000 | 38549999 | lgreen | ppa011523m | 38545200 | 38546068 | RD22_ARATH | Arabidopsis thaliana (Mouse-ear cress) Dehydration-responsive protein RD22 | Swiss-Prot |
| Scaffold_1 | 38540000 | 38549999 | purple | ppa011523m | 38545200 | 38546068 | RD22_ARATH | Arabidopsis thaliana (Mouse-ear cress) Dehydration-responsive protein RD22 | Swiss-Prot |
| Scaffold_1 | 38650000 | 38659999 | blue | ppa025730m | 38656222 | 38657679 | B9NFQ4_POPTR | Populus trichocarpa (Western balsam poplar) (Populus balsamifera subsp. trichocarpa) Predicted protein | TrEMBL |
| Scaffold_1 | 38650000 | 38659999 | dblue | ppa025730m | 38656222 | 38657679 | B9NFQ4_POPTR | Populus trichocarpa (Western balsam poplar) (Populus balsamifera subsp. trichocarpa) Predicted protein | TrEMBL |
| Scaffold_1 | 38650000 | 38659999 | green | ppa025730m | 38656222 | 38657679 | B9NFQ4_POPTR | Populus trichocarpa (Western balsam poplar) (Populus balsamifera subsp. trichocarpa) Predicted protein | TrEMBL |
| Scaffold_1 | 38650000 | 38659999 | lgreen | ppa025730m | 38656222 | 38657679 | B9NFQ4_POPTR | Populus trichocarpa (Western balsam poplar) (Populus balsamifera subsp. trichocarpa) Predicted protein | TrEMBL |
| Scaffold_1 | 38920000 | 38929999 | blue | ppa015722m | 38921541 | 38922718 | Y3720_ARATH | Arabidopsis thaliana (Mouse-ear cress) UPF0481 protein At3g47200 | Swiss-Prot |
| Scaffold_1 | 38920000 | 38929999 | dblue | ppa015722m | 38921541 | 38922718 | Y3720_ARATH | Arabidopsis thaliana (Mouse-ear cress) UPF0481 protein At3g47200 | Swiss-Prot |
| Scaffold_1 | 38920000 | 38929999 | green | ppa015722m | 38921541 | 38922718 | Y3720_ARATH | Arabidopsis thaliana (Mouse-ear cress) UPF0481 protein At3g47200 | Swiss-Prot |
| Scaffold_1 | 38920000 | 38929999 | lgreen | ppa015722m | 38921541 | 38922718 | Y3720_ARATH | Arabidopsis thaliana (Mouse-ear cress) UPF0481 protein At3g47200 | Swiss-Prot |
| Scaffold_1 | 38920000 | 38929999 | purple | ppa015722m | 38921541 | 38922718 | Y3720_ARATH | Arabidopsis thaliana (Mouse-ear cress) UPF0481 protein At3g47200 | Swiss-Prot |
| Scaffold_1 | 39000000 | 39009999 | blue | ppa023243m | 39007925 | 39009219 | Y3720_ARATH | Arabidopsis thaliana (Mouse-ear cress) UPF0481 protein At3g47200 | Swiss-Prot |
| Scaffold_1 | 39000000 | 39009999 | dblue | ppa023243m | 39007925 | 39009219 | Y3720_ARATH | Arabidopsis thaliana (Mouse-ear cress) UPF0481 protein At3g47200 | Swiss-Prot |
| Scaffold_1 | 39000000 | 39009999 | green | ppa023243m | 39007925 | 39009219 | Y3720_ARATH | Arabidopsis thaliana (Mouse-ear cress) UPF0481 protein At3g47200 | Swiss-Prot |
| Scaffold_1 | 39000000 | 39009999 | lgreen | ppa023243m | 39007925 | 39009219 | Y3720_ARATH | Arabidopsis thaliana (Mouse-ear cress) UPF0481 protein At3g47200 | Swiss-Prot |
| Scaffold_1 | 39000000 | 39009999 | purple | ppa023243m | 39007925 | 39009219 | Y3720_ARATH | Arabidopsis thaliana (Mouse-ear cress) UPF0481 protein At3g47200 | Swiss-Prot |
| Scaffold_1 | 39120000 | 39129999 | blue | ppa025007m | 39128238 | 39131015 | PGLR3_ARATH | Arabidopsis thaliana (Mouse-ear cress) Probable polygalacturonase At3g15720 | Swiss-Prot |
| Scaffold_1 | 39120000 | 39129999 | dblue | ppa025007m | 39128238 | 39131015 | PGLR3_ARATH | Arabidopsis thaliana (Mouse-ear cress) Probable polygalacturonase At3g15720 | Swiss-Prot |
| Scaffold_1 | 39120000 | 39129999 | green | ppa025007m | 39128238 | 39131015 | PGLR3_ARATH | Arabidopsis thaliana (Mouse-ear cress) Probable polygalacturonase At3g15720 | Swiss-Prot |
| Scaffold_1 | 39120000 | 39129999 | lgreen | ppa025007m | 39128238 | 39131015 | PGLR3_ARATH | Arabidopsis thaliana (Mouse-ear cress) Probable polygalacturonase At3g15720 | Swiss-Prot |
| Scaffold_1 | 40750000 | 40759999 | blue | ppa005780m | 40759914 | 40762567 | Y1567_ARATH | Arabidopsis thaliana (Mouse-ear cress) Uncharacterized UDP-glucosyltransferase At1g05670 | Swiss-Prot |
| Scaffold_1 | 40750000 | 40759999 | dblue | ppa005780m | 40759914 | 40762567 | Y1567_ARATH | Arabidopsis thaliana (Mouse-ear cress) Uncharacterized UDP-glucosyltransferase At1g05670 | Swiss-Prot |
| Scaffold_1 | 40750000 | 40759999 | green | ppa005780m | 40759914 | 40762567 | Y1567_ARATH | Arabidopsis thaliana (Mouse-ear cress) Uncharacterized UDP-glucosyltransferase At1g05670 | Swiss-Prot |
| Scaffold_1 | 40750000 | 40759999 | lgreen | ppa005780m | 40759914 | 40762567 | Y1567_ARATH | Arabidopsis thaliana (Mouse-ear cress) Uncharacterized UDP-glucosyltransferase At1g05670 | Swiss-Prot |
| Scaffold_1 | 40940000 | 40949999 | blue | ppa007593m | 40943437 | 40945713 | TGA7_ARATH | Arabidopsis thaliana (Mouse-ear cress) Transcription factor TGA7 | Swiss-Prot |
| Scaffold_1 | 40940000 | 40949999 | dblue | ppa007593m | 40943437 | 40945713 | TGA7_ARATH | Arabidopsis thaliana (Mouse-ear cress) Transcription factor TGA7 | Swiss-Prot |
| Scaffold_1 | 40940000 | 40949999 | green | ppa007593m | 40943437 | 40945713 | TGA7_ARATH | Arabidopsis thaliana (Mouse-ear cress) Transcription factor TGA7 | Swiss-Prot |
| Scaffold_1 | 40940000 | 40949999 | lgreen | ppa007593m | 40943437 | 40945713 | TGA7_ARATH | Arabidopsis thaliana (Mouse-ear cress) Transcription factor TGA7 | Swiss-Prot |
| Scaffold_1 | 41090000 | 41099999 | blue | ppa017599m | 41097280 | 41100674 | Y4885_ARATH | Arabidopsis thaliana (Mouse-ear cress) Probable LRR receptor-like serine/threonine-protein kinase At4g08850 | Swiss-Prot |
| Scaffold_1 | 41090000 | 41099999 | dblue | ppa017599m | 41097280 | 41100674 | Y4885_ARATH | Arabidopsis thaliana (Mouse-ear cress) Probable LRR receptor-like serine/threonine-protein kinase At4g08850 | Swiss-Prot |
| Scaffold_1 | 41090000 | 41099999 | green | ppa017599m | 41097280 | 41100674 | Y4885_ARATH | Arabidopsis thaliana (Mouse-ear cress) Probable LRR receptor-like serine/threonine-protein kinase At4g08850 | Swiss-Prot |
| Scaffold_1 | 41090000 | 41099999 | lgreen | ppa017599m | 41097280 | 41100674 | Y4885_ARATH | Arabidopsis thaliana (Mouse-ear cress) Probable LRR receptor-like serine/threonine-protein kinase At4g08850 | Swiss-Prot |
| Scaffold_1 | 41090000 | 41099999 | purple | ppa017599m | 41097280 | 41100674 | Y4885_ARATH | Arabidopsis thaliana (Mouse-ear cress) Probable LRR receptor-like serine/threonine-protein kinase At4g08850 | Swiss-Prot |
| Scaffold_1 | 41190000 | 41199999 | blue | ppa012701m | 41197383 | 41198682 | AT1G78020.1 | Arabidopsis thaliana senescence-associated protein-related | TAIR |
| Scaffold_1 | 41190000 | 41199999 | dblue | ppa012701m | 41197383 | 41198682 | AT1G78020.1 | Arabidopsis thaliana senescence-associated protein-related | TAIR |
| Scaffold_1 | 41190000 | 41199999 | green | ppa012701m | 41197383 | 41198682 | AT1G78020.1 | Arabidopsis thaliana senescence-associated protein-related | TAIR |
| Scaffold_1 | 41190000 | 41199999 | lgreen | ppa012701m | 41197383 | 41198682 | AT1G78020.1 | Arabidopsis thaliana senescence-associated protein-related | TAIR |
| Scaffold_1 | 41500000 | 41509999 | blue | ppa023945m | 41504516 | 41509744 | CCR4A_ARATH | Arabidopsis thaliana (Mouse-ear cress) Carbon catabolite repressor protein 4 homolog 1 | Swiss-Prot |
| Scaffold_1 | 41500000 | 41509999 | dblue | ppa023945m | 41504516 | 41509744 | CCR4A_ARATH | Arabidopsis thaliana (Mouse-ear cress) Carbon catabolite repressor protein 4 homolog 1 | Swiss-Prot |
| Scaffold_1 | 41500000 | 41509999 | green | ppa023945m | 41504516 | 41509744 | CCR4A_ARATH | Arabidopsis thaliana (Mouse-ear cress) Carbon catabolite repressor protein 4 homolog 1 | Swiss-Prot |
| Scaffold_1 | 41500000 | 41509999 | lgreen | ppa023945m | 41504516 | 41509744 | CCR4A_ARATH | Arabidopsis thaliana (Mouse-ear cress) Carbon catabolite repressor protein 4 homolog 1 | Swiss-Prot |
| Scaffold_1 | 41500000 | 41509999 | purple | ppa023945m | 41504516 | 41509744 | CCR4A_ARATH | Arabidopsis thaliana (Mouse-ear cress) Carbon catabolite repressor protein 4 homolog 1 | Swiss-Prot |
| Scaffold_1 | 41510000 | 41519999 | blue | ppa019174m | 41516293 | 41517272 | EXPA9_ARATH | Arabidopsis thaliana (Mouse-ear cress) Expansin-A9 | Swiss-Prot |
| Scaffold_1 | 41510000 | 41519999 | dblue | ppa019174m | 41516293 | 41517272 | EXPA9_ARATH | Arabidopsis thaliana (Mouse-ear cress) Expansin-A9 | Swiss-Prot |
| Scaffold_1 | 41510000 | 41519999 | green | ppa019174m | 41516293 | 41517272 | EXPA9_ARATH | Arabidopsis thaliana (Mouse-ear cress) Expansin-A9 | Swiss-Prot |
| Scaffold_1 | 41510000 | 41519999 | lgreen | ppa019174m | 41516293 | 41517272 | EXPA9_ARATH | Arabidopsis thaliana (Mouse-ear cress) Expansin-A9 | Swiss-Prot |
| Scaffold_1 | 41510000 | 41519999 | purple | ppa019174m | 41516293 | 41517272 | EXPA9_ARATH | Arabidopsis thaliana (Mouse-ear cress) Expansin-A9 | Swiss-Prot |
| Scaffold_1 | 41530000 | 41539999 | blue | ppa004853m | 41537562 | 41541751 | AT1G34630.1 | Arabidopsis thaliana unknown protein | TAIR |
| Scaffold_1 | 41530000 | 41539999 | dblue | ppa004853m | 41537562 | 41541751 | AT1G34630.1 | Arabidopsis thaliana unknown protein | TAIR |
| Scaffold_1 | 41530000 | 41539999 | green | ppa004853m | 41537562 | 41541751 | AT1G34630.1 | Arabidopsis thaliana unknown protein | TAIR |
| Scaffold_1 | 41530000 | 41539999 | lgreen | ppa004853m | 41537562 | 41541751 | AT1G34630.1 | Arabidopsis thaliana unknown protein | TAIR |
| Scaffold_1 | 41550000 | 41559999 | blue | ppa026006m | 41557908 | 41559331 | MYB39_ARATH | Arabidopsis thaliana (Mouse-ear cress) Transcription factor MYB39 | Swiss-Prot |
| Scaffold_1 | 41550000 | 41559999 | dblue | ppa026006m | 41557908 | 41559331 | MYB39_ARATH | Arabidopsis thaliana (Mouse-ear cress) Transcription factor MYB39 | Swiss-Prot |
| Scaffold_1 | 41550000 | 41559999 | green | ppa026006m | 41557908 | 41559331 | MYB39_ARATH | Arabidopsis thaliana (Mouse-ear cress) Transcription factor MYB39 | Swiss-Prot |
| Scaffold_1 | 41550000 | 41559999 | lgreen | ppa026006m | 41557908 | 41559331 | MYB39_ARATH | Arabidopsis thaliana (Mouse-ear cress) Transcription factor MYB39 | Swiss-Prot |
| Scaffold_1 | 41550000 | 41559999 | purple | ppa026006m | 41557908 | 41559331 | MYB39_ARATH | Arabidopsis thaliana (Mouse-ear cress) Transcription factor MYB39 | Swiss-Prot |
| Scaffold_1 | 41700000 | 41709999 | blue | ppa004956m | 41709197 | 41710824 | COGT2_ARATH | Arabidopsis thaliana (Mouse-ear cress) Cytokinin-O-glucosyltransferase 2 | Swiss-Prot |
| Scaffold_1 | 41700000 | 41709999 | dblue | ppa004956m | 41709197 | 41710824 | COGT2_ARATH | Arabidopsis thaliana (Mouse-ear cress) Cytokinin-O-glucosyltransferase 2 | Swiss-Prot |
| Scaffold_1 | 41700000 | 41709999 | green | ppa004956m | 41709197 | 41710824 | COGT2_ARATH | Arabidopsis thaliana (Mouse-ear cress) Cytokinin-O-glucosyltransferase 2 | Swiss-Prot |
| Scaffold_1 | 41700000 | 41709999 | lgreen | ppa004956m | 41709197 | 41710824 | COGT2_ARATH | Arabidopsis thaliana (Mouse-ear cress) Cytokinin-O-glucosyltransferase 2 | Swiss-Prot |
| Scaffold_1 | 41710000 | 41719999 | blue | ppa005043m | 41718603 | 41720233 | COGT2_ARATH | Arabidopsis thaliana (Mouse-ear cress) Cytokinin-O-glucosyltransferase 2 | Swiss-Prot |
| Scaffold_1 | 41710000 | 41719999 | dblue | ppa005043m | 41718603 | 41720233 | COGT2_ARATH | Arabidopsis thaliana (Mouse-ear cress) Cytokinin-O-glucosyltransferase 2 | Swiss-Prot |
| Scaffold_1 | 41710000 | 41719999 | green | ppa005043m | 41718603 | 41720233 | COGT2_ARATH | Arabidopsis thaliana (Mouse-ear cress) Cytokinin-O-glucosyltransferase 2 | Swiss-Prot |
| Scaffold_1 | 41710000 | 41719999 | lgreen | ppa005043m | 41718603 | 41720233 | COGT2_ARATH | Arabidopsis thaliana (Mouse-ear cress) Cytokinin-O-glucosyltransferase 2 | Swiss-Prot |
| Scaffold_1 | 41720000 | 41729999 | blue | ppa023851m | 41725189 | 41726819 | COGT2_ARATH | Arabidopsis thaliana (Mouse-ear cress) Cytokinin-O-glucosyltransferase 2 | Swiss-Prot |
| Scaffold_1 | 41720000 | 41729999 | dblue | ppa023851m | 41725189 | 41726819 | COGT2_ARATH | Arabidopsis thaliana (Mouse-ear cress) Cytokinin-O-glucosyltransferase 2 | Swiss-Prot |
| Scaffold_1 | 41720000 | 41729999 | green | ppa023851m | 41725189 | 41726819 | COGT2_ARATH | Arabidopsis thaliana (Mouse-ear cress) Cytokinin-O-glucosyltransferase 2 | Swiss-Prot |
| Scaffold_1 | 41720000 | 41729999 | lgreen | ppa023851m | 41725189 | 41726819 | COGT2_ARATH | Arabidopsis thaliana (Mouse-ear cress) Cytokinin-O-glucosyltransferase 2 | Swiss-Prot |
| Scaffold_1 | 45070000 | 45079999 | blue | ppa013136m | 45077348 | 45079614 | AT1G33810.1 | Arabidopsis thaliana unknown protein | TAIR |
| Scaffold_1 | 45070000 | 45079999 | dblue | ppa013136m | 45077348 | 45079614 | AT1G33810.1 | Arabidopsis thaliana unknown protein | TAIR |
| Scaffold_1 | 45070000 | 45079999 | green | ppa013136m | 45077348 | 45079614 | AT1G33810.1 | Arabidopsis thaliana unknown protein | TAIR |
| Scaffold_1 | 45070000 | 45079999 | lgreen | ppa013136m | 45077348 | 45079614 | AT1G33810.1 | Arabidopsis thaliana unknown protein | TAIR |
| Scaffold_1 | 45070000 | 45079999 | purple | ppa013136m | 45077348 | 45079614 | AT1G33810.1 | Arabidopsis thaliana unknown protein | TAIR |
| Scaffold_1 | 45080000 | 45089999 | blue | ppa019161m | 45086795 | 45088675 | PP340_ARATH | Arabidopsis thaliana (Mouse-ear cress) Pentatricopeptide repeat-containing protein At4g28010 | Swiss-Prot |
| Scaffold_1 | 45080000 | 45089999 | dblue | ppa019161m | 45086795 | 45088675 | PP340_ARATH | Arabidopsis thaliana (Mouse-ear cress) Pentatricopeptide repeat-containing protein At4g28010 | Swiss-Prot |
| Scaffold_1 | 45080000 | 45089999 | green | ppa019161m | 45086795 | 45088675 | PP340_ARATH | Arabidopsis thaliana (Mouse-ear cress) Pentatricopeptide repeat-containing protein At4g28010 | Swiss-Prot |
| Scaffold_1 | 45080000 | 45089999 | lgreen | ppa019161m | 45086795 | 45088675 | PP340_ARATH | Arabidopsis thaliana (Mouse-ear cress) Pentatricopeptide repeat-containing protein At4g28010 | Swiss-Prot |
| Scaffold_1 | 45290000 | 45299999 | blue | ppa009375m | 45298329 | 45299769 | D1ID67_VITVI | Vitis vinifera (Grape) "Whole genome shotgun sequence of line PN40024, Scaffold_82.assembly12x" | TrEMBL |
| Scaffold_1 | 45290000 | 45299999 | dblue | ppa009375m | 45298329 | 45299769 | D1ID67_VITVI | Vitis vinifera (Grape) "Whole genome shotgun sequence of line PN40024, Scaffold_82.assembly12x" | TrEMBL |
| Scaffold_1 | 45290000 | 45299999 | green | ppa009375m | 45298329 | 45299769 | D1ID67_VITVI | Vitis vinifera (Grape) "Whole genome shotgun sequence of line PN40024, Scaffold_82.assembly12x" | TrEMBL |
| Scaffold_1 | 45290000 | 45299999 | lgreen | ppa009375m | 45298329 | 45299769 | D1ID67_VITVI | Vitis vinifera (Grape) "Whole genome shotgun sequence of line PN40024, Scaffold_82.assembly12x" | TrEMBL |
| Scaffold_1 | 45290000 | 45299999 | purple | ppa009375m | 45298329 | 45299769 | D1ID67_VITVI | Vitis vinifera (Grape) "Whole genome shotgun sequence of line PN40024, Scaffold_82.assembly12x" | TrEMBL |
| Scaffold_1 | 45980000 | 45989999 | blue | ppa012329m | 45988859 | 45993579 | T2AG_ARATH | Arabidopsis thaliana (Mouse-ear cress) Transcription initiation factor IIA subunit 2 | Swiss-Prot |
| Scaffold_1 | 45980000 | 45989999 | dblue | ppa012329m | 45988859 | 45993579 | T2AG_ARATH | Arabidopsis thaliana (Mouse-ear cress) Transcription initiation factor IIA subunit 2 | Swiss-Prot |
| Scaffold_1 | 45980000 | 45989999 | green | ppa012329m | 45988859 | 45993579 | T2AG_ARATH | Arabidopsis thaliana (Mouse-ear cress) Transcription initiation factor IIA subunit 2 | Swiss-Prot |
| Scaffold_1 | 45980000 | 45989999 | lgreen | ppa012329m | 45988859 | 45993579 | T2AG_ARATH | Arabidopsis thaliana (Mouse-ear cress) Transcription initiation factor IIA subunit 2 | Swiss-Prot |
| Scaffold_1 | 45980000 | 45989999 | purple | ppa012329m | 45988859 | 45993579 | T2AG_ARATH | Arabidopsis thaliana (Mouse-ear cress) Transcription initiation factor IIA subunit 2 | Swiss-Prot |
| Scaffold_1 | 45990000 | 45999999 | blue | ppa000345m | 45994513 | 46007992 | GWD2_ARATH | Arabidopsis thaliana (Mouse-ear cress) Alpha-glucan water dikinase 2 | Swiss-Prot |
| Scaffold_1 | 45990000 | 45999999 | dblue | ppa000345m | 45994513 | 46007992 | GWD2_ARATH | Arabidopsis thaliana (Mouse-ear cress) Alpha-glucan water dikinase 2 | Swiss-Prot |
| Scaffold_1 | 45990000 | 45999999 | green | ppa000345m | 45994513 | 46007992 | GWD2_ARATH | Arabidopsis thaliana (Mouse-ear cress) Alpha-glucan water dikinase 2 | Swiss-Prot |
| Scaffold_1 | 45990000 | 45999999 | lgreen | ppa000345m | 45994513 | 46007992 | GWD2_ARATH | Arabidopsis thaliana (Mouse-ear cress) Alpha-glucan water dikinase 2 | Swiss-Prot |
| Scaffold_1 | 45990000 | 45999999 | purple | ppa000345m | 45994513 | 46007992 | GWD2_ARATH | Arabidopsis thaliana (Mouse-ear cress) Alpha-glucan water dikinase 2 | Swiss-Prot |
| Scaffold_1 | 46050000 | 46059999 | blue | ppa008968m | 46059904 | 46065346 | AT4G24460.1 | Arabidopsis thaliana unknown protein | TAIR |
| Scaffold_1 | 46050000 | 46059999 | dblue | ppa008968m | 46059904 | 46065346 | AT4G24460.1 | Arabidopsis thaliana unknown protein | TAIR |
| Scaffold_1 | 46050000 | 46059999 | green | ppa008968m | 46059904 | 46065346 | AT4G24460.1 | Arabidopsis thaliana unknown protein | TAIR |
| Scaffold_1 | 46050000 | 46059999 | lgreen | ppa008968m | 46059904 | 46065346 | AT4G24460.1 | Arabidopsis thaliana unknown protein | TAIR |
| Scaffold_1 | 46050000 | 46059999 | purple | ppa008968m | 46059904 | 46065346 | AT4G24460.1 | Arabidopsis thaliana unknown protein | TAIR |
| Scaffold_1 | 46510000 | 46519999 | blue | ppa026740m | 46519680 | 46520138 | FB135_ARATH | Arabidopsis thaliana (Mouse-ear cress) F-box protein At3g07870 | Swiss-Prot |
| Scaffold_1 | 46510000 | 46519999 | dblue | ppa026740m | 46519680 | 46520138 | FB135_ARATH | Arabidopsis thaliana (Mouse-ear cress) F-box protein At3g07870 | Swiss-Prot |
| Scaffold_1 | 46510000 | 46519999 | green | ppa026740m | 46519680 | 46520138 | FB135_ARATH | Arabidopsis thaliana (Mouse-ear cress) F-box protein At3g07870 | Swiss-Prot |
| Scaffold_1 | 46510000 | 46519999 | lgreen | ppa026740m | 46519680 | 46520138 | FB135_ARATH | Arabidopsis thaliana (Mouse-ear cress) F-box protein At3g07870 | Swiss-Prot |
| Scaffold_2 | 6000000 | 6009999 | blue | ppa009027m | 6008190 | 6012659 | S6PD_MALDO | Malus domestica (Apple) (Pyrus malus) NADP-dependent D-sorbitol-6-phosphate dehydrogenase | Swiss-Prot |
| Scaffold_2 | 6000000 | 6009999 | dblue | ppa009027m | 6008190 | 6012659 | S6PD_MALDO | Malus domestica (Apple) (Pyrus malus) NADP-dependent D-sorbitol-6-phosphate dehydrogenase | Swiss-Prot |
| Scaffold_2 | 6000000 | 6009999 | green | ppa009027m | 6008190 | 6012659 | S6PD_MALDO | Malus domestica (Apple) (Pyrus malus) NADP-dependent D-sorbitol-6-phosphate dehydrogenase | Swiss-Prot |
| Scaffold_2 | 6000000 | 6009999 | lgreen | ppa009027m | 6008190 | 6012659 | S6PD_MALDO | Malus domestica (Apple) (Pyrus malus) NADP-dependent D-sorbitol-6-phosphate dehydrogenase | Swiss-Prot |
| Scaffold_2 | 6000000 | 6009999 | purple | ppa009027m | 6008190 | 6012659 | S6PD_MALDO | Malus domestica (Apple) (Pyrus malus) NADP-dependent D-sorbitol-6-phosphate dehydrogenase | Swiss-Prot |
| Scaffold_2 | 8130000 | 8139999 | blue | ppa016239m | 8139069 | 8140763 | PYRG2_XENLA | Xenopus laevis (African clawed frog) CTP synthase 2 | Swiss-Prot |
| Scaffold_2 | 8130000 | 8139999 | dblue | ppa016239m | 8139069 | 8140763 | PYRG2_XENLA | Xenopus laevis (African clawed frog) CTP synthase 2 | Swiss-Prot |
| Scaffold_2 | 8130000 | 8139999 | green | ppa016239m | 8139069 | 8140763 | PYRG2_XENLA | Xenopus laevis (African clawed frog) CTP synthase 2 | Swiss-Prot |
| Scaffold_2 | 8130000 | 8139999 | lgreen | ppa016239m | 8139069 | 8140763 | PYRG2_XENLA | Xenopus laevis (African clawed frog) CTP synthase 2 | Swiss-Prot |
| Scaffold_2 | 15760000 | 15769999 | blue | ppa024369m | 15762126 | 15763553 | AT2G45060.1 | Arabidopsis thaliana FUNCTIONS IN: molecular_function unknown; INVOLVED IN: biological_process unknown | TAIR |
| Scaffold_2 | 15760000 | 15769999 | dblue | ppa024369m | 15762126 | 15763553 | AT2G45060.1 | Arabidopsis thaliana FUNCTIONS IN: molecular_function unknown; INVOLVED IN: biological_process unknown | TAIR |
| Scaffold_2 | 15760000 | 15769999 | green | ppa024369m | 15762126 | 15763553 | AT2G45060.1 | Arabidopsis thaliana FUNCTIONS IN: molecular_function unknown; INVOLVED IN: biological_process unknown | TAIR |
| Scaffold_2 | 15760000 | 15769999 | lgreen | ppa024369m | 15762126 | 15763553 | AT2G45060.1 | Arabidopsis thaliana FUNCTIONS IN: molecular_function unknown; INVOLVED IN: biological_process unknown | TAIR |
| Scaffold_2 | 15760000 | 15769999 | purple | ppa024369m | 15762126 | 15763553 | AT2G45060.1 | Arabidopsis thaliana FUNCTIONS IN: molecular_function unknown; INVOLVED IN: biological_process unknown | TAIR |
| Scaffold_2 | 20070000 | 20079999 | blue | ppa024343m | 20075386 | 20078216 | COMT1_POPKI | Populus kitakamiensis (Aspen) Caffeic acid 3-O-methyltransferase 1 | Swiss-Prot |
| Scaffold_2 | 20070000 | 20079999 | dblue | ppa024343m | 20075386 | 20078216 | COMT1_POPKI | Populus kitakamiensis (Aspen) Caffeic acid 3-O-methyltransferase 1 | Swiss-Prot |
| Scaffold_2 | 20070000 | 20079999 | green | ppa024343m | 20075386 | 20078216 | COMT1_POPKI | Populus kitakamiensis (Aspen) Caffeic acid 3-O-methyltransferase 1 | Swiss-Prot |
| Scaffold_2 | 20070000 | 20079999 | lgreen | ppa024343m | 20075386 | 20078216 | COMT1_POPKI | Populus kitakamiensis (Aspen) Caffeic acid 3-O-methyltransferase 1 | Swiss-Prot |
| Scaffold_2 | 20070000 | 20079999 | purple | ppa024343m | 20075386 | 20078216 | COMT1_POPKI | Populus kitakamiensis (Aspen) Caffeic acid 3-O-methyltransferase 1 | Swiss-Prot |
| Scaffold_2 | 24010000 | 24019999 | blue | ppb020951m | 24018529 | 24018974 | N/A | N/A N/A | N/A |
| Scaffold_2 | 24010000 | 24019999 | dblue | ppb020951m | 24018529 | 24018974 | N/A | N/A N/A | N/A |
| Scaffold_2 | 24010000 | 24019999 | green | ppb020951m | 24018529 | 24018974 | N/A | N/A N/A | N/A |
| Scaffold_2 | 24010000 | 24019999 | lgreen | ppb020951m | 24018529 | 24018974 | N/A | N/A N/A | N/A |
| Scaffold_3 | 1620000 | 1629999 | blue | ppa018513m | 1626104 | 1627947 | AT3G63270.1 | Arabidopsis thaliana unknown protein | TAIR |
| Scaffold_3 | 1620000 | 1629999 | dblue | ppa018513m | 1626104 | 1627947 | AT3G63270.1 | Arabidopsis thaliana unknown protein | TAIR |
| Scaffold_3 | 1620000 | 1629999 | green | ppa018513m | 1626104 | 1627947 | AT3G63270.1 | Arabidopsis thaliana unknown protein | TAIR |
| Scaffold_3 | 1620000 | 1629999 | lgreen | ppa018513m | 1626104 | 1627947 | AT3G63270.1 | Arabidopsis thaliana unknown protein | TAIR |
| Scaffold_3 | 1620000 | 1629999 | purple | ppa018513m | 1626104 | 1627947 | AT3G63270.1 | Arabidopsis thaliana unknown protein | TAIR |
| Scaffold_3 | 5310000 | 5319999 | blue | ppa026804m | 5317079 | 5320158 | VRN1_ARATH | Arabidopsis thaliana (Mouse-ear cress) B3 domain-containing transcription factor VRN1 | Swiss-Prot |
| Scaffold_3 | 5310000 | 5319999 | dblue | ppa026804m | 5317079 | 5320158 | VRN1_ARATH | Arabidopsis thaliana (Mouse-ear cress) B3 domain-containing transcription factor VRN1 | Swiss-Prot |
| Scaffold_3 | 5310000 | 5319999 | green | ppa026804m | 5317079 | 5320158 | VRN1_ARATH | Arabidopsis thaliana (Mouse-ear cress) B3 domain-containing transcription factor VRN1 | Swiss-Prot |
| Scaffold_3 | 5310000 | 5319999 | lgreen | ppa026804m | 5317079 | 5320158 | VRN1_ARATH | Arabidopsis thaliana (Mouse-ear cress) B3 domain-containing transcription factor VRN1 | Swiss-Prot |
| Scaffold_3 | 5310000 | 5319999 | purple | ppa026804m | 5317079 | 5320158 | VRN1_ARATH | Arabidopsis thaliana (Mouse-ear cress) B3 domain-containing transcription factor VRN1 | Swiss-Prot |
| Scaffold_3 | 7520000 | 7529999 | blue | ppa007284m | 7526778 | 7532666 | RD23A_ARATH | Arabidopsis thaliana (Mouse-ear cress) Putative DNA repair protein RAD23-1 | Swiss-Prot |
| Scaffold_3 | 7520000 | 7529999 | dblue | ppa007284m | 7526778 | 7532666 | RD23A_ARATH | Arabidopsis thaliana (Mouse-ear cress) Putative DNA repair protein RAD23-1 | Swiss-Prot |
| Scaffold_3 | 7520000 | 7529999 | green | ppa007284m | 7526778 | 7532666 | RD23A_ARATH | Arabidopsis thaliana (Mouse-ear cress) Putative DNA repair protein RAD23-1 | Swiss-Prot |
| Scaffold_3 | 7520000 | 7529999 | lgreen | ppa007284m | 7526778 | 7532666 | RD23A_ARATH | Arabidopsis thaliana (Mouse-ear cress) Putative DNA repair protein RAD23-1 | Swiss-Prot |
| Scaffold_3 | 7520000 | 7529999 | purple | ppa007284m | 7526778 | 7532666 | RD23A_ARATH | Arabidopsis thaliana (Mouse-ear cress) Putative DNA repair protein RAD23-1 | Swiss-Prot |
| Scaffold_3 | 11420000 | 11429999 | blue | ppa010658m | 11423624 | 11424776 | TLP2_PRUPE | Prunus persica (Peach) Thaumatin-like protein 2 | Swiss-Prot |
| Scaffold_3 | 11420000 | 11429999 | dblue | ppa010658m | 11423624 | 11424776 | TLP2_PRUPE | Prunus persica (Peach) Thaumatin-like protein 2 | Swiss-Prot |
| Scaffold_3 | 11420000 | 11429999 | green | ppa010658m | 11423624 | 11424776 | TLP2_PRUPE | Prunus persica (Peach) Thaumatin-like protein 2 | Swiss-Prot |
| Scaffold_3 | 11420000 | 11429999 | lgreen | ppa010658m | 11423624 | 11424776 | TLP2_PRUPE | Prunus persica (Peach) Thaumatin-like protein 2 | Swiss-Prot |
| Scaffold_3 | 11420000 | 11429999 | purple | ppa010658m | 11423624 | 11424776 | TLP2_PRUPE | Prunus persica (Peach) Thaumatin-like protein 2 | Swiss-Prot |
| Scaffold_3 | 15640000 | 15649999 | blue | ppa003301m | 15648482 | 15651467 | AT5G48160.2 | Arabidopsis thaliana OBE2 (OBERON2); protein binding / zinc ion binding | TAIR |
| Scaffold_3 | 15640000 | 15649999 | dblue | ppa003301m | 15648482 | 15651467 | AT5G48160.2 | Arabidopsis thaliana OBE2 (OBERON2); protein binding / zinc ion binding | TAIR |
| Scaffold_3 | 15640000 | 15649999 | green | ppa003301m | 15648482 | 15651467 | AT5G48160.2 | Arabidopsis thaliana OBE2 (OBERON2); protein binding / zinc ion binding | TAIR |
| Scaffold_3 | 15640000 | 15649999 | lgreen | ppa003301m | 15648482 | 15651467 | AT5G48160.2 | Arabidopsis thaliana OBE2 (OBERON2); protein binding / zinc ion binding | TAIR |
| Scaffold_3 | 15640000 | 15649999 | purple | ppa003301m | 15648482 | 15651467 | AT5G48160.2 | Arabidopsis thaliana OBE2 (OBERON2); protein binding / zinc ion binding | TAIR |
| Scaffold_3 | 15650000 | 15659999 | blue | ppa019604m | 15651594 | 15651935 | AT5G26730.1 | Arabidopsis thaliana FUNCTIONS IN: molecular_function unknown; INVOLVED IN: biological_process unknown | TAIR |
| Scaffold_3 | 15650000 | 15659999 | dblue | ppa019604m | 15651594 | 15651935 | AT5G26730.1 | Arabidopsis thaliana FUNCTIONS IN: molecular_function unknown; INVOLVED IN: biological_process unknown | TAIR |
| Scaffold_3 | 15650000 | 15659999 | green | ppa019604m | 15651594 | 15651935 | AT5G26730.1 | Arabidopsis thaliana FUNCTIONS IN: molecular_function unknown; INVOLVED IN: biological_process unknown | TAIR |
| Scaffold_3 | 15650000 | 15659999 | lgreen | ppa019604m | 15651594 | 15651935 | AT5G26730.1 | Arabidopsis thaliana FUNCTIONS IN: molecular_function unknown; INVOLVED IN: biological_process unknown | TAIR |
| Scaffold_3 | 16490000 | 16499999 | blue | ppa005320m | 16495678 | 16499892 | ODO2_DICDI | Dictyostelium discoideum (Slime mold) "Dihydrolipoyllysine-residue succinyltransferase component of 2-oxoglutarate dehydrogenase complex, mitochondrial" | Swiss-Prot |
| Scaffold_3 | 16490000 | 16499999 | dblue | ppa005320m | 16495678 | 16499892 | ODO2_DICDI | Dictyostelium discoideum (Slime mold) "Dihydrolipoyllysine-residue succinyltransferase component of 2-oxoglutarate dehydrogenase complex, mitochondrial" | Swiss-Prot |
| Scaffold_3 | 16490000 | 16499999 | green | ppa005320m | 16495678 | 16499892 | ODO2_DICDI | Dictyostelium discoideum (Slime mold) "Dihydrolipoyllysine-residue succinyltransferase component of 2-oxoglutarate dehydrogenase complex, mitochondrial" | Swiss-Prot |
| Scaffold_3 | 16490000 | 16499999 | lgreen | ppa005320m | 16495678 | 16499892 | ODO2_DICDI | Dictyostelium discoideum (Slime mold) "Dihydrolipoyllysine-residue succinyltransferase component of 2-oxoglutarate dehydrogenase complex, mitochondrial" | Swiss-Prot |
| Scaffold_3 | 16500000 | 16509999 | blue | ppa003877m | 16508775 | 16512042 | ASOL_BRANA | Brassica napus (Rape) L-ascorbate oxidase homolog | Swiss-Prot |
| Scaffold_3 | 16500000 | 16509999 | dblue | ppa003877m | 16508775 | 16512042 | ASOL_BRANA | Brassica napus (Rape) L-ascorbate oxidase homolog | Swiss-Prot |
| Scaffold_3 | 16500000 | 16509999 | green | ppa003877m | 16508775 | 16512042 | ASOL_BRANA | Brassica napus (Rape) L-ascorbate oxidase homolog | Swiss-Prot |
| Scaffold_3 | 16500000 | 16509999 | lgreen | ppa003877m | 16508775 | 16512042 | ASOL_BRANA | Brassica napus (Rape) L-ascorbate oxidase homolog | Swiss-Prot |
| Scaffold_3 | 16500000 | 16509999 | purple | ppa003877m | 16508775 | 16512042 | ASOL_BRANA | Brassica napus (Rape) L-ascorbate oxidase homolog | Swiss-Prot |
| Scaffold_3 | 21160000 | 21169999 | blue | ppa018758m | 21164951 | 21166212 | AT4G18810.1 | Arabidopsis thaliana binding / catalytic/ transcription repressor | TAIR |
| Scaffold_3 | 21160000 | 21169999 | dblue | ppa018758m | 21164951 | 21166212 | AT4G18810.1 | Arabidopsis thaliana binding / catalytic/ transcription repressor | TAIR |
| Scaffold_3 | 21160000 | 21169999 | green | ppa018758m | 21164951 | 21166212 | AT4G18810.1 | Arabidopsis thaliana binding / catalytic/ transcription repressor | TAIR |
| Scaffold_3 | 21160000 | 21169999 | lgreen | ppa018758m | 21164951 | 21166212 | AT4G18810.1 | Arabidopsis thaliana binding / catalytic/ transcription repressor | TAIR |
| Scaffold_3 | 21160000 | 21169999 | purple | ppa018758m | 21164951 | 21166212 | AT4G18810.1 | Arabidopsis thaliana binding / catalytic/ transcription repressor | TAIR |
| Scaffold_3 | 21230000 | 21239999 | blue | ppa019812m | 21237443 | 21239580 | LHT1_ARATH | Arabidopsis thaliana (Mouse-ear cress) Lysine histidine transporter 1 | Swiss-Prot |
| Scaffold_3 | 21230000 | 21239999 | dblue | ppa019812m | 21237443 | 21239580 | LHT1_ARATH | Arabidopsis thaliana (Mouse-ear cress) Lysine histidine transporter 1 | Swiss-Prot |
| Scaffold_3 | 21230000 | 21239999 | green | ppa019812m | 21237443 | 21239580 | LHT1_ARATH | Arabidopsis thaliana (Mouse-ear cress) Lysine histidine transporter 1 | Swiss-Prot |
| Scaffold_3 | 21230000 | 21239999 | lgreen | ppa019812m | 21237443 | 21239580 | LHT1_ARATH | Arabidopsis thaliana (Mouse-ear cress) Lysine histidine transporter 1 | Swiss-Prot |
| Scaffold_3 | 21230000 | 21239999 | purple | ppa019812m | 21237443 | 21239580 | LHT1_ARATH | Arabidopsis thaliana (Mouse-ear cress) Lysine histidine transporter 1 | Swiss-Prot |
| Scaffold_4 | 2020000 | 2029999 | blue | ppa015051m | 2020820 | 2022223 | Y4990_ARATH | Arabidopsis thaliana (Mouse-ear cress) Uncharacterized protein At4g19900 | Swiss-Prot |
| Scaffold_4 | 2020000 | 2029999 | dblue | ppa015051m | 2020820 | 2022223 | Y4990_ARATH | Arabidopsis thaliana (Mouse-ear cress) Uncharacterized protein At4g19900 | Swiss-Prot |
| Scaffold_4 | 2020000 | 2029999 | green | ppa015051m | 2020820 | 2022223 | Y4990_ARATH | Arabidopsis thaliana (Mouse-ear cress) Uncharacterized protein At4g19900 | Swiss-Prot |
| Scaffold_4 | 2020000 | 2029999 | lgreen | ppa015051m | 2020820 | 2022223 | Y4990_ARATH | Arabidopsis thaliana (Mouse-ear cress) Uncharacterized protein At4g19900 | Swiss-Prot |
| Scaffold_4 | 2020000 | 2029999 | purple | ppa015051m | 2020820 | 2022223 | Y4990_ARATH | Arabidopsis thaliana (Mouse-ear cress) Uncharacterized protein At4g19900 | Swiss-Prot |
| Scaffold_4 | 2380000 | 2389999 | blue | ppa024902m | 2389020 | 2389921 | AT4G21020.1 | Arabidopsis thaliana late embryogenesis abundant domain-containing protein / LEA domain-containing protein | TAIR |
| Scaffold_4 | 2380000 | 2389999 | dblue | ppa024902m | 2389020 | 2389921 | AT4G21020.1 | Arabidopsis thaliana late embryogenesis abundant domain-containing protein / LEA domain-containing protein | TAIR |
| Scaffold_4 | 2380000 | 2389999 | green | ppa024902m | 2389020 | 2389921 | AT4G21020.1 | Arabidopsis thaliana late embryogenesis abundant domain-containing protein / LEA domain-containing protein | TAIR |
| Scaffold_4 | 2380000 | 2389999 | lgreen | ppa024902m | 2389020 | 2389921 | AT4G21020.1 | Arabidopsis thaliana late embryogenesis abundant domain-containing protein / LEA domain-containing protein | TAIR |
| Scaffold_4 | 2410000 | 2419999 | blue | ppa001854m | 2415957 | 2421480 | TYSD1_HUMAN | Homo sapiens (Human) Peroxisomal leader peptide-processing protease | Swiss-Prot |
| Scaffold_4 | 2410000 | 2419999 | dblue | ppa001854m | 2415957 | 2421480 | TYSD1_HUMAN | Homo sapiens (Human) Peroxisomal leader peptide-processing protease | Swiss-Prot |
| Scaffold_4 | 2410000 | 2419999 | green | ppa001854m | 2415957 | 2421480 | TYSD1_HUMAN | Homo sapiens (Human) Peroxisomal leader peptide-processing protease | Swiss-Prot |
| Scaffold_4 | 2410000 | 2419999 | lgreen | ppa001854m | 2415957 | 2421480 | TYSD1_HUMAN | Homo sapiens (Human) Peroxisomal leader peptide-processing protease | Swiss-Prot |
| Scaffold_4 | 2420000 | 2429999 | blue | ppa009446m | 2425264 | 2429942 | RING2_HUMAN | Homo sapiens (Human) E3 ubiquitin-protein ligase RING2 | Swiss-Prot |
| Scaffold_4 | 2420000 | 2429999 | dblue | ppa009446m | 2425264 | 2429942 | RING2_HUMAN | Homo sapiens (Human) E3 ubiquitin-protein ligase RING2 | Swiss-Prot |
| Scaffold_4 | 2420000 | 2429999 | green | ppa009446m | 2425264 | 2429942 | RING2_HUMAN | Homo sapiens (Human) E3 ubiquitin-protein ligase RING2 | Swiss-Prot |
| Scaffold_4 | 2420000 | 2429999 | lgreen | ppa009446m | 2425264 | 2429942 | RING2_HUMAN | Homo sapiens (Human) E3 ubiquitin-protein ligase RING2 | Swiss-Prot |
| Scaffold_4 | 5950000 | 5959999 | blue | ppa016324m | 5953676 | 5957072 | SEY1_DICDI | Dictyostelium discoideum (Slime mold) Protein SEY1 homolog | Swiss-Prot |
| Scaffold_4 | 5950000 | 5959999 | dblue | ppa016324m | 5953676 | 5957072 | SEY1_DICDI | Dictyostelium discoideum (Slime mold) Protein SEY1 homolog | Swiss-Prot |
| Scaffold_4 | 5950000 | 5959999 | green | ppa016324m | 5953676 | 5957072 | SEY1_DICDI | Dictyostelium discoideum (Slime mold) Protein SEY1 homolog | Swiss-Prot |
| Scaffold_4 | 5950000 | 5959999 | lgreen | ppa016324m | 5953676 | 5957072 | SEY1_DICDI | Dictyostelium discoideum (Slime mold) Protein SEY1 homolog | Swiss-Prot |
| Scaffold_4 | 5950000 | 5959999 | purple | ppa016324m | 5953676 | 5957072 | SEY1_DICDI | Dictyostelium discoideum (Slime mold) Protein SEY1 homolog | Swiss-Prot |
| Scaffold_4 | 11880000 | 11889999 | blue | ppb015779m | 11880583 | 11881563 | AT4G27300.1 | Arabidopsis thaliana "S-locus protein kinase, putative" | TAIR |
| Scaffold_4 | 11880000 | 11889999 | dblue | ppb015779m | 11880583 | 11881563 | AT4G27300.1 | Arabidopsis thaliana "S-locus protein kinase, putative" | TAIR |
| Scaffold_4 | 11880000 | 11889999 | green | ppb015779m | 11880583 | 11881563 | AT4G27300.1 | Arabidopsis thaliana "S-locus protein kinase, putative" | TAIR |
| Scaffold_4 | 11880000 | 11889999 | lgreen | ppb015779m | 11880583 | 11881563 | AT4G27300.1 | Arabidopsis thaliana "S-locus protein kinase, putative" | TAIR |
| Scaffold_4 | 11880000 | 11889999 | purple | ppb015779m | 11880583 | 11881563 | AT4G27300.1 | Arabidopsis thaliana "S-locus protein kinase, putative" | TAIR |
| Scaffold_4 | 20380000 | 20389999 | blue | ppb012679m | 20385193 | 20387335 | AT5G28950.1 | Arabidopsis thaliana unknown protein | TAIR |
| Scaffold_4 | 20380000 | 20389999 | dblue | ppb012679m | 20385193 | 20387335 | AT5G28950.1 | Arabidopsis thaliana unknown protein | TAIR |
| Scaffold_4 | 20380000 | 20389999 | green | ppb012679m | 20385193 | 20387335 | AT5G28950.1 | Arabidopsis thaliana unknown protein | TAIR |
| Scaffold_4 | 20380000 | 20389999 | lgreen | ppb012679m | 20385193 | 20387335 | AT5G28950.1 | Arabidopsis thaliana unknown protein | TAIR |
| Scaffold_4 | 20380000 | 20389999 | purple | ppb012679m | 20385193 | 20387335 | AT5G28950.1 | Arabidopsis thaliana unknown protein | TAIR |
| Scaffold_4 | 20430000 | 20439999 | blue | ppb023407m | 20434290 | 20435258 | R13L1_ARATH | Arabidopsis thaliana (Mouse-ear cress) Putative disease resistance RPP13-like protein 1 | Swiss-Prot |
| Scaffold_4 | 20430000 | 20439999 | dblue | ppb023407m | 20434290 | 20435258 | R13L1_ARATH | Arabidopsis thaliana (Mouse-ear cress) Putative disease resistance RPP13-like protein 1 | Swiss-Prot |
| Scaffold_4 | 20430000 | 20439999 | green | ppb023407m | 20434290 | 20435258 | R13L1_ARATH | Arabidopsis thaliana (Mouse-ear cress) Putative disease resistance RPP13-like protein 1 | Swiss-Prot |
| Scaffold_4 | 20430000 | 20439999 | lgreen | ppb023407m | 20434290 | 20435258 | R13L1_ARATH | Arabidopsis thaliana (Mouse-ear cress) Putative disease resistance RPP13-like protein 1 | Swiss-Prot |
| Scaffold_4 | 21850000 | 21859999 | blue | ppb018704m | 21854590 | 21856367 | AT2G10560.1 | Arabidopsis thaliana unknown protein | TAIR |
| Scaffold_4 | 21850000 | 21859999 | dblue | ppb018704m | 21854590 | 21856367 | AT2G10560.1 | Arabidopsis thaliana unknown protein | TAIR |
| Scaffold_4 | 21850000 | 21859999 | green | ppb018704m | 21854590 | 21856367 | AT2G10560.1 | Arabidopsis thaliana unknown protein | TAIR |
| Scaffold_4 | 21850000 | 21859999 | lgreen | ppb018704m | 21854590 | 21856367 | AT2G10560.1 | Arabidopsis thaliana unknown protein | TAIR |
| Scaffold_4 | 21850000 | 21859999 | purple | ppb018704m | 21854590 | 21856367 | AT2G10560.1 | Arabidopsis thaliana unknown protein | TAIR |
| Scaffold_4 | 23220000 | 23229999 | blue | ppa022684m | 23229470 | 23232252 | POL2_MOUSE | Mus musculus (Mouse) Retrovirus-related Pol polyprotein LINE-1 | Swiss-Prot |
| Scaffold_4 | 23220000 | 23229999 | dblue | ppa022684m | 23229470 | 23232252 | POL2_MOUSE | Mus musculus (Mouse) Retrovirus-related Pol polyprotein LINE-1 | Swiss-Prot |
| Scaffold_4 | 23220000 | 23229999 | green | ppa022684m | 23229470 | 23232252 | POL2_MOUSE | Mus musculus (Mouse) Retrovirus-related Pol polyprotein LINE-1 | Swiss-Prot |
| Scaffold_4 | 23220000 | 23229999 | lgreen | ppa022684m | 23229470 | 23232252 | POL2_MOUSE | Mus musculus (Mouse) Retrovirus-related Pol polyprotein LINE-1 | Swiss-Prot |
| Scaffold_5 | 7880000 | 7889999 | blue | ppa000974m | 7884954 | 7892237 | HOS1_ARATH | Arabidopsis thaliana (Mouse-ear cress) E3 ubiquitin-protein ligase HOS1 | Swiss-Prot |
| Scaffold_5 | 7880000 | 7889999 | dblue | ppa000974m | 7884954 | 7892237 | HOS1_ARATH | Arabidopsis thaliana (Mouse-ear cress) E3 ubiquitin-protein ligase HOS1 | Swiss-Prot |
| Scaffold_5 | 7880000 | 7889999 | green | ppa000974m | 7884954 | 7892237 | HOS1_ARATH | Arabidopsis thaliana (Mouse-ear cress) E3 ubiquitin-protein ligase HOS1 | Swiss-Prot |
| Scaffold_5 | 7880000 | 7889999 | lgreen | ppa000974m | 7884954 | 7892237 | HOS1_ARATH | Arabidopsis thaliana (Mouse-ear cress) E3 ubiquitin-protein ligase HOS1 | Swiss-Prot |
| Scaffold_5 | 7880000 | 7889999 | purple | ppa000974m | 7884954 | 7892237 | HOS1_ARATH | Arabidopsis thaliana (Mouse-ear cress) E3 ubiquitin-protein ligase HOS1 | Swiss-Prot |
| Scaffold_5 | 15190000 | 15199999 | blue | ppa024758m | 15194566 | 15196204 | AT5G02700.1 | Arabidopsis thaliana F-box family protein | TAIR |
| Scaffold_5 | 15190000 | 15199999 | dblue | ppa024758m | 15194566 | 15196204 | AT5G02700.1 | Arabidopsis thaliana F-box family protein | TAIR |
| Scaffold_5 | 15190000 | 15199999 | green | ppa024758m | 15194566 | 15196204 | AT5G02700.1 | Arabidopsis thaliana F-box family protein | TAIR |
| Scaffold_5 | 15190000 | 15199999 | lgreen | ppa024758m | 15194566 | 15196204 | AT5G02700.1 | Arabidopsis thaliana F-box family protein | TAIR |
| Scaffold_5 | 15510000 | 15519999 | blue | ppa022543m | 15514578 | 15519637 | AB2A_ARATH | Arabidopsis thaliana (Mouse-ear cress) ABC transporter A family member 2 | Swiss-Prot |
| Scaffold_5 | 15510000 | 15519999 | dblue | ppa022543m | 15514578 | 15519637 | AB2A_ARATH | Arabidopsis thaliana (Mouse-ear cress) ABC transporter A family member 2 | Swiss-Prot |
| Scaffold_5 | 15510000 | 15519999 | green | ppa022543m | 15514578 | 15519637 | AB2A_ARATH | Arabidopsis thaliana (Mouse-ear cress) ABC transporter A family member 2 | Swiss-Prot |
| Scaffold_5 | 15510000 | 15519999 | lgreen | ppa022543m | 15514578 | 15519637 | AB2A_ARATH | Arabidopsis thaliana (Mouse-ear cress) ABC transporter A family member 2 | Swiss-Prot |
| Scaffold_5 | 15510000 | 15519999 | purple | ppa022543m | 15514578 | 15519637 | AB2A_ARATH | Arabidopsis thaliana (Mouse-ear cress) ABC transporter A family member 2 | Swiss-Prot |
| Scaffold_5 | 16110000 | 16119999 | blue | ppa000133m | 16118504 | 16126374 | SCAPE_HUMAN | Homo sapiens (Human) S phase cyclin A-associated protein in the endoplasmic reticulum | Swiss-Prot |
| Scaffold_5 | 16110000 | 16119999 | dblue | ppa000133m | 16118504 | 16126374 | SCAPE_HUMAN | Homo sapiens (Human) S phase cyclin A-associated protein in the endoplasmic reticulum | Swiss-Prot |
| Scaffold_5 | 16110000 | 16119999 | green | ppa000133m | 16118504 | 16126374 | SCAPE_HUMAN | Homo sapiens (Human) S phase cyclin A-associated protein in the endoplasmic reticulum | Swiss-Prot |
| Scaffold_5 | 16110000 | 16119999 | lgreen | ppa000133m | 16118504 | 16126374 | SCAPE_HUMAN | Homo sapiens (Human) S phase cyclin A-associated protein in the endoplasmic reticulum | Swiss-Prot |
| Scaffold_5 | 16110000 | 16119999 | purple | ppa000133m | 16118504 | 16126374 | SCAPE_HUMAN | Homo sapiens (Human) S phase cyclin A-associated protein in the endoplasmic reticulum | Swiss-Prot |
| Scaffold_5 | 16380000 | 16389999 | blue | ppa020262m | 16388127 | 16390492 | AT5G61120.1 | EXPRESSED DURING: 6 growth stages; BEST Arabidopsis thaliana protein match is: nucleic acid binding (TAIR:AT5G61090.1) | TAIR |
| Scaffold_5 | 16380000 | 16389999 | dblue | ppa020262m | 16388127 | 16390492 | AT5G61120.1 | Arabidopsis thaliana INVOLVED IN: biological_process unknown; EXPRESSED IN: 8 plant structures; | TAIR |
| Scaffold_5 | 16380000 | 16389999 | green | ppa020262m | 16388127 | 16390492 | AT5G61120.1 | Arabidopsis thaliana INVOLVED IN: biological_process unknown; EXPRESSED IN: 8 plant structures; | TAIR |
| Scaffold_5 | 16380000 | 16389999 | lgreen | ppa020262m | 16388127 | 16390492 | AT5G61120.1 | Arabidopsis thaliana INVOLVED IN: biological_process unknown; EXPRESSED IN: 8 plant structures; | TAIR |
| Scaffold_5 | 16380000 | 16389999 | purple | ppa020262m | 16388127 | 16390492 | AT5G61120.1 | Arabidopsis thaliana INVOLVED IN: biological_process unknown; EXPRESSED IN: 8 plant structures; | TAIR |
| Scaffold_5 | 16390000 | 16399999 | blue | ppa003353m | 16395644 | 16400857 | HDA5_ARATH | Arabidopsis thaliana (Mouse-ear cress) Histone deacetylase 5 | Swiss-Prot |
| Scaffold_5 | 16390000 | 16399999 | dblue | ppa003353m | 16395644 | 16400857 | HDA5_ARATH | Arabidopsis thaliana (Mouse-ear cress) Histone deacetylase 5 | Swiss-Prot |
| Scaffold_5 | 16390000 | 16399999 | green | ppa003353m | 16395644 | 16400857 | HDA5_ARATH | Arabidopsis thaliana (Mouse-ear cress) Histone deacetylase 5 | Swiss-Prot |
| Scaffold_5 | 16390000 | 16399999 | lgreen | ppa003353m | 16395644 | 16400857 | HDA5_ARATH | Arabidopsis thaliana (Mouse-ear cress) Histone deacetylase 5 | Swiss-Prot |
| Scaffold_5 | 16410000 | 16419999 | blue | ppa002101m | 16418437 | 16422940 | RBOHF_ARATH | Arabidopsis thaliana (Mouse-ear cress) Respiratory burst oxidase homolog protein F | Swiss-Prot |
| Scaffold_5 | 16410000 | 16419999 | dblue | ppa002101m | 16418437 | 16422940 | RBOHF_ARATH | Arabidopsis thaliana (Mouse-ear cress) Respiratory burst oxidase homolog protein F | Swiss-Prot |
| Scaffold_5 | 16410000 | 16419999 | green | ppa002101m | 16418437 | 16422940 | RBOHF_ARATH | Arabidopsis thaliana (Mouse-ear cress) Respiratory burst oxidase homolog protein F | Swiss-Prot |
| Scaffold_5 | 16410000 | 16419999 | lgreen | ppa002101m | 16418437 | 16422940 | RBOHF_ARATH | Arabidopsis thaliana (Mouse-ear cress) Respiratory burst oxidase homolog protein F | Swiss-Prot |
| Scaffold_5 | 16410000 | 16419999 | purple | ppa002101m | 16418437 | 16422940 | RBOHF_ARATH | Arabidopsis thaliana (Mouse-ear cress) Respiratory burst oxidase homolog protein F | Swiss-Prot |
| Scaffold_5 | 16460000 | 16469999 | blue | ppa005346m | 16469317 | 16473788 | G3BP_SCHPO | Schizosaccharomyces pombe (Fission yeast) Putative G3BP-like protein | Swiss-Prot |
| Scaffold_5 | 16460000 | 16469999 | dblue | ppa005346m | 16469317 | 16473788 | G3BP_SCHPO | Schizosaccharomyces pombe (Fission yeast) Putative G3BP-like protein | Swiss-Prot |
| Scaffold_5 | 16460000 | 16469999 | green | ppa005346m | 16469317 | 16473788 | G3BP_SCHPO | Schizosaccharomyces pombe (Fission yeast) Putative G3BP-like protein | Swiss-Prot |
| Scaffold_5 | 16460000 | 16469999 | lgreen | ppa005346m | 16469317 | 16473788 | G3BP_SCHPO | Schizosaccharomyces pombe (Fission yeast) Putative G3BP-like protein | Swiss-Prot |
| Scaffold_5 | 16460000 | 16469999 | purple | ppa005346m | 16469317 | 16473788 | G3BP_SCHPO | Schizosaccharomyces pombe (Fission yeast) Putative G3BP-like protein | Swiss-Prot |
| Scaffold_5 | 17720000 | 17729999 | blue | ppa005395m | 17722727 | 17727207 | PEAM1_ARATH | Arabidopsis thaliana (Mouse-ear cress) Phosphoethanolamine N-methyltransferase 1 | Swiss-Prot |
| Scaffold_5 | 17720000 | 17729999 | dblue | ppa005395m | 17722727 | 17727207 | PEAM1_ARATH | Arabidopsis thaliana (Mouse-ear cress) Phosphoethanolamine N-methyltransferase 1 | Swiss-Prot |
| Scaffold_5 | 17720000 | 17729999 | green | ppa005395m | 17722727 | 17727207 | PEAM1_ARATH | Arabidopsis thaliana (Mouse-ear cress) Phosphoethanolamine N-methyltransferase 1 | Swiss-Prot |
| Scaffold_5 | 17720000 | 17729999 | lgreen | ppa005395m | 17722727 | 17727207 | PEAM1_ARATH | Arabidopsis thaliana (Mouse-ear cress) Phosphoethanolamine N-methyltransferase 1 | Swiss-Prot |
| Scaffold_5 | 17720000 | 17729999 | purple | ppa005395m | 17722727 | 17727207 | PEAM1_ARATH | Arabidopsis thaliana (Mouse-ear cress) Phosphoethanolamine N-methyltransferase 1 | Swiss-Prot |
| Scaffold_5 | 17730000 | 17739999 | blue | ppa002944m | 17737211 | 17740595 | PINI_ARATH | Arabidopsis thaliana (Mouse-ear cress) Auxin efflux carrier component 1 | Swiss-Prot |
| Scaffold_5 | 17730000 | 17739999 | dblue | ppa002944m | 17737211 | 17740595 | PINI_ARATH | Arabidopsis thaliana (Mouse-ear cress) Auxin efflux carrier component 1 | Swiss-Prot |
| Scaffold_5 | 17730000 | 17739999 | green | ppa002944m | 17737211 | 17740595 | PINI_ARATH | Arabidopsis thaliana (Mouse-ear cress) Auxin efflux carrier component 1 | Swiss-Prot |
| Scaffold_5 | 17730000 | 17739999 | lgreen | ppa002944m | 17737211 | 17740595 | PINI_ARATH | Arabidopsis thaliana (Mouse-ear cress) Auxin efflux carrier component 1 | Swiss-Prot |
| Scaffold_5 | 17730000 | 17739999 | purple | ppa002944m | 17737211 | 17740595 | PINI_ARATH | Arabidopsis thaliana (Mouse-ear cress) Auxin efflux carrier component 1 | Swiss-Prot |
| Scaffold_5 | 17820000 | 17829999 | blue | ppa012361m | 17825695 | 17827120 | VAS_ARATH | Arabidopsis thaliana (Mouse-ear cress) Lipid transfer-like protein VAS | Swiss-Prot |
| Scaffold_5 | 17820000 | 17829999 | dblue | ppa012361m | 17825695 | 17827120 | VAS_ARATH | Arabidopsis thaliana (Mouse-ear cress) Lipid transfer-like protein VAS | Swiss-Prot |
| Scaffold_5 | 17820000 | 17829999 | green | ppa012361m | 17825695 | 17827120 | VAS_ARATH | Arabidopsis thaliana (Mouse-ear cress) Lipid transfer-like protein VAS | Swiss-Prot |
| Scaffold_5 | 17820000 | 17829999 | lgreen | ppa012361m | 17825695 | 17827120 | VAS_ARATH | Arabidopsis thaliana (Mouse-ear cress) Lipid transfer-like protein VAS | Swiss-Prot |
| Scaffold_5 | 17820000 | 17829999 | purple | ppa012361m | 17825695 | 17827120 | VAS_ARATH | Arabidopsis thaliana (Mouse-ear cress) Lipid transfer-like protein VAS | Swiss-Prot |
| Scaffold_5 | 17910000 | 17919999 | blue | ppa011098m | 17916661 | 17917727 | M2K4_ARATH | Arabidopsis thaliana (Mouse-ear cress) Mitogen-activated protein kinase kinase 4 | Swiss-Prot |
| Scaffold_5 | 17910000 | 17919999 | dblue | ppa011098m | 17916661 | 17917727 | M2K4_ARATH | Arabidopsis thaliana (Mouse-ear cress) Mitogen-activated protein kinase kinase 4 | Swiss-Prot |
| Scaffold_5 | 17910000 | 17919999 | green | ppa011098m | 17916661 | 17917727 | M2K4_ARATH | Arabidopsis thaliana (Mouse-ear cress) Mitogen-activated protein kinase kinase 4 | Swiss-Prot |
| Scaffold_5 | 17910000 | 17919999 | lgreen | ppa011098m | 17916661 | 17917727 | M2K4_ARATH | Arabidopsis thaliana (Mouse-ear cress) Mitogen-activated protein kinase kinase 4 | Swiss-Prot |
| Scaffold_5 | 17990000 | 17999999 | blue | ppa016187m | 17997449 | 18000112 | MYBF_ARATH | Arabidopsis thaliana (Mouse-ear cress) Putative Myb family transcription factor At1g14600 | Swiss-Prot |
| Scaffold_5 | 17990000 | 17999999 | dblue | ppa016187m | 17997449 | 18000112 | MYBF_ARATH | Arabidopsis thaliana (Mouse-ear cress) Putative Myb family transcription factor At1g14600 | Swiss-Prot |
| Scaffold_5 | 17990000 | 17999999 | green | ppa016187m | 17997449 | 18000112 | MYBF_ARATH | Arabidopsis thaliana (Mouse-ear cress) Putative Myb family transcription factor At1g14600 | Swiss-Prot |
| Scaffold_5 | 17990000 | 17999999 | lgreen | ppa016187m | 17997449 | 18000112 | MYBF_ARATH | Arabidopsis thaliana (Mouse-ear cress) Putative Myb family transcription factor At1g14600 | Swiss-Prot |
| Scaffold_5 | 17990000 | 17999999 | purple | ppa016187m | 17997449 | 18000112 | MYBF_ARATH | Arabidopsis thaliana (Mouse-ear cress) Putative Myb family transcription factor At1g14600 | Swiss-Prot |
| Scaffold_5 | 18020000 | 18029999 | blue | ppa006664m | 18029174 | 18031545 | IP5P3_ARATH | Arabidopsis thaliana (Mouse-ear cress) "Type I inositol-1,4,5-trisphosphate 5-phosphatase CVP2" | Swiss-Prot |
| Scaffold_5 | 18020000 | 18029999 | dblue | ppa006664m | 18029174 | 18031545 | IP5P3_ARATH | Arabidopsis thaliana (Mouse-ear cress) "Type I inositol-1,4,5-trisphosphate 5-phosphatase CVP2" | Swiss-Prot |
| Scaffold_5 | 18020000 | 18029999 | green | ppa006664m | 18029174 | 18031545 | IP5P3_ARATH | Arabidopsis thaliana (Mouse-ear cress) "Type I inositol-1,4,5-trisphosphate 5-phosphatase CVP2" | Swiss-Prot |
| Scaffold_5 | 18020000 | 18029999 | lgreen | ppa006664m | 18029174 | 18031545 | IP5P3_ARATH | Arabidopsis thaliana (Mouse-ear cress) "Type I inositol-1,4,5-trisphosphate 5-phosphatase CVP2" | Swiss-Prot |
| Scaffold_5 | 18300000 | 18309999 | blue | ppa001999m | 18299590 | 18303956 | YSL8_ARATH | Arabidopsis thaliana (Mouse-ear cress) Probable metal-nicotianamine transporter YSL8 | Swiss-Prot |
| Scaffold_5 | 18300000 | 18309999 | dblue | ppa001999m | 18299590 | 18303956 | YSL8_ARATH | Arabidopsis thaliana (Mouse-ear cress) Probable metal-nicotianamine transporter YSL8 | Swiss-Prot |
| Scaffold_5 | 18300000 | 18309999 | green | ppa001999m | 18299590 | 18303956 | YSL8_ARATH | Arabidopsis thaliana (Mouse-ear cress) Probable metal-nicotianamine transporter YSL8 | Swiss-Prot |
| Scaffold_5 | 18300000 | 18309999 | lgreen | ppa001999m | 18299590 | 18303956 | YSL8_ARATH | Arabidopsis thaliana (Mouse-ear cress) Probable metal-nicotianamine transporter YSL8 | Swiss-Prot |
| Scaffold_5 | 18320000 | 18329999 | blue | ppa012487m | 18328728 | 18331844 | BTF3_HUMAN | Homo sapiens (Human) Transcription factor BTF3 | Swiss-Prot |
| Scaffold_5 | 18320000 | 18329999 | dblue | ppa012487m | 18328728 | 18331844 | BTF3_HUMAN | Homo sapiens (Human) Transcription factor BTF3 | Swiss-Prot |
| Scaffold_5 | 18320000 | 18329999 | green | ppa012487m | 18328728 | 18331844 | BTF3_HUMAN | Homo sapiens (Human) Transcription factor BTF3 | Swiss-Prot |
| Scaffold_5 | 18320000 | 18329999 | lgreen | ppa012487m | 18328728 | 18331844 | BTF3_HUMAN | Homo sapiens (Human) Transcription factor BTF3 | Swiss-Prot |
| Scaffold_6 | 9160000 | 9169999 | blue | ppa014117m | 9160028 | 9160670 | N/A | N/A N/A | N/A |
| Scaffold_6 | 9160000 | 9169999 | dblue | ppa014117m | 9160028 | 9160670 | N/A | N/A N/A | N/A |
| Scaffold_6 | 9160000 | 9169999 | green | ppa014117m | 9160028 | 9160670 | N/A | N/A N/A | N/A |
| Scaffold_6 | 9160000 | 9169999 | lgreen | ppa014117m | 9160028 | 9160670 | N/A | N/A N/A | N/A |
| Scaffold_6 | 14140000 | 14149999 | blue | ppb017462m | 14145915 | 14147281 | A5B099_VITVI | Vitis vinifera (Grape) Putative uncharacterized protein | TrEMBL |
| Scaffold_6 | 14140000 | 14149999 | dblue | ppb017462m | 14145915 | 14147281 | A5B099_VITVI | Vitis vinifera (Grape) Putative uncharacterized protein | TrEMBL |
| Scaffold_6 | 14140000 | 14149999 | green | ppb017462m | 14145915 | 14147281 | A5B099_VITVI | Vitis vinifera (Grape) Putative uncharacterized protein | TrEMBL |
| Scaffold_6 | 14140000 | 14149999 | lgreen | ppb017462m | 14145915 | 14147281 | A5B099_VITVI | Vitis vinifera (Grape) Putative uncharacterized protein | TrEMBL |
| Scaffold_6 | 14140000 | 14149999 | purple | ppb017462m | 14145915 | 14147281 | A5B099_VITVI | Vitis vinifera (Grape) Putative uncharacterized protein | TrEMBL |
| Scaffold_6 | 16340000 | 16349999 | blue | ppa007578m | 16341479 | 16347112 | TGA1_ARATH | Arabidopsis thaliana (Mouse-ear cress) Transcription factor TGA1 | Swiss-Prot |
| Scaffold_6 | 16340000 | 16349999 | dblue | ppa007578m | 16341479 | 16347112 | TGA1_ARATH | Arabidopsis thaliana (Mouse-ear cress) Transcription factor TGA1 | Swiss-Prot |
| Scaffold_6 | 16340000 | 16349999 | green | ppa007578m | 16341479 | 16347112 | TGA1_ARATH | Arabidopsis thaliana (Mouse-ear cress) Transcription factor TGA1 | Swiss-Prot |
| Scaffold_6 | 16340000 | 16349999 | lgreen | ppa007578m | 16341479 | 16347112 | TGA1_ARATH | Arabidopsis thaliana (Mouse-ear cress) Transcription factor TGA1 | Swiss-Prot |
| Scaffold_6 | 17680000 | 17689999 | blue | ppa008544m | 17686969 | 17689797 | S35A3_BOVIN | Bos taurus (Bovine) UDP-N-acetylglucosamine transporter | Swiss-Prot |
| Scaffold_6 | 17680000 | 17689999 | dblue | ppa008544m | 17686969 | 17689797 | S35A3_BOVIN | Bos taurus (Bovine) UDP-N-acetylglucosamine transporter | Swiss-Prot |
| Scaffold_6 | 17680000 | 17689999 | green | ppa008544m | 17686969 | 17689797 | S35A3_BOVIN | Bos taurus (Bovine) UDP-N-acetylglucosamine transporter | Swiss-Prot |
| Scaffold_6 | 17680000 | 17689999 | lgreen | ppa008544m | 17686969 | 17689797 | S35A3_BOVIN | Bos taurus (Bovine) UDP-N-acetylglucosamine transporter | Swiss-Prot |
| Scaffold_6 | 17680000 | 17689999 | purple | ppa008544m | 17686969 | 17689797 | S35A3_BOVIN | Bos taurus (Bovine) UDP-N-acetylglucosamine transporter | Swiss-Prot |
| Scaffold_6 | 21300000 | 21309999 | blue | ppa018515m | 21301307 | 21302534 | AT4G37360.1 | Arabidopsis thaliana CYP81D2; electron carrier/ heme binding / iron ion binding / monooxygenase/ oxygen binding | TAIR |
| Scaffold_6 | 21300000 | 21309999 | dblue | ppa018515m | 21301307 | 21302534 | AT4G37360.1 | Arabidopsis thaliana CYP81D2; electron carrier/ heme binding / iron ion binding / monooxygenase/ oxygen binding | TAIR |
| Scaffold_6 | 21300000 | 21309999 | green | ppa018515m | 21301307 | 21302534 | AT4G37360.1 | Arabidopsis thaliana CYP81D2; electron carrier/ heme binding / iron ion binding / monooxygenase/ oxygen binding | TAIR |
| Scaffold_6 | 21300000 | 21309999 | lgreen | ppa018515m | 21301307 | 21302534 | AT4G37360.1 | Arabidopsis thaliana CYP81D2; electron carrier/ heme binding / iron ion binding / monooxygenase/ oxygen binding | TAIR |
| Scaffold_6 | 21300000 | 21309999 | purple | ppa018515m | 21301307 | 21302534 | AT4G37360.1 | Arabidopsis thaliana CYP81D2; electron carrier/ heme binding / iron ion binding / monooxygenase/ oxygen binding | TAIR |
| Scaffold_6 | 27800000 | 27809999 | blue | ppa024915m | 27807801 | 27809122 | PP418_ARATH | Arabidopsis thaliana (Mouse-ear cress) Pentatricopeptide repeat-containing protein At5g46100 | Swiss-Prot |
| Scaffold_6 | 27800000 | 27809999 | dblue | ppa024915m | 27807801 | 27809122 | PP418_ARATH | Arabidopsis thaliana (Mouse-ear cress) Pentatricopeptide repeat-containing protein At5g46100 | Swiss-Prot |
| Scaffold_6 | 27800000 | 27809999 | green | ppa024915m | 27807801 | 27809122 | PP418_ARATH | Arabidopsis thaliana (Mouse-ear cress) Pentatricopeptide repeat-containing protein At5g46100 | Swiss-Prot |
| Scaffold_6 | 27800000 | 27809999 | lgreen | ppa024915m | 27807801 | 27809122 | PP418_ARATH | Arabidopsis thaliana (Mouse-ear cress) Pentatricopeptide repeat-containing protein At5g46100 | Swiss-Prot |
| Scaffold_6 | 27800000 | 27809999 | purple | ppa024915m | 27807801 | 27809122 | PP418_ARATH | Arabidopsis thaliana (Mouse-ear cress) Pentatricopeptide repeat-containing protein At5g46100 | Swiss-Prot |
| Scaffold_6 | 28440000 | 28449999 | blue | ppa003455m | 28447464 | 28452731 | C97B2_SOYBN | Glycine max (Soybean) Cytochrome P450 97B2 | Swiss-Prot |
| Scaffold_6 | 28440000 | 28449999 | dblue | ppa003455m | 28447464 | 28452731 | C97B2_SOYBN | Glycine max (Soybean) Cytochrome P450 97B2 | Swiss-Prot |
| Scaffold_6 | 28440000 | 28449999 | green | ppa003455m | 28447464 | 28452731 | C97B2_SOYBN | Glycine max (Soybean) Cytochrome P450 97B2 | Swiss-Prot |
| Scaffold_6 | 28440000 | 28449999 | lgreen | ppa003455m | 28447464 | 28452731 | C97B2_SOYBN | Glycine max (Soybean) Cytochrome P450 97B2 | Swiss-Prot |
| Scaffold_6 | 28440000 | 28449999 | purple | ppa003455m | 28447464 | 28452731 | C97B2_SOYBN | Glycine max (Soybean) Cytochrome P450 97B2 | Swiss-Prot |
| Scaffold_6 | 28460000 | 28469999 | blue | ppa023856m | 28469880 | 28473018 | WAK2_ARATH | Arabidopsis thaliana (Mouse-ear cress) Wall-associated receptor kinase 2 | Swiss-Prot |
| Scaffold_6 | 28460000 | 28469999 | dblue | ppa023856m | 28469880 | 28473018 | WAK2_ARATH | Arabidopsis thaliana (Mouse-ear cress) Wall-associated receptor kinase 2 | Swiss-Prot |
| Scaffold_6 | 28460000 | 28469999 | green | ppa023856m | 28469880 | 28473018 | WAK2_ARATH | Arabidopsis thaliana (Mouse-ear cress) Wall-associated receptor kinase 2 | Swiss-Prot |
| Scaffold_6 | 28460000 | 28469999 | lgreen | ppa023856m | 28469880 | 28473018 | WAK2_ARATH | Arabidopsis thaliana (Mouse-ear cress) Wall-associated receptor kinase 2 | Swiss-Prot |
| Scaffold_6 | 28460000 | 28469999 | purple | ppa023856m | 28469880 | 28473018 | WAK2_ARATH | Arabidopsis thaliana (Mouse-ear cress) Wall-associated receptor kinase 2 | Swiss-Prot |
| Scaffold_6 | 28790000 | 28799999 | blue | ppa005628m | 28797761 | 28803086 | HTSF1_HUMAN | Homo sapiens (Human) HIV Tat-specific factor 1 | Swiss-Prot |
| Scaffold_6 | 28790000 | 28799999 | dblue | ppa005628m | 28797761 | 28803086 | HTSF1_HUMAN | Homo sapiens (Human) HIV Tat-specific factor 1 | Swiss-Prot |
| Scaffold_6 | 28790000 | 28799999 | green | ppa005628m | 28797761 | 28803086 | HTSF1_HUMAN | Homo sapiens (Human) HIV Tat-specific factor 1 | Swiss-Prot |
| Scaffold_6 | 28790000 | 28799999 | lgreen | ppa005628m | 28797761 | 28803086 | HTSF1_HUMAN | Homo sapiens (Human) HIV Tat-specific factor 1 | Swiss-Prot |
| Scaffold_6 | 28790000 | 28799999 | purple | ppa005628m | 28797761 | 28803086 | HTSF1_HUMAN | Homo sapiens (Human) HIV Tat-specific factor 1 | Swiss-Prot |
| Scaffold_7 | 320000 | 329999 | blue | ppa016448m | 327054 | 328854 | D1H9A4_VITVI | Vitis vinifera (Grape) "Whole genome shotgun sequence of line PN40024, Scaffold_143.assembly12x" | TrEMBL |
| Scaffold_7 | 320000 | 329999 | dblue | ppa016448m | 327054 | 328854 | D1H9A4_VITVI | Vitis vinifera (Grape) "Whole genome shotgun sequence of line PN40024, Scaffold_143.assembly12x" | TrEMBL |
| Scaffold_7 | 320000 | 329999 | green | ppa016448m | 327054 | 328854 | D1H9A4_VITVI | Vitis vinifera (Grape) "Whole genome shotgun sequence of line PN40024, Scaffold_143.assembly12x" | TrEMBL |
| Scaffold_7 | 320000 | 329999 | lgreen | ppa016448m | 327054 | 328854 | D1H9A4_VITVI | Vitis vinifera (Grape) "Whole genome shotgun sequence of line PN40024, Scaffold_143.assembly12x" | TrEMBL |
| Scaffold_7 | 320000 | 329999 | purple | ppa016448m | 327054 | 328854 | D1H9A4_VITVI | Vitis vinifera (Grape) "Whole genome shotgun sequence of line PN40024, Scaffold_143.assembly12x" | TrEMBL |
| Scaffold_7 | 2990000 | 2999999 | blue | ppa021709m | 2992012 | 2996223 | AT1G64260.1 | Arabidopsis thaliana zinc finger protein-related | TAIR |
| Scaffold_7 | 2990000 | 2999999 | dblue | ppa021709m | 2992012 | 2996223 | AT1G64260.1 | Arabidopsis thaliana zinc finger protein-related | TAIR |
| Scaffold_7 | 2990000 | 2999999 | green | ppa021709m | 2992012 | 2996223 | AT1G64260.1 | Arabidopsis thaliana zinc finger protein-related | TAIR |
| Scaffold_7 | 2990000 | 2999999 | lgreen | ppa021709m | 2992012 | 2996223 | AT1G64260.1 | Arabidopsis thaliana zinc finger protein-related | TAIR |
| Scaffold_7 | 3090000 | 3099999 | blue | ppb013468m | 3095849 | 3101031 | AT1G64260.1 | Arabidopsis thaliana zinc finger protein-related | TAIR |
| Scaffold_7 | 3090000 | 3099999 | dblue | ppb013468m | 3095849 | 3101031 | AT1G64260.1 | Arabidopsis thaliana zinc finger protein-related | TAIR |
| Scaffold_7 | 3090000 | 3099999 | green | ppb013468m | 3095849 | 3101031 | AT1G64260.1 | Arabidopsis thaliana zinc finger protein-related | TAIR |
| Scaffold_7 | 3090000 | 3099999 | lgreen | ppb013468m | 3095849 | 3101031 | AT1G64260.1 | Arabidopsis thaliana zinc finger protein-related | TAIR |
| Scaffold_7 | 3090000 | 3099999 | purple | ppb013468m | 3095849 | 3101031 | AT1G64260.1 | Arabidopsis thaliana zinc finger protein-related | TAIR |
| Scaffold_7 | 3240000 | 3249999 | blue | ppa008977m | 3244118 | 3248601 | AT5G13890.3 | Arabidopsis thaliana unknown protein | TAIR |
| Scaffold_7 | 3240000 | 3249999 | dblue | ppa008977m | 3244118 | 3248601 | AT5G13890.3 | Arabidopsis thaliana unknown protein | TAIR |
| Scaffold_7 | 3240000 | 3249999 | green | ppa008977m | 3244118 | 3248601 | AT5G13890.3 | Arabidopsis thaliana unknown protein | TAIR |
| Scaffold_7 | 3240000 | 3249999 | lgreen | ppa008977m | 3244118 | 3248601 | AT5G13890.3 | Arabidopsis thaliana unknown protein | TAIR |
| Scaffold_7 | 11250000 | 11259999 | blue | ppa013597m | 11252551 | 11253451 | MAVI_CUCPE | Cucurbita pepo (Vegetable marrow) (Summer squash) Mavicyanin | Swiss-Prot |
| Scaffold_7 | 11250000 | 11259999 | dblue | ppa013597m | 11252551 | 11253451 | MAVI_CUCPE | Cucurbita pepo (Vegetable marrow) (Summer squash) Mavicyanin | Swiss-Prot |
| Scaffold_7 | 11250000 | 11259999 | green | ppa013597m | 11252551 | 11253451 | MAVI_CUCPE | Cucurbita pepo (Vegetable marrow) (Summer squash) Mavicyanin | Swiss-Prot |
| Scaffold_7 | 11250000 | 11259999 | lgreen | ppa013597m | 11252551 | 11253451 | MAVI_CUCPE | Cucurbita pepo (Vegetable marrow) (Summer squash) Mavicyanin | Swiss-Prot |
| Scaffold_7 | 11250000 | 11259999 | purple | ppa013597m | 11252551 | 11253451 | MAVI_CUCPE | Cucurbita pepo (Vegetable marrow) (Summer squash) Mavicyanin | Swiss-Prot |
| Scaffold_7 | 15020000 | 15029999 | blue | ppa023784m | 15028865 | 15030661 | PME15_ARATH | Arabidopsis thaliana (Mouse-ear cress) Probable pectinesterase 15 | Swiss-Prot |
| Scaffold_7 | 15020000 | 15029999 | dblue | ppa023784m | 15028865 | 15030661 | PME15_ARATH | Arabidopsis thaliana (Mouse-ear cress) Probable pectinesterase 15 | Swiss-Prot |
| Scaffold_7 | 15020000 | 15029999 | green | ppa023784m | 15028865 | 15030661 | PME15_ARATH | Arabidopsis thaliana (Mouse-ear cress) Probable pectinesterase 15 | Swiss-Prot |
| Scaffold_7 | 15020000 | 15029999 | lgreen | ppa023784m | 15028865 | 15030661 | PME15_ARATH | Arabidopsis thaliana (Mouse-ear cress) Probable pectinesterase 15 | Swiss-Prot |
| Scaffold_7 | 15020000 | 15029999 | purple | ppa023784m | 15028865 | 15030661 | PME15_ARATH | Arabidopsis thaliana (Mouse-ear cress) Probable pectinesterase 15 | Swiss-Prot |
| Scaffold_7 | 16140000 | 16149999 | blue | ppa024687m | 16142834 | 16145927 | CDA7L_HUMAN | Homo sapiens (Human) Cell division cycle-associated 7-like protein | Swiss-Prot |
| Scaffold_7 | 16140000 | 16149999 | dblue | ppa024687m | 16142834 | 16145927 | CDA7L_HUMAN | Homo sapiens (Human) Cell division cycle-associated 7-like protein | Swiss-Prot |
| Scaffold_7 | 16140000 | 16149999 | green | ppa024687m | 16142834 | 16145927 | CDA7L_HUMAN | Homo sapiens (Human) Cell division cycle-associated 7-like protein | Swiss-Prot |
| Scaffold_7 | 16140000 | 16149999 | lgreen | ppa024687m | 16142834 | 16145927 | CDA7L_HUMAN | Homo sapiens (Human) Cell division cycle-associated 7-like protein | Swiss-Prot |
| Scaffold_7 | 16280000 | 16289999 | blue | ppa005090m | 16286800 | 16290282 | AT4G37210.1 | Arabidopsis thaliana tetratricopeptide repeat (TPR)-containing protein | TAIR |
| Scaffold_7 | 16280000 | 16289999 | dblue | ppa005090m | 16286800 | 16290282 | AT4G37210.1 | Arabidopsis thaliana tetratricopeptide repeat (TPR)-containing protein | TAIR |
| Scaffold_7 | 16280000 | 16289999 | green | ppa005090m | 16286800 | 16290282 | AT4G37210.1 | Arabidopsis thaliana tetratricopeptide repeat (TPR)-containing protein | TAIR |
| Scaffold_7 | 16280000 | 16289999 | lgreen | ppa005090m | 16286800 | 16290282 | AT4G37210.1 | Arabidopsis thaliana tetratricopeptide repeat (TPR)-containing protein | TAIR |
| Scaffold_7 | 16280000 | 16289999 | purple | ppa005090m | 16286800 | 16290282 | AT4G37210.1 | Arabidopsis thaliana tetratricopeptide repeat (TPR)-containing protein | TAIR |
| Scaffold_7 | 16310000 | 16319999 | blue | ppa012835m | 16317212 | 16319158 | U497K_ARATH | Arabidopsis thaliana (Mouse-ear cress) UPF0497 membrane protein At3g50810 | Swiss-Prot |
| Scaffold_7 | 16310000 | 16319999 | dblue | ppa012835m | 16317212 | 16319158 | U497K_ARATH | Arabidopsis thaliana (Mouse-ear cress) UPF0497 membrane protein At3g50810 | Swiss-Prot |
| Scaffold_7 | 16310000 | 16319999 | green | ppa012835m | 16317212 | 16319158 | U497K_ARATH | Arabidopsis thaliana (Mouse-ear cress) UPF0497 membrane protein At3g50810 | Swiss-Prot |
| Scaffold_7 | 16310000 | 16319999 | lgreen | ppa012835m | 16317212 | 16319158 | U497K_ARATH | Arabidopsis thaliana (Mouse-ear cress) UPF0497 membrane protein At3g50810 | Swiss-Prot |
| Scaffold_7 | 16310000 | 16319999 | purple | ppa012835m | 16317212 | 16319158 | U497K_ARATH | Arabidopsis thaliana (Mouse-ear cress) UPF0497 membrane protein At3g50810 | Swiss-Prot |
| Scaffold_7 | 16370000 | 16379999 | blue | ppa003965m | 16377339 | 16380384 | AT3G50780.1 | protein match is: PRLI-interacting factor-related (TAIR:AT1G63850.1); Has 173 Blast hits to 173 proteins in 14 species: Archae - 0; Bacteria - 0; Metazoa - 8; Fungi - 0; Plants - 164; Viruses - 0; Other Eukaryotes - 1 (source: NCBI BLink). | TAIR |
| Scaffold_7 | 16370000 | 16379999 | dblue | ppa003965m | 16377339 | 16380384 | AT3G50780.1 | protein match is: PRLI-interacting factor-related (TAIR:AT1G63850.1); Has 173 Blast hits to 173 proteins in 14 species: Archae - 0; Bacteria - 0; Metazoa - 8; Fungi - 0; Plants - 164; Viruses - 0; Other Eukaryotes - 1 (source: NCBI BLink). | TAIR |
| Scaffold_7 | 16370000 | 16379999 | green | ppa003965m | 16377339 | 16380384 | AT3G50780.1 | protein match is: PRLI-interacting factor-related (TAIR:AT1G63850.1); Has 173 Blast hits to 173 proteins in 14 species: Archae - 0; Bacteria - 0; Metazoa - 8; Fungi - 0; Plants - 164; Viruses - 0; Other Eukaryotes - 1 (source: NCBI BLink). | TAIR |
| Scaffold_7 | 16370000 | 16379999 | lgreen | ppa003965m | 16377339 | 16380384 | AT3G50780.1 | protein match is: PRLI-interacting factor-related (TAIR:AT1G63850.1); Has 173 Blast hits to 173 proteins in 14 species: Archae - 0; Bacteria - 0; Metazoa - 8; Fungi - 0; Plants - 164; Viruses - 0; Other Eukaryotes - 1 (source: NCBI BLink). | TAIR |
| Scaffold_7 | 16380000 | 16389999 | blue | ppa001286m | 16387142 | 16392777 | BLH2_ARATH | Arabidopsis thaliana (Mouse-ear cress) BEL1-like homeodomain protein 2 | Swiss-Prot |
| Scaffold_7 | 16380000 | 16389999 | dblue | ppa001286m | 16387142 | 16392777 | BLH2_ARATH | Arabidopsis thaliana (Mouse-ear cress) BEL1-like homeodomain protein 2 | Swiss-Prot |
| Scaffold_7 | 16380000 | 16389999 | green | ppa001286m | 16387142 | 16392777 | BLH2_ARATH | Arabidopsis thaliana (Mouse-ear cress) BEL1-like homeodomain protein 2 | Swiss-Prot |
| Scaffold_7 | 16380000 | 16389999 | lgreen | ppa001286m | 16387142 | 16392777 | BLH2_ARATH | Arabidopsis thaliana (Mouse-ear cress) BEL1-like homeodomain protein 2 | Swiss-Prot |
| Scaffold_7 | 16410000 | 16419999 | blue | ppa002949m | 16419369 | 16421228 | GSO1_ARATH | Arabidopsis thaliana (Mouse-ear cress) LRR receptor-like serine/threonine-protein kinase GSO1 | Swiss-Prot |
| Scaffold_7 | 16410000 | 16419999 | dblue | ppa002949m | 16419369 | 16421228 | GSO1_ARATH | Arabidopsis thaliana (Mouse-ear cress) LRR receptor-like serine/threonine-protein kinase GSO1 | Swiss-Prot |
| Scaffold_7 | 16410000 | 16419999 | green | ppa002949m | 16419369 | 16421228 | GSO1_ARATH | Arabidopsis thaliana (Mouse-ear cress) LRR receptor-like serine/threonine-protein kinase GSO1 | Swiss-Prot |
| Scaffold_7 | 16410000 | 16419999 | lgreen | ppa002949m | 16419369 | 16421228 | GSO1_ARATH | Arabidopsis thaliana (Mouse-ear cress) LRR receptor-like serine/threonine-protein kinase GSO1 | Swiss-Prot |
| Scaffold_7 | 16410000 | 16419999 | purple | ppa002949m | 16419369 | 16421228 | GSO1_ARATH | Arabidopsis thaliana (Mouse-ear cress) LRR receptor-like serine/threonine-protein kinase GSO1 | Swiss-Prot |
| Scaffold_7 | 17070000 | 17079999 | blue | ppa023962m | 17078693 | 17082142 | SEC14_SCHPO | Schizosaccharomyces pombe (Fission yeast) Sec14 cytosolic factor | Swiss-Prot |
| Scaffold_7 | 17070000 | 17079999 | dblue | ppa023962m | 17078693 | 17082142 | SEC14_SCHPO | Schizosaccharomyces pombe (Fission yeast) Sec14 cytosolic factor | Swiss-Prot |
| Scaffold_7 | 17070000 | 17079999 | green | ppa023962m | 17078693 | 17082142 | SEC14_SCHPO | Schizosaccharomyces pombe (Fission yeast) Sec14 cytosolic factor | Swiss-Prot |
| Scaffold_7 | 17070000 | 17079999 | lgreen | ppa023962m | 17078693 | 17082142 | SEC14_SCHPO | Schizosaccharomyces pombe (Fission yeast) Sec14 cytosolic factor | Swiss-Prot |
| Scaffold_7 | 17070000 | 17079999 | purple | ppa023962m | 17078693 | 17082142 | SEC14_SCHPO | Schizosaccharomyces pombe (Fission yeast) Sec14 cytosolic factor | Swiss-Prot |
| Scaffold_7 | 17130000 | 17139999 | blue | ppa009997m | 17137970 | 17139156 | B8Y3J1_PYRPY | Pyrus pyrifolia (Japanese pear) (Pyrus serotina) Dehydrin | TrEMBL |
| Scaffold_7 | 17130000 | 17139999 | dblue | ppa009997m | 17137970 | 17139156 | B8Y3J1_PYRPY | Pyrus pyrifolia (Japanese pear) (Pyrus serotina) Dehydrin | TrEMBL |
| Scaffold_7 | 17130000 | 17139999 | green | ppa009997m | 17137970 | 17139156 | B8Y3J1_PYRPY | Pyrus pyrifolia (Japanese pear) (Pyrus serotina) Dehydrin | TrEMBL |
| Scaffold_7 | 17130000 | 17139999 | lgreen | ppa009997m | 17137970 | 17139156 | B8Y3J1_PYRPY | Pyrus pyrifolia (Japanese pear) (Pyrus serotina) Dehydrin | TrEMBL |
| Scaffold_7 | 17130000 | 17139999 | purple | ppa009997m | 17137970 | 17139156 | B8Y3J1_PYRPY | Pyrus pyrifolia (Japanese pear) (Pyrus serotina) Dehydrin | TrEMBL |
| Scaffold_7 | 17180000 | 17189999 | blue | ppa010766m | 17183284 | 17184910 | HYES_PIG | Sus scrofa (Pig) Epoxide hydrolase 2 | Swiss-Prot |
| Scaffold_7 | 17180000 | 17189999 | dblue | ppa010766m | 17183284 | 17184910 | HYES_PIG | Sus scrofa (Pig) Epoxide hydrolase 2 | Swiss-Prot |
| Scaffold_7 | 17180000 | 17189999 | green | ppa010766m | 17183284 | 17184910 | HYES_PIG | Sus scrofa (Pig) Epoxide hydrolase 2 | Swiss-Prot |
| Scaffold_7 | 17180000 | 17189999 | lgreen | ppa010766m | 17183284 | 17184910 | HYES_PIG | Sus scrofa (Pig) Epoxide hydrolase 2 | Swiss-Prot |
| Scaffold_7 | 17180000 | 17189999 | purple | ppa010766m | 17183284 | 17184910 | HYES_PIG | Sus scrofa (Pig) Epoxide hydrolase 2 | Swiss-Prot |
| Scaffold_7 | 17280000 | 17289999 | blue | ppa017480m | 17286201 | 17287044 | POL2_MOUSE | Mus musculus (Mouse) Retrovirus-related Pol polyprotein LINE-1 | Swiss-Prot |
| Scaffold_7 | 17280000 | 17289999 | dblue | ppa017480m | 17286201 | 17287044 | POL2_MOUSE | Mus musculus (Mouse) Retrovirus-related Pol polyprotein LINE-1 | Swiss-Prot |
| Scaffold_7 | 17280000 | 17289999 | green | ppa017480m | 17286201 | 17287044 | POL2_MOUSE | Mus musculus (Mouse) Retrovirus-related Pol polyprotein LINE-1 | Swiss-Prot |
| Scaffold_7 | 17280000 | 17289999 | lgreen | ppa017480m | 17286201 | 17287044 | POL2_MOUSE | Mus musculus (Mouse) Retrovirus-related Pol polyprotein LINE-1 | Swiss-Prot |
| Scaffold_7 | 17280000 | 17289999 | purple | ppa017480m | 17286201 | 17287044 | POL2_MOUSE | Mus musculus (Mouse) Retrovirus-related Pol polyprotein LINE-1 | Swiss-Prot |
| Scaffold_7 | 17340000 | 17349999 | blue | ppa000244m | 17346170 | 17353072 | C3H44_ARATH | Arabidopsis thaliana (Mouse-ear cress) Zinc finger CCCH domain-containing protein 44 | Swiss-Prot |
| Scaffold_7 | 17340000 | 17349999 | dblue | ppa000244m | 17346170 | 17353072 | C3H44_ARATH | Arabidopsis thaliana (Mouse-ear cress) Zinc finger CCCH domain-containing protein 44 | Swiss-Prot |
| Scaffold_7 | 17340000 | 17349999 | green | ppa000244m | 17346170 | 17353072 | C3H44_ARATH | Arabidopsis thaliana (Mouse-ear cress) Zinc finger CCCH domain-containing protein 44 | Swiss-Prot |
| Scaffold_7 | 17340000 | 17349999 | lgreen | ppa000244m | 17346170 | 17353072 | C3H44_ARATH | Arabidopsis thaliana (Mouse-ear cress) Zinc finger CCCH domain-containing protein 44 | Swiss-Prot |
| Scaffold_7 | 17340000 | 17349999 | purple | ppa000244m | 17346170 | 17353072 | C3H44_ARATH | Arabidopsis thaliana (Mouse-ear cress) Zinc finger CCCH domain-containing protein 44 | Swiss-Prot |
| Scaffold_7 | 17390000 | 17399999 | blue | ppa001146m | 17392779 | 17397937 | KIF4_DICDI | Dictyostelium discoideum (Slime mold) Kinesin-related protein 4 | Swiss-Prot |
| Scaffold_7 | 17390000 | 17399999 | dblue | ppa001146m | 17392779 | 17397937 | KIF4_DICDI | Dictyostelium discoideum (Slime mold) Kinesin-related protein 4 | Swiss-Prot |
| Scaffold_7 | 17390000 | 17399999 | green | ppa001146m | 17392779 | 17397937 | KIF4_DICDI | Dictyostelium discoideum (Slime mold) Kinesin-related protein 4 | Swiss-Prot |
| Scaffold_7 | 17390000 | 17399999 | lgreen | ppa001146m | 17392779 | 17397937 | KIF4_DICDI | Dictyostelium discoideum (Slime mold) Kinesin-related protein 4 | Swiss-Prot |
| Scaffold_7 | 17390000 | 17399999 | purple | ppa001146m | 17392779 | 17397937 | KIF4_DICDI | Dictyostelium discoideum (Slime mold) Kinesin-related protein 4 | Swiss-Prot |
| Scaffold_7 | 17790000 | 17799999 | blue | ppa004265m | 17799074 | 17802441 | ASPL1_ARATH | Arabidopsis thaliana (Mouse-ear cress) Aspartic proteinase-like protein 1 | Swiss-Prot |
| Scaffold_7 | 17790000 | 17799999 | dblue | ppa004265m | 17799074 | 17802441 | ASPL1_ARATH | Arabidopsis thaliana (Mouse-ear cress) Aspartic proteinase-like protein 1 | Swiss-Prot |
| Scaffold_7 | 17790000 | 17799999 | green | ppa004265m | 17799074 | 17802441 | ASPL1_ARATH | Arabidopsis thaliana (Mouse-ear cress) Aspartic proteinase-like protein 1 | Swiss-Prot |
| Scaffold_7 | 17790000 | 17799999 | lgreen | ppa004265m | 17799074 | 17802441 | ASPL1_ARATH | Arabidopsis thaliana (Mouse-ear cress) Aspartic proteinase-like protein 1 | Swiss-Prot |
| Scaffold_7 | 17820000 | 17829999 | blue | ppa018170m | 17827657 | 17830676 | AT1G21740.1 | Arabidopsis thaliana unknown protein | TAIR |
| Scaffold_7 | 17820000 | 17829999 | dblue | ppa018170m | 17827657 | 17830676 | AT1G21740.1 | Arabidopsis thaliana unknown protein | TAIR |
| Scaffold_7 | 17820000 | 17829999 | green | ppa018170m | 17827657 | 17830676 | AT1G21740.1 | Arabidopsis thaliana unknown protein | TAIR |
| Scaffold_7 | 17820000 | 17829999 | lgreen | ppa018170m | 17827657 | 17830676 | AT1G21740.1 | Arabidopsis thaliana unknown protein | TAIR |
| Scaffold_7 | 17820000 | 17829999 | purple | ppa018170m | 17827657 | 17830676 | AT1G21740.1 | Arabidopsis thaliana unknown protein | TAIR |
| Scaffold_7 | 17950000 | 17959999 | blue | ppa016932m | 17957037 | 17958620 | GDL87_ARATH | Arabidopsis thaliana (Mouse-ear cress) GDSL esterase/lipase At5g55050 | Swiss-Prot |
| Scaffold_7 | 17950000 | 17959999 | dblue | ppa016932m | 17957037 | 17958620 | GDL87_ARATH | Arabidopsis thaliana (Mouse-ear cress) GDSL esterase/lipase At5g55050 | Swiss-Prot |
| Scaffold_7 | 17950000 | 17959999 | green | ppa016932m | 17957037 | 17958620 | GDL87_ARATH | Arabidopsis thaliana (Mouse-ear cress) GDSL esterase/lipase At5g55050 | Swiss-Prot |
| Scaffold_7 | 17950000 | 17959999 | lgreen | ppa016932m | 17957037 | 17958620 | GDL87_ARATH | Arabidopsis thaliana (Mouse-ear cress) GDSL esterase/lipase At5g55050 | Swiss-Prot |
| Scaffold_7 | 17950000 | 17959999 | purple | ppa016932m | 17957037 | 17958620 | GDL87_ARATH | Arabidopsis thaliana (Mouse-ear cress) GDSL esterase/lipase At5g55050 | Swiss-Prot |
| Scaffold_7 | 18100000 | 18109999 | blue | ppb018959m | 18109439 | 18110369 | AT4G35280.1 | Arabidopsis thaliana zinc finger (C2H2 type) family protein | TAIR |
| Scaffold_7 | 18100000 | 18109999 | dblue | ppb018959m | 18109439 | 18110369 | AT4G35280.1 | Arabidopsis thaliana zinc finger (C2H2 type) family protein | TAIR |
| Scaffold_7 | 18100000 | 18109999 | green | ppb018959m | 18109439 | 18110369 | AT4G35280.1 | Arabidopsis thaliana zinc finger (C2H2 type) family protein | TAIR |
| Scaffold_7 | 18100000 | 18109999 | lgreen | ppb018959m | 18109439 | 18110369 | AT4G35280.1 | Arabidopsis thaliana zinc finger (C2H2 type) family protein | TAIR |
| Scaffold_7 | 18110000 | 18119999 | blue | ppa007586m | 18119432 | 18122116 | DDPS6_ARATH | Arabidopsis thaliana (Mouse-ear cress) Dehydrodolichyl diphosphate synthase 6 | Swiss-Prot |
| Scaffold_7 | 18110000 | 18119999 | dblue | ppa007586m | 18119432 | 18122116 | DDPS6_ARATH | Arabidopsis thaliana (Mouse-ear cress) Dehydrodolichyl diphosphate synthase 6 | Swiss-Prot |
| Scaffold_7 | 18110000 | 18119999 | green | ppa007586m | 18119432 | 18122116 | DDPS6_ARATH | Arabidopsis thaliana (Mouse-ear cress) Dehydrodolichyl diphosphate synthase 6 | Swiss-Prot |
| Scaffold_7 | 18110000 | 18119999 | lgreen | ppa007586m | 18119432 | 18122116 | DDPS6_ARATH | Arabidopsis thaliana (Mouse-ear cress) Dehydrodolichyl diphosphate synthase 6 | Swiss-Prot |
| Scaffold_7 | 18200000 | 18209999 | blue | ppa021400m | 18207083 | 18208637 | WOX8_ORYSJ | Oryza sativa subsp. japonica (Rice) WUSCHEL-related homeobox 8 | Swiss-Prot |
| Scaffold_7 | 18200000 | 18209999 | dblue | ppa021400m | 18207083 | 18208637 | WOX8_ORYSJ | Oryza sativa subsp. japonica (Rice) WUSCHEL-related homeobox 8 | Swiss-Prot |
| Scaffold_7 | 18200000 | 18209999 | green | ppa021400m | 18207083 | 18208637 | WOX8_ORYSJ | Oryza sativa subsp. japonica (Rice) WUSCHEL-related homeobox 8 | Swiss-Prot |
| Scaffold_7 | 18200000 | 18209999 | lgreen | ppa021400m | 18207083 | 18208637 | WOX8_ORYSJ | Oryza sativa subsp. japonica (Rice) WUSCHEL-related homeobox 8 | Swiss-Prot |
| Scaffold_7 | 18220000 | 18229999 | blue | ppa005746m | 18227804 | 18230820 | SRPK1_HUMAN | Homo sapiens (Human) Serine/threonine-protein kinase SRPK1 | Swiss-Prot |
| Scaffold_7 | 18220000 | 18229999 | dblue | ppa005746m | 18227804 | 18230820 | SRPK1_HUMAN | Homo sapiens (Human) Serine/threonine-protein kinase SRPK1 | Swiss-Prot |
| Scaffold_7 | 18220000 | 18229999 | green | ppa005746m | 18227804 | 18230820 | SRPK1_HUMAN | Homo sapiens (Human) Serine/threonine-protein kinase SRPK1 | Swiss-Prot |
| Scaffold_7 | 18220000 | 18229999 | lgreen | ppa005746m | 18227804 | 18230820 | SRPK1_HUMAN | Homo sapiens (Human) Serine/threonine-protein kinase SRPK1 | Swiss-Prot |
| Scaffold_7 | 18310000 | 18319999 | blue | ppa017100m | 18312300 | 18314092 | CRK35_ARATH | Arabidopsis thaliana (Mouse-ear cress) Putative cysteine-rich receptor-like protein kinase 35 | Swiss-Prot |
| Scaffold_7 | 18310000 | 18319999 | dblue | ppa017100m | 18312300 | 18314092 | CRK35_ARATH | Arabidopsis thaliana (Mouse-ear cress) Putative cysteine-rich receptor-like protein kinase 35 | Swiss-Prot |
| Scaffold_7 | 18310000 | 18319999 | green | ppa017100m | 18312300 | 18314092 | CRK35_ARATH | Arabidopsis thaliana (Mouse-ear cress) Putative cysteine-rich receptor-like protein kinase 35 | Swiss-Prot |
| Scaffold_7 | 18310000 | 18319999 | lgreen | ppa017100m | 18312300 | 18314092 | CRK35_ARATH | Arabidopsis thaliana (Mouse-ear cress) Putative cysteine-rich receptor-like protein kinase 35 | Swiss-Prot |
| Scaffold_7 | 19000000 | 19009999 | blue | ppa026438m | 19009611 | 19011268 | F3PH_PETHY | Petunia hybrida (Petunia) Flavonoid 3'-monooxygenase | Swiss-Prot |
| Scaffold_7 | 19000000 | 19009999 | dblue | ppa026438m | 19009611 | 19011268 | F3PH_PETHY | Petunia hybrida (Petunia) Flavonoid 3'-monooxygenase | Swiss-Prot |
| Scaffold_7 | 19000000 | 19009999 | green | ppa026438m | 19009611 | 19011268 | F3PH_PETHY | Petunia hybrida (Petunia) Flavonoid 3'-monooxygenase | Swiss-Prot |
| Scaffold_7 | 19000000 | 19009999 | lgreen | ppa026438m | 19009611 | 19011268 | F3PH_PETHY | Petunia hybrida (Petunia) Flavonoid 3'-monooxygenase | Swiss-Prot |
| Scaffold_7 | 19000000 | 19009999 | purple | ppa026438m | 19009611 | 19011268 | F3PH_PETHY | Petunia hybrida (Petunia) Flavonoid 3'-monooxygenase | Swiss-Prot |
| Scaffold_7 | 19010000 | 19019999 | blue | ppa022846m | 19019084 | 19020119 | PAT03_SOLTU | Solanum tuberosum (Potato) Patatin-03 | Swiss-Prot |
| Scaffold_7 | 19010000 | 19019999 | dblue | ppa022846m | 19019084 | 19020119 | PAT03_SOLTU | Solanum tuberosum (Potato) Patatin-03 | Swiss-Prot |
| Scaffold_7 | 19010000 | 19019999 | green | ppa022846m | 19019084 | 19020119 | PAT03_SOLTU | Solanum tuberosum (Potato) Patatin-03 | Swiss-Prot |
| Scaffold_7 | 19010000 | 19019999 | lgreen | ppa022846m | 19019084 | 19020119 | PAT03_SOLTU | Solanum tuberosum (Potato) Patatin-03 | Swiss-Prot |
| Scaffold_7 | 19020000 | 19029999 | blue | ppa023335m | 19026328 | 19028081 | F3PH_PETHY | Petunia hybrida (Petunia) Flavonoid 3'-monooxygenase | Swiss-Prot |
| Scaffold_7 | 19020000 | 19029999 | dblue | ppa023335m | 19026328 | 19028081 | F3PH_PETHY | Petunia hybrida (Petunia) Flavonoid 3'-monooxygenase | Swiss-Prot |
| Scaffold_7 | 19020000 | 19029999 | green | ppa023335m | 19026328 | 19028081 | F3PH_PETHY | Petunia hybrida (Petunia) Flavonoid 3'-monooxygenase | Swiss-Prot |
| Scaffold_7 | 19020000 | 19029999 | lgreen | ppa023335m | 19026328 | 19028081 | F3PH_PETHY | Petunia hybrida (Petunia) Flavonoid 3'-monooxygenase | Swiss-Prot |
| Scaffold_7 | 19020000 | 19029999 | purple | ppa023335m | 19026328 | 19028081 | F3PH_PETHY | Petunia hybrida (Petunia) Flavonoid 3'-monooxygenase | Swiss-Prot |
| Scaffold_7 | 20670000 | 20679999 | blue | ppa022590m | 20678438 | 20679358 | AT5G19650.1 | Arabidopsis thaliana OFP8 (ARABIDOPSIS THALIANA OVATE FAMILY PROTEIN 8) | TAIR |
| Scaffold_7 | 20670000 | 20679999 | dblue | ppa022590m | 20678438 | 20679358 | AT5G19650.1 | Arabidopsis thaliana OFP8 (ARABIDOPSIS THALIANA OVATE FAMILY PROTEIN 8) | TAIR |
| Scaffold_7 | 20670000 | 20679999 | green | ppa022590m | 20678438 | 20679358 | AT5G19650.1 | Arabidopsis thaliana OFP8 (ARABIDOPSIS THALIANA OVATE FAMILY PROTEIN 8) | TAIR |
| Scaffold_7 | 20670000 | 20679999 | lgreen | ppa022590m | 20678438 | 20679358 | AT5G19650.1 | Arabidopsis thaliana OFP8 (ARABIDOPSIS THALIANA OVATE FAMILY PROTEIN 8) | TAIR |
| Scaffold_8 | 360000 | 369999 | blue | ppa008342m | 368172 | 369629 | EXT1C_DANRE | Danio rerio (Zebrafish) (Brachydanio rerio) Exostosin-1c | Swiss-Prot |
| Scaffold_8 | 360000 | 369999 | dblue | ppa008342m | 368172 | 369629 | EXT1C_DANRE | Danio rerio (Zebrafish) (Brachydanio rerio) Exostosin-1c | Swiss-Prot |
| Scaffold_8 | 360000 | 369999 | green | ppa008342m | 368172 | 369629 | EXT1C_DANRE | Danio rerio (Zebrafish) (Brachydanio rerio) Exostosin-1c | Swiss-Prot |
| Scaffold_8 | 360000 | 369999 | lgreen | ppa008342m | 368172 | 369629 | EXT1C_DANRE | Danio rerio (Zebrafish) (Brachydanio rerio) Exostosin-1c | Swiss-Prot |
| Scaffold_8 | 360000 | 369999 | purple | ppa008342m | 368172 | 369629 | EXT1C_DANRE | Danio rerio (Zebrafish) (Brachydanio rerio) Exostosin-1c | Swiss-Prot |
| Scaffold_8 | 1320000 | 1329999 | blue | ppa026764m | 1328670 | 1331578 | Q7F966_ORYSJ | Oryza sativa subsp. japonica (Rice) OSJNBa0091C07.2 protein | TrEMBL |
| Scaffold_8 | 1320000 | 1329999 | dblue | ppa026764m | 1328670 | 1331578 | Q7F966_ORYSJ | Oryza sativa subsp. japonica (Rice) OSJNBa0091C07.2 protein | TrEMBL |
| Scaffold_8 | 1320000 | 1329999 | green | ppa026764m | 1328670 | 1331578 | Q7F966_ORYSJ | Oryza sativa subsp. japonica (Rice) OSJNBa0091C07.2 protein | TrEMBL |
| Scaffold_8 | 1320000 | 1329999 | lgreen | ppa026764m | 1328670 | 1331578 | Q7F966_ORYSJ | Oryza sativa subsp. japonica (Rice) OSJNBa0091C07.2 protein | TrEMBL |
| Scaffold_8 | 1320000 | 1329999 | purple | ppa026764m | 1328670 | 1331578 | Q7F966_ORYSJ | Oryza sativa subsp. japonica (Rice) OSJNBa0091C07.2 protein | TrEMBL |
| Scaffold_8 | 1480000 | 1489999 | blue | ppa015377m | 1488497 | 1489705 | CRR38_ARATH | Arabidopsis thaliana (Mouse-ear cress) Cysteine-rich repeat secretory protein 38 | Swiss-Prot |
| Scaffold_8 | 1480000 | 1489999 | dblue | ppa015377m | 1488497 | 1489705 | CRR38_ARATH | Arabidopsis thaliana (Mouse-ear cress) Cysteine-rich repeat secretory protein 38 | Swiss-Prot |
| Scaffold_8 | 1480000 | 1489999 | green | ppa015377m | 1488497 | 1489705 | CRR38_ARATH | Arabidopsis thaliana (Mouse-ear cress) Cysteine-rich repeat secretory protein 38 | Swiss-Prot |
| Scaffold_8 | 1480000 | 1489999 | lgreen | ppa015377m | 1488497 | 1489705 | CRR38_ARATH | Arabidopsis thaliana (Mouse-ear cress) Cysteine-rich repeat secretory protein 38 | Swiss-Prot |
| Scaffold_8 | 1480000 | 1489999 | purple | ppa015377m | 1488497 | 1489705 | CRR38_ARATH | Arabidopsis thaliana (Mouse-ear cress) Cysteine-rich repeat secretory protein 38 | Swiss-Prot |
| Scaffold_8 | 1790000 | 1799999 | blue | ppa013550m | 1799651 | 1800512 | N/A | N/A | N/A |
| Scaffold_8 | 1790000 | 1799999 | dblue | ppa013550m | 1799651 | 1800512 | N/A | N/A | N/A |
| Scaffold_8 | 1790000 | 1799999 | green | ppa013550m | 1799651 | 1800512 | N/A | N/A | N/A |
| Scaffold_8 | 1790000 | 1799999 | lgreen | ppa013550m | 1799651 | 1800512 | N/A | N/A | N/A |
| Scaffold_8 | 2070000 | 2079999 | blue | ppa015430m | 2072389 | 2076289 | TMVRN_NICGU | Nicotiana glutinosa (Tobacco) TMV resistance protein N | Swiss-Prot |
| Scaffold_8 | 2070000 | 2079999 | dblue | ppa015430m | 2072389 | 2076289 | TMVRN_NICGU | Nicotiana glutinosa (Tobacco) TMV resistance protein N | Swiss-Prot |
| Scaffold_8 | 2070000 | 2079999 | green | ppa015430m | 2072389 | 2076289 | TMVRN_NICGU | Nicotiana glutinosa (Tobacco) TMV resistance protein N | Swiss-Prot |
| Scaffold_8 | 2070000 | 2079999 | lgreen | ppa015430m | 2072389 | 2076289 | TMVRN_NICGU | Nicotiana glutinosa (Tobacco) TMV resistance protein N | Swiss-Prot |
| Scaffold_8 | 7350000 | 7359999 | blue | ppa026222m | 7351860 | 7353871 | AT5G38720.1 | Arabidopsis thaliana unknown protein | TAIR |
| Scaffold_8 | 7350000 | 7359999 | dblue | ppa026222m | 7351860 | 7353871 | AT5G38720.1 | Arabidopsis thaliana unknown protein | TAIR |
| Scaffold_8 | 7350000 | 7359999 | green | ppa026222m | 7351860 | 7353871 | AT5G38720.1 | Arabidopsis thaliana unknown protein | TAIR |
| Scaffold_8 | 7350000 | 7359999 | lgreen | ppa026222m | 7351860 | 7353871 | AT5G38720.1 | Arabidopsis thaliana unknown protein | TAIR |
| Scaffold_8 | 7370000 | 7379999 | blue | ppa024736m | 7379503 | 7379852 | MAD57_ORYSJ | Oryza sativa subsp. japonica (Rice) MADS-box transcription factor 57 | Swiss-Prot |
| Scaffold_8 | 7370000 | 7379999 | dblue | ppa024736m | 7379503 | 7379852 | MAD57_ORYSJ | Oryza sativa subsp. japonica (Rice) MADS-box transcription factor 57 | Swiss-Prot |
| Scaffold_8 | 7370000 | 7379999 | green | ppa024736m | 7379503 | 7379852 | MAD57_ORYSJ | Oryza sativa subsp. japonica (Rice) MADS-box transcription factor 57 | Swiss-Prot |
| Scaffold_8 | 7370000 | 7379999 | lgreen | ppa024736m | 7379503 | 7379852 | MAD57_ORYSJ | Oryza sativa subsp. japonica (Rice) MADS-box transcription factor 57 | Swiss-Prot |
| Scaffold_8 | 7840000 | 7849999 | blue | ppa019021m | 7846371 | 7849542 | Y4862_ARATH | Arabidopsis thaliana (Mouse-ear cress) Uncharacterized protein At4g38062 | Swiss-Prot |
| Scaffold_8 | 7840000 | 7849999 | dblue | ppa019021m | 7846371 | 7849542 | Y4862_ARATH | Arabidopsis thaliana (Mouse-ear cress) Uncharacterized protein At4g38062 | Swiss-Prot |
| Scaffold_8 | 7840000 | 7849999 | green | ppa019021m | 7846371 | 7849542 | Y4862_ARATH | Arabidopsis thaliana (Mouse-ear cress) Uncharacterized protein At4g38062 | Swiss-Prot |
| Scaffold_8 | 7840000 | 7849999 | lgreen | ppa019021m | 7846371 | 7849542 | Y4862_ARATH | Arabidopsis thaliana (Mouse-ear cress) Uncharacterized protein At4g38062 | Swiss-Prot |
| Scaffold_8 | 8720000 | 8729999 | blue | ppa023175m | 8728011 | 8728346 | COGT2_ARATH | Arabidopsis thaliana (Mouse-ear cress) Cytokinin-O-glucosyltransferase 2 | Swiss-Prot |
| Scaffold_8 | 8720000 | 8729999 | dblue | ppa023175m | 8728011 | 8728346 | COGT2_ARATH | Arabidopsis thaliana (Mouse-ear cress) Cytokinin-O-glucosyltransferase 2 | Swiss-Prot |
| Scaffold_8 | 8720000 | 8729999 | green | ppa023175m | 8728011 | 8728346 | COGT2_ARATH | Arabidopsis thaliana (Mouse-ear cress) Cytokinin-O-glucosyltransferase 2 | Swiss-Prot |
| Scaffold_8 | 8720000 | 8729999 | lgreen | ppa023175m | 8728011 | 8728346 | COGT2_ARATH | Arabidopsis thaliana (Mouse-ear cress) Cytokinin-O-glucosyltransferase 2 | Swiss-Prot |
| Scaffold_8 | 8720000 | 8729999 | purple | ppa023175m | 8728011 | 8728346 | COGT2_ARATH | Arabidopsis thaliana (Mouse-ear cress) Cytokinin-O-glucosyltransferase 2 | Swiss-Prot |
| Scaffold_8 | 9150000 | 9159999 | blue | ppa002469m | 9159460 | 9172313 | AT5G64170.2 | Arabidopsis thaliana dentin sialophosphoprotein-related | TAIR |
| Scaffold_8 | 9150000 | 9159999 | dblue | ppa002469m | 9159460 | 9172313 | AT5G64170.2 | Arabidopsis thaliana dentin sialophosphoprotein-related | TAIR |
| Scaffold_8 | 9150000 | 9159999 | green | ppa002469m | 9159460 | 9172313 | AT5G64170.2 | Arabidopsis thaliana dentin sialophosphoprotein-related | TAIR |
| Scaffold_8 | 9150000 | 9159999 | lgreen | ppa002469m | 9159460 | 9172313 | AT5G64170.2 | Arabidopsis thaliana dentin sialophosphoprotein-related | TAIR |
| Scaffold_8 | 10030000 | 10039999 | blue | ppa022617m | 10038657 | 10042261 | A5BU47_VITVI | Vitis vinifera (Grape) Putative uncharacterized protein | TrEMBL |
| Scaffold_8 | 10030000 | 10039999 | dblue | ppa022617m | 10038657 | 10042261 | A5BU47_VITVI | Vitis vinifera (Grape) Putative uncharacterized protein | TrEMBL |
| Scaffold_8 | 10030000 | 10039999 | green | ppa022617m | 10038657 | 10042261 | A5BU47_VITVI | Vitis vinifera (Grape) Putative uncharacterized protein | TrEMBL |
| Scaffold_8 | 10030000 | 10039999 | lgreen | ppa022617m | 10038657 | 10042261 | A5BU47_VITVI | Vitis vinifera (Grape) Putative uncharacterized protein | TrEMBL |
| Scaffold_8 | 10030000 | 10039999 | purple | ppa022617m | 10038657 | 10042261 | A5BU47_VITVI | Vitis vinifera (Grape) Putative uncharacterized protein | TrEMBL |
| Scaffold_8 | 10750000 | 10759999 | blue | ppa009920m | 10757487 | 10760361 | AF9_ASHGO | Ashbya gossypii (Yeast) (Eremothecium gossypii) Protein AF-9 homolog | Swiss-Prot |
| Scaffold_8 | 10750000 | 10759999 | dblue | ppa009920m | 10757487 | 10760361 | AF9_ASHGO | Ashbya gossypii (Yeast) (Eremothecium gossypii) Protein AF-9 homolog | Swiss-Prot |
| Scaffold_8 | 10750000 | 10759999 | green | ppa009920m | 10757487 | 10760361 | AF9_ASHGO | Ashbya gossypii (Yeast) (Eremothecium gossypii) Protein AF-9 homolog | Swiss-Prot |
| Scaffold_8 | 10750000 | 10759999 | lgreen | ppa009920m | 10757487 | 10760361 | AF9_ASHGO | Ashbya gossypii (Yeast) (Eremothecium gossypii) Protein AF-9 homolog | Swiss-Prot |
| Scaffold_8 | 12160000 | 12169999 | blue | ppa013928m | 12167597 | 12170664 | AT4G39300.1 | Arabidopsis thaliana unknown protein | TAIR |
| Scaffold_8 | 12160000 | 12169999 | dblue | ppa013928m | 12167597 | 12170664 | AT4G39300.1 | Arabidopsis thaliana unknown protein | TAIR |
| Scaffold_8 | 12160000 | 12169999 | green | ppa013928m | 12167597 | 12170664 | AT4G39300.1 | Arabidopsis thaliana unknown protein | TAIR |
| Scaffold_8 | 12160000 | 12169999 | lgreen | ppa013928m | 12167597 | 12170664 | AT4G39300.1 | Arabidopsis thaliana unknown protein | TAIR |
| Scaffold_8 | 12370000 | 12379999 | blue | ppa018397m | 12378730 | 12379855 | BURP3_ORYSJ | Oryza sativa subsp. japonica (Rice) BURP domain-containing protein 3 | Swiss-Prot |
| Scaffold_8 | 12370000 | 12379999 | dblue | ppa018397m | 12378730 | 12379855 | BURP3_ORYSJ | Oryza sativa subsp. japonica (Rice) BURP domain-containing protein 3 | Swiss-Prot |
| Scaffold_8 | 12370000 | 12379999 | green | ppa018397m | 12378730 | 12379855 | BURP3_ORYSJ | Oryza sativa subsp. japonica (Rice) BURP domain-containing protein 3 | Swiss-Prot |
| Scaffold_8 | 12370000 | 12379999 | lgreen | ppa018397m | 12378730 | 12379855 | BURP3_ORYSJ | Oryza sativa subsp. japonica (Rice) BURP domain-containing protein 3 | Swiss-Prot |
| Scaffold_8 | 12370000 | 12379999 | purple | ppa018397m | 12378730 | 12379855 | BURP3_ORYSJ | Oryza sativa subsp. japonica (Rice) BURP domain-containing protein 3 | Swiss-Prot |
| Scaffold_8 | 12550000 | 12559999 | blue | ppa007633m | 12555738 | 12558431 | PLT5_ARATH | Arabidopsis thaliana Polyol transporter 5 | Swiss-Prot |
| Scaffold_8 | 12550000 | 12559999 | dblue | ppa007633m | 12555738 | 12558431 | PLT5_ARATH | Arabidopsis thaliana Polyol transporter 5 | Swiss-Prot |
| Scaffold_8 | 12550000 | 12559999 | green | ppa007633m | 12555738 | 12558431 | PLT5_ARATH | Arabidopsis thaliana Polyol transporter 5 | Swiss-Prot |
| Scaffold_8 | 12550000 | 12559999 | lgreen | ppa007633m | 12555738 | 12558431 | PLT5_ARATH | Arabidopsis thaliana Polyol transporter 5 | Swiss-Prot |
| Scaffold_8 | 12550000 | 12559999 | purple | ppa007633m | 12555738 | 12558431 | PLT5_ARATH | Arabidopsis thaliana Polyol transporter 5 | Swiss-Prot |
| Scaffold_8 | 13530000 | 13539999 | blue | ppa010900m | 13539487 | 13541206 | ARGC_ORYSJ | Oryza sativa subsp. japonica (Rice) "Probable N-acetyl-gamma-glutamyl-phosphate reductase, chloroplastic" | Swiss-Prot |
| Scaffold_8 | 13530000 | 13539999 | dblue | ppa010900m | 13539487 | 13541206 | ARGC_ORYSJ | Oryza sativa subsp. japonica (Rice) "Probable N-acetyl-gamma-glutamyl-phosphate reductase, chloroplastic" | Swiss-Prot |
| Scaffold_8 | 13530000 | 13539999 | green | ppa010900m | 13539487 | 13541206 | ARGC_ORYSJ | Oryza sativa subsp. japonica (Rice) "Probable N-acetyl-gamma-glutamyl-phosphate reductase, chloroplastic" | Swiss-Prot |
| Scaffold_8 | 13530000 | 13539999 | lgreen | ppa010900m | 13539487 | 13541206 | ARGC_ORYSJ | Oryza sativa subsp. japonica (Rice) "Probable N-acetyl-gamma-glutamyl-phosphate reductase, chloroplastic" | Swiss-Prot |
| Scaffold_8 | 13530000 | 13539999 | purple | ppa010900m | 13539487 | 13541206 | ARGC_ORYSJ | Oryza sativa subsp. japonica (Rice) "Probable N-acetyl-gamma-glutamyl-phosphate reductase, chloroplastic" | Swiss-Prot |
| Scaffold_8 | 14450000 | 14459999 | blue | ppa012998m | 14457662 | 14458359 | RS16_GOSHI | Gossypium hirsutum (Upland cotton) (Gossypium mexicanum) 40S ribosomal protein S16 | Swiss-Prot |
| Scaffold_8 | 14450000 | 14459999 | dblue | ppa012998m | 14457662 | 14458359 | RS16_GOSHI | Gossypium hirsutum (Upland cotton) (Gossypium mexicanum) 40S ribosomal protein S16 | Swiss-Prot |
| Scaffold_8 | 14450000 | 14459999 | green | ppa012998m | 14457662 | 14458359 | RS16_GOSHI | Gossypium hirsutum (Upland cotton) (Gossypium mexicanum) 40S ribosomal protein S16 | Swiss-Prot |
| Scaffold_8 | 14450000 | 14459999 | lgreen | ppa012998m | 14457662 | 14458359 | RS16_GOSHI | Gossypium hirsutum (Upland cotton) (Gossypium mexicanum) 40S ribosomal protein S16 | Swiss-Prot |
| Scaffold_8 | 14450000 | 14459999 | purple | ppa012998m | 14457662 | 14458359 | RS16_GOSHI | Gossypium hirsutum (Upland cotton) (Gossypium mexicanum) 40S ribosomal protein S16 | Swiss-Prot |
| Scaffold_8 | 15700000 | 15709999 | blue | ppa000024m | 15705658 | 15729749 | TOR_DICDI | Dictyostelium discoideum (Slime mold) Target of rapamycin | Swiss-Prot |
| Scaffold_8 | 15700000 | 15709999 | dblue | ppa000024m | 15705658 | 15729749 | TOR_DICDI | Dictyostelium discoideum (Slime mold) Target of rapamycin | Swiss-Prot |
| Scaffold_8 | 15700000 | 15709999 | green | ppa000024m | 15705658 | 15729749 | TOR_DICDI | Dictyostelium discoideum (Slime mold) Target of rapamycin | Swiss-Prot |
| Scaffold_8 | 15700000 | 15709999 | lgreen | ppa000024m | 15705658 | 15729749 | TOR_DICDI | Dictyostelium discoideum (Slime mold) Target of rapamycin | Swiss-Prot |
| Scaffold_8 | 15700000 | 15709999 | purple | ppa000024m | 15705658 | 15729749 | TOR_DICDI | Dictyostelium discoideum (Slime mold) Target of rapamycin | Swiss-Prot |
| Scaffold_8 | 19630000 | 19639999 | blue | ppa019939m | 19632251 | 19632996 | LBD29_ARATH | Arabidopsis thaliana (Mouse-ear cress) LOB domain-containing protein 29 | Swiss-Prot |
| Scaffold_8 | 19630000 | 19639999 | dblue | ppa019939m | 19632251 | 19632996 | LBD29_ARATH | Arabidopsis thaliana (Mouse-ear cress) LOB domain-containing protein 29 | Swiss-Prot |
| Scaffold_8 | 19630000 | 19639999 | green | ppa019939m | 19632251 | 19632996 | LBD29_ARATH | Arabidopsis thaliana (Mouse-ear cress) LOB domain-containing protein 29 | Swiss-Prot |
| Scaffold_8 | 19630000 | 19639999 | lgreen | ppa019939m | 19632251 | 19632996 | LBD29_ARATH | Arabidopsis thaliana (Mouse-ear cress) LOB domain-containing protein 29 | Swiss-Prot |
| Scaffold_8 | 19630000 | 19639999 | purple | ppa019939m | 19632251 | 19632996 | LBD29_ARATH | Arabidopsis thaliana (Mouse-ear cress) LOB domain-containing protein 29 | Swiss-Prot |
| Scaffold_8 | 19960000 | 19969999 | blue | ppa001772m | 19967605 | 19971038 | AT3G63430.1 | Arabidopsis thaliana unknown protein | TAIR |
| Scaffold_8 | 19960000 | 19969999 | dblue | ppa001772m | 19967605 | 19971038 | AT3G63430.1 | Arabidopsis thaliana unknown protein | TAIR |
| Scaffold_8 | 19960000 | 19969999 | green | ppa001772m | 19967605 | 19971038 | AT3G63430.1 | Arabidopsis thaliana unknown protein | TAIR |
| Scaffold_8 | 19960000 | 19969999 | lgreen | ppa001772m | 19967605 | 19971038 | AT3G63430.1 | Arabidopsis thaliana unknown protein | TAIR |
| Scaffold_8 | 19960000 | 19969999 | purple | ppa001772m | 19967605 | 19971038 | AT3G63430.1 | Arabidopsis thaliana unknown protein | TAIR |
| Scaffold_8 | 21290000 | 21299999 | blue | ppa014148m | 21298781 | 21301362 | AT2G47690.1 | Arabidopsis thaliana NADH-ubiquinone oxidoreductase-related | TAIR |
| Scaffold_8 | 21290000 | 21299999 | dblue | ppa014148m | 21298781 | 21301362 | AT2G47690.1 | Arabidopsis thaliana NADH-ubiquinone oxidoreductase-related | TAIR |
| Scaffold_8 | 21290000 | 21299999 | green | ppa014148m | 21298781 | 21301362 | AT2G47690.1 | Arabidopsis thaliana NADH-ubiquinone oxidoreductase-related | TAIR |
| Scaffold_8 | 21290000 | 21299999 | lgreen | ppa014148m | 21298781 | 21301362 | AT2G47690.1 | Arabidopsis thaliana NADH-ubiquinone oxidoreductase-related | TAIR |
| Scaffold_8 | 21290000 | 21299999 | purple | ppa014148m | 21298781 | 21301362 | AT2G47690.1 | Arabidopsis thaliana NADH-ubiquinone oxidoreductase-related | TAIR |
| Scaffold_8 | 21550000 | 21559999 | blue | ppa005900m | 21557222 | 21560142 | VTA1_BOVIN | Bos taurus (Bovine) Vacuolar protein sorting-associated protein VTA1 homolog | Swiss-Prot |
| Scaffold_8 | 21550000 | 21559999 | dblue | ppa005900m | 21557222 | 21560142 | VTA1_BOVIN | Bos taurus (Bovine) Vacuolar protein sorting-associated protein VTA1 homolog | Swiss-Prot |
| Scaffold_8 | 21550000 | 21559999 | green | ppa005900m | 21557222 | 21560142 | VTA1_BOVIN | Bos taurus (Bovine) Vacuolar protein sorting-associated protein VTA1 homolog | Swiss-Prot |
| Scaffold_8 | 21550000 | 21559999 | lgreen | ppa005900m | 21557222 | 21560142 | VTA1_BOVIN | Bos taurus (Bovine) Vacuolar protein sorting-associated protein VTA1 homolog | Swiss-Prot |
| Scaffold_8 | 21550000 | 21559999 | purple | ppa005900m | 21557222 | 21560142 | VTA1_BOVIN | Bos taurus (Bovine) Vacuolar protein sorting-associated protein VTA1 homolog | Swiss-Prot |
| Scaffold_rest | 580000 | 589999 | blue | ppa016390m | 589479 | 591136 | GOX1_ARATH | Arabidopsis thaliana (Mouse-ear cress) Probable peroxisomal (S)-2-hydroxy-acid oxidase 1 | Swiss-Prot |
| Scaffold_rest | 580000 | 589999 | dblue | ppa016390m | 589479 | 591136 | GOX1_ARATH | Arabidopsis thaliana (Mouse-ear cress) Probable peroxisomal (S)-2-hydroxy-acid oxidase 1 | Swiss-Prot |
| Scaffold_rest | 580000 | 589999 | green | ppa016390m | 589479 | 591136 | GOX1_ARATH | Arabidopsis thaliana (Mouse-ear cress) Probable peroxisomal (S)-2-hydroxy-acid oxidase 1 | Swiss-Prot |
| Scaffold_rest | 580000 | 589999 | lgreen | ppa016390m | 589479 | 591136 | GOX1_ARATH | Arabidopsis thaliana (Mouse-ear cress) Probable peroxisomal (S)-2-hydroxy-acid oxidase 1 | Swiss-Prot |
| Scaffold_rest | 580000 | 589999 | purple | ppa016390m | 589479 | 591136 | GOX1_ARATH | Arabidopsis thaliana (Mouse-ear cress) Probable peroxisomal (S)-2-hydroxy-acid oxidase 1 | Swiss-Prot |
| Scaffold_rest | 1140000 | 1149999 | blue | ppa015033m | 293037 | 295590 | YG31B_YEAST | Saccharomyces cerevisiae (Baker's yeast) Transposon Ty3-G Gag-Pol polyprotein | Swiss-Prot |
| Scaffold_rest | 1140000 | 1149999 | dblue | ppa015033m | 293037 | 295590 | YG31B_YEAST | Saccharomyces cerevisiae (Baker's yeast) Transposon Ty3-G Gag-Pol polyprotein | Swiss-Prot |
| Scaffold_rest | 1140000 | 1149999 | green | ppa015033m | 293037 | 295590 | YG31B_YEAST | Saccharomyces cerevisiae (Baker's yeast) Transposon Ty3-G Gag-Pol polyprotein | Swiss-Prot |
| Scaffold_rest | 1140000 | 1149999 | lgreen | ppa015033m | 293037 | 295590 | YG31B_YEAST | Saccharomyces cerevisiae (Baker's yeast) Transposon Ty3-G Gag-Pol polyprotein | Swiss-Prot |
| Scaffold_rest | 1150000 | 1159999 | blue | ppa014346m | 307203 | 308201 | N/A | N/A | N/A |
| Scaffold_rest | 1150000 | 1159999 | dblue | ppa014346m | 307203 | 308201 | N/A | N/A | N/A |
| Scaffold_rest | 1150000 | 1159999 | green | ppa014346m | 307203 | 308201 | N/A | N/A | N/A |
| Scaffold_rest | 1150000 | 1159999 | lgreen | ppa014346m | 307203 | 308201 | N/A | N/A | N/A |
| Scaffold_rest | 1170000 | 1179999 | blue | ppa020344m | 322847 | 323491 | A5AWJ4_VITVI | Vitis vinifera (Grape) Putative uncharacterized protein | TrEMBL |
| Scaffold_rest | 1170000 | 1179999 | dblue | ppa020344m | 322847 | 323491 | A5AWJ4_VITVI | Vitis vinifera (Grape) Putative uncharacterized protein | TrEMBL |
| Scaffold_rest | 1170000 | 1179999 | green | ppa020344m | 322847 | 323491 | A5AWJ4_VITVI | Vitis vinifera (Grape) Putative uncharacterized protein | TrEMBL |
| Scaffold_rest | 1170000 | 1179999 | lgreen | ppa020344m | 322847 | 323491 | A5AWJ4_VITVI | Vitis vinifera (Grape) Putative uncharacterized protein | TrEMBL |
| Scaffold_rest | 1210000 | 1219999 | blue | ppa026621m | 363148 | 363852 | AT1G71970.1 | Arabidopsis thaliana unknown protein | TAIR |
| Scaffold_rest | 1210000 | 1219999 | dblue | ppa026621m | 363148 | 363852 | AT1G71970.1 | Arabidopsis thaliana unknown protein | TAIR |
| Scaffold_rest | 1210000 | 1219999 | green | ppa026621m | 363148 | 363852 | AT1G71970.1 | Arabidopsis thaliana unknown protein | TAIR |
| Scaffold_rest | 1210000 | 1219999 | lgreen | ppa026621m | 363148 | 363852 | AT1G71970.1 | Arabidopsis thaliana unknown protein | TAIR |
| Scaffold_rest | 1430000 | 1439999 | blue | ppa014169m | 586486 | 587239 | N/A | N/A | N/A |
| Scaffold_rest | 1430000 | 1439999 | dblue | ppa014169m | 586486 | 587239 | N/A | N/A | N/A |
| Scaffold_rest | 1430000 | 1439999 | green | ppa014169m | 586486 | 587239 | N/A | N/A | N/A |
| Scaffold_rest | 1430000 | 1439999 | lgreen | ppa014169m | 586486 | 587239 | N/A | N/A | N/A |
| Scaffold_rest | 1450000 | 1459999 | blue | ppb013297m | 604719 | 605063 | N/A | N/A | N/A |
| Scaffold_rest | 1450000 | 1459999 | dblue | ppb013297m | 604719 | 605063 | N/A | N/A | N/A |
| Scaffold_rest | 1450000 | 1459999 | green | ppb013297m | 604719 | 605063 | N/A | N/A | N/A |
| Scaffold_rest | 1450000 | 1459999 | lgreen | ppb013297m | 604719 | 605063 | N/A | N/A | N/A |
| Scaffold_rest | 1450000 | 1459999 | purple | ppb013297m | 604719 | 605063 | N/A | N/A | N/A |

**Supplementary Table 9c**. The statistics of the density of the related genes in whole genome and regions under selection.

# means the number of the related genes

| Function Annotation | The classification of biological process | Whole genome # | # / Mbp (ρ1) | Region under ornamental selection # | # / Mbp (ρ2) | ρ2 / ρ1 | Region under edible selection # | # / Mbp (ρ3) | ρ3 / ρ1 |
| --- | --- | --- | --- | --- | --- | --- | --- | --- | --- |
| Transcription factor EDM2 | Ⅰ (Flower development) | 1 | 0.004 | 1 | 0.382 | 85.73 | 0 | / | / |
| Transcriptional corepressor SEUSS | Ⅰ (Flower development) | 2 | 0.009 | 1 | 0.382 | 42.86 | 0 | / | / |
| Flowering time control protein | Ⅰ (Flower development) | 6 | 0.027 | 1 | 0.382 | 14.29 | 0 | / | / |
| Mandelonitrile lyase | Ⅱ (Flavonoid biosynthesis) | 7 | 0.031 | 1 | 0.382 | 12.25 | 0 | / | / |
| Anthocyanin 5-aromatic acyltransferase | Ⅱ (Flavonoid biosynthesis) | 18 | 0.080 | 2 | 0.763 | 9.53 | 0 | / | / |
| Caffeic acid 3-O-methyltransferase | Ⅱ (Flavonoid biosynthesis) | 14 | 0.062 | 1 | 0.382 | 6.12 | 1 | 0.680 | 10.91 |
| Protein TRANSPARENT TESTA | Ⅱ (Flavonoid biosynthesis) | 45 | 0.200 | 3 | 1.145 | 5.72 | 0 | / | / |
| Photosystem I reaction center subunit | Ⅳ (Photosynthesis) | 7 | 0.031 | 1 | 0.382 | 12.25 | 0 | / | / |
| Peptidyl-prolyl isomerase | Ⅴ (Cell division) | 8 | 0.036 | 1 | 0.382 | 10.72 | 0 | / | / |
| RING-box protein | Ⅴ (Cell division) | 2 | 0.009 | 2 | 0.763 | 85.73 | 0 | / | / |
| SKP1-like protein | Ⅴ (Cell division) | 16 | 0.071 | 1 | 0.382 | 5.36 | 0 | / | / |
| Wall-associated receptor kinase 2 | Ⅴ (Cell division) | 7 | 0.031 | 4 | 1.527 | 48.99 | 1 | 0.680 | 21.83 |
| LEA protein-related | Ⅵ (Response to stress) | 13 | 0.058 | 2 | 0.763 | 13.19 | 0 | / | / |
| Putative germin-like protein | Ⅵ (Response to stress) | 8 | 0.036 | 2 | 0.763 | 21.43 | 0 | / | / |
| Isoamylase | Ⅲ (Carbohydrate metabolism) | 2 | 0.009 | 1 | 0.382 | 42.86 | 0 | / | / |
| Alpha-glucosidase | Ⅲ (Carbohydrate metabolism) | 4 | 0.018 | 1 | 0.382 | 21.43 | 0 | / | / |
| Cytokinin-O-glucosyltransferase 2 | Ⅲ (Carbohydrate metabolism) | 60 | 0.267 | 1 | 0.382 | 1.43 | 4 | 2.721 | 10.19 |
| Epoxide hydrolase 2 | Ⅲ (Carbohydrate metabolism) | 13 | 0.058 | 0 | / | / | 2 | 1.361 | 23.51 |
| Flavonoid 3'-monooxygenase | Ⅱ (Flavonoid biosynthesis) | 16 | 0.071 | 0 | / | / | 2 | 1.361 | 19.10 |
| Retrovirus-related Pol polyprotein LINE-1 | Ⅶ (RNA-dependent DNA replication) | 9 | 0.040 | 0 | / | / | 2 | 1.361 | 33.95 |
| 6-phosphogluconolactonase | Ⅲ (Carbohydrate metabolism) | 2 | 0.009 | 0 | / | / | 1 | 0.680 | 76.40 |
| D-sorbitol-6-phosphate dehydrogenase | Ⅲ (Carbohydrate metabolism) | 2 | 0.009 | 0 | / | / | 1 | 0.680 | 76.40 |
| Alpha-glucan water dikinase | Ⅲ (Carbohydrate metabolism) | 2 | 0.009 | 0 | / | / | 1 | 0.680 | 76.40 |
| Chlorophyll a-b binding protein | Ⅳ (Photosynthesis) | 19 | 0.085 | 0 | / | / | 1 | 0.680 | 8.04 |
| Electron carrier | Ⅳ (Photosynthesis) | 13 | 0.058 | 0 | / | / | 1 | 0.680 | 11.75 |
| Expansin | Ⅴ (Cell division) | 22 | 0.098 | 0 | / | / | 1 | 0.680 | 6.95 |
| Cell division cycle-associated | Ⅴ (Cell division) | 1 | 0.004 | 0 | / | / | 1 | 0.680 | 152.80 |
| Dihydrolipoyllysine-residue succinyltransferase | Ⅷ (Tricarboxylic acid cycle) | 2 | 0.009 | 0 | / | / | 1 | 0.680 | 76.40 |

#### Table S10. The ratio of the heterozygous SNPs in each group/subgroup of peach.

| **Groups** | **Wild** | **Ornamental** | **Edible** | | | | | | **Total Cultivated** | **Total** |
| --- | --- | --- | --- | --- | --- | --- | --- | --- | --- | --- |
|  |  |  | B | C | D | E | F | Total |  |  |
| **Ratio of heterozygote SNPs in group** | 3.126% | 0.499% | 1.256% | 1.006% | 0.921% | 0.968% | 1.192% | 1.094% | 1.021% | 1.272% |
| **Ratio of heterozygote SNPs in group (modified)** | 3.770% | 0.670% | 1.540% | 1.249% | 1.111% | 1.210% | 1.472% | 1.332% | 1.252% | 1.552% |

**Table S11.** The ratio of the average heterozygous SNPs in wild group vs. cultivated group in five plants.

| **Resquencing Projects of different plants** | **The ratio of heterozygosity between wild group and cultivated group (Wild : Cultivated)** |
| --- | --- |
| **Apple** | 0.926~0.951 : 1 |
| **Cherry** | 0.985~1.032 : 1 |
| **Rice** | 1.200 : 1 |
| **Soybean** | 1.552 : 1 |
| **Peach** | 3.061 : 1 |
| **Peach (modified)** | 3.012 : 1 |

#### Table S12. The influencing factors upon heterozygosity in different plants.

| **Influencing factors upon heterozygosity** | | **Apple, Cherry** | **Rice, Soybean** | **Peach** |
| --- | --- | --- | --- | --- |
| **Mating system** | Self compatibility |  | √ | √ |
|  | Self incompatibility | √ |  |  |
| **Mode of reproduction** | Grafting | √ |  | √ |
|  | Seedling |  | √ |  |
| **Life span** | Short |  | √ |  |
|  | Long | √ |  | √ |
| **Population size** | Small | √ |  | √ |
|  | Large |  | √ |  |
