# Supplementary material for: Comparative population genomics reveals the domestication history of the peach, Prunus persica, and human influences on perennial fruit crops
Source: Genome Biol. 2014 Jul 31;15(7):415. doi: 10.1186/s13059-014-0415-1 (PMC4174323; doi:10.1186/s13059-014-0415-1)
Supplement: Additional file 2: Figure S1. — Geographic distribution of the 84 peach accessions. Figure S2. Relationship between the identified SNPs and the sample sizes. Figure S3. Relationship between the called SNPs and the sequencing depth. Figure S4. Total depth of all genotype sites according to SNPs in the 84 samples. Figure S5. SNP depth of each sample (L09, L13, W18, and L34). Figure S6. Details of the mapping results in SNP sites. Figure S7. SNP depth distributions of samples without SNPs in repeat regions or homologous sequences. Figure S8. Depth of homozygous (n = 0) and heterozygous sites (n > 0) in the genotype. Figure S9. Two supposed models for explaining why the depth of heterozygous SNP sites was higher than the depth of homozygous SNP sites. Figure S10. Relationship between missed genotype ratio and sequencing depth in heterozygotes and homozygotes by data fitting. Figure S11. Venn diagram of the unique and common SNPs in three groups. Figure S12. The maximum-likelihood tree and the neighbor-joining tree of the 84 peach accessions. Figure S13. Principal component analysis of wild, ornamental, and edible peaches. Figure S14. Population structure of 84 peach accessions by FRAPPE. Figure S15. The selection judgment outline of the 'region under selection', based on population structure. Figure S16. ROD and Fst values in the regions under edible and ornamental selection. Figure S17. R (resistance) genes and the genes under selection in the chromosomes. Figure S18. Gene Ontology analysis of the genes under ornamental selection. Figure S19. Gene Ontology analysis of the genes under edible selection. Figure S20. Linkage disequilibrium decay rates in different groups and subgroups. Figure S21. Linkage disequilibrium analysis of two regions under selection. Figure S22. Genome-wide association studies of the flesh adhesion trait. [file 13059_2014_415_MOESM2_ESM.docx]

#### Figure S1. Geographic distribution of the 84 peach accessions.


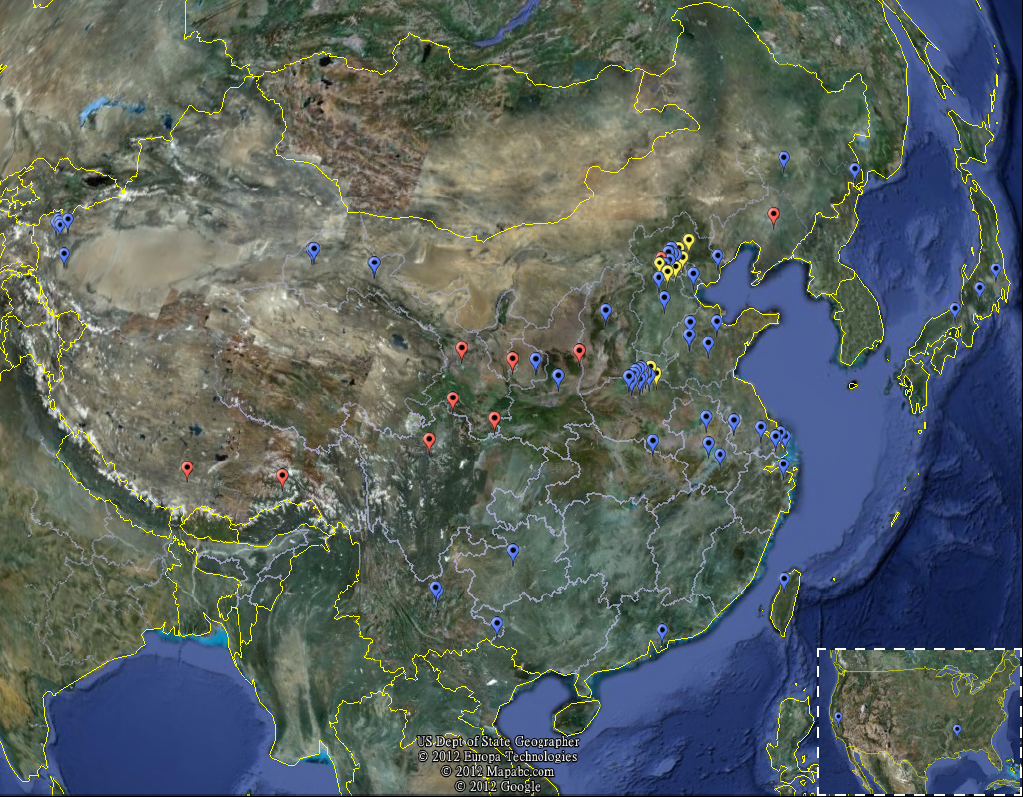


The majority of the 84 samples were collected in the peach’s area of origin, China, distributed among latitudes 22.5°N~52.5°N. Two accessions were collected from the United States and three were from Japan. The three different colors represent three different groups (red, wild; yellow, ornamental; blue, edible). This map was produced using Google Earth.

#### Figure S2. Relationship between the identified single-nucleotide polymorphisms (SNPs) and the sample sizes.


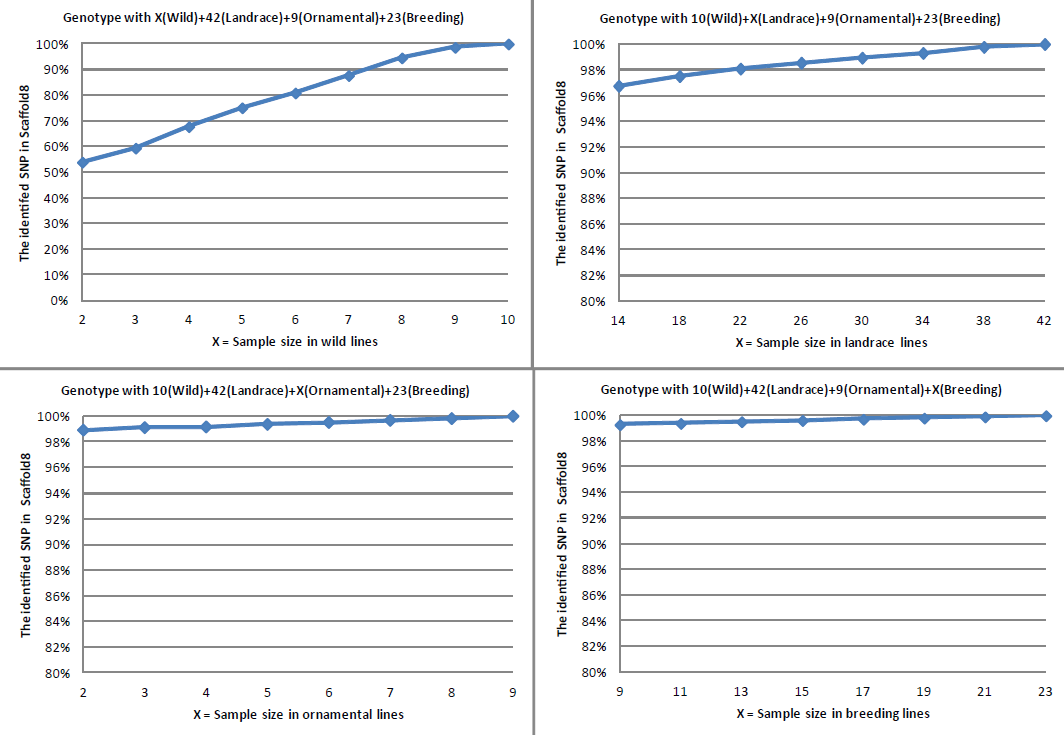


We analyzed the relationship between the identified SNPs in the genotype and the sample sizes in each group (statistics from Scaffold 8). We found that the increase in the sample size in wild lines greatly aided the identification of SNPs. However, the rate of increase slowed for sample sizes > 8. The contributions from cultivated lines such as ornamental, landrace, and breeding lines were weak, but these lines contained many ecotypes and various phenotypes. Considering that a sufficient number of SNPs and phenotypes existed in different lines and in three main groups (wild, ornamental, and edible), we concluded that the sample sizes of 10, 9, 42, and 23 in wild, ornamental, landrace, and breeding lines, respectively, were suitable.

#### Figure S3. Relationship between the called single-nucleotide polymorphisms (SNPs) and the sequencing depth.

We performed sequencing of two samples (L11 and B65) to the 7× depth and used stepped-up reads with different depths (1×, 2×, 3×…) to call SNPs to analyze the relationship between the called SNPs and the sequencing depth. The Y-axis is the ratio of the called SNPs in sample L11 vs. the whole genotype, which contained 84 samples; the X-axis is the sequencing depth of the reads in sample L11 from 1× to 7×. The graph shows the growth trend of the called SNPs with as sequencing depth increases; the ratio of called SNPs vs. the whole genotype reached 82.9% by a depth of 3×, after which growth slowed down.

#### Figure S4. Total depth of all genotype sites according to single-nucleotide polymorphisms (SNPs) in the 84 samples.


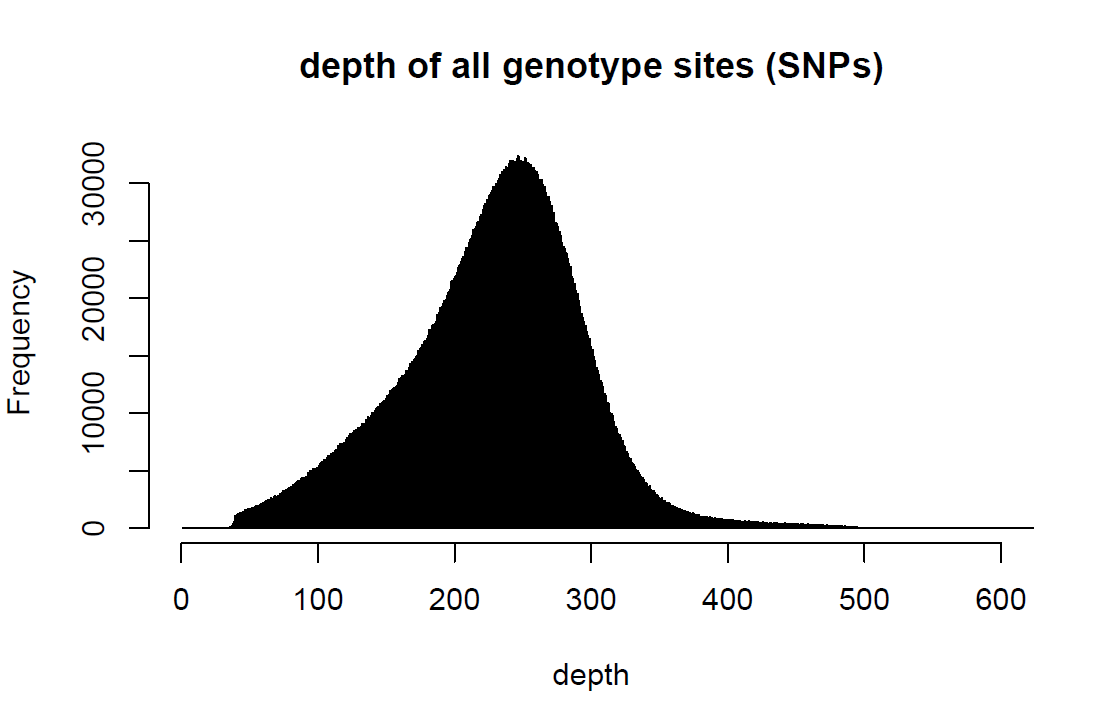


We performed a statistical analysis of the total depth of all genotype sites (or called population SNPs) in the 84 samples. The pick depth was around 250× (~3× × 84 = 252×). Although some sites from some samples were missing, the total depth of the sites was still high enough to conduct population SNP calling.

#### Figure S5. Single-nucleotide polymorphism (SNP) depth in each sample (L09, L13, W18, and L34).


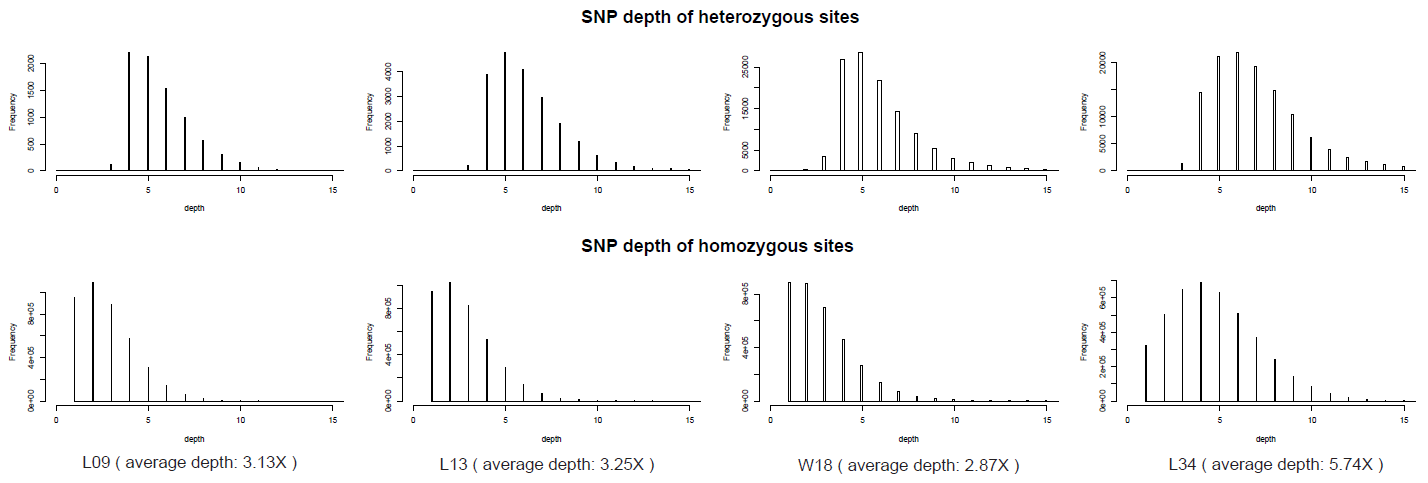


We performed statistical analysis of the SNP depth in each sample. These graphs display the SNP depth distributions of heterozygous and homozygous sites in four samples (L09, L13, W18, and L34). The four samples had different average depths in the genome (L09: 3.13×, L13: 3.25×, W18: 2.87×, L34: 5.74×). However, the SNP depth of the heterozygous and homozygous sites differed from the average depth in the whole genome. The peak SNP depth (2~4×) of homozygous sites was a little smaller than the average depth in whole genome, whereas the peak SNP depth (4~6×) of heterozygous sites was a little higher than the average depth in the whole genome.

#### Figure S6. Details of the mapping results in single-nucleotide polymorphism (SNP) sites.

1. Examples of homozygous SNPs:

Sample: B69, SNP position: Scaffold 2 (2,014,579 bp)


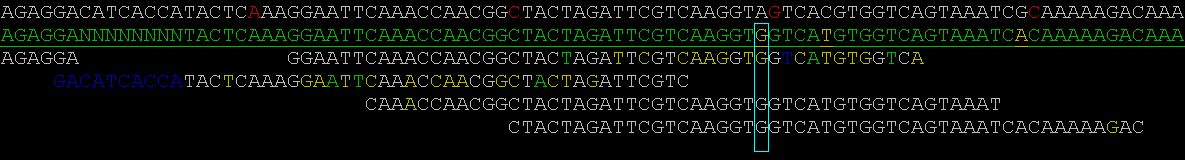
**Reference**

**Genotype**

**Mapping Reads**

**(bases)**


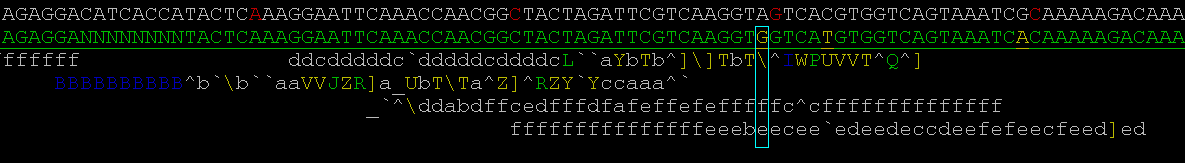
**Reference**

**Genotype**

**Mapping Reads**

**(Quality values)**

Sample: L11, SNP position: Scaffold 2 (2,014,579 bp)


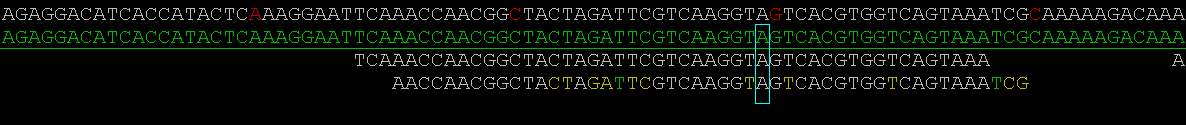
**Reference**

**Genotype**

**Mapping Reads**

**(bases)**


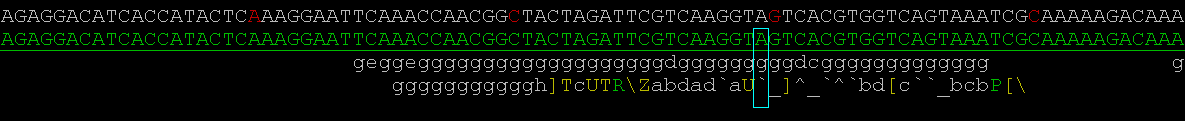
**Reference**

**Genotype**

**Mapping Reads**

**(Quality values)**

1. Example of heterozygous SNP:

Sample: O62, SNP position: Scaffold 4 (5,063,466 bp)


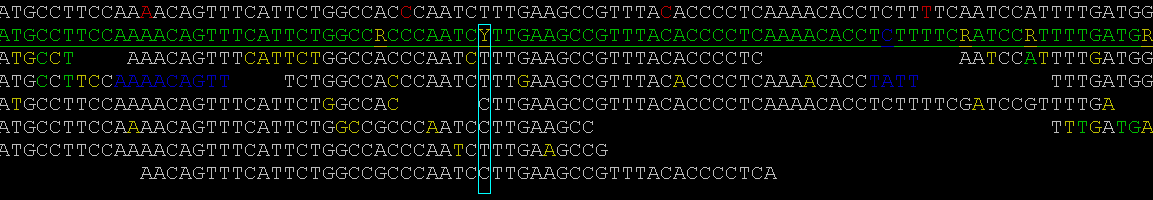
**Reference**

**Genotype**

**Mapping Reads**

**(bases)**


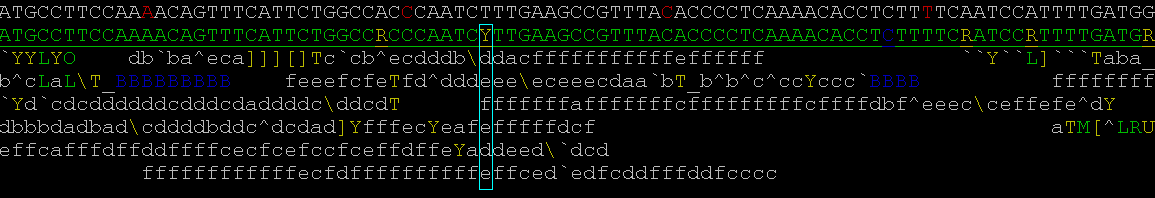
**Reference**

**Genotype**

**Mapping Reads**

**(Quality values)**

These figures present the details of the mapping results in homozygous and heterozygous SNP sites. All the reads used for SNP calling were unique mapping reads that could map only once at one location in the genome. For homozygous SNPs (a), sometimes the mapping depth was as low as 2×, but the mapping reads had very high quality values in the SNP sites. For heterozygous SNPs (b), the actual mapping depths were much higher than expected; the majority of them were 4×, 5×, 6×, or higher). These findings provided significant guarantees of the quality of our results of SNP calling.

#### Figure S7. Single-nucleotide polymorphism (SNP) depth distributions of samples without SNPs in repeat regions or homologous sequences.

| 1. Without SNPs in repeat regions:   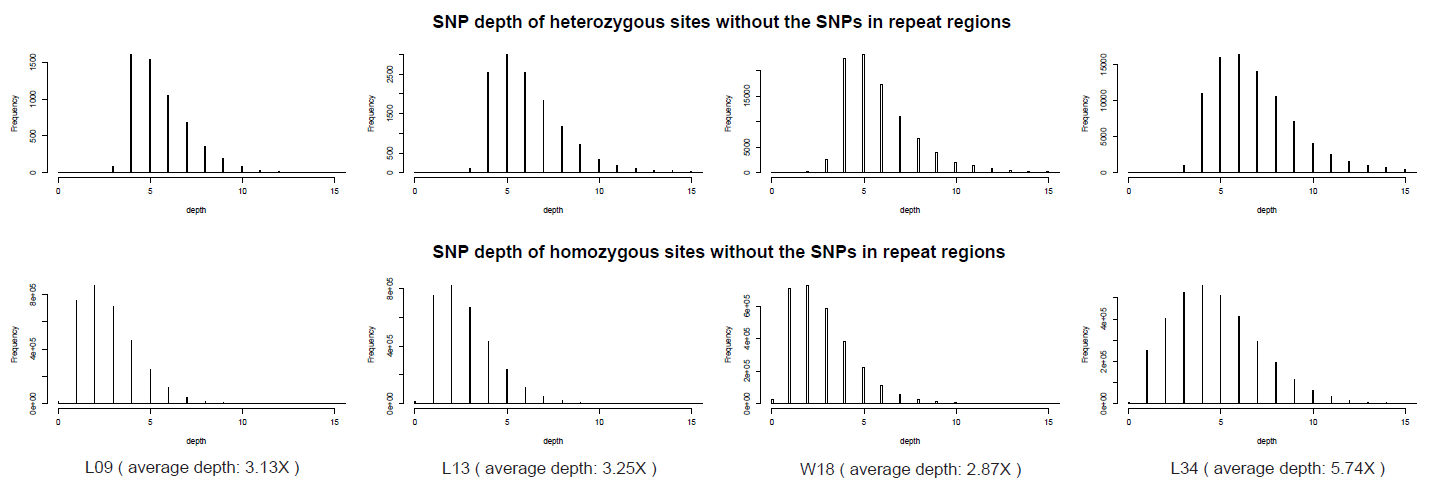 |
| --- |
| 1. Without SNPs in repeat regions or homologous sequences:   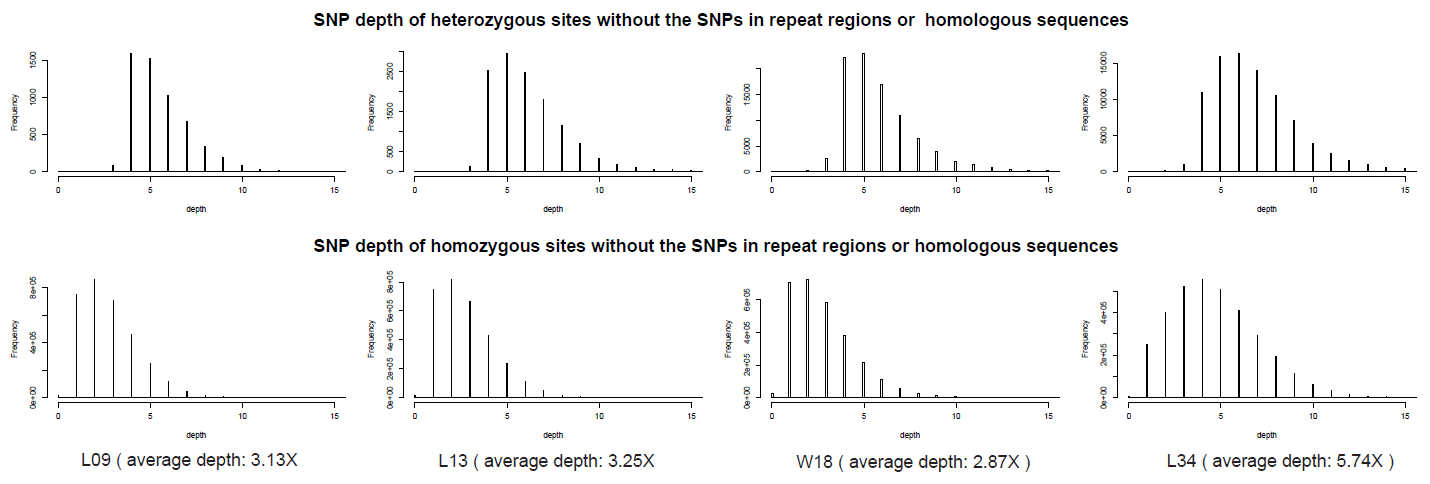 |

(a) We filtered the SNPs in repeat regions and recalculated the statistics. The central peaks of the SNP depth of heterozygous sites were around 4~6×, and the central peaks of the SNP depth of homozygous sites were around 2~4×. (b) We filtered the SNPs in repeat regions and in homologous sequences and recalculated the statistics. The distributions were similar; the homologous sequences we defined here comprised the two or more sequences of the reference genome that were the same for the majority of the sequences and had only a few different bases (fewer than 5%).

####
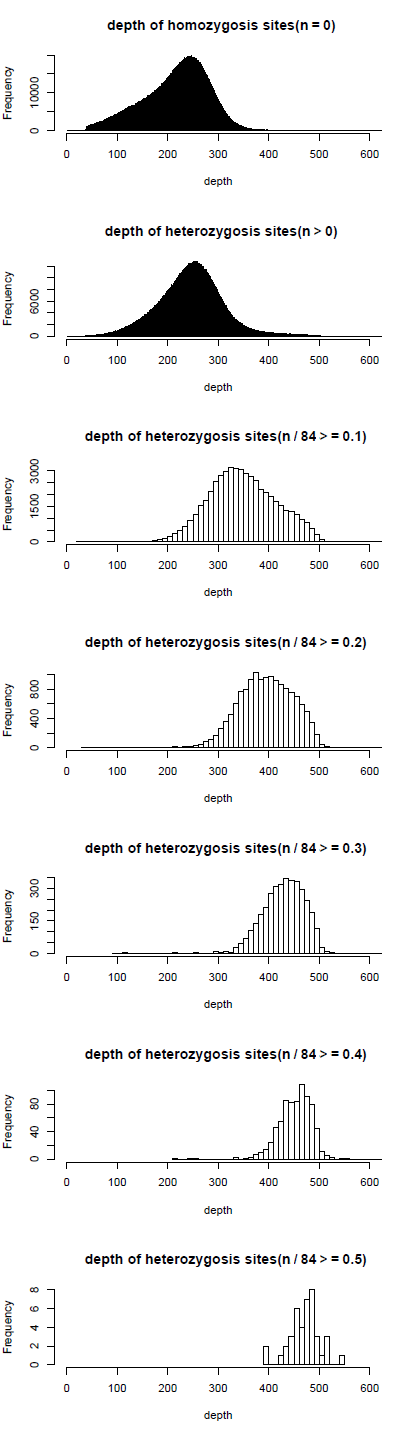
Figure S8. Depth of homozygosis (n = 0) and heterozygosis sites (n > 0) in the genotype.

In the total genotype of 84 samples, not all the sites in all samples were homozygous or heterozygous. Some sites contained homozygotes or data were missing, so we called them homozygosis sites (n = 0); some sites contained heterozygotes in at least one sample, and we called them heterozygosis sites (n > 0). Here “n” was the number of samples with heterozygotes in the site. As shown in the figure, sites with higher heterozygote frequency in the population also had higher total depth.

#### Figure S9. Two supposed models for explaining why the depth of heterozygous SNP sites was higher than the depth of homozygous SNP sites.


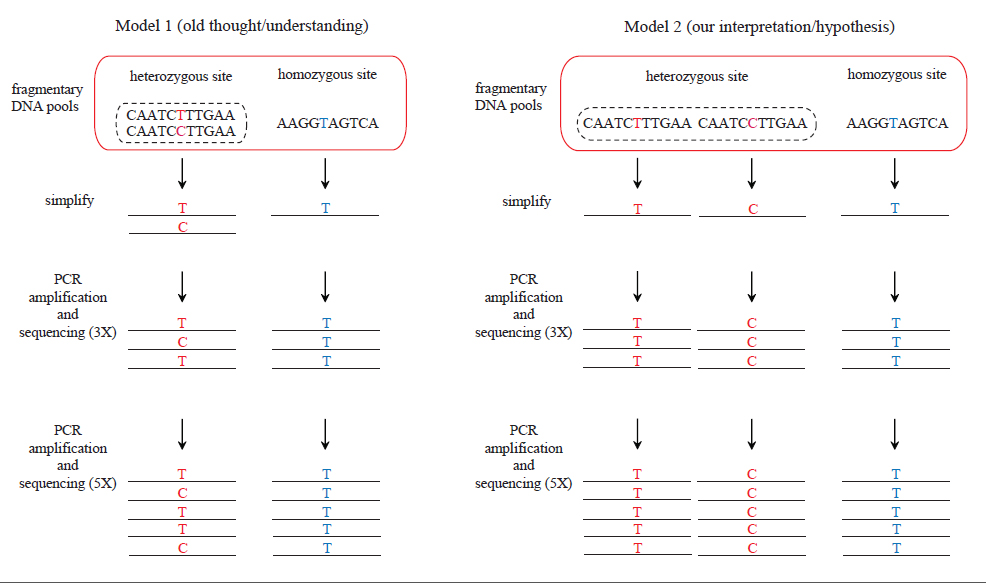


The model 1 is old thought or previous understanding, the model 2 is our interpretation or hypothesis. We believe that both of the models should exist in actual sequencing process. Model 2 could be the reason why the depth of heterozygous SNP sites was higher than the depth of homozygous SNP sites.

#### Figure S10. Relationship between missed genotype ratio and sequencing depth in heterozygotes and homozygotes by data fitting.


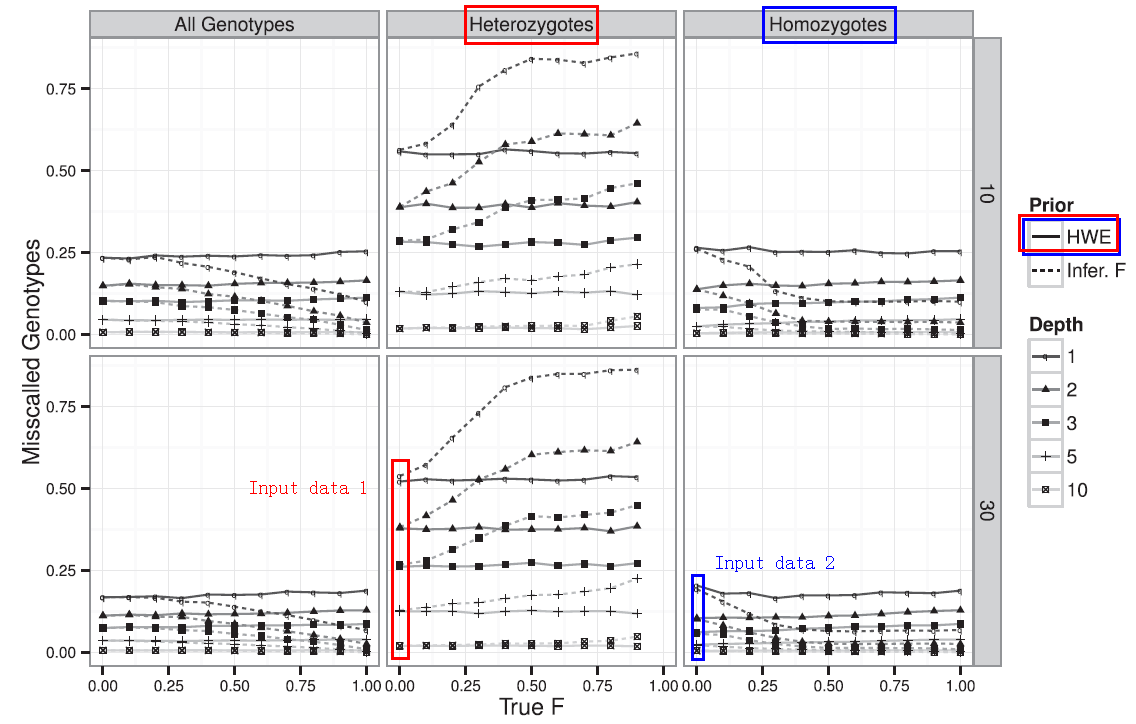
In general, if sequencing depth was higher, the missed genotype (SNPs) would be lower. In order to find the relationship between missed genotype ratio and the sequencing depth, we chose simulated data from Figure 2 of Vieira et al. [12] (shown here), and made a Logistic Fit by OriginLab.


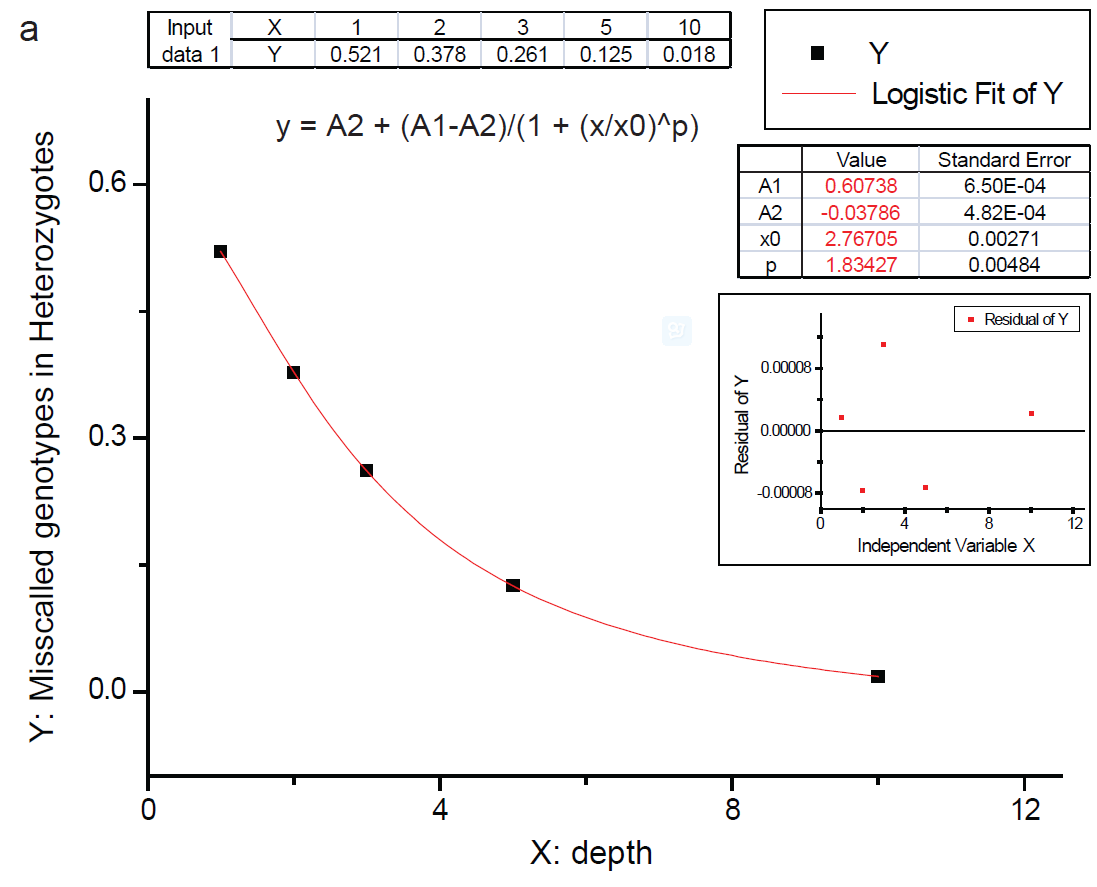
Figure (a) was the fitting result of the missed genotype ratio in heterozygotes and the depth. The Input data 1 were chosen and delineated in the red box (heterozygotes, Prior = HWE, True F = 0.00). The best-fitting mathematical relationship between miscalled genotypes in heterozygotes (y) and the depth (x) is

$$y=-0.03786+\frac{0.60738+0.03786}{1+{(x/2.76705)}^{1.83427}}$$

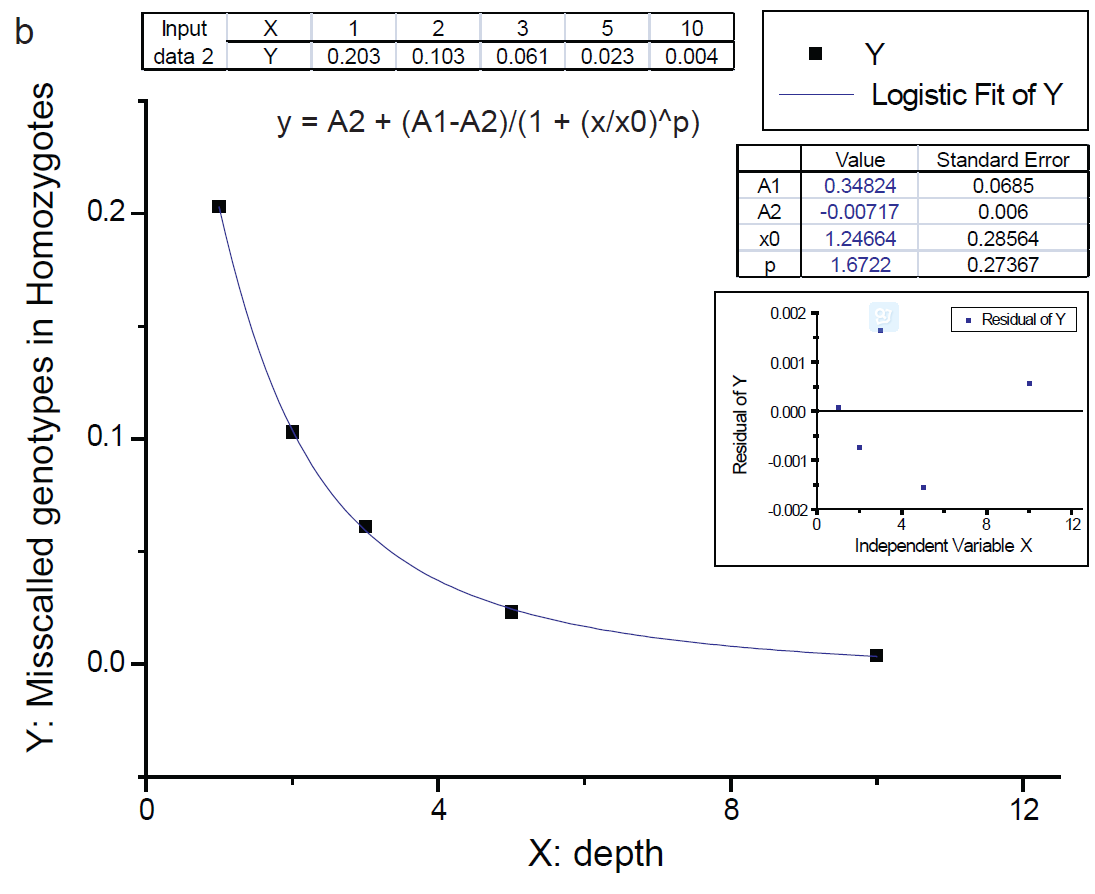
Figure (b) was the fitting result of the missed genotype ratio in homozygotes and the depth. Input data 2 were chosen and delineated in the blue box (Homozygotes, Prior = HWE, True F = 0.00). The best-fitting mathematical relationship between miscalled genotypes in homozygotes (y) and the depth (x) is

$$y=-0.00717+\frac{0.34824+0.00717}{1+{(x/1.24664)}^{0.27367}}$$

#### Figure S11. Venn diagram of the unique and common single-nucleotide polymorphisms (SNPs) in three groups.


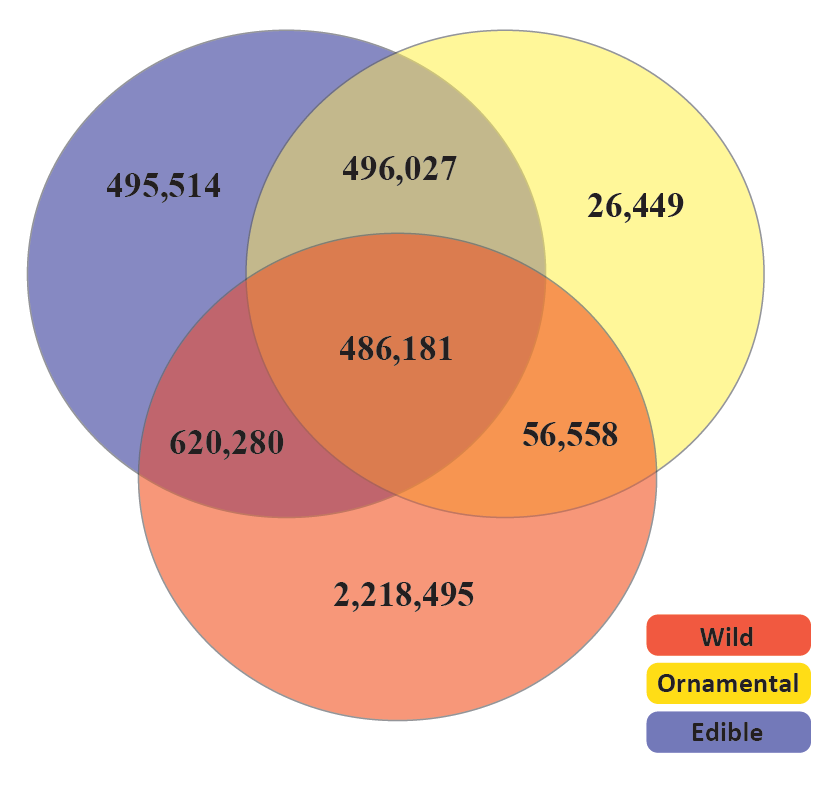


The quantities of the unique SNPs in wild, ornamental, and edible groups were 2,218,495, 26,449, and 495,514, respectively. The number of SNPs that occurred in all three groups was 486,181. The quantities of the SNPs shared in common in two groups were 496,027 (edible and ornamental), 620,280 (edible and wild), and 56,558 (ornamental and wild). In other words, 50.95% of the SNPs in ornamental peach and 52.74% of the SNPs in edible peach are found in wild accessions, indicating that the cultivated groups underwent a long domestication history and the wild group could provide useful genetic resources for peach improvement in the future.

#### Figure S12. The maximum-likelihood tree and the neighbor-joining tree of the 84 peach accessions.


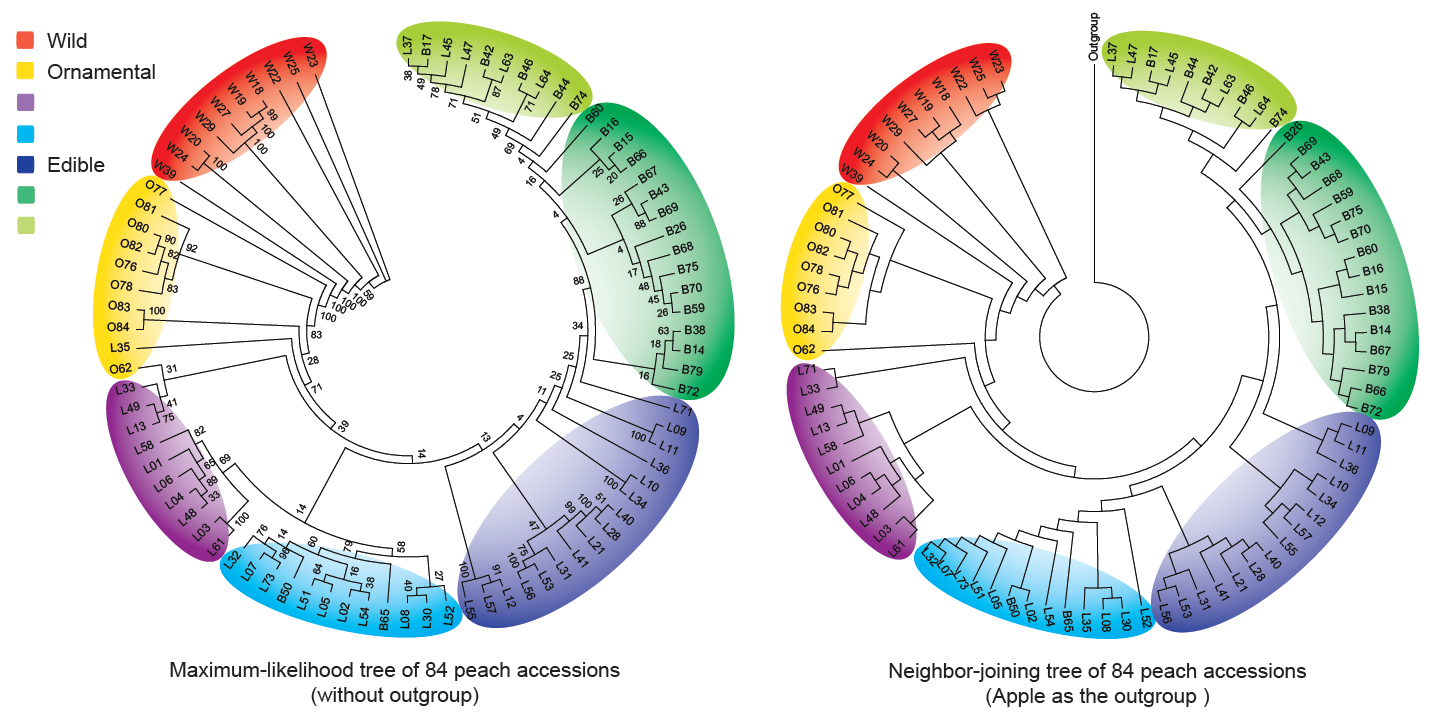


The left-hand figure is the maximum-likelihood tree of the 84 peach accessions; the right-hand figure is the neighbor-joining tree of the 84 peach accessions. The accessions colored red are wild peaches, those colored yellow are ornamental peaches, and those colored purple, blue, dark blue, green, and light green are edible peaches. Among these, the majority of the accessions colored blue and dark blue are landraces, and the majority of the accessions colored green and light green are breeding lines. Some of the details of the branches are different; however, majority of the topology and the main branches of the two trees are similar, although they were constructed with different algorithms.

#### Figure S13. Principal Component Analysis (PCA) of wild, ornamental, and edible peaches.


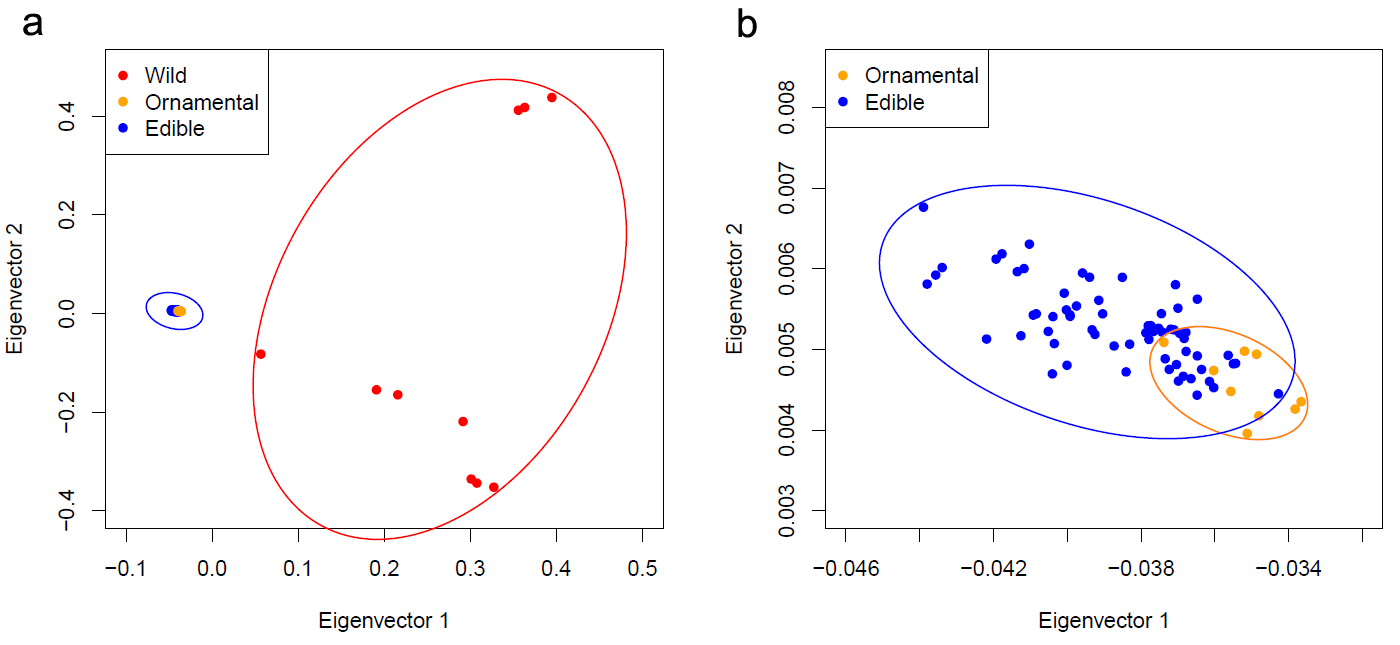


We used all the identified SNPs as markers to perform PCA analysis. In the two-dimensional eigenvector space, each point represents an independent accession of peach. Figure (b) provides a more detailed view of Figure (a). This analysis supports the concept that ornamental peach originated from edible peach or ancient cultivated peach; in other words, it suggests that an ancient group of edible and ornamental peach divided from wild peach.

#### Figure S14. Population structure of 84 peach accessions by FRAPPE.


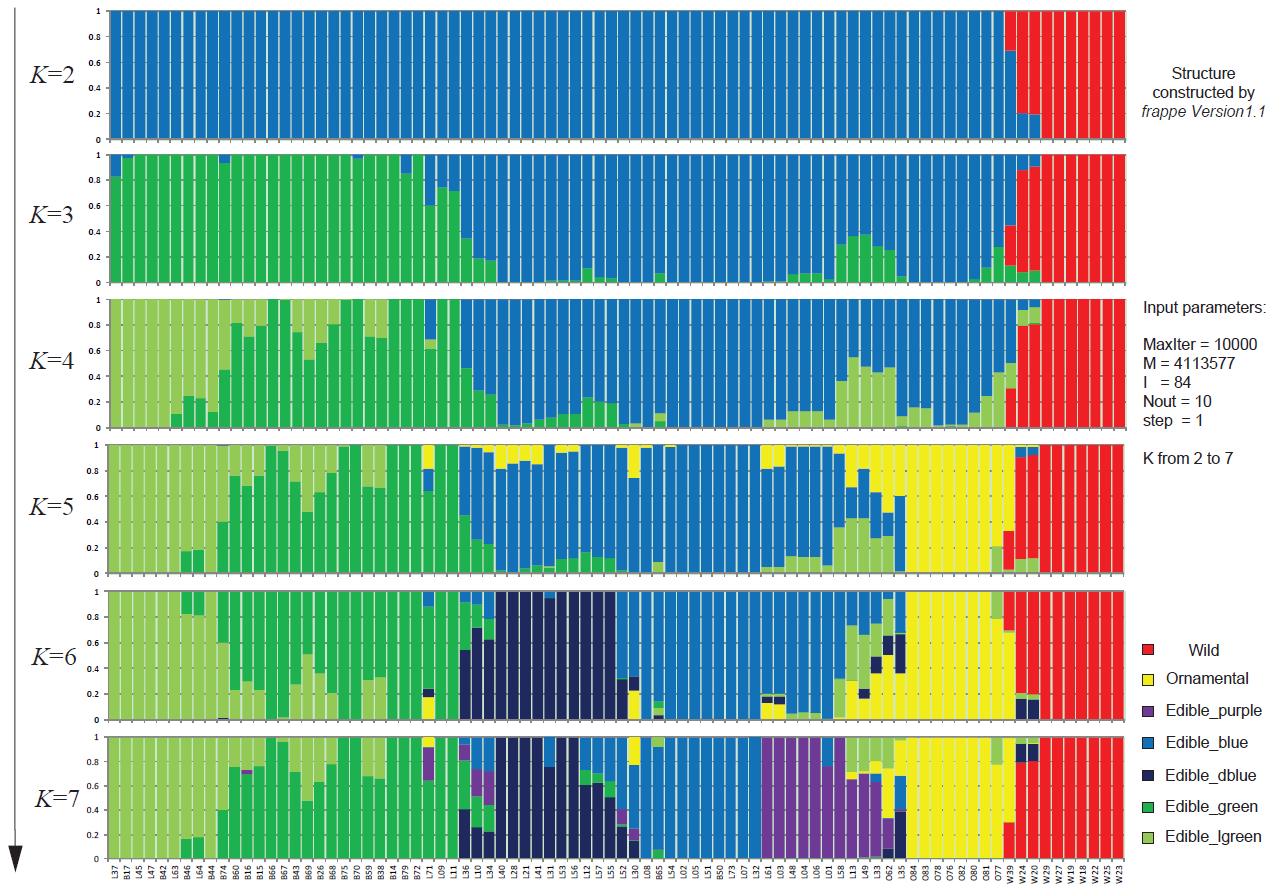


The population structure of the 84 peach accessions was constructed by FRAPPE (from K = 2 to K = 7) with all the genotype/SNPs. Each color represented a population, while K represented the number of populations. If we increased the input parameter K from 2 to N (N > 2), the two original populations were divided into N – 2 subgroups. The accessions list was ordered in accordance with the maximum-likelihood tree.

**Figure S15.** The selection judgment outline of the “region under selection” based on population structure.


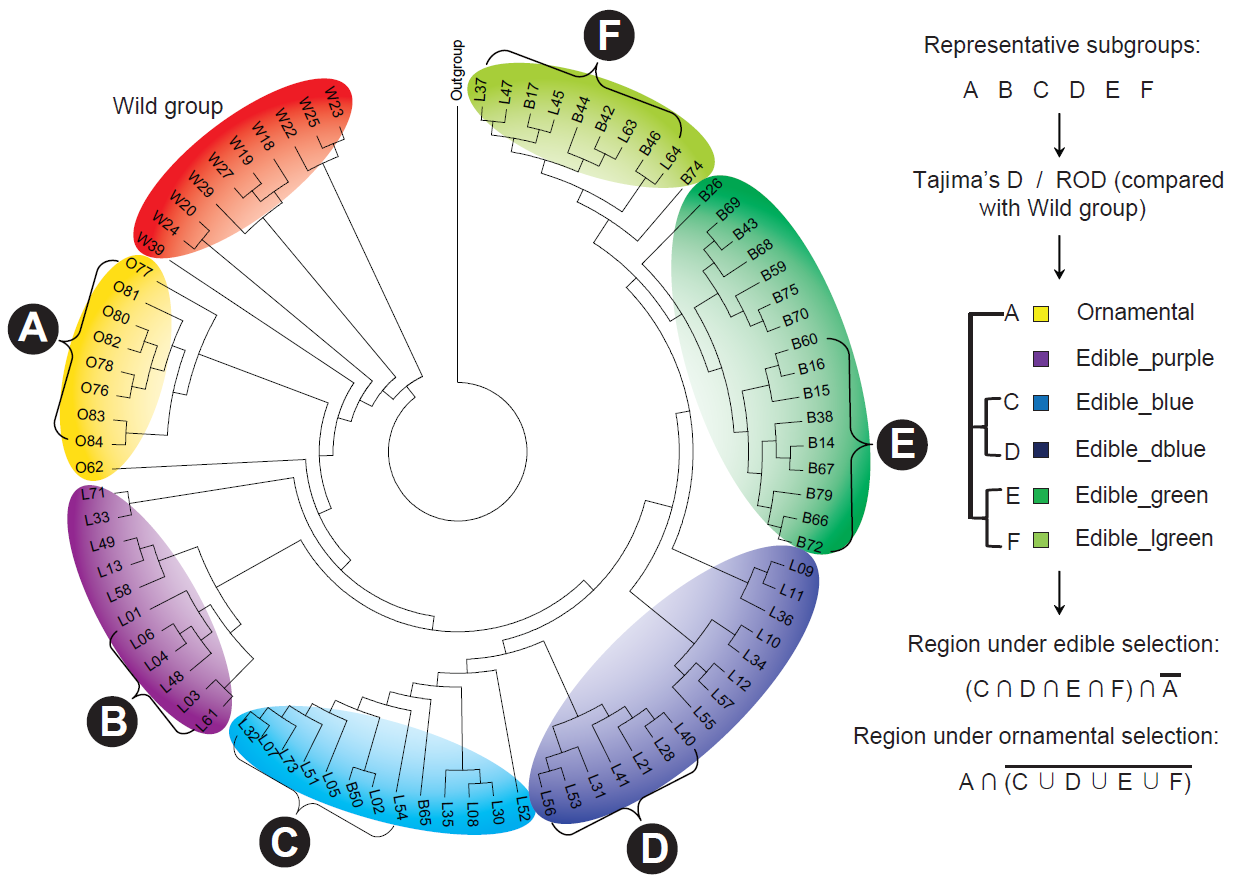


We picked six representative subgroups (A–F) from six main branches of cultivated peach. From these subgroups, we chose candidate regions under selection with the value of Tajima’s *D* outside the confidence limits (Neutral Mutation Range, Table 2). From the candidate regions in each subgroup, the judgment of the regions under edible selection could be defined as the candidate regions in the C, D, E, and F subgroups but not in the A subgroup; the judgment of the regions under ornamental selection could be defined as the candidate regions in the A subgroup but not in the C, D, E, and F subgroups.

#### Figure S16. *ROD* and *Fst* values in the regions under edible and ornamental selection.

**
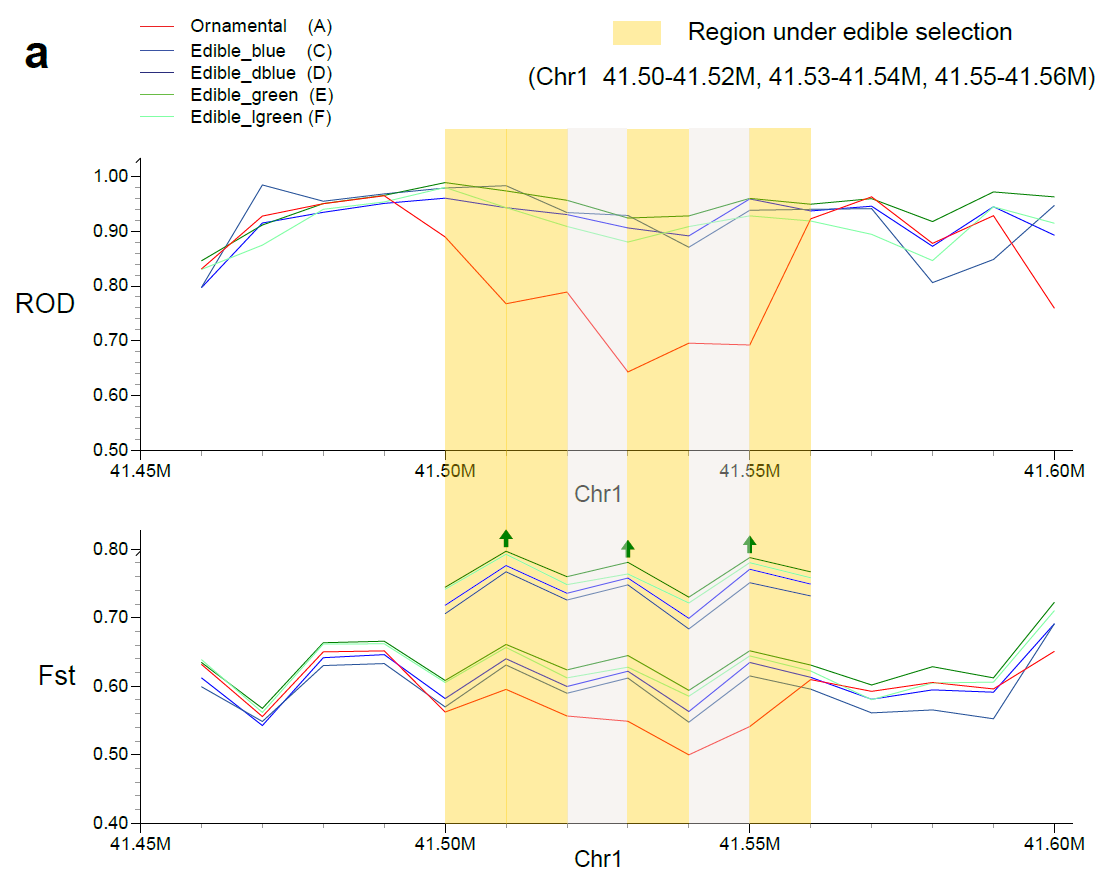
**

**
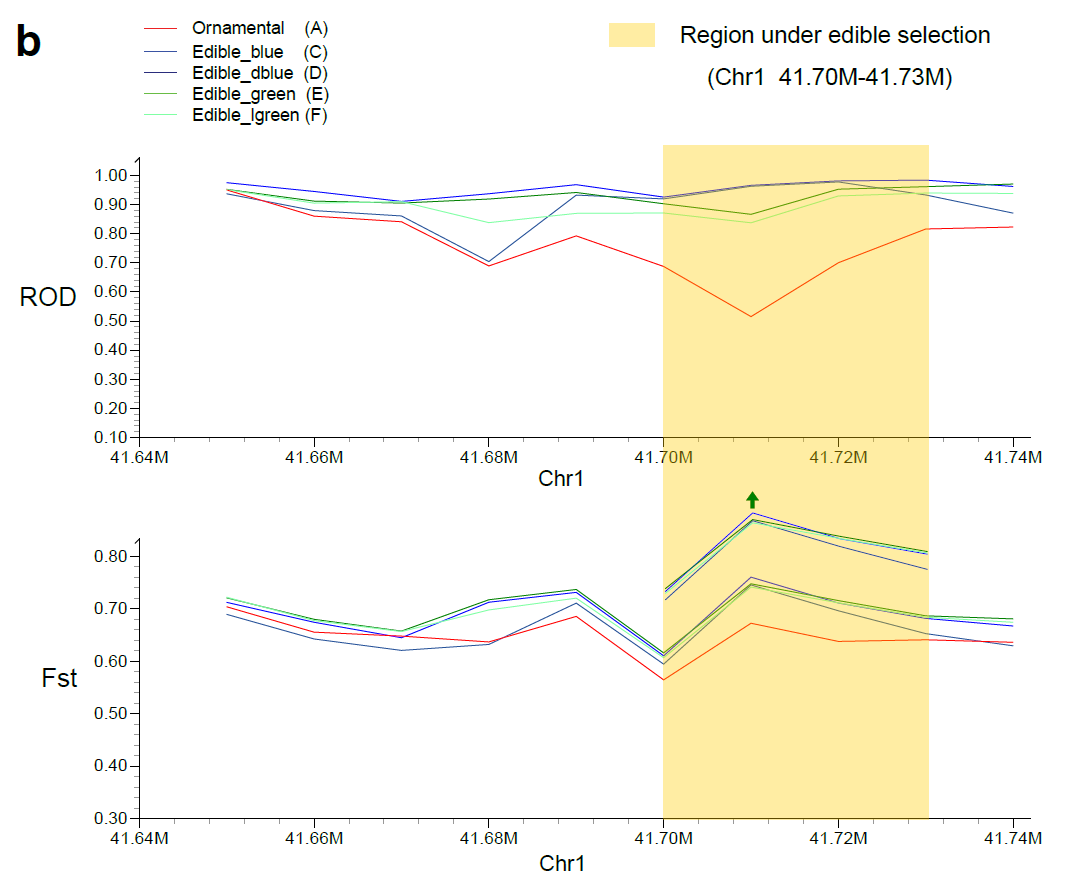
**


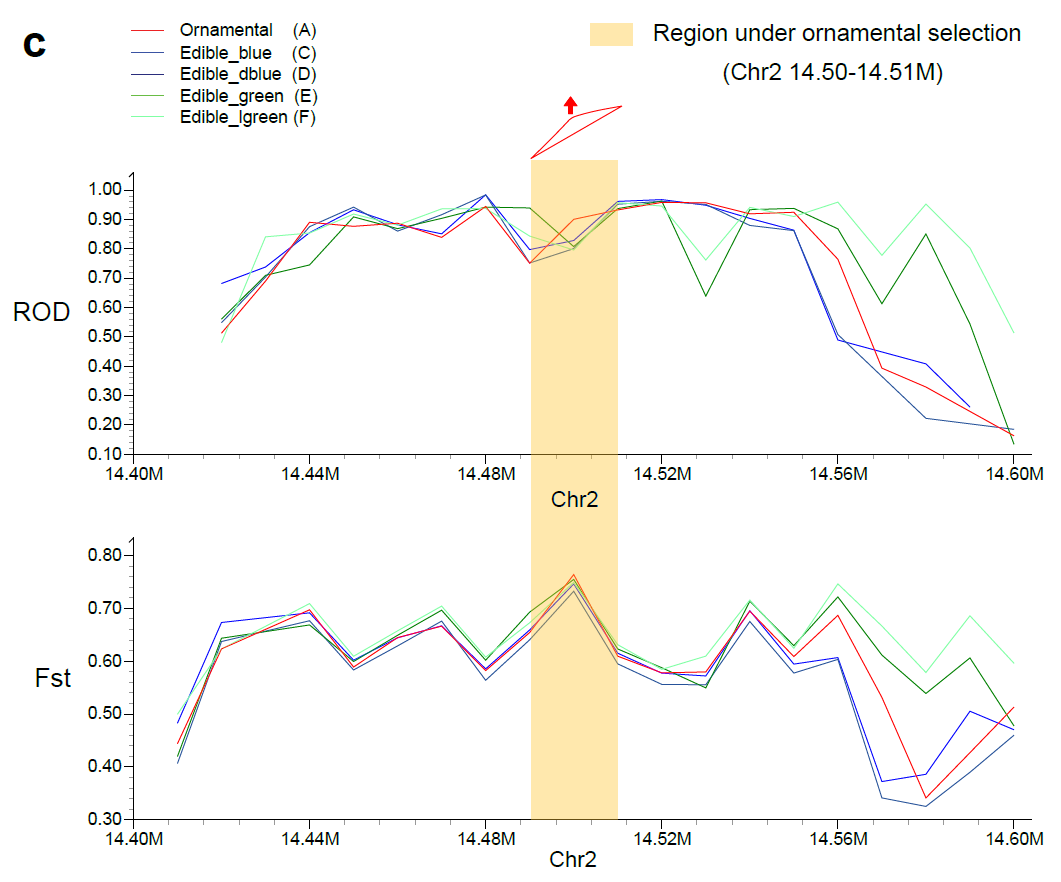

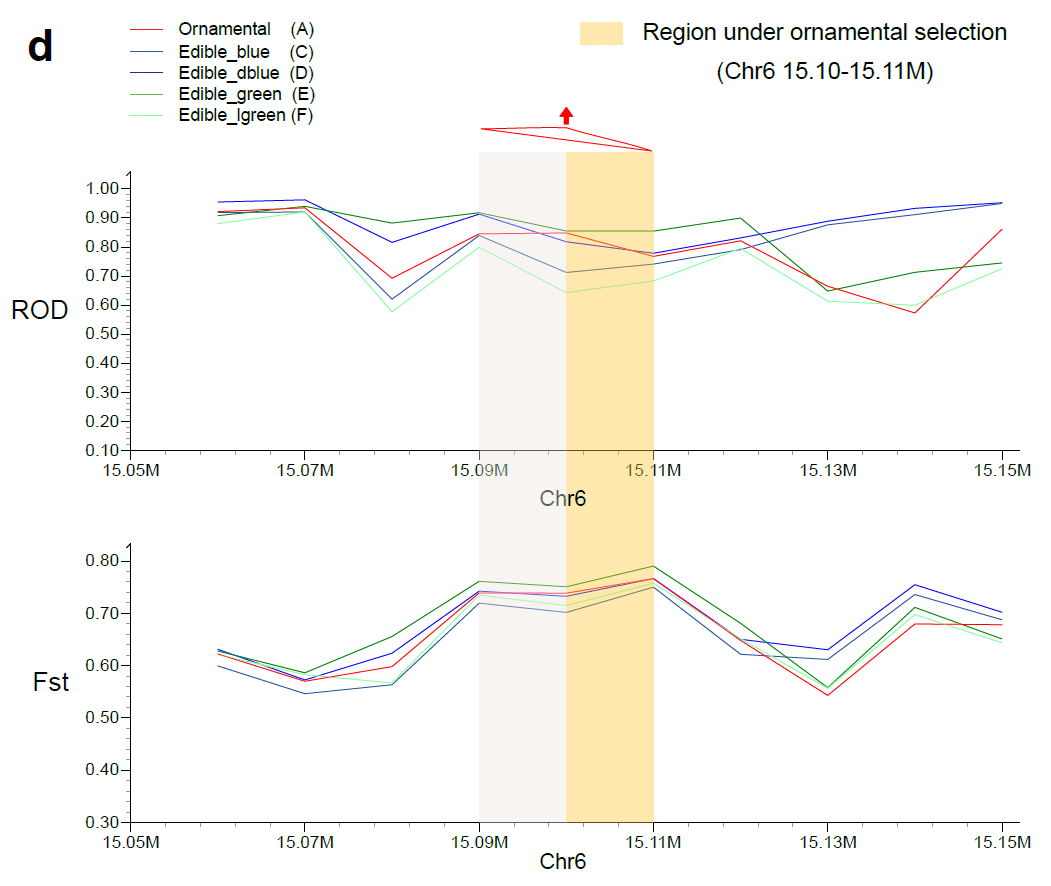


We calculated the *ROD* and *Fst* between each subgroup and the wild group, using a 10 kb window. In the region under edible selection (**a** and **b**), the values of *ROD* and *Fst* in four edible subgroups were all higher than in the ornamental subgroup. The huge difference between them shows obvious domestication difference or differentiated selection in this region. In the region under ornamental selection (**c** and **d**), the values of *ROD* and *Fst* in the ornamental subgroup were slightly higher than the other subgroups. These findings confirm our method for identifying the region under ornamental selection. The arrows show the signals of selection.

#### Figure S17. *R* (resistance) genes and the genes under selection in the chromosomes.

**a.**


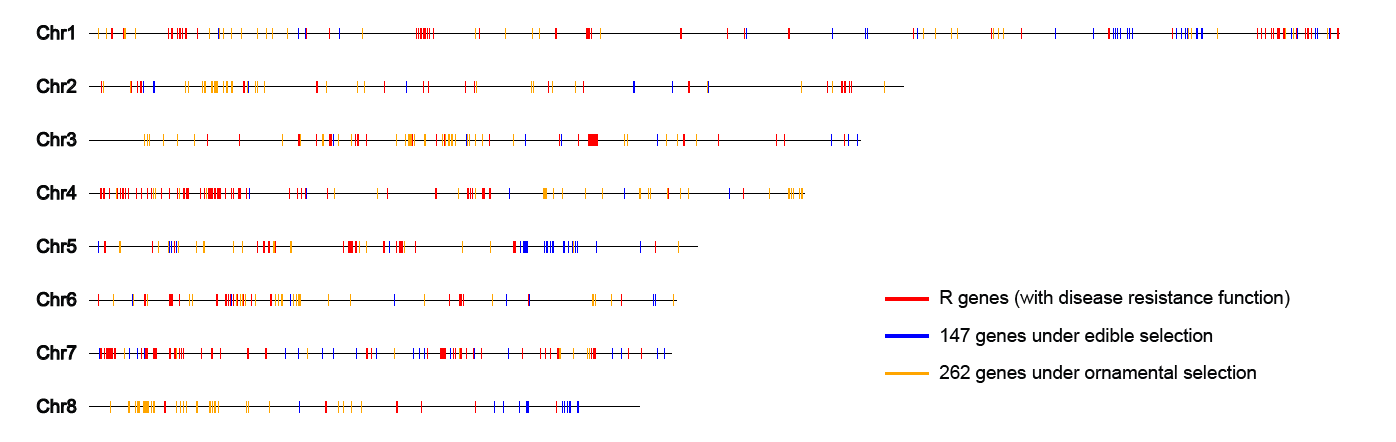


**b.**

**
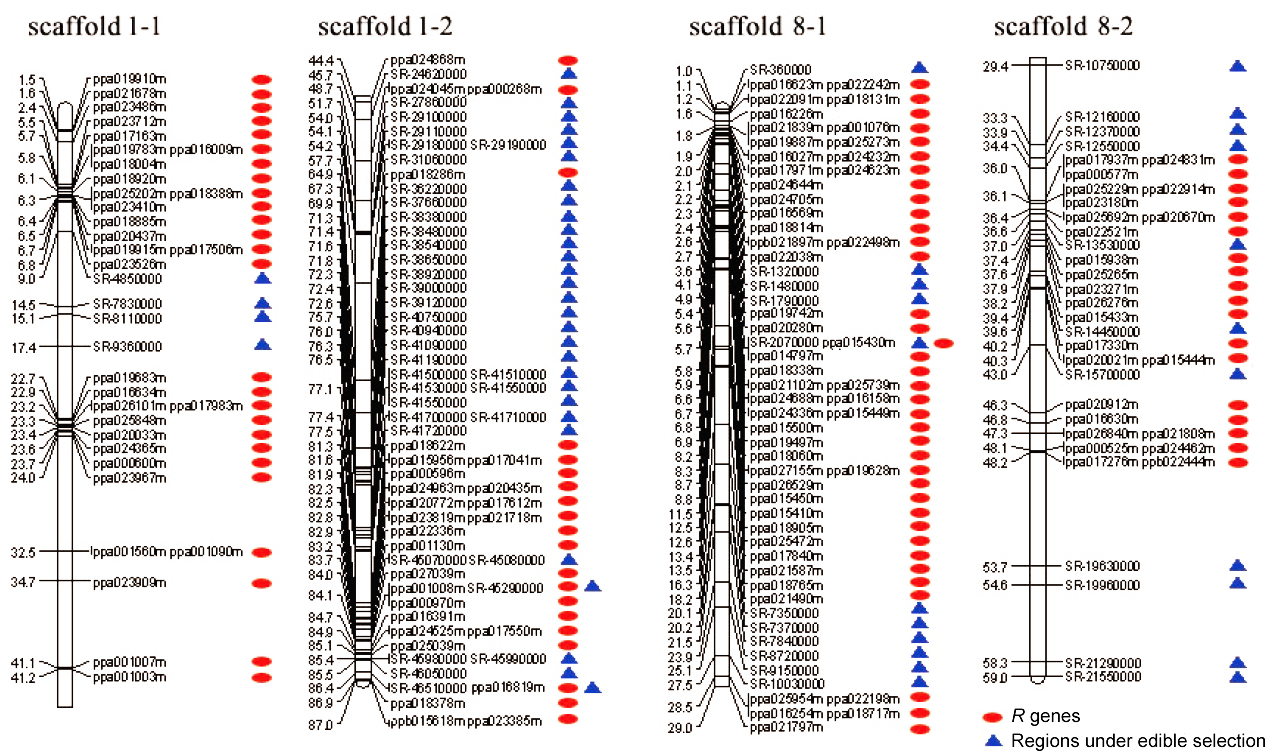
**

**(a)** The distribution of the *R* genes, including 147 genes under edible selection and 262 genes under ornamental selection along the chromosomes (**b)** The distribution of regions under edible selection and *R* genes in scaffold 1 and scaffold 8 of the genetic map. The regions under edible selection and the *R* genes were mostly distributed in different regions.

#### Figure S18. Gene ontology analysis of the genes under ornamental selection.


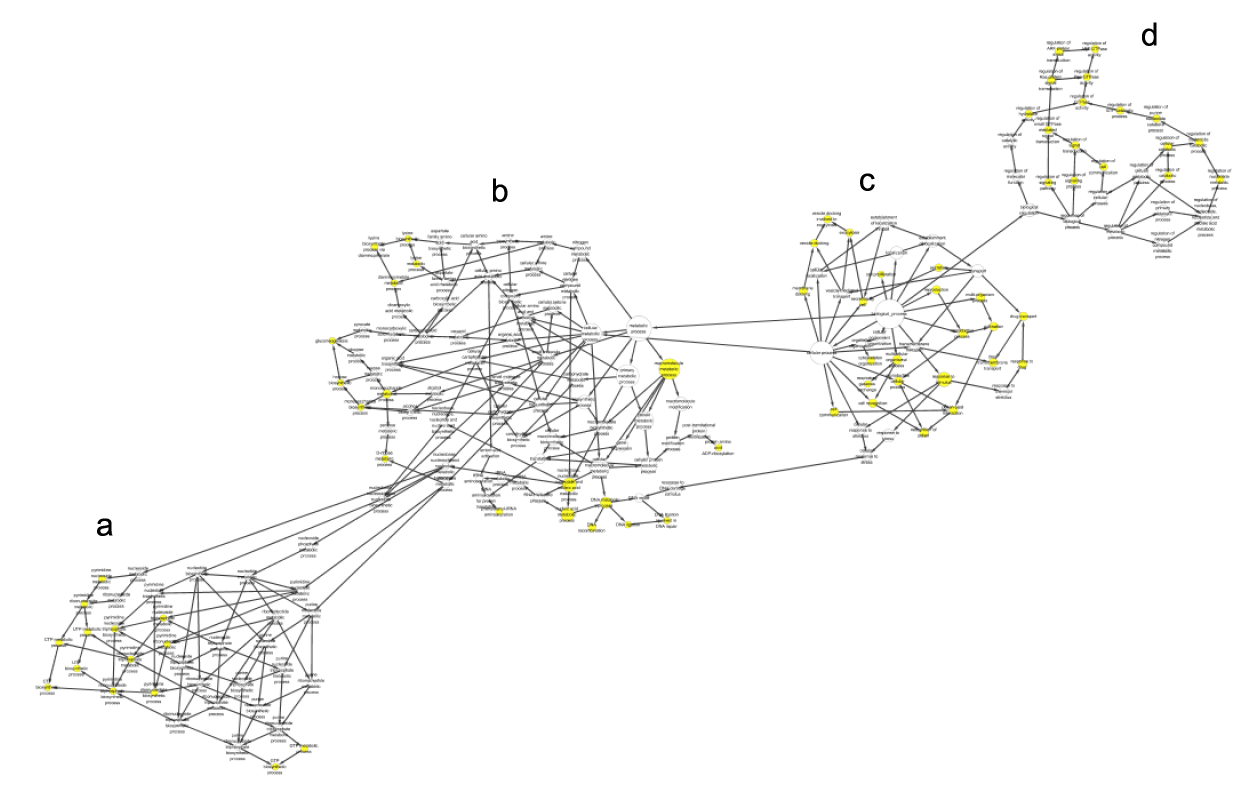


More Details:


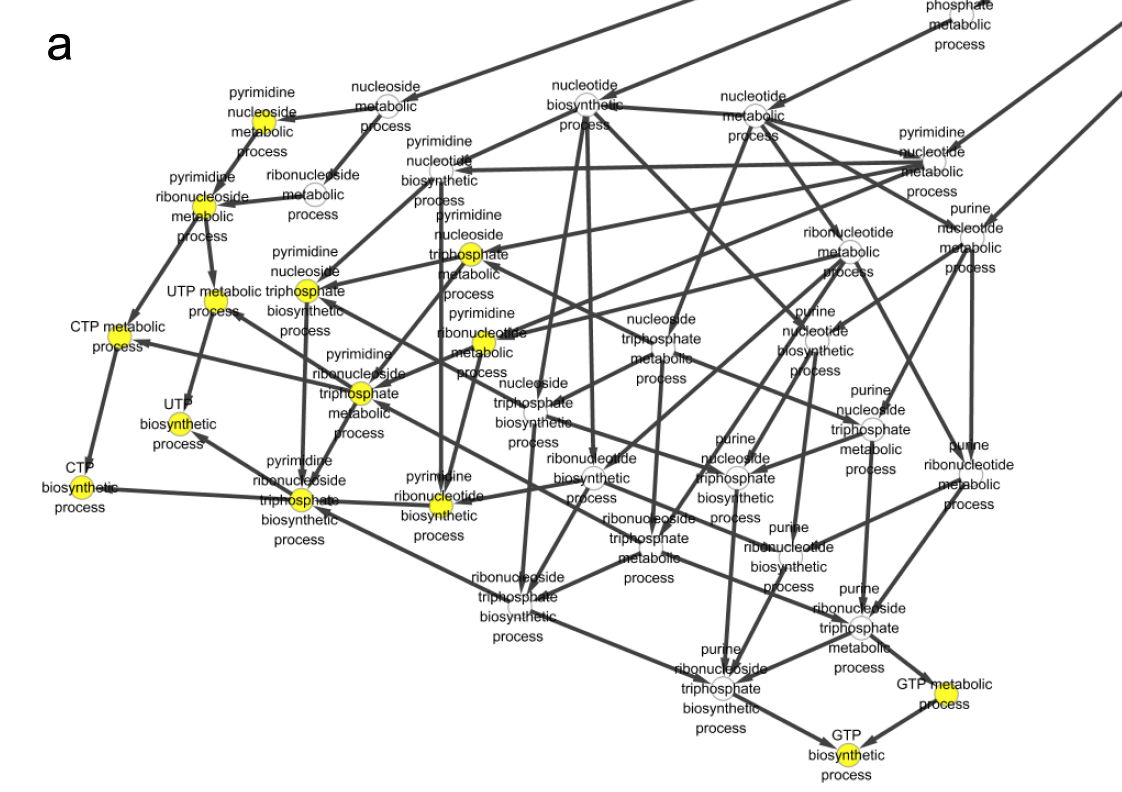


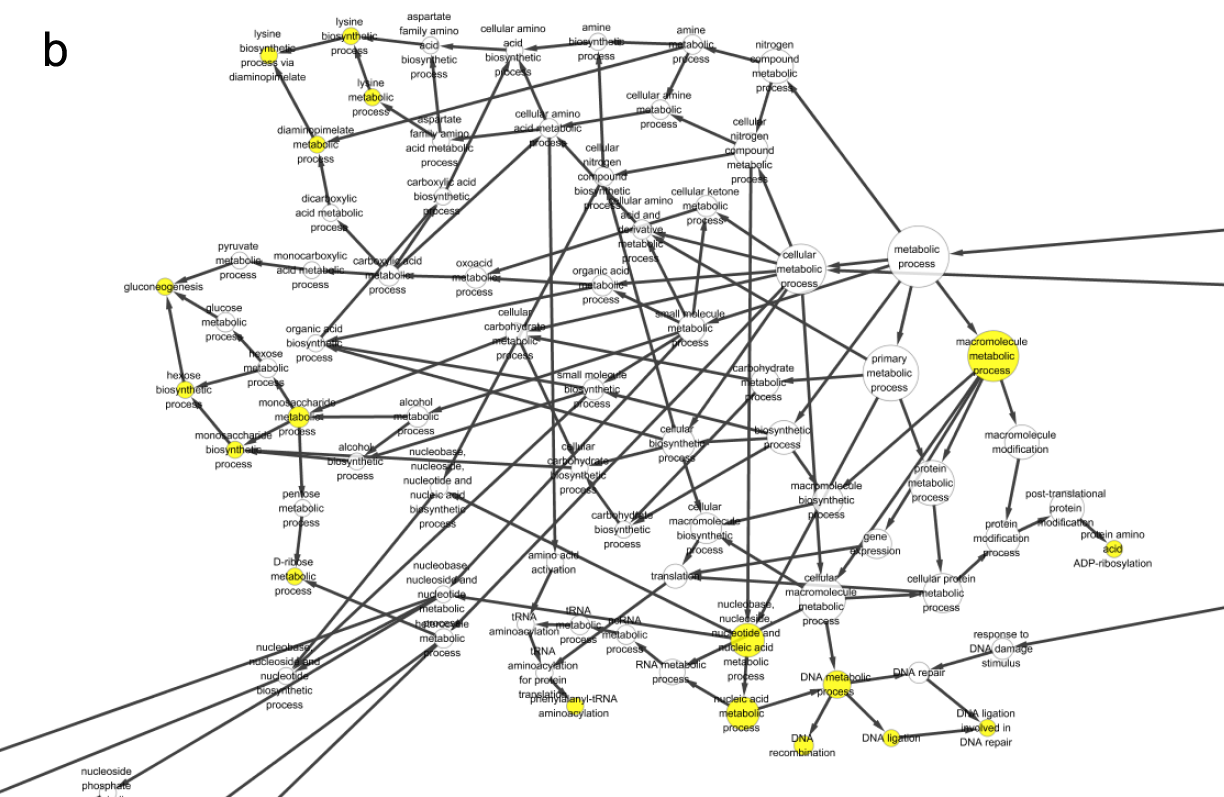


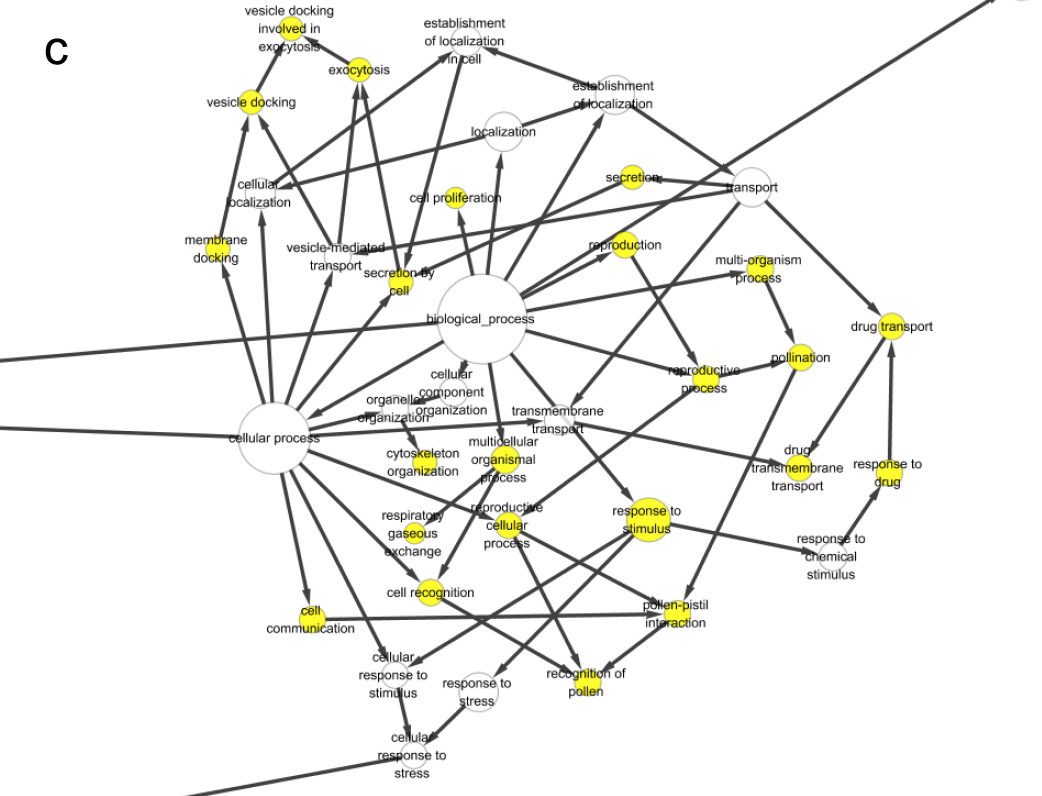


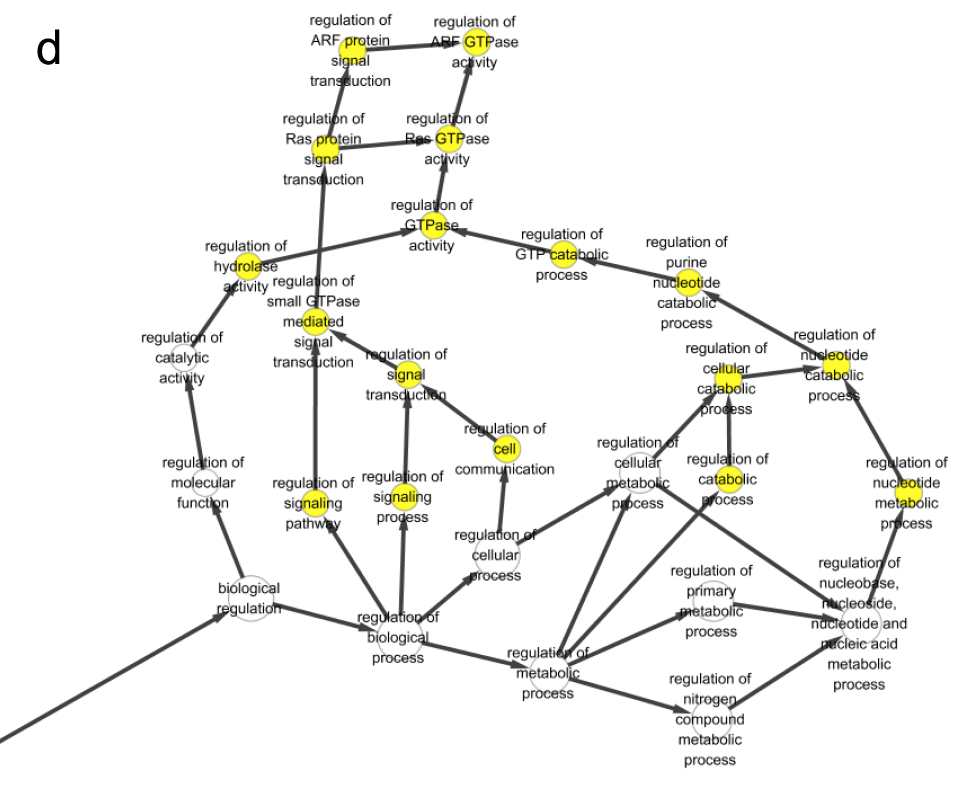


The figures were constructed using Cytoscape V2.8.0 with its plugin BINGO.

**Figure S19.** Gene ontology analysis of the genes under edible selection.


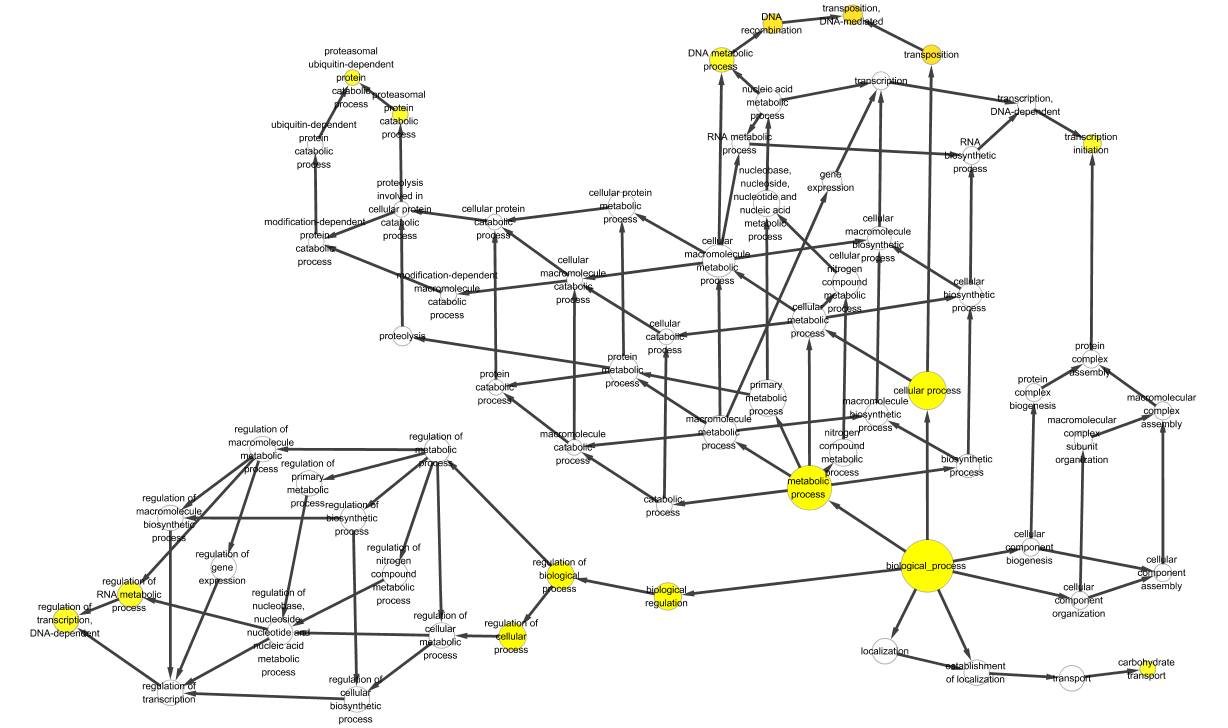


The figure was constructed using Cytoscape V2.8.0 with its plugin BINGO.

**Figure S20.** Linkage disequilibrium (LD) decays in different groups and subgroups.


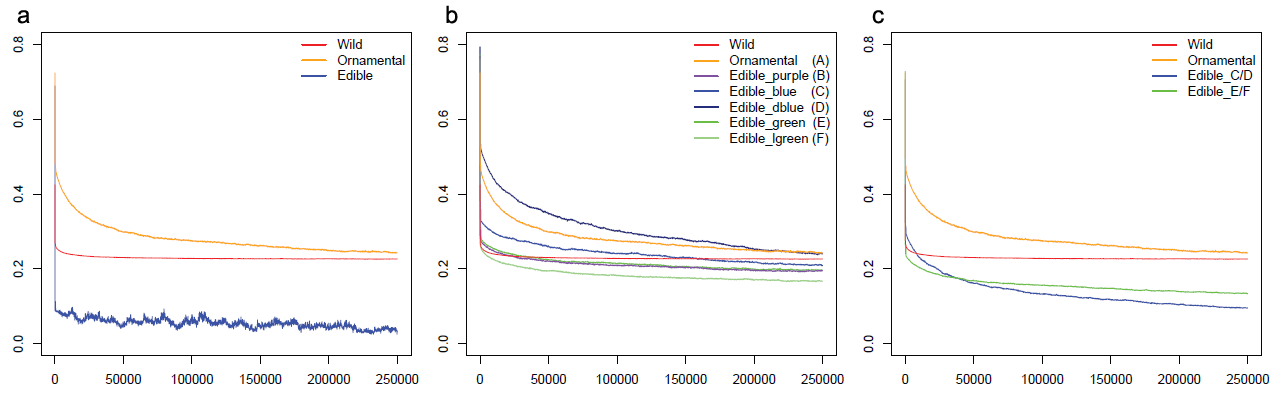


**a)** LD decays in wild, ornamental, and edible groups. **b)** LD decays in each subgroup, including the five subgroups of the edible group according to **Fig.2**. **c)** LD decays in four subgroups; Edible_C/D is composed of C and D, most of which were landraces; and Edible_E/F is composed of E and F, most of which were breeding lines.

#### Figure S21. Linkage disequilibrium (LD) analysis of two regions under selection.


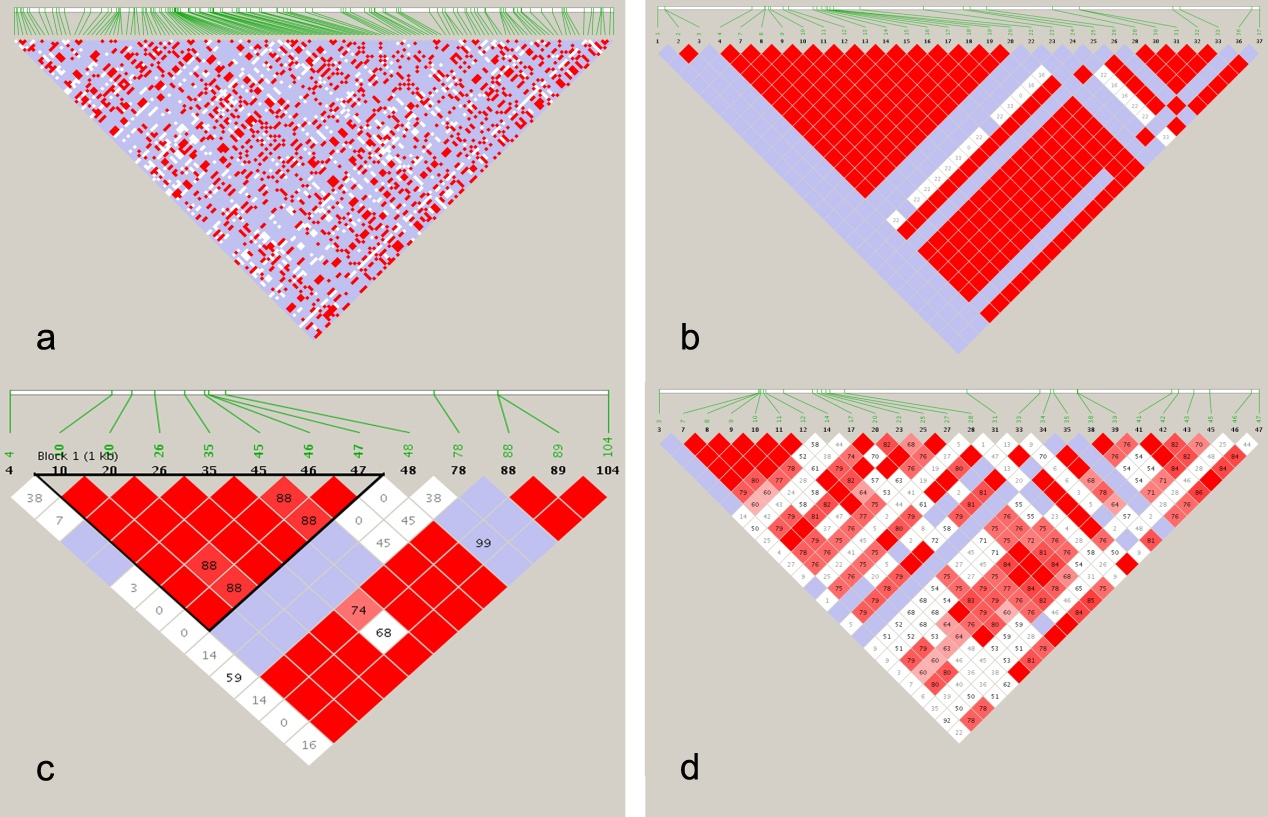


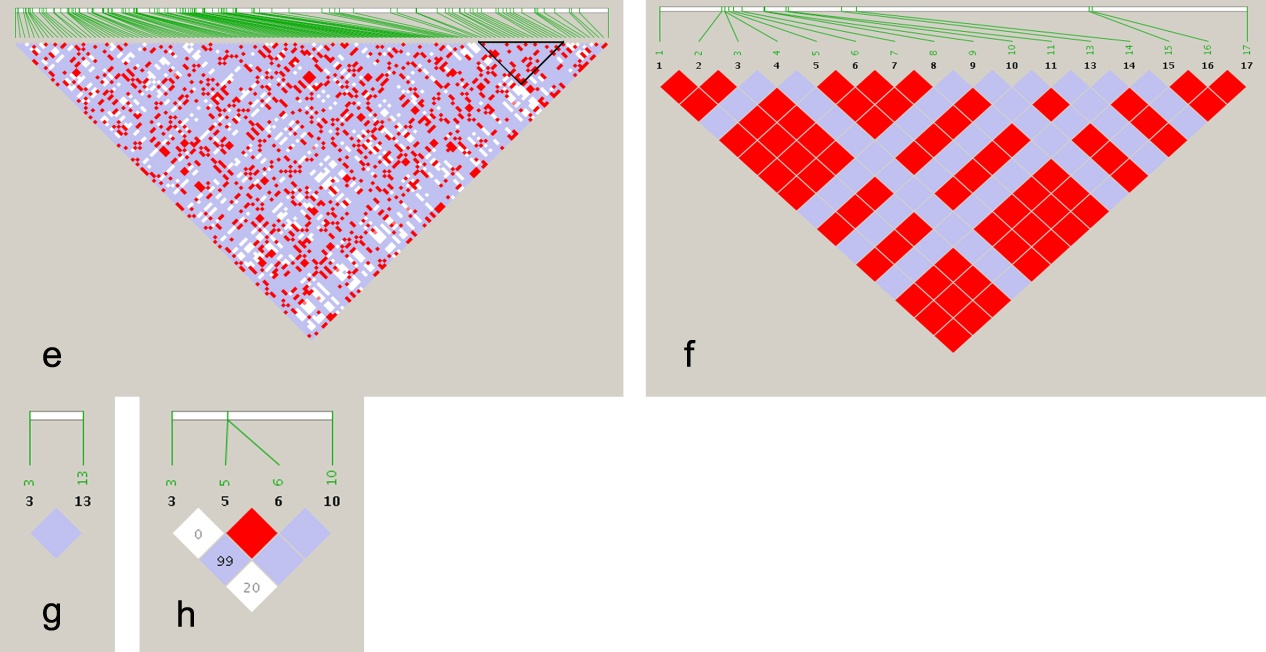


LD of two regions under edible selection. The **a–d** figures are the region Scaffold 4: 2420–2430 Kb (ppa009446m), and the **e–h** figures are the region Scaffold 5: 7880–7890 Kb (ppa000974m). Figures **a** and **e** depict the wild group, figures **b** and **f** depict the ornamental group, figures **c** and **g** were from landraces (edible_blue and edible_dblue subgroups), and figures **d** and **h** depict improved varieties (edible_green and edible_lgreen subgroups). Red and white spots indicate strong (r^2^ = 1) and weak (r^2^ = 0) LD, respectively.

#### Figure S22. Genome-wide association studies of flesh adhesion trait.

**
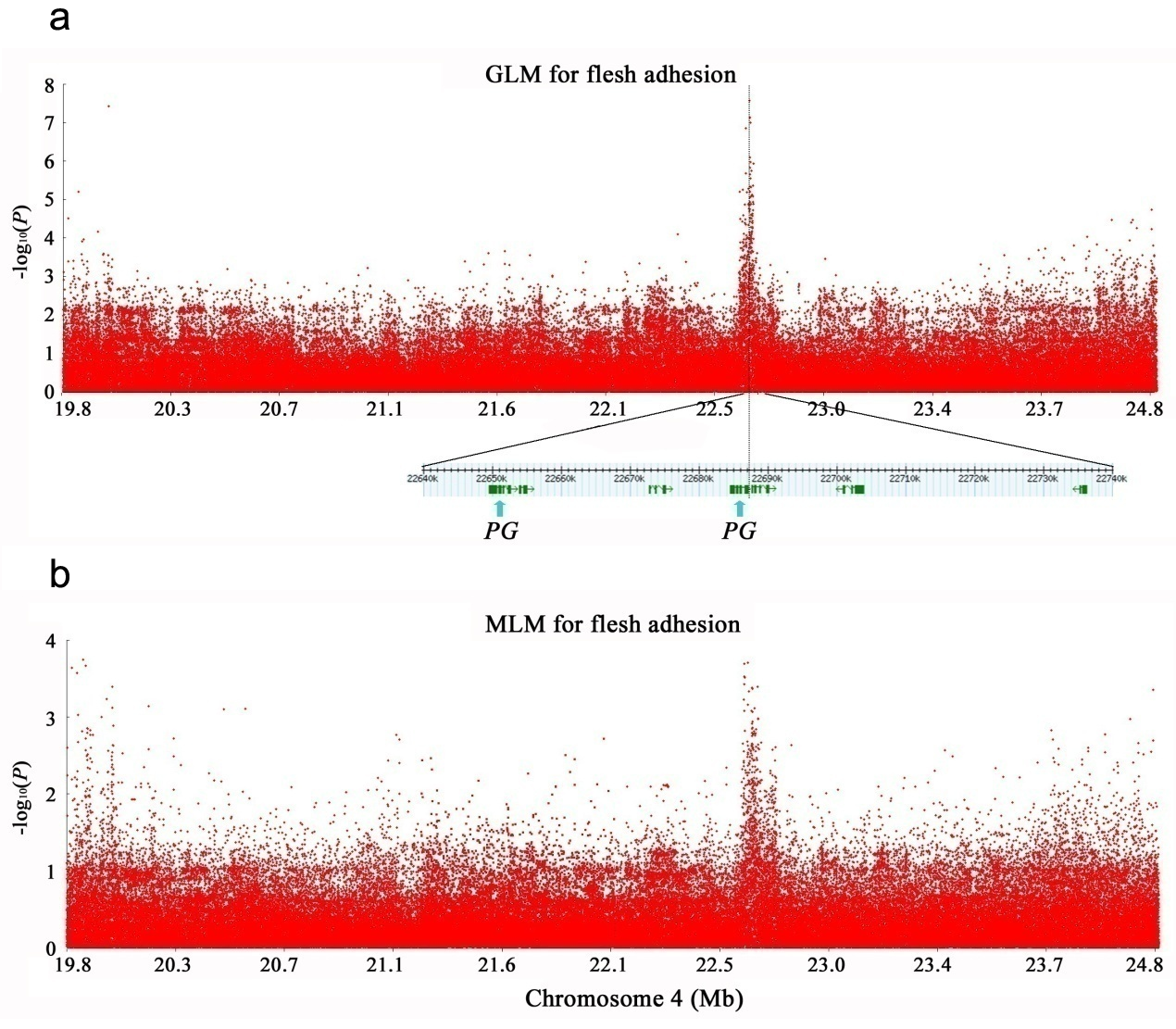
**

**(a)** Manhattan plots of the simple general linear model (GLM) for flesh adhesion. Negative log_10_-transformed P values from a genome-wide scan are plotted against position at 19.8–24.8 Mb on Scaffold 4 with 100,000 SNPs. (**b)** Manhattan plots of compressed mixed linear model (MLM) for flesh adhesion as in Figure **S21a**. This figure shows that the limited number of samples (84) can be used for genome-wide association studies to generate useful result, which may be a benefit from the low ratio of heterozygous SNPs in peach.
